# Preliminary evaluation of DeepSeek-R1 and GPT-5.3 in selected PET/CT clinical scenarios: patient preparation, report interpretation, and diagnostic reasoning

**DOI:** 10.3389/fmed.2026.1785671

**Published:** 2026-06-11

**Authors:** Runze Duan, Jing Pang, Lu Zheng, Ziyu Guo, Tianyue Li, Yanzhu Bian, Yujing Hu

**Affiliations:** 1 Hebei Medical University, Shijiazhuang, China; 2 Department of Nuclear Medicine, Hebei General Hospital, Shijiazhuang, China

**Keywords:** [18F]FDG PET/CT, artificial intelligence, Chatbot, DeepSeek-R1, GPT-5.3, patient communication

## Abstract

**Objective:**

To evaluate the performance of DeepSeek (R1 version), an open-source large language model, in three core clinical scenarios: answering patients’ common questions, interpreting PET/CT reports with follow-up inquiries, and diagnosing complex cases, and comparison with GPT-5.3, to verify the clinical applicability of DeepSeek-R1 as an alternative AI assistant.

**Methods:**

A total of 39 standardized tasks were assigned to both models, including responding to 15 frequently asked questions about [^18^F]FDG PET/CT, interpreting 12 anonymized reports of lung cancer and lymphoma (with follow-up inquiries regarding tumor staging or treatment), and providing primary and differential diagnoses for 10 difficult cases. Both models were accessed via their official platforms with default parameters, and all prompts and evaluation criteria were kept identical for cross-model comparison. Two senior nuclear medicine physicians independently rated the model responses using a 4-point standardized scale (assessing appropriateness, helpfulness, inter-trial consistency, and reference validity) and a binary scale for empathy; Cohen’s Kappa coefficient was used to evaluate inter-rater agreement. McNemar’s test was used to compare paired proportions of appropriateness, empathy, and response inconsistency between the two models.

**Results:**

Across the 39 tasks, DeepSeek-R1 achieved 94.9% appropriateness and 100% helpfulness. Specifically, 91.7% of responses to follow-up inquiries about tumor staging or treatment were rated empathetic. However, 7.7% of regenerated responses showed substantial inconsistencies, primarily in tumor staging, and only 37% of cited references were fully valid, with 11.1% being invalid. GPT-5.3 exhibited equivalent core performance to DeepSeek-R1 with 94.9% appropriateness and 100% helpfulness, a slightly lower substantial inconsistency rate (5.1%), favorable reference validity (33% fully valid, 7.4% invalid), but a notably lower empathy score (66.7%) for follow-up inquiries. McNemar tests showed identical appropriateness (*p* = 1.00) and no significant difference in inconsistency (*p* = 1.00, 95% CI 0.60–14.80) between models. DeepSeek-R1 had higher empathy, the difference was not significant (*p* = 0.25, 95% CI 0.09–0.66). For the 10 identical difficult cases, both models reached 10% primary diagnosis accuracy and 60% differential diagnosis accuracy.

**Conclusion:**

DeepSeek-R1 and GPT-5.3 have complementary strengths but similar reference hallucination issues and cannot replace clinicians. DeepSeek-R1 is a cost-effective auxiliary tool, with future optimization needed for consistency, diagnostic accuracy and reference validity.

## 1 Introduction

The growing demand for [^18^F]FDG PET/CT stems from its unique advantage of integrating metabolic and anatomical imaging, coupled with the public’s increasing health awareness (1, 2). However, this surge in clinical utilization has exacerbated the burden on nuclear medicine professionals, who face persistent staffing shortages and mounting workloads, thus creating an urgent need for efficiency-enhancing solutions (3, 4). Artificial intelligence (AI), particularly large language models (LLMs), has emerged as a promising candidate to address this gap (5–7).

Among such LLMs, DeepSeek distinguishes itself with open-source accessibility, cost effectiveness, and robust capabilities in medical reasoning and empathetic communication, highlighting its untapped potential to support specialized workflows in nuclear medicine (8, 9). In contrast, commercial state-of-the-art LLMs represented by GPT-5.3 possess advanced model architectures, optimized sampling parameters for stable output, and rigorous reference generation mechanisms, but are limited by closed-source access and high usage costs. However, DeepSeek may still make errors like other large language models such as Generative Pretrained Transformer (GPT), including incorrect answers and inconsistencies (10, 11). Additionally, the clinical application of AI tools remains limited by unresolved legal liabilities, ethical concerns, and inadequate emergency response capabilities, meaning AI cannot replace physicians in critical processes such as obtaining informed consent (12). Nevertheless, with appropriate clinical validation, AI is expected to alleviate the workload of nuclear medicine professionals by delivering standardized patient information, thereby improving patient compliance (13).

This pioneering study systematically evaluates DeepSeek-R1’s capabilities in [18F]FDG PET/CT patient communication, assessing its performance in answering common questions, interpreting reports, and diagnose difficult cases. In contrast, GPT-5.3 was also evaluated in parallel using the same 39 standardized tasks and evaluation criteria.

## 2 Materials and methods

### 2.1 Model selection and access

We use the R1 model released by DeepSeek on 20 January, 2025, for research purposes. The model was accessed via the DeepSeek platform^1^ on February 16 and 17, 2025. After entering the official website, enabling the deep thinking mode activates the R1 model. We use the GPT-5.3 model for research purposes. The model was accessed via the official platform.^2^ DeepSeek R1 model and GPT-5.3 Instant model with the default system prompt, default temperature, and default sampling parameters (see Supplementary Files 1, 2, 4).

Each question and PET/CT report were tested by independent chat sessions, with triple-run iterations per prompt. For comparison, the same 39 tasks were independently evaluated using GPT-5.3, a widely recognized state-of-the-art large language model. To ensure comparability, standardized prompt templates were used for all tasks, and the same prompts were applied to both DeepSeek-R1 and GPT-5.3. Each task was tested in a new independent chat session to avoid contextual carry-over effects. Three repeated trials were performed for each prompt under the default platform settings to assess test–retest variability under fixed prompt conditions. All prompts and evaluation criteria were identical for both models (prompts are provided in Supplementary Files 1, 2, and 4; evaluation criteria are listed in Table 1). Furthermore, both models analyzed 10 identical complex PET/CT cases to evaluate their performance in diagnosis and differential diagnosis.

**Table 1:** Criteria and categories used for rating.

Criterion	Description
Appropriateness	
1: Highly appropriate	Meeting standards of information given by medical staff in nuclear medicine department
2: Quite appropriate	Minor aspects incorrect or inconsistent
3: Quite inappropriate	Relevant aspects inconsistent
4: Highly inappropriate	Major aspects incorrect; potentially harmful
Helpfulness	
1: Very helpful	Comprehensive and likely to fully answer patient’s question
2: Quite helpful	Specific but lacking potentially helpful information
3: Quite unhelpful	Specific but lacking crucial information related to patient’s question
4: Clearly unhelpful	Unspecific and lacking crucial information
Empathetic	
Yes	Shows humanlike empathy
No	Is neutral and shows no empathy
Inconsistent between trials	
1: Irrelevant	Differences only in wording, style, or layout
2: Minor	Differences in content of response but none relevant to main content required to answer patient’s question
3: Major	Some differences relevant to main content
4: Incompatible	Responses incompatible with each other
Validity of references	
1: Fully valid	Appropriate, identifiable, and accessible source
2: Appropriate but outdated	Appropriate reference but outdated uniform resource locator or only generic references
3: Appropriate, incorrectly cited, but possible to find	Appropriate reference with incorrect bibliographic data but still possible to find
4: Invalid	Invalid reference that cannot be found (hallucinations)

### 2.2 Rating process

Two nuclear medicine specialists with more than 10 years of clinical experience independently evaluated the responses of both DeepSeek-R1 and GPT-5.3 based on the standardized criteria outlined in Table 1 (11). The raters were blinded to model identity, and disagreements were resolved by adjudication from a third senior physician. A 4-point ordinal scale was adopted for assessing appropriateness, helpfulness, inconsistency between trials, and validity of references to ensure definitive ratings and facilitate dichotomous classification of response quality; Empathy assessment was restricted to follow-up questions related to PET/CT reports, and a binary (yes/no) rating system was utilized to minimize subjective variability inherent in graded scales. The core dimensions in Table 1 were independently evaluated by all two raters. Inter-rater reliability was quantified using Cohen‘s Kappa coefficient (*κ*) to assess agreement across the two evaluators. Evaluators were blinded to model identity throughout the assessment to minimize bias.

### 2.3 Generating questions and PET/CT reports

The tasks were divided into three parts: (1) Patient questions: Fifteen frequently asked questions regarding [^18^F]FDG PET/CT examinations were systematically compiled (Tables 2, 3, Q1–Q15). These questions were formulated using simple, non-technical language to reflect typical patient inquiries (e.g., “Can people with metal implants undergo PET/CT?”) (2). Report interpretation and follow-up question response: Twelve anonymized PET/CT reports were collected, including six for lung cancer and six for lymphoma. Each report was comprehensively interpreted using the prompt “Please explain my PET report,” followed by answering a related question about tumour staging or therapeutic management (Tables 2, 3, R1–R12). To maintain objectivity, staging information was removed from all 12 reports (3). Difficult case analysis: Ten difficult cases, which were initially misdiagnosed by our department but later confirmed by definitive pathological results, were included in the study (Table 4, R1–R10). The 10 cases were selected by the corresponding author before any comparative analysis was performed. The original diagnoses and conclusions were manually removed. The model was prompted as follows: “Provide a primary diagnosis and differential diagnoses based on the report and clinical history.” This sample size aligns with the study’s pilot scope, which prioritized identifying DeepSeek’s core strengths and critical limitations for nuclear medicine practice over large-scale validation. Inferences are restricted to adult [^18^F]FDG PET/CT workflows in nuclear medicine, specifically lung cancer/lymphoma report interpretation, routine patient communication, and complex case analysis, with findings guiding further testing in larger, multi-center cohorts.

**Table 2:** 15 questions and 12 reports submitted to DeepSeek and majority rating.

Question/report	Description	Appropriate	Helpful	Inconsistent
Q1	Why do I need to fast in the morning when I undergo [^18^F]FDG PET/CT?	1	1	2
Q2	The purpose of drinking more water before undergoing PET/CT	1	1	2
Q3	Is minimal conversation recommended post-[^18^F]FDG injection?	1	1	1
Q4	Can pregnant women or lactating women undergo PET/CT?	1	1	2
Q5	Can people with metal implants undergo PET/CT?	1	1	1
Q6	Why do I need to have PET/CT scan 1 hour after the first one?	2	2	1
Q7	Will family members accompanying me be affected by the radiation? How to avoid?	1	1	1
Q8	Is [^18^F]FDG PET/CT only used for diseases like malignant tumors?	1	1	1
Q9	Does hypermetabolism on [^18^F]FDG PET/CT mean tumor?	2	1	1
Q10	How soon can I work and live normally after PET/CT? Precautions	1	1	2
Q11	Can I perform PET/CT after just having a barium meal yesterday?	1	1	1
Q12	I am claustrophobic, can I take [^18^F]FDG PET/CT?	1	1	2
Q13	Can diabetics undergo FDG PET/CT? requirements for insulin?	1	1	2
Q14	Does patient with epilepsy stop related medication before [^18^F]FDG PET/CT?	1	1	1
Q15	Can patient with renal insufficiency undergo PET/CT?	1	1	2
R1	lymphoma stage I	1	1	2
R1Q1	What’s my life expectancy?	1	1	1
R2	lymphoma stage II	1	1	2
R2Q1	What is the stage of my lymphoma?	1	1	1
R3	lymphoma stage III	1	1	1
R3Q1	How should my lymphoma be treated?	1	2	1
R4	lymphoma stage IV	2	1	1
R4Q1	What is the stage of my lymphoma?	1	1	1
R5	stage IA lung cancer	1	1	1
R5Q1	What’s my life expectancy?	1	1	2
R6	stage IB lung cancer	1	1	1
R6Q1	What is the stage of my lung cancer?	3	2	2
R7	stage IIA lung cancer	1	1	2
R7Q1	How should my lung cancer be treated?	1	1	1
R8	stage IIB lung cancer	1	1	2
R8Q1	What is the stage of my lung cancer?	1	1	2
R9	stage IIIA lung cancer	1	1	1
R9Q1	How should my lung cancer be treated?	1	1	2
R10	stage IIIB lung cancer	1	1	1
R10Q1	What is the stage of my lung cancer?	3	2	1
R11	stage IVA lung cancer	1	1	1
R11Q1	What is the stage of my lung cancer?	2	1	1
R12	stage IVB lung cancer	1	1	1
Q12Q1	What’s my life expectancy?	1	1	2

**Table 3:** 15 questions and 12 reports submitted to GPT-5.3 and majority rating.

Question/report	Description	Appropriate	Helpful	Inconsistent
Q1	Why do I need to fast in the morning when I undergo [^18^F]FDG PET/CT?	1	1	2
Q2	The purpose of drinking more water before undergoing PET/CT	1	1	2
Q3	Is minimal conversation recommended post-[^18^F]FDG injection?	1	1	1
Q4	Can pregnant women or lactating women undergo PET/CT?	1	1	1
Q5	Can people with metal implants undergo PET/CT?	1	1	1
Q6	Why do I need to have PET/CT scan 1 hour after the first one?	2	1	1
Q7	Will family members accompanying me be affected by the radiation? How to avoid?	1	1	2
Q8	Is [^18^F]FDG PET/CT only used for diseases like malignant tumors?	1	1	1
Q9	Does hypermetabolism on [^18^F]FDG PET/CT mean tumor?	1	1	1
Q10	How soon can I work and live normally after PET/CT? Precautions	1	1	2
Q11	Can I perform PET/CT after just having a barium meal yesterday?	1	1	2
Q12	I am claustrophobic, can I take [^18^F]FDG PET/CT?	1	1	2
Q13	Can diabetics undergo FDG PET/CT? requirements for insulin?	1	1	2
Q14	Does patient with epilepsy stop related medication before [^18^F]FDG PET/CT?	1	1	1
Q15	Can patient with renal insufficiency undergo PET/CT?	1	1	2
R1	lymphoma stage I	1	1	1
R1Q1	What’s my life expectancy?	1	1	2
R2	lymphoma stage II	1	1	1
R2Q1	What is the stage of my lymphoma?	1	1	1
R3	lymphoma stage III	1	1	1
R3Q1	How should my lymphoma be treated?	1	1	2
R4	lymphoma stage IV	2	1	2
R4Q1	What is the stage of my lymphoma?	1	1	2
R5	stage IA lung cancer	1	1	1
R5Q1	What’s my life expectancy?	1	1	1
R6	stage IB lung cancer	2	1	2
R6Q1	What is the stage of my lung cancer?	3	2	1
R7	stage IIA lung cancer	1	1	1
R7Q1	How should my lung cancer be treated?	1	1	2
R8	stage IIB lung cancer	1	1	2
R8Q1	What is the stage of my lung cancer?	1	1	3
R9	stage IIIA lung cancer	1	1	1
R9Q1	How should my lung cancer be treated?	1	1	2
R10	stage IIIB lung cancer	1	1	1
R10Q1	What is the stage of my lung cancer?	3	2	1
R11	stage IVA lung cancer	1	1	1
R11Q1	What is the stage of my lung cancer?	1	1	1
R12	stage IVB lung cancer	1	1	1
Q12Q1	What’s my life expectancy?	1	1	1

**Table 4:** DeepSeek-R1 and GPT-5.3’s responses to 10 difficult reports: “√“indicates that the answer is consistent with the pathological result.

Report	Description	Primary diagnosis	Differential diagnosis	None
R1	pseudomyxoma peritonei			√
R2	Lung complex small cell carcinoma (small cell carcinoma + large cell neuroendocrine carcinoma)	√		
R3	Sarcoidosis		√	
R4	Localized interstitial fibrosis of pancreatic tissue with chronic inflammation		√	
R5	Chronic inflammation of the lung tissue		√	
R6	Epithelioid angiosarcoma		√	
R7	Infectious mononucleosis		√	
R8	Lymphadenitis			√
R9	histiocytic necrotizing lymphadenitis		√	
R10	Occult lung cancer with lymph node metastasis			√

Primary diagnosis: Indicates that the primary diagnosis provided by DeepSeek aligns with the pathological result. Differential diagnosis: Indicates that DeepSeek’s differential diagnosis recommendations include content consistent with the pathological result. None: Indicates that DeepSeek failed to provide an effective diagnosis/differential diagnosis.

All the above cases were obtained from the Department of Nuclear Medicine, Hebei General Hospital. All the reports were translated from Chinese by DeepL and subsequently verified for accuracy. This study used only anonymized clinical reports with all personal identifiers removed. It was conducted in accordance with institutional ethical guidelines. Ethical approval and informed consent were waived because only de-identified retrospective data were used without affecting patient care or privacy.

### 2.4 Statistical analysis

Final rating for each task was determined by majority vote. When there was disagreement between the two raters on any rating item, the final rating was determined by adjudication from a third senior nuclear medicine physician with >15 years of clinical experience to ensure rating accuracy. Statistical analyses were performed using SPSS for Windows (Version 27.0.1). McNemar’s test was used to compare paired proportions of appropriateness, empathy, and response inconsistency between the two models. Cohen‘s Kappa was calculated to evaluate the inter-rater agreement between the two researchers, The *κ* values for the two models (R1 and GPT-5.3, respectively) were 0.77 and 0.88 for appropriateness, 0.77 and 1.00 for helpfulness, 0.95 and 0.85 for between-trial inconsistency, 1.00 and 1.00 for empathy, and 0.88 and 0.88 for reference validity, indicating substantial to near-perfect agreement.

## 3 Results

**Figure 1:**
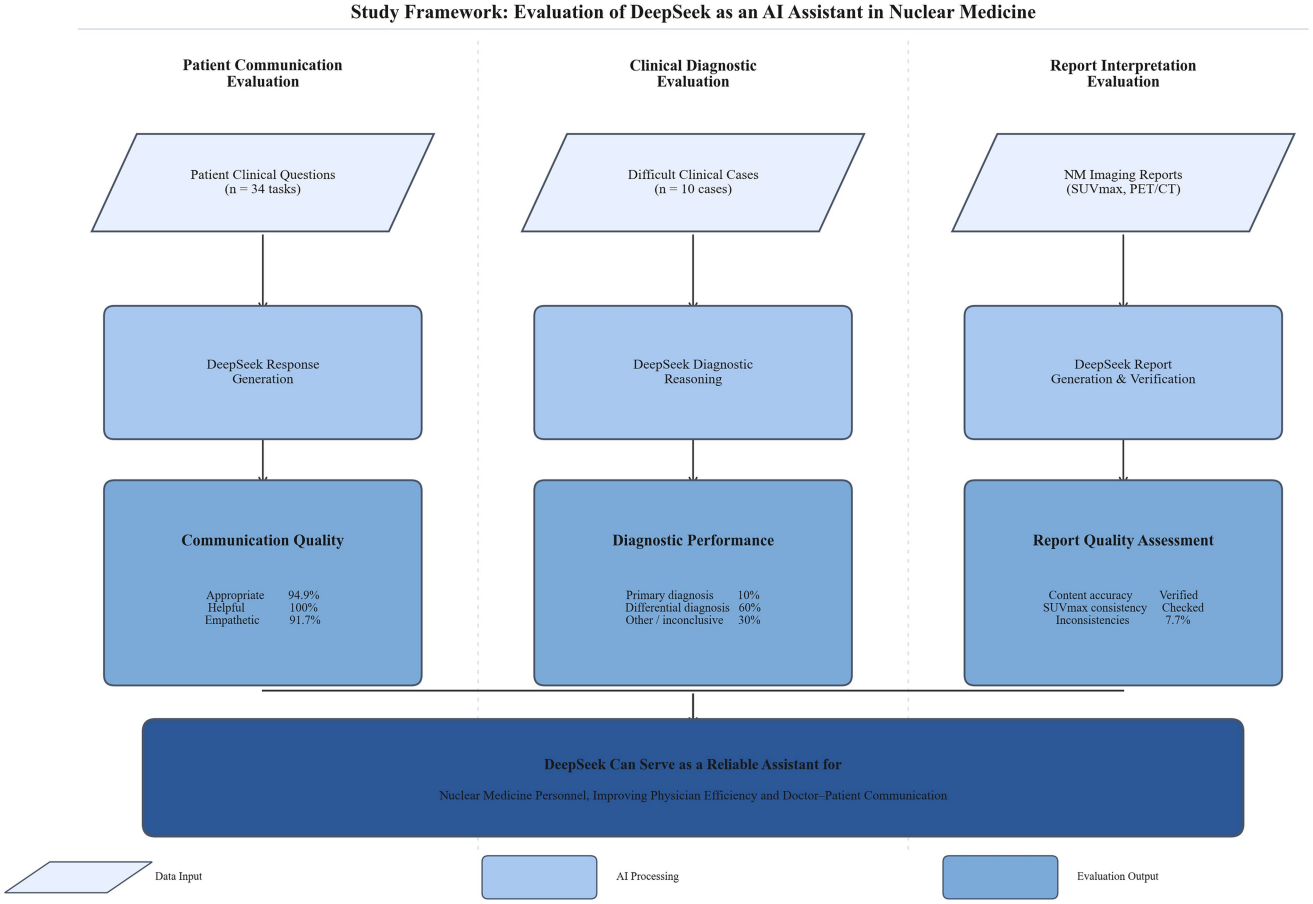
Study framework for evaluating DeepSeek as an artificial intelligence assistant in nuclear medicine. The evaluation consisted of three domains: (1) patient communication evaluation using 34 patient clinical questions to assess response quality, including appropriateness, helpfulness, and empathy; (2) clinical diagnostic evaluation using 10 difficult clinical cases to assess diagnostic reasoning performance, including primary diagnosis and differential diagnosis capability; and (3) report interpretation evaluation using nuclear medicine imaging reports (including SUVmax and PET/CT reports) to assess report generation, verification, and consistency checking. Overall, the framework aimed to determine whether DeepSeek could serve as a reliable assistant for nuclear medicine personnel by improving physician efficiency and doctor–patient communication.

All questions, PET/CT reports, and DeepSeek-R1 responses can be found in Supplementary Files 1, 2, 3. The responses of GPT-5.3 in Supplementary Files 4, 5. The process is Figure 1.

### 3.1 Rating of two models responses

**Figure 2:**
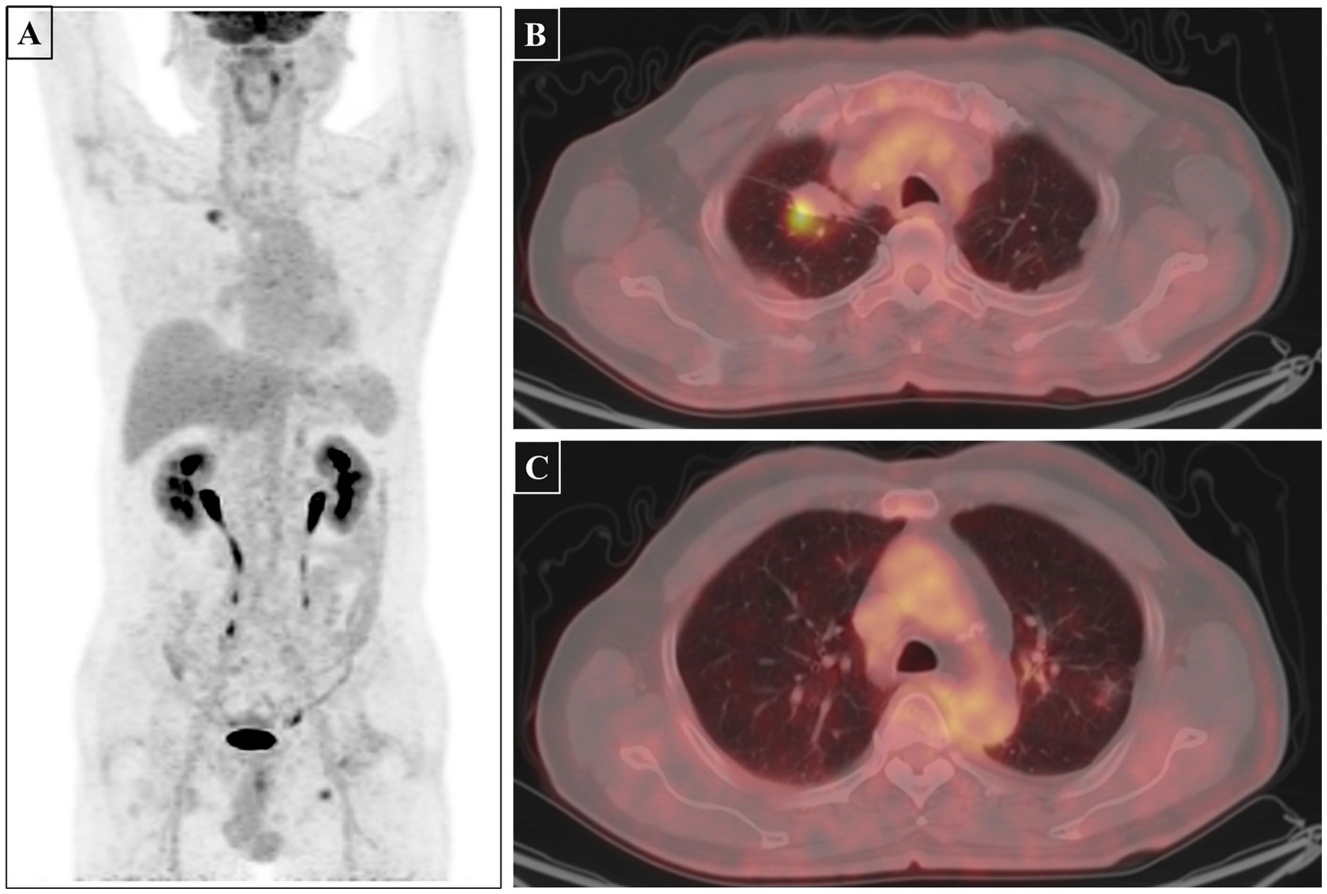
Examples of DeepSeek’s lung cancer staging errors (A:MIP), DeepSeek considered the upper lobe of the right lung (B:axial CT) to be the primary focus of lung cancer, but for the hypermetabolic nodule in the upper lobe of the left lung (C:axial CT), it gave two possibilities in the first two responses: primary lung cancer or intrapulmonary metastatic spread, and in the third time DeepSeek considered this nodule to be an intrapulmonary metastasis thus leading to a change in the M staging from M0 to M1a leading to an increase in the staging from IB to IVA.

DeepSeek-R1 and GPT-5.3 demonstrated high appropriateness (94.9%) across 39 tasks, with 37 responses rated as “quite appropriate” or “fully appropriate” (Tables 2, 3). The two “quite inappropriate” responses (5.1%, R6Q1 and R10Q1) both involved lung cancer staging (Figure 2). All responses from two models were rated as “very helpful” or “quite helpful” (100%), demonstrating that DeepSeek-R1 exhibits comparable practical utility to GPT-5.3. Furthermore, for the subset of follow-up questions related to PET/CT report interpretation, DeepSeek-R1 provided empathetic responses in 91.7% (11/12) of cases, whereas GPT-5.3 did so in 66.7% (8/12) of cases, suggesting that DeepSeek-R1 demonstrates superior empathetic communication in the context of PET/CT report interpretation (Table 5).

**Table 5:** Examples of empathetic responses from DeepSeek-R1.

Example
In the PET/CT report explaining a stage II lymphoma (R2): “I should structure the explanation in a clear, step-by-step manner, starting with the patient’s history, then moving through each section of the report, highlighting key findings, and translating medical terms into simpler language. I’ll also need to mention the recommendations for further tests and what the findings could mean in terms of possible relapse or other conditions. Making sure to reassure where results are normal and clarify areas of concern without causing unnecessary alarm.” “Let your care team know if you have questions or need help coordinating follow-up tests. Wishing you strength as you navigate this!💪”
On the patient’s life expectancy with stage IV lung cancer (R12Q1): “Okay, the user asked about their life expectancy after receiving a detailed PET/CT report indicating Stage IVB lung cancer. First, I need to acknowledge the sensitivity of this question. Life expectancy in cancer cases is highly variable and depends on many factors. I should start by reiterating that I’m not a doctor, but based on general medical knowledge, here’s what I know.” “I understand that this is an incredibly difficult question, and I want to approach it with both honesty and compassion. Life expectancy in Stage IVB lung cancer is highly variable and depends on many factors, including your overall health, treatment response, and access to advanced therapies.” “Please discuss these points thoroughly with your doctors, who can provide the most accurate and personalized guidance. You are not alone in this—support is available every step of the way. 💙”

### 3.2 Diagnosis of difficult cases

Among these 10 difficult cases, the primary and differential diagnoses of DeepSeek-R1 and GPT-5.3 were nearly identical. The accuracy of the primary diagnosis was 10% (1/10 cases) for both models, and the correct diagnosis was mentioned in 60% (6/10 cases) for differential diagnosis. In addition, 30% (3/10 cases) failed to yield an accurate clinical conclusion in either primary or differential diagnosis (Table 4).

### 3.3 Variation among trials

Across the three replicate tests of the 39 tasks, 19 (48.7%) showed “irrelevant” differences, 17 (43.6%) showed “minor” differences, and 3 tasks (7.7%) exhibited “substantial” inconsistencies, particularly regarding the tumour stage (Table 2). For GPT-5.3, “irrelevant” differences were observed in 21 (53.8%) tasks, “minor” differences in 16 (41.0%) tasks, and “substantial” inconsistencies in 2 (5.1%) tasks (Table 3). GPT-5.3 was slightly superior to DeepSeek-R1 in this regard, with no substantial difference observed between the two models.

### 3.4 Validity of references

Among the 27 tasks in which references were cited by the DeepSeek-R1 model, in 3 of the 27 tasks (11.1%), 1 reference was considered invalid because the article could not be found through a manual search (Table 6). References were fully valid in only 10 of the 27 tasks (37%). In 12 tasks (44.4%), at least one reference contained an outdated URL or was all generic (e.g., “American Joint Committee on Cancer (AJCC) Cancer Staging Manual, 8th Edition”). In responses to 2 of 27 tasks (7.4%), the referenced article could only be found through a manual search. For GPT-5.3, 2 tasks (7.4%) had invalid references, 9 (33.3%) had fully valid references, 11 (40.7%) had at least one outdated or generic reference, and 5 (18.5%) required manual searching to verify the cited article. A qualitative error analysis revealed that most inconsistencies arose from model hallucinations, including factual errors, invalid citations, and unsubstantiated clinical conclusions, with only a small minority attributable to minor wording or tone variations with no clinical impact.

**Table 6:** Example of invalid reference generated by DeepSeek.

Example
The reference that DeepSeek provided: “Article: “Delayed Imaging Improves Lesion Detection in PSMA PET/CT for Prostate Cancer” Authors: Afshar-Oromieh A, et al. Journal: European Journal of Nuclear Medicine and Molecular Imaging (2017).” The article was not found in PubMed or the European Journal of Nuclear Medicine and Molecular Imaging. The closest match is this article by the same author: Diagnostic performance of 68Ga-PSMA-11 (HBED-CC) PET/CT in patients with recurrent prostate cancer: evaluation in 1007 patientsEuropean Journal of Nuclear Medicine and Molecular Imaging(2017)10.1007/s00259-017-3711-7
The reference that DeepSeek provided: “Besson FL, et al. (2019). “FDG-PET/CT in Non-Oncologic Inflammation or Infection: A Systematic Review.” Seminars in Nuclear Medicine 49(4): 298–315.” This article was not found in PUBMED or Seminars in Nuclear Medicine, We found no similar articles.

## 4 Discussion

When patients posed practical questions related to PET/CT, the responses generated by DeepSeek were rated 100% helpful and risk-free. The model exhibited comparable performance to ChatGPT, another large language model, in terms of response appropriateness, helpfulness, and empathy (DeepSeek: 94.9, 100, 91.7%; ChatGPT: 94.9, 100, 66.7%). These findings suggest that DeepSeek has the potential to serve as a cost-effective AI assistant for clinicians and patients, delivering performance equivalent to or even superior to ChatGPT while providing more detailed reasoning processes (14–16). The identical 94.9% appropriateness rate most likely reflects a ceiling effect, as most tasks were clear, straightforward, and designed to be patient-centered. However, nearly half of the 39 tasks (48.7%) yielded discrepancies in wording, style, or formatting. To enhance its clinical utility, future development should prioritize improving the consistency and reliability of model responses.

For difficult cases, DeepSeek-R1 exhibited limited diagnostic accuracy, similar to the GPT-5.3 model. With only 10% of cases correctly diagnosed and 30% entirely incorrect results. Although these cases are inherently prone to misdiagnosis even in clinical practice, the findings reveal significant limitations in the model’s ability to handle complex medical assessments, especially those involving specialized content, highlighting the need for further optimization.

Notably, the core role of AI in medicine should be to augment clinical expertise, not to replace physicians. DeepSeek exemplifies this principle by consistently advising users to “consult your physician for next steps,” positioning itself as a decision-support tool rather than a diagnostic authority (17–19). Therefore, similar to ChatGPT, all responses generated by DeepSeek should be reviewed and verified by clinicians; this practice will greatly improve clinical workflow efficiency and optimize the overall treatment process (20).

Despite these findings, it is important to note several limitations in our research. A key limitation of this study lies in its relatively small sample size, with only 39 tasks and 10 difficult cases included in the assessment. This limited scale may restrict the generalizability of the findings, as DeepSeek’s performance might vary when applied to a broader range of clinical scenarios, patient populations, or PET/CT-related questions with greater complexity and diversity. Further, although the responses were assessed using predefined criteria by two independent nuclear medicine specialists, the evaluation of endpoints such as “appropriateness” and “helpfulness” still involved expert judgment and may therefore introduce some subjectivity. Notably, although the ninth edition of the tumour staging system was introduced in 2024, we observed that both DeepSeek and ChatGPT utilized the eighth edition of the lung cancer staging criteria. This discrepancy highlights a potential limitation of AI tools that warrants further attention. As a preliminary exploratory evaluation, this study is also restricted by a narrow disease spectrum, which constitutes another major limitation. Future investigations are required to enroll more diverse disease types and clinical cases for more comprehensive validation.

Notably, our domain-specific results differ from general public benchmarks showing that GPT-5.3 outperforms DeepSeek-R1, although the observed between-group difference in empathy was not statistically significant (*p* = 0.25). This discrepancy likely arises because general chatbot benchmarks do not fully capture performance in specialized clinical communication scenarios. Furthermore, there was no systematic difference in response length between models, suggesting that such differences are unlikely to account for the empathy ratings.

An important paradox in this study is the high response appropriateness but low reference validity in both models; both achieved 94.9% appropriateness, yet only 37.0% (DeepSeek-R1) and 33.3% (GPT-5.3) of references were fully valid. This occurs because current large language models generate clinically coherent responses mainly through pattern matching and contextual generation rather than strict evidence-based reasoning, allowing appropriate clinical advice even with incomplete, inaccurate, or fabricated references. This paradox raises critical safety concerns, as clinicians who unconditionally trust AI-generated references without independent verification may rely on unreliable evidence, leading to knowledge misinterpretation or inappropriate decision-making; thus, independent human verification remains essential. Our findings indicate that current LLMs lack rigorous evidence-based reasoning, and improvements in factual and reference reliability are urgently needed for safe clinical deployment.

AI hallucinations (DeepSeek-R1 had 11.1% invalid references and only 37% fully valid references, compared with 7.4% invalid references and 33.3% fully valid references for GPT-5.3) constitute a critical clinical safety risk. Some scholars define AI hallucination as “instances where an AI chatbot generates fictional, erroneous, or unsubstantiated information in response to queries” (20). They stem from pure prompt-response LLMs’ lack of real-time fact-checking, generating content from fragmented training data instead of verified knowledge. In clinical practice, this misleads clinicians (especially inexperienced ones) and erodes patient trust (21). Aligning with modern medical AI trends, the integration of Retrieval-Augmented Generation (RAG) and Agentic Workflows may provide a solution: RAG connects DeepSeek-R1 to verified nuclear medicine knowledge bases such as EANM guidelines and the 9th AJCC staging manual, enabling the model to retrieve accurate, traceable bibliographic information and clinical evidence, thus substantially reducing reference fabrication and the use of outdated clinical criteria. Meanwhile, the Agentic Workflows layer adds a built-in self-reflection/verification step that automatically cross-checks all generated content including DOIs, tumor staging and diagnostic inferences against the RAG-linked knowledge base before final output, flagging or revising unvalidated information in real time to resolve response inconsistencies and further enhance the model’s clinical reliability and evidence-based rigor (22, 23).

Recent public PET/CT datasets, such as PET2Rep and PETWB-REP, provide important resources for standardized evaluation of AI models in PET/CT imaging (24, 25). These datasets contain paired PET/CT imaging data and corresponding radiology reports, and are particularly valuable for developing and validating vision-language models for automated PET/CT report generation. Although the present study focused on text-based clinical interaction scenarios rather than image-to-report generation, these resources highlight an important direction for future research. Future studies should incorporate public PET/CT datasets to establish larger and more objective external benchmarks, and to evaluate multimodal models capable of integrating PET/CT images, clinical history, and textual reports.

Furthermore, although API-based evaluation enables automated, reproducible, and scalable assessment of AI performance, it remains limited by the selection of evaluation tasks, dataset representativeness, and the absence of nuanced clinical context typically provided by human experts. In addition, API-based assessments may not fully capture complex dimensions such as empathy, reasoning depth, or patient-specific considerations, and model outputs may vary according to prompt design and platform-specific constraints. Future studies should therefore incorporate more comprehensive API-based evaluation frameworks to systematically assess response consistency, reference validity, and diagnostic reliability across broader and more diverse clinical scenarios.

## 5 Conclusion

Compared with GPT-5.3, DeepSeek-R1 shows potential throughout the PET/CT workflow and boost physician efficiency and doctor-patient communication, yet critical limitations—AI citation hallucinations, inconsistent tumor staging responses, low primary diagnosis accuracy for complex cases and reliance on outdated clinical criteria—mean it lacks sufficient reliability for routine clinical use; addressing these flaws requires targeted technical optimizations (e.g., integrated RAG and agentic self-verification) and standardized clinical governance (e.g., mandatory human AI output verification). This study provides initial insights into DeepSeek’s strengths and key limitations in nuclear medicine practice, and further research with larger samples, diverse clinical indications and rigorous validation is needed to refine the model and enable its safe, effective clinical translation as a PET/CT AI assistant.

## Data Availability

The original contributions presented in the study are included in the article/Supplementary material, further inquiries can be directed to the corresponding author.

## Footnotes

1
https://chat.deepseek.com/

2
https://chatgpt.com/
