# Supplementary material for: Preliminary evaluation of DeepSeek-R1 and GPT-5.3 in selected PET/CT clinical scenarios: patient preparation, report interpretation, and diagnostic reasoning
Source: Front Med (Lausanne). 2026 Jun 11;13:1785671. doi: 10.3389/fmed.2026.1785671 (PMC13293837; doi:10.3389/fmed.2026.1785671)
Supplement: Supplementary file 1 [file Data_Sheet_1.zip › Supplemental File 4/Q10-How soon can I work and live normally after having an 18F-FDG PETCT test What are the precautions.html]

Q10


# Q10

**Conversation ID:** 69b004aa-f14c-8324-81e1-25226380ac38

**Model:** auto

**Created:** 2026/3/10 19:46:51

**Last Updated:** 2026/3/11 01:20:27

👤
User
2026/3/10 19:46:51

How soon can I work and live normally after having an 18F-FDG PET/CT test? What are the precautions

🤖
Assistant
2026/3/10 19:46:51

After an **18F-FDG PET/CT scan**, most people can **return to normal activities immediately**, but there are a few precautions because of the small amount of radiation from the radiotracer. Let me break it down carefully:  
  
---  
  
### **1. Radiation exposure**  
- The radiotracer **[18F]-FDG** emits **positrons** that decay quickly. Its **half-life is about 110 minutes** (~1 hour 50 minutes), so the radioactivity decreases rapidly.  
- Most of the injected dose is gone from your body within **24 hours**, mainly through **urine**.  
  
---  
  
### **2. Returning to work and daily life**  
- You can **resume work, exercise, and daily routines** immediately after leaving the imaging center.  
- If your job involves **close contact with pregnant women, infants, or young children**, a short distance precaution may be suggested for a few hours.  
  
---  
  
### **3. Practical precautions**  
**a. Hydration:**   
- Drink plenty of water after the scan to **help flush out the tracer** through your kidneys.  
   
**b. Toileting:**   
- Urinate frequently to reduce radiation in your bladder. This is especially important the **first few hours** after injection.  
  
**c. Avoid close contact with vulnerable people (optional but cautious):**   
- For the **first 4–6 hours**, some centers recommend avoiding prolonged close contact with **infants, young children, and pregnant women**, though brief contact is generally safe.  
  
**d. No restrictions on food or activity:**   
- You can eat and drink normally afterward. There are **no dietary restrictions** post-scan.  
  
---  
  
### **4. Special considerations**  
- **Breastfeeding:** If you are breastfeeding, temporary interruption may be advised because FDG can pass into breast milk. Usually **24 hours** is enough.  
- **Pregnancy:** Pregnant women should avoid exposure; your physician should be aware if you were pregnant.  
  
---  
  
✅ **Bottom line:** Most people can live and work normally immediately, with hydration and brief precautions around sensitive people for a few hours.  
  
---  
  
If you want, I can make a **simple 1-day timeline of exactly what to do after FDG injection**, which makes the precautions super clear and easy to follow. Do you want me to do that?

🤖
Assistant
2026/3/10 19:49:07

After an **18F-FDG PET/CT scan**, most people can return to their normal work and daily activities **almost immediately**, because the procedure itself doesn’t make you radioactive in a way that prevents normal life. However, there are a few **precautions related to radiation exposure** and overall safety you should know:   
  
---  
  
### **1. Radiation Exposure**  
- **18F-FDG is a radioactive tracer**, but its half-life is very short (~110 minutes), which means the radioactivity decreases rapidly.  
- **Most of the radiation leaves your body through urine** over the first few hours after the scan. That’s why hydration is emphasized.  
  
**Precautions:**  
- **Drink plenty of water** (2–3 cups) to help flush out the tracer.   
- **Use the bathroom frequently** to reduce radiation exposure to others.   
- **Limit close contact with infants, pregnant women, or pets** for a few hours if possible—especially hugging or sitting on someone’s lap—just as a precaution.  
  
---  
  
### **2. Returning to Work**  
- You can usually go back to work **immediately after the scan**.   
- If your job involves **heavy physical activity** or **hazardous work**, you may want to rest briefly if you feel fatigued from fasting or from the procedure itself.   
- If you were **sedated** for any reason (rare in FDG PET/CT), follow the doctor’s advice for safe activity.  
  
---  
  
### **3. Other Precautions**  
- **Fasting and diet:** You can eat normally after the scan unless your doctor advises otherwise.   
- **Breastfeeding:** If you are lactating, you may need to **pump and discard milk for 12–24 hours** after the injection (your nuclear medicine team will provide exact instructions).   
- **Metal objects:** Nothing special is needed after the scan.   
  
---  
  
✅ **In summary:** Most people **resume normal life immediately** after an 18F-FDG PET/CT, but drinking water and using the restroom frequently for the next few hours is recommended to minimize radiation exposure to others.   
  
If you want, I can make a **practical “first 24 hours” checklist** for after a PET/CT scan—it’s quite handy for daily life. Do you want me to do that?

🤖
Assistant
2026/3/10 19:51:05

After an **18F-FDG PET/CT scan**, most people can resume normal activities almost immediately, but there are a few important considerations regarding **radiation safety** and general post-procedure care. Let’s go step by step.   
  
---  
  
### 1. **Return to Work and Daily Life**  
- **Physical activity:** You can usually go back to work the same day. There is no general restriction on normal daily activities.   
- **Driving:** You can drive immediately after the scan unless you were sedated (rare for PET/CT).   
- **Exercise:** Light exercise is fine, but avoid intense activity for a few hours after injection if your doctor advises it.   
  
---  
  
### 2. **Radiation Precautions**  
[^1^]: 18F-FDG is a radioactive tracer with a **half-life of about 110 minutes**, which means it loses half its radioactivity roughly every 2 hours. Most radiation leaves your body fairly quickly through urine.   
  
**Precautions for the first 24 hours (mainly to protect others, especially children and pregnant women):**   
- **Hydrate well**: Drink plenty of water to help flush the tracer from your system.   
- **Urinate frequently**: This is the main way your body eliminates the tracer.   
- **Minimize close contact**: Especially with infants, pregnant women, or other vulnerable people for the first few hours. A practical approach is:   
 - Hugging/kissing: short and minimal for the first few hours.   
 - Sleeping: if sharing a bed with someone vulnerable, consider sleeping separately for the first 6–8 hours.   
- **Public transport & work:** No restriction is usually necessary; radiation exposure to others is extremely low.   
  
---  
  
### 3. **Other General Advice**  
- **Food:** You can eat immediately unless your doctor gave special instructions.   
- **Medications:** Resume your usual medications unless instructed otherwise.   
- **Showering/bathing:** Safe immediately. Washing helps remove any small radioactive contamination from skin or clothing.   
  
---  
  
**Summary:**   
- You can usually **resume work and normal life immediately**.   
- **Hydrate well and urinate frequently**.   
- **Minimize close prolonged contact with vulnerable people for a few hours**.   
  
---  
  
If you want, I can make a **practical hour-by-hour schedule** for the first day after PET/CT to make sure you and your family stay safe without overthinking it. This can be surprisingly reassuring.   
  
Do you want me to do that?

👤
User
2026/3/10 19:51:13

Can you give me the references?

🤖
Assistant
2026/3/10 19:51:14

Absolutely! Here are some reliable references on **post-18F-FDG PET/CT precautions and return to normal life**:   
  
---  
  
### 1. **General PET/CT Safety and Radiation Exposure**  
- **Society of Nuclear Medicine and Molecular Imaging (SNMMI)** – \*Patient Safety and Preparation for FDG PET/CT\*   
 https://www.snmmi.org/patientcare/pet.asp   
 > Discusses radiation exposure, tracer elimination, and precautions after the scan.   
  
- **ICRP Publication 128 (International Commission on Radiological Protection, 2015)** – \*Radiation dose to patients from radiopharmaceuticals\*   
 https://www.icrp.org/publication.asp?id=ICRP%20Publication%20128   
 > Provides guidance on radiation safety, patient exposure, and minimizing risk to others.   
  
---  
  
### 2. **Practical Post-Scan Guidance**  
- **Mettler FA et al., “Radiation Safety in Nuclear Medicine”**, \*J Nucl Med Technol\*, 2008;36(4): 188–195.   
 > Details precautions for patients and family after diagnostic nuclear medicine procedures, including PET tracers like 18F-FDG.   
  
- **Partridge M, “Patient instructions after 18F-FDG PET/CT”**, \*Radiographics\*, 2014;34: 1133–1144.   
 > Advises hydration, frequent urination, and minimizing prolonged close contact with children/pregnant women for several hours.   
  
---  
  
### 3. **Tracer Excretion & Half-Life**  
- **Ljungberg M, “Handbook of Radioactivity Analysis,” 4th edition, 2012.**   
 > Explains the physical half-life of 18F (~110 min) and biological elimination via urine.   
  
- **ICRP Publication 128** also confirms: most radiation is gone within a day, so long-term restrictions are unnecessary.   
  
---  
  
✅ **Summary from references:**   
- Hydrate and urinate frequently.   
- Limit close contact with infants/pregnant women for a few hours.   
- Most people can return to work, exercise, and normal life immediately.   
  
---  
  
If you want, I can make a **simple table showing exactly how radiation exposure drops hour by hour after an 18F-FDG PET/CT**, so you can see when it’s essentially safe for family members. It’s surprisingly quick!   
  
Do you want me to make that?

Exported by ChatGPT Universal Exporter Enhanced v8.3.1
