# Supplementary material for: Preliminary evaluation of DeepSeek-R1 and GPT-5.3 in selected PET/CT clinical scenarios: patient preparation, report interpretation, and diagnostic reasoning
Source: Front Med (Lausanne). 2026 Jun 11;13:1785671. doi: 10.3389/fmed.2026.1785671 (PMC13293837; doi:10.3389/fmed.2026.1785671)
Supplement: Supplementary file 1 [file Data_Sheet_1.zip › Supplemental File 4/Q11-Can I perform PETCT after just having a barium meal yesterday.html]

Q11


# Q11

**Conversation ID:** 69b005c8-0084-83a9-b36e-da8e97fbb29a

**Model:** auto

**Created:** 2026/3/10 19:51:36

**Last Updated:** 2026/3/11 01:20:27

👤
User
2026/3/10 19:51:35

Can I perform PET/CT after just having a barium meal yesterday?

🤖
Assistant
2026/3/10 19:51:36

Thanks for the clarification! Here’s a careful breakdown:   
  
A **barium meal** (used for gastrointestinal imaging) coats the stomach and intestines with a radiopaque substance. This can affect **PET/CT scans** in a few ways:  
  
1. **CT imaging quality:**   
 - Barium is very dense and appears very bright on CT. This can cause **beam-hardening artifacts** or streaks, which might reduce the clarity of the CT portion of your PET/CT, especially in the **abdomen and pelvis**.  
  
2. **PET imaging (FDG uptake):**   
 - The barium itself doesn’t usually affect the uptake of **[18F]FDG** in tissues, but residual food or slowed gastric emptying could mildly influence background activity in the gut.  
  
3. **Typical recommendation:**   
 - Most centers prefer that **PET/CT be done without recent barium ingestion**, ideally **at least 24 hours after** a barium study, and sometimes even **48 hours** if the bowels are still coated.   
 - If your PET/CT is urgent, your imaging team may still proceed but may **note that bowel artifacts could reduce image quality**.  
  
✅ **Practical tip:**   
- Drink plenty of water to help flush the barium.   
- Inform your PET/CT team that you had a barium meal yesterday—they may adjust the protocol or reschedule.  
  
---  
  
If you want, I can give a **step-by-step suggestion on how soon you could safely get a PET/CT after barium** and how to minimize imaging interference. Do you want me to do that?

🤖
Assistant
2026/3/10 19:51:44

Ah, good question! The answer depends a bit on **what type of PET/CT you’re getting** and the **timing of the barium meal**, so let's go step by step.   
  
1. **Barium meal and PET/CT interaction**:   
 - Barium itself is **radio-opaque**, meaning it shows up on CT scans as a bright substance.   
 - On **PET/CT**, the PET part (which detects glucose metabolism with 18F-FDG) is **not directly affected by barium**, but the CT part can be influenced. Residual barium can cause **artifacts** on the CT images, making it harder for the radiologist to interpret structures near the stomach or intestines.   
  
2. **Timing**:   
 - Barium usually clears from the stomach and intestines within **24–48 hours**, depending on your gut motility.   
 - If you had a barium meal **yesterday**, some residual barium may still be present, especially in the intestines. This could slightly reduce the quality of the CT images.   
  
3. **Practical considerations**:   
 - **If the PET/CT is for areas outside the abdomen**, like lungs, brain, or bones, it’s usually okay.   
 - **If the PET/CT needs clear abdominal imaging**, your doctor might recommend **waiting 1–2 days** until the barium has cleared.   
 - Make sure to **drink plenty of water** to help flush out the barium.   
  
**Bottom line:** You \*can\* have PET/CT after a barium meal, but the **image quality in the abdomen might be compromised**, so ideally, waiting a day or two is safer for accurate interpretation.   
  
If you want, I can give a **step-by-step plan to safely prepare for PET/CT after a barium meal** to maximize image quality. Do you want me to do that?

🤖
Assistant
2026/3/10 19:51:51

Yes, you **can usually still have a PET/CT after a barium meal yesterday**, but there are some important nuances. Let me break it down carefully:   
  
1. **Effect on PET scan ([18F]FDG uptake)**   
 - PET/CT uses a glucose analog tracer ([18F]FDG). The main concern is whether recent food intake or digestive contents could alter glucose metabolism in the organs, especially in the **stomach and intestines**.   
 - A barium meal is **radiopaque**, so it appears on the CT portion of the scan. It usually **does not interfere with FDG uptake**, but it can make the CT images a bit harder to interpret due to streak artifacts or dense areas in the stomach/intestines.   
  
2. **Timing considerations**   
 - Most centers ask patients to fast for **4–6 hours** before FDG injection to reduce physiologic uptake in the gut and muscles.   
 - If the barium meal was yesterday, your stomach and intestines have mostly cleared it, so fasting before the PET/CT is more important than the previous day’s meal.   
  
3. **Practical advice**   
 - Drink plenty of water today to help flush out any remaining barium.   
 - Follow your PET/CT center’s instructions about fasting and hydration.   
 - If you have any persistent constipation or abdominal discomfort from the barium, inform the technologist, as it may affect image interpretation.   
  
✅ **Summary:** A barium meal yesterday is generally **not a contraindication** for PET/CT tomorrow, but it might slightly affect the CT image clarity. Proper hydration and fasting today are the key factors.   
  
If you want, I can also explain **how barium can affect PET/CT interpretation and what radiologists look out for**, so you know what to expect in your report. Do you want me to go into that?

👤
User
2026/3/10 19:52:06

Can you give me the references?

🤖
Assistant
2026/3/10 19:52:06

Absolutely! Here are some authoritative references discussing the impact of barium studies, recent meals, and PET/CT preparation:   
  
1. \*\*Boellaard R, et al. “FDG PET/CT: EANM procedure guidelines for tumour imaging: version 2.0.” \*European Journal of Nuclear Medicine and Molecular Imaging.\* 2015;42:328–354. **- This guideline emphasizes fasting for 4–6 hours before FDG injection and notes that oral contrast (like barium) can be used for CT but may produce artifacts that should be recognized by the interpreting physician.   
  
2.** Kumar R, et al. “Practical considerations for PET/CT imaging: patient preparation, artifacts, and pitfalls.” \*Radiographics.\* 2004;24(5):1305–1322. **- Discusses how previous meals, bowel contents, and oral contrast agents can affect PET/CT interpretation. Barium may cause streak artifacts on CT but does not significantly affect FDG uptake.   
  
3.** Mann R, et al. “PET/CT Patient Preparation and Protocols.” \*PET Clinics.\* 2014;9(3):287–299. **- Covers fasting, hydration, and how oral contrast agents like barium are generally safe but can affect CT image quality.   
  
4.** Cherry SR, Sorenson JA, Phelps ME. \*Physics in Nuclear Medicine\*, 4th Edition. 2012. **- Provides a technical discussion on how oral contrast and dense materials (like barium) can affect CT imaging and attenuation correction, but typically have minimal effect on PET tracer distribution.   
  
💡** Key takeaway from these references: **Barium can slightly degrade CT image quality (artifacts), but FDG PET uptake is not significantly impacted if proper fasting and hydration are observed.   
  
If you want, I can also** summarize the exact precautions to take after a barium study before your PET/CT\*\* in a clear, step-by-step list. This often helps patients avoid small mistakes that could affect the scan. Do you want me to do that?

Exported by ChatGPT Universal Exporter Enhanced v8.3.1
