# Supplementary material for: Preliminary evaluation of DeepSeek-R1 and GPT-5.3 in selected PET/CT clinical scenarios: patient preparation, report interpretation, and diagnostic reasoning
Source: Front Med (Lausanne). 2026 Jun 11;13:1785671. doi: 10.3389/fmed.2026.1785671 (PMC13293837; doi:10.3389/fmed.2026.1785671)
Supplement: Supplementary file 1 [file Data_Sheet_1.zip › Supplemental File 4/Q3-Is minimal conversation recommended post-[18F]FDG injection.html]

Q3


# Q3

**Conversation ID:** 69b000c3-9f88-8323-9c7c-fa76a7df6a28

**Model:** auto

**Created:** 2026/3/10 19:30:12

**Last Updated:** 2026/3/11 01:20:22

👤
User
2026/3/10 19:30:11

Should I try to chat as little as possible during the waiting period after I've been injected with 18F - FDG?

🤖
Assistant
2026/3/10 19:30:12

Yes, you should minimize talking and physical activity after being injected with 18F-FDG. Here’s why:   
  
1. **FDG uptake is affected by muscle activity** – 18F-FDG is a glucose analog, and muscles actively use glucose. Talking, chewing, or moving your arms and legs can increase FDG uptake in those muscles, which may obscure or mimic disease in the PET/CT images.   
  
2. **Brain activity matters** – Any mental activity (like talking, reading, or thinking intensely) can slightly increase FDG uptake in certain brain regions, which may affect interpretation if the scan is evaluating the brain or nearby structures.   
  
3. **Resting quietly is recommended** – Patients are typically advised to sit or lie down quietly, keep calm, and avoid unnecessary movement for about 30–60 minutes after injection (the uptake phase).   
  
**Practical tips:**   
- Sit quietly, preferably in a dimly lit room.   
- Avoid talking, chewing, or using your phone if it involves a lot of mental activity.   
- Stay warm and comfortable, because shivering can also increase FDG uptake in muscles.   
  
This quiet waiting period helps the scan produce the most accurate and reliable images.   
  
If you want, I can explain **why water intake doesn’t interfere with this waiting period**, since many patients wonder about that too. Do you want me to?

🤖
Assistant
2026/3/10 19:30:18

Yes, that’s actually a **recommended practice**. After you are injected with ^18F-FDG for a PET/CT scan, the main goal during the uptake period (usually about 45–60 minutes before the scan) is to **minimize muscle activity and mental stimulation** that could increase glucose metabolism in unintended areas. Here’s why:   
  
1. **^18F-FDG is a glucose analog**: Your muscles and brain take up FDG in proportion to their metabolic activity.   
2. **Talking, chewing, or moving can increase FDG uptake in certain muscles**: For example, excessive talking could increase uptake in the neck, jaw, or facial muscles, potentially creating **false positives or artifacts** in the PET images.   
3. **Relaxation helps more accurate imaging**: Sitting quietly, resting your arms, and staying calm ensures FDG accumulates primarily in tissues relevant for detecting disease (like tumors or inflammatory sites).   
  
💡 Practical tips while waiting:   
- Sit or lie down quietly.   
- Avoid talking, reading on a tablet that requires lots of eye movement, or chewing gum.   
- Keep your arms and hands relaxed and still.   
  
So yes, chatting as little as possible is wise. Light conversation is usually okay, but anything energetic or prolonged can slightly affect the images.   
  
If you want, I can give a **short list of things to absolutely avoid vs. things that are generally safe** during the uptake period—it’s surprisingly specific. Do you want me to do that?

🤖
Assistant
2026/3/10 19:30:25

Yes, it’s generally recommended to minimize talking, moving, or any unnecessary activity during the uptake phase after injection of ¹⁸F-FDG. Here’s why:   
  
1. **Muscle uptake:** FDG is a glucose analog, and muscles consume glucose when active. Talking, moving your arms, or fidgeting can cause FDG to accumulate in those muscles, which may obscure or mimic disease on the PET images. For example, if you talk a lot, FDG may show up in your neck or jaw muscles.   
  
2. **Optimal imaging:** The goal is for FDG to be taken up primarily by tissues of interest (like tumors or areas of inflammation) rather than by normal muscles. Staying calm, relaxed, and mostly still helps improve the accuracy of the scan.   
  
3. **Comfort tips:**   
 - Sit or lie quietly in a comfortable position.   
 - Avoid chewing gum, heavy talking, or using your phone excessively (holding it is fine).   
 - Relaxing in a dimly lit, quiet room is often suggested by PET centers.   
  
In short: **yes, chat as little as possible—soft whispers or minimal talking are fine, but keep your muscles relaxed.**   
  
If you want, I can explain **why even small movements like fidgeting your hands or crossing/uncrossing legs can slightly affect the scan**—it’s more subtle than most people realize. Do you want me to go into that?

👤
User
2026/3/10 19:30:37

Can you give me the references?

🤖
Assistant
2026/3/10 19:30:38

Absolutely! Here are some reliable references and guidelines discussing activity restrictions (including talking) during the uptake phase of ¹⁸F-FDG PET/CT:   
  
---  
  
\*\*1. Boellaard R. et al., “FDG PET and PET/CT: EANM procedure guidelines for tumor PET imaging: version 2.0,” \*European Journal of Nuclear Medicine and Molecular Imaging\*, 2015;42:328–354. **- States that patients should** rest quietly **during the uptake phase to avoid physiologic uptake in muscles.   
- Mentions minimizing talking, chewing, or other muscle activity to prevent increased FDG uptake in the neck, jaw, or other muscles.**2. Kinahan PE, Fletcher JW. “Positron emission tomography–computed tomography standardized uptake values in clinical practice and assessing response to therapy.” \*Seminars in Ultrasound, CT and MRI.\* 2010;31:496–505. **- Discusses that** muscle activity before or during the uptake period can increase FDG accumulation**, potentially confounding interpretation.**3. Jadvar H., “PET in oncology: current status and future directions,” \*Journal of Nuclear Medicine Technology\*, 2009;37:115–125. **- Recommends patients** avoid talking, chewing, or physical activity **during the 45–60 minute uptake period.**4. Society of Nuclear Medicine and Molecular Imaging (SNMMI) Patient Preparation Guidelines: **- Advise that** patients remain seated and relaxed **during the uptake phase to reduce non-specific muscular uptake.   
- Source: SNMMI PET Patient Preparation Guidelines   
  
---  
  
💡** Practical takeaway: **Sit quietly, relax your muscles, and avoid talking or chewing to help your PET/CT scan reflect only the tissues of interest. Even subtle movements like fidgeting, talking, or crossing/uncrossing legs can slightly increase FDG in muscles.   
  
If you want, I can make a** concise table summarizing what activities to avoid during FDG uptake and why\*\*, with references attached—it’s handy for patient prep. Do you want me to do that?

Exported by ChatGPT Universal Exporter Enhanced v8.3.1
