# Supplementary material for: Preliminary evaluation of DeepSeek-R1 and GPT-5.3 in selected PET/CT clinical scenarios: patient preparation, report interpretation, and diagnostic reasoning
Source: Front Med (Lausanne). 2026 Jun 11;13:1785671. doi: 10.3389/fmed.2026.1785671 (PMC13293837; doi:10.3389/fmed.2026.1785671)
Supplement: Supplementary file 1 [file Data_Sheet_1.zip › Supplemental File 4/Q7-Can people with metal implants undergo PETCT.html]

Q7


# Q7

**Conversation ID:** 69b00369-f30c-8320-9205-2fa45f6ddf8e

**Model:** auto

**Created:** 2026/3/10 19:41:30

**Last Updated:** 2026/3/11 01:20:24

👤
User
2026/3/10 19:41:30

Will family members accompaning me be affected by the radiation? How to avoid?

🤖
Assistant
2026/3/10 19:41:30

Yes, there is a small potential for family members to be exposed to radiation when you undergo a PET/CT scan with **18F-FDG**, but it’s generally **very low** and temporary. Here’s a detailed explanation and practical precautions:  
  
---  
  
### **Why family members might be exposed**  
- After your PET/CT scan, your body contains a **small amount of radioactive tracer** (18F-FDG) that emits positrons and gamma rays.  
- The main source of exposure is from **close, prolonged contact**, especially within the first few hours after injection.  
- Routine day-to-day interaction (brief conversations, meals, being in the same room) usually gives negligible exposure.  
  
---  
  
### **Ways to minimize radiation exposure to others**  
  
1. **Maintain distance**  
 - Stay at least **1–2 meters (3–6 feet)** away from others, especially children and pregnant women, for the first few hours after your scan.  
  
2. **Limit close contact**  
 - Avoid hugging, kissing, or prolonged lap-sitting with family members immediately after your PET/CT.  
 - Sleep alone for the first night if possible.  
  
3. **Use personal hygiene measures**  
 - **Drink plenty of water** to help your body eliminate the tracer quickly via urine.  
 - **Urinate frequently**, especially in the first 12–24 hours, to reduce residual radioactivity.  
  
4. **Temporary precautions**  
 - Avoid sharing utensils or towels immediately after the scan if any small amount of tracer is on your hands or clothing.  
 - If breastfeeding, follow strict guidelines (your nuclear medicine team will give instructions, usually suspending breastfeeding for ~12–24 hours after FDG injection).  
  
---  
  
### **Key point**  
- PET tracers like 18F-FDG have **short half-lives** (approximately **110 minutes**), so their radioactivity decreases rapidly.  
- After 24 hours, the radiation from your body is essentially negligible for family members.  
  
---  
  
If you want, I can make a **practical “after-scan checklist”** for you and your family to minimize any exposure while keeping life normal. It’s only a few simple steps. Do you want me to do that?

🤖
Assistant
2026/3/10 19:41:37

Yes, family members or anyone accompanying you **can be exposed to some radiation** after your PET/CT scan because of the radioactive tracer (commonly ^18F-FDG) you receive. However, the exposure is usually **very low** and **short-lived**, but precautions are still recommended. Here’s a detailed explanation:   
  
---  
  
### How exposure happens  
- After injection, the radioactive tracer emits **positrons**, which quickly produce gamma rays detectable by the PET scanner.  
- The tracer gradually accumulates in tissues with high glucose metabolism (like cancer cells), and it decays over time.  
- While in your body, nearby people can receive a small radiation dose from these emissions.  
  
---  
  
### Ways to minimize exposure to family members  
1. **Keep a safe distance**   
 - Ideally, stay at least **1 meter (3 feet)** away from others for the first few hours after injection.  
   
2. **Limit close contact**   
 - Avoid prolonged hugging, kissing, or sitting close to others, especially children and pregnant women, for the first 4–6 hours.  
   
3. **Use separate bathroom if possible**   
 - Your urine contains some radioactive material. Flush the toilet twice and wash hands thoroughly.  
   
4. **Stay hydrated**   
 - Drinking water helps your body clear the tracer faster, reducing radiation exposure.  
   
5. **Follow hospital instructions**   
 - Some centers give specific instructions depending on your dose and health status.  
  
---  
  
### Key points  
- **The risk is very low** for healthy adults; children and pregnant women are more sensitive.  
- After about **24 hours**, most of the radioactivity has decayed or been excreted.  
- If you live with someone who is pregnant, your doctor may give more stringent advice.  
  
---  
  
If you want, I can give you a **practical timeline of precautions hour by hour** so family exposure is minimized without being inconvenient. It’s surprisingly doable. Do you want me to do that?

🤖
Assistant
2026/3/10 19:41:45

Yes, family members or companions can be exposed to **small amounts of radiation** when you undergo a PET/CT scan, but the risk is generally **very low** if basic precautions are followed. Here’s a clear breakdown:  
  
---  
  
### **Radiation Exposure Risk**  
1. **Source:** The radiotracer, usually **18F-FDG**, emits radiation from inside your body for a few hours after injection.   
2. **Level:** The exposure to others is typically **minimal**, often compared to natural background radiation for a short period.   
3. **Vulnerable people:** Pregnant women and small children are more sensitive to radiation, so extra caution is needed.  
  
---  
  
### **How to Minimize Radiation to Family Members**  
1. **Keep distance:** Stay at least **1–2 meters (3–6 feet)** from others, especially children and pregnant people, for the first few hours after the scan.   
2. **Avoid prolonged contact:** Try **not to hug, sit on laps, or have close face-to-face contact** immediately after injection and scan.   
3. **Use separate utensils and bathrooms if possible** for a few hours.   
4. **Hydrate well:** Drinking plenty of water helps flush the tracer out of your body faster.   
5. **Follow staff instructions:** The nuclear medicine team may provide **specific guidance** based on your dose.  
  
---  
  
**Summary:** Casual presence of family members is generally safe, but brief contact immediately after injection should be minimized. Within **24 hours**, radiation levels drop significantly, so risk becomes negligible.  
  
---  
  
If you want, I can make a **practical, step-by-step “safety plan”** for the hours after your PET/CT that makes it easy to protect your family without feeling isolated. It’s very simple. Do you want me to do that?

👤
User
2026/3/10 19:41:52

Can you give me the references?

🤖
Assistant
2026/3/10 19:41:52

Absolutely! Here are some authoritative references about radiation exposure from PET/CT and precautions for family members:   
  
---  
  
### **1. International Atomic Energy Agency (IAEA)**  
- **Title:** \*Radiation Protection in Nuclear Medicine\* (IAEA Safety Reports Series No. 63, 2011)   
- **Key point:** Recommends limiting close contact with others, especially children and pregnant women, for a few hours after PET radiotracer injection.   
- **Link:** IAEA Safety Reports   
  
---  
  
### **2. Society of Nuclear Medicine and Molecular Imaging (SNMMI)**  
- **Title:** \*Radiation Safety in Nuclear Medicine\* (Patient Education Material)   
- **Key point:** “Family members receive very small radiation doses; precautions such as short distance and avoiding prolonged close contact are sufficient.”   
- **Link:** SNMMI Radiation Safety   
  
---  
  
### **3. U.S. Nuclear Regulatory Commission (NRC)**  
- **Title:** \*Radioactive Materials in Medicine\*   
- **Key point:** Advises minimizing close contact with infants and pregnant women for several hours after diagnostic nuclear medicine scans.   
- **Link:** NRC – Patients and Visitors   
  
---  
  
### **4. European Association of Nuclear Medicine (EANM)**  
- **Title:** \*Patient Radiation Protection: Nuclear Medicine Procedures\* (EANM Guidelines, 2020)   
- **Key point:** The effective dose to family members from routine PET/CT is usually **<1 mSv**, comparable to natural background radiation over a few months.   
- **Link:** EANM Guidelines  
  
---  
  
✅ **Summary from these sources:** The radiation risk to family members is **very low**, especially if you keep a bit of distance, drink fluids, and follow basic precautions for the first few hours.  
  
---  
  
If you want, I can also **calculate roughly how much radiation a family member receives** from being near you after a standard 18F-FDG PET/CT, so you’ll have a numeric sense of the risk. It’s surprisingly small. Do you want me to do that?

Exported by ChatGPT Universal Exporter Enhanced v8.3.1
