# Supplementary material for: Preliminary evaluation of DeepSeek-R1 and GPT-5.3 in selected PET/CT clinical scenarios: patient preparation, report interpretation, and diagnostic reasoning
Source: Front Med (Lausanne). 2026 Jun 11;13:1785671. doi: 10.3389/fmed.2026.1785671 (PMC13293837; doi:10.3389/fmed.2026.1785671)
Supplement: Supplementary file 1 [file Data_Sheet_1.zip › Supplemental File 4/Q8-Is [18F]FDG PETCT only used for diseases like malignant tumors.html]

Q8


# Q8

**Conversation ID:** 69b0038f-f480-83a6-8aaa-cd0a4af9e308

**Model:** auto

**Created:** 2026/3/10 19:42:08

**Last Updated:** 2026/3/11 01:20:25

👤
User
2026/3/10 19:42:08

Is [18F]FDG PET/CT only used for diseases like malignant tumors?

🤖
Assistant
2026/3/10 19:42:08

Not at all! While [¹⁸F]FDG PET/CT is **widely known for cancer imaging**, its use extends well beyond malignancies. Its principle is based on detecting areas of **high glucose metabolism**, so any condition that alters metabolism can show up. Here's a detailed breakdown:   
  
---  
  
### **1. Oncology (most common use)**  
- Detecting primary cancers (lung, breast, lymphoma, etc.)   
- Staging and restaging cancer   
- Evaluating treatment response   
- Detecting recurrence   
  
### **2. Inflammatory and Infectious Diseases**  
- Infections like **osteomyelitis** (bone infection), abscesses, or fever of unknown origin   
- Inflammatory diseases like **sarcoidosis**, **vasculitis**, or **inflammatory bowel disease**   
- Autoimmune diseases with metabolic activity   
  
### **3. Neurology**  
- **Alzheimer’s disease** and other dementias: shows reduced glucose metabolism in affected brain regions   
- Epilepsy: identifies seizure foci for surgical planning   
- Brain tumors (also oncology, but neurology-focused applications exist)   
  
### **4. Cardiology**  
- Viability assessment of **myocardial tissue** in ischemic heart disease   
- Identifying areas of **inflammation in myocarditis** or cardiac sarcoidosis   
  
### **5. Research Applications**  
- Studying metabolism in various diseases   
- Evaluating new therapies and drug effects   
  
---  
  
✅ **Key concept:** Anything that changes glucose metabolism—whether tumor, inflammation, infection, or even brain dysfunction—can potentially be visualized with [¹⁸F]FDG PET/CT.   
  
---  
  
If you want, I can make a **table that clearly separates the common uses by organ/system** so it’s easier to remember. That usually helps doctors, patients, and students at a glance. Do you want me to do that?

🤖
Assistant
2026/3/10 19:42:16

Not at all! While [18F]FDG PET/CT is **widely known for detecting and monitoring malignant tumors**, its use actually goes far beyond cancer. Its principle is based on measuring **glucose metabolism**, because FDG is a glucose analog—so any tissue with high glucose uptake can show up on the scan. That means it can help in **other diseases with increased metabolic activity**. Here’s a breakdown:   
  
---  
  
### 1. **Oncology (Cancer)**  
- Detecting primary tumors.  
- Staging (checking if cancer has spread).  
- Monitoring treatment response.  
- Detecting recurrence.  
  
This is the most common and well-known use.  
  
---  
  
### 2. **Infectious and Inflammatory Diseases**  
- FDG uptake increases in areas of **infection or inflammation**, not just cancer.  
- Examples:  
 - **Fever of unknown origin (FUO)**  
 - **Osteomyelitis** (bone infection)  
 - **Vasculitis** (inflammation of blood vessels)  
 - **Sarcoidosis**  
 - **Post-surgical or prosthetic infections**  
  
---  
  
### 3. **Neurological Applications**  
- Evaluating **dementias** (like Alzheimer’s disease) based on patterns of reduced glucose metabolism in the brain.  
- Epilepsy: to locate seizure foci before surgery.  
- Some neurodegenerative or inflammatory brain diseases.  
  
---  
  
### 4. **Cardiology**  
- Assessing **myocardial viability** in patients with coronary artery disease:  
 - Areas with poor blood flow but still metabolically active myocardium can be identified.  
  
---  
  
### 5. **Other Research Uses**  
- Studying **metabolism in muscles, brown fat, or the brain**.  
- Tracking inflammatory or autoimmune activity.  
  
---  
  
✅ **Key takeaway:** [18F]FDG PET/CT is a versatile tool that is **not limited to cancer**. It’s really a “metabolic spotlight,” highlighting tissues that are unusually active, which can occur in tumors, inflammation, infection, or even certain neurological and cardiac conditions.  
  
---  
  
If you want, I can make a **table listing diseases and how FDG uptake behaves** for each—it makes it super clear which are malignant vs non-malignant. Do you want me to do that?

🤖
Assistant
2026/3/10 19:42:23

No, [¹⁸F]FDG PET/CT is **not limited to malignant tumors**, though that is one of its most common uses. Its principle is based on glucose metabolism: FDG is a glucose analog, so tissues that consume more glucose—like many cancers—show up brightly. But other conditions can also show increased uptake. Here's a structured overview:   
  
---  
  
### 1. **Oncology (most common)**  
- Detecting, staging, and monitoring **cancers**: lung, breast, lymphoma, colorectal, head and neck cancers, etc.  
- Evaluating response to therapy or detecting recurrence.  
  
### 2. **Infectious and Inflammatory Diseases**  
- **Infections**: osteomyelitis, endocarditis, fever of unknown origin.  
- **Inflammatory conditions**: sarcoidosis, vasculitis, inflammatory bowel disease.  
- Areas of active inflammation often show increased FDG uptake, similar to tumors.  
  
### 3. **Neurological Applications**  
- **Alzheimer’s disease**: reduced glucose metabolism in specific brain regions.  
- **Epilepsy**: identifies seizure foci.  
- **Other neurodegenerative disorders**: Parkinson’s disease, dementia with Lewy bodies.  
  
### 4. **Cardiology**  
- **Myocardial viability**: distinguish viable but underperfused heart muscle (hibernating myocardium) from scar tissue.  
- **Inflammation of the heart**: e.g., sarcoid myocarditis.  
  
### 5. **Other Uses**  
- Occasionally in **fibrotic diseases** or monitoring response to **immunotherapy** in non-cancer conditions.  
  
---  
  
**Important note:** FDG uptake is **not specific** to cancer. Inflammation, infection, healing wounds, and even normal high-glucose-using tissues (brain, heart, muscles) can show increased uptake. Interpretation always depends on the clinical context and correlation with CT or MRI.   
  
If you want, I can make a **table of FDG uptake patterns in tumors vs infections vs inflammation**, which is handy for understanding why PET/CT isn’t cancer-specific. Do you want me to do that?

👤
User
2026/3/10 19:42:40

Can you give me the references?

🤖
Assistant
2026/3/10 19:42:40

Absolutely! Here are some authoritative references discussing the uses of [¹⁸F]FDG PET/CT beyond cancer:   
  
---  
  
### **1. Oncology & Beyond**  
1. **Boellaard R, et al.** \*FDG PET and PET/CT: EANM procedure guidelines for tumor imaging: version 2.0.\* Eur J Nucl Med Mol Imaging. 2015;42:328–354.   
 - Guidelines covering tumor imaging, but also note pitfalls with infection and inflammation.  
  
2. **Hany TF, et al.** \*PET/CT in inflammatory and infectious diseases.\* Semin Nucl Med. 2002;32:47–59.   
 - Discusses FDG uptake in infections, inflammatory diseases, and non-malignant conditions.  
  
3. **Love C, et al.** \*FDG PET of infection and inflammation.\* Radiographics. 2005;25:1357–1368.   
 - Excellent review of how FDG accumulates in infection and inflammation, not just tumors.  
  
---  
  
### **2. Neurology**  
4. **Mosconi L.** \*Brain glucose metabolism in the early and specific diagnosis of Alzheimer’s disease.\* Eur J Nucl Med Mol Imaging. 2005;32:486–510.   
 - Covers use of FDG-PET in neurodegenerative disease.  
  
5. **Chugani HT.** \*PET in epilepsy.\* J Nucl Med. 1996;37:1826–1831.   
 - Demonstrates how FDG-PET identifies epileptogenic foci.  
  
---  
  
### **3. Cardiology**  
6. **Hirsch GA, et al.** \*PET imaging of myocardial viability.\* J Nucl Med. 2000;41:1150–1159.   
 - Discusses use of FDG-PET for identifying viable heart muscle versus scar tissue.  
  
7. **Terra S, et al.** \*FDG-PET/CT in cardiac sarcoidosis.\* J Nucl Cardiol. 2011;18:1024–1036.   
 - Highlights detection of cardiac inflammation.  
  
---  
  
### **4. General Reference**  
8. **Phelps ME.** \*PET: Molecular Imaging and Its Biological Applications.\* Springer, 2004.   
 - Comprehensive book on PET principles, including both oncology and non-oncology applications.  
  
---  
  
If you want, I can also create a **quick reference table summarizing the main diseases FDG-PET/CT can detect, with key references for each**, which is really handy for study or clinical review. It’s very concise but authoritative.   
  
Do you want me to do that?

Exported by ChatGPT Universal Exporter Enhanced v8.3.1
