# Supplementary material for: Preliminary evaluation of DeepSeek-R1 and GPT-5.3 in selected PET/CT clinical scenarios: patient preparation, report interpretation, and diagnostic reasoning
Source: Front Med (Lausanne). 2026 Jun 11;13:1785671. doi: 10.3389/fmed.2026.1785671 (PMC13293837; doi:10.3389/fmed.2026.1785671)
Supplement: Supplementary file 1 [file Data_Sheet_1.zip › Supplemental File 4/Q9-Does [18F]FDG PETCT showing a hypermetabolic focus mean tumor it is a tumor.html]

Q9


# Q9

**Conversation ID:** 69b00407-5e4c-8320-9f34-62aded0541b2

**Model:** auto

**Created:** 2026/3/10 19:44:07

**Last Updated:** 2026/3/11 01:20:26

👤
User
2026/3/10 19:44:07

Does [18F]FDG PET/CT showing a hypermetabolic focus mean tumor?
  
it is a tumor?

🤖
Assistant
2026/3/10 19:44:07

Not necessarily. A **hypermetabolic focus on [18F]FDG PET/CT** means that a certain area is taking up more glucose than surrounding tissue, but that **does not automatically mean it is a tumor**. FDG (fluorodeoxyglucose) is taken up by cells that are metabolically active, and this can include **both cancerous and non-cancerous processes**.   
  
Here’s a more detailed breakdown:  
  
1. **Malignant tumors** – Many cancers have high glucose metabolism, so they often appear as hypermetabolic areas. Examples include lymphoma, lung cancer, and melanoma.  
  
2. **Inflammation and infection** – Active infections, abscesses, or inflammatory processes (e.g., sarcoidosis, rheumatoid arthritis) can also show high FDG uptake.  
  
3. **Physiologic uptake** – Certain normal tissues can show high FDG uptake naturally, such as:  
 - Brain  
 - Heart (especially after recent food intake)  
 - Urinary tract (due to excretion)  
 - Muscle (after exercise)  
  
4. **Benign tumors or hyperplasia** – Some benign conditions, like adenomas or healing tissue, can also be FDG-avid.  
  
**Key point:** PET/CT shows metabolic activity, **not a definitive diagnosis**. A hypermetabolic spot needs correlation with:  
  
- **CT morphology** (size, shape, borders)  
- **Clinical context** (symptoms, history)  
- **Other tests** (biopsy, lab tests)  
  
So, a “hot spot” doesn’t automatically mean cancer, but it **needs careful evaluation**.   
  
If you want, I can explain **how doctors decide if a hypermetabolic focus is likely malignant or not** using SUV values and patterns, which often comes up in reports. Do you want me to go into that?

🤖
Assistant
2026/3/10 19:44:23

Not necessarily. A hypermetabolic focus on an [¹⁸F]FDG PET/CT means that the area is taking up more glucose than surrounding tissues. Tumors often do this because cancer cells have high glucose metabolism—but **high FDG uptake is not specific to cancer**. Other possibilities include:   
  
1. **Inflammation or infection** – Active immune cells also consume a lot of glucose. Examples: abscesses, pneumonia, autoimmune disease.   
2. **Benign tumors or hyperplasia** – Some non-cancerous growths can be metabolically active.   
3. **Physiological uptake** – Certain organs or tissues normally show high FDG uptake (brain, heart, sometimes bowel or muscles after activity).   
  
So, a hypermetabolic focus is a **clue, not a diagnosis**. The pattern, location, intensity, and correlation with CT anatomy or other imaging, plus clinical history, are all needed. Often, **biopsy or follow-up imaging** is required to confirm whether it’s cancer.   
  
If you want, I can explain **how doctors differentiate tumor vs non-tumor causes on FDG PET/CT**, step by step. Do you want me to do that?

🤖
Assistant
2026/3/10 19:44:29

Not necessarily. A hypermetabolic focus on an [18F]FDG PET/CT scan **does not automatically mean a tumor**. Let me break it down carefully:   
  
1. **What a hypermetabolic focus is:**   
 - [18F]FDG is a glucose analog, so PET/CT shows areas where cells are using a lot of glucose.   
 - Hypermetabolism means the tissue is taking up more FDG than normal.   
  
2. **Why it happens:**   
 - **Malignant tumors**: Many cancers have high glucose metabolism, so they often show as hypermetabolic.   
 - **Benign conditions**: Inflammation, infection, healing wounds, or active muscles can also take up FDG and appear hypermetabolic.   
 - **Physiological uptake**: Some organs normally show high FDG uptake, like the brain, heart, urinary tract, and sometimes the bowel.   
  
3. **What it means for diagnosis:**   
 - A hypermetabolic focus is a **red flag**, not a diagnosis.   
 - Further evaluation is usually required: correlation with CT or MRI findings, clinical history, lab tests, and sometimes biopsy.   
  
✅ **Key point:** FDG-PET/CT is highly sensitive but not specific. It can tell you “something is metabolically active here” but **cannot by itself confirm cancer**.   
  
If you want, I can explain a **step-by-step way doctors decide whether a hypermetabolic focus is likely tumor or benign**, which might make the picture much clearer. Do you want me to do that?

👤
User
2026/3/10 19:44:40

Can you give me the references?

🤖
Assistant
2026/3/10 19:44:40

Absolutely! Here are some reliable references that explain the relationship between hypermetabolic foci on [18F]FDG PET/CT and tumors, emphasizing that **not all hypermetabolic foci are malignant**:   
  
1. \*\*Huang, W., & Chen, Y. (2011). “FDG PET/CT in oncology: Current status and future directions.” \*Radiology and Oncology, 45\*(2), 87–96. **- Discusses FDG uptake in malignant vs. benign conditions and the role of PET/CT in cancer detection.   
 - Key point: Inflammation and infection can mimic tumor uptake.  
  
2.** Boellaard, R., et al. (2015). “FDG PET/CT: EANM procedure guidelines for tumour imaging.” \*European Journal of Nuclear Medicine and Molecular Imaging, 42\*, 328–354. **- Official guidelines from the European Association of Nuclear Medicine.   
 - Emphasizes that FDG is sensitive but** not specific**, and interpretation must consider clinical context.  
  
3.** Kumar, R., et al. (2009). “18F-FDG PET/CT in infection and inflammation.” \*International Journal of Infectious Diseases, 13\*(2), 136–147. **- Explains that inflammatory lesions can also appear hypermetabolic, mimicking malignancy.  
  
4.** Herholz, K. (2010). “PET and PET/CT in oncology: Indications, limitations, and pitfalls.” \*Journal of Nuclear Medicine, 51\*(1), 35S–45S. **- Discusses physiological and benign causes of FDG uptake, including brown fat, healing tissues, and inflammatory sites.  
  
5.** Schoder, H., & Noy, A. (2004). “Clinical application of PET/CT in cancer.” \*The Oncologist, 9\*(5), 412–426. **- Explains that hypermetabolic foci should be interpreted in combination with anatomical imaging and patient history.  
  
---  
  
If you want, I can also make a** short table listing common benign vs malignant causes of hypermetabolic foci\*\* with references—it’s very practical for understanding your scan results. Do you want me to do that?

Exported by ChatGPT Universal Exporter Enhanced v8.3.1
