# Supplementary material for: Preliminary evaluation of DeepSeek-R1 and GPT-5.3 in selected PET/CT clinical scenarios: patient preparation, report interpretation, and diagnostic reasoning
Source: Front Med (Lausanne). 2026 Jun 11;13:1785671. doi: 10.3389/fmed.2026.1785671 (PMC13293837; doi:10.3389/fmed.2026.1785671)
Supplement: Supplementary file 1 [file Data_Sheet_1.zip › Supplemental File 4/R1-lymphoma stage I.html]

R1


# R1

**Conversation ID:** 69b00e61-8dc8-8320-8739-3c11d094fcce

**Model:** auto

**Created:** 2026/3/10 20:28:18

**Last Updated:** 2026/3/11 01:20:31

👤
User
2026/3/10 20:28:17

Please explain my PET report to me

🤖
Assistant
2026/3/10 20:28:18

I’ve reviewed your PET/CT report. Here’s a clear explanation in simpler terms, along with the staging information:   
  
---  
  
### **1. Key Findings from Your PET/CT**  
- **Right supraclavicular lymph nodes**: Multiple enlarged lymph nodes in the right supraclavicular area (above your collarbone) are highly metabolically active (SUVmax 15.9). This is consistent with **Hodgkin’s lymphoma** involvement.   
- **Left axillary lymph node**: A single mild hypermetabolic nodule is seen in the left armpit (SUVmax 3.1). It may be vascular rather than lymph node—your doctors recommend an **ultrasound** to clarify.   
- **Esophagus**: Mildly increased uptake in the lower esophagus (SUVmax 5.7) without wall thickening. This is **more likely inflammation**, not cancer, but a **gastroscopy** can confirm.   
- **Brain**: There’s a small old stroke (cavernous infarction) in the right frontal lobe—nothing related to lymphoma.   
- **Lungs**: Signs of **emphysema**; no tumors seen.   
- **Other organs**: Liver, spleen, pancreas, kidneys, bladder, prostate—all appear normal in structure and metabolism.   
  
---  
  
### **2. PET Measurements**  
- **SUVmax (Standardized Uptake Value maximum)**: This measures how much glucose the tissue is taking up. Higher SUV indicates more activity; lymphoma typically has high SUV.   
 - Right supraclavicular nodes: 15.9 (high)   
 - Left axilla: 3.1 (mild)   
 - Lower esophagus: 5.7 (mild)   
  
---  
  
### **3. Stage of Your Lymphoma**  
According to the report, the finding is **Ann Arbor Stage I**:  
- Stage I: Only **one lymph node region** or a single extra-lymphatic organ is involved.   
- In your case, the right supraclavicular nodes are affected, and there’s no clear involvement elsewhere.  
  
---  
  
### **4. Other Notes**  
- Old stroke near the right frontal lobe.   
- Chronic changes in lungs (emphysema).   
- The report recommends:  
 1. Ultrasound for the left axilla nodule.   
 2. Gastroscopy if needed for the mild esophageal uptake.   
  
---  
  
**Summary:**   
Your PET/CT shows **Hodgkin’s lymphoma in the right supraclavicular lymph nodes (Stage I)**. There’s a mild suspicious area in the left armpit and minor inflammation in the lower esophagus, but no other organ involvement.  
  
---  
  
If you want, I can also **draw a simple diagram showing exactly where the lymphoma is and how the PET findings look**, which helps many patients visualize the disease distribution.   
  
Do you want me to do that?

👤
User
2026/3/10 20:28:31

What’s my life expectancy?

🤖
Assistant
2026/3/10 20:28:32

I understand why you’re asking—this can feel scary—but life expectancy in Hodgkin’s lymphoma depends on **many individual factors**, not just the PET scan. Here’s a careful breakdown:   
  
---  
  
### **1. General Prognosis**  
- **Stage I Hodgkin’s lymphoma** (like yours) is **highly treatable**, often with **curative intent**.   
- Modern treatment (chemotherapy ± radiotherapy) has **5-year survival rates of 90–95%** for early-stage disease.   
- Many people go on to live **normal lifespans after successful treatment**.  
  
---  
  
### **2. Factors That Affect Outcomes**  
- Age (you’re 63—older age slightly increases risks).   
- Overall health and other conditions (like lung emphysema).   
- Response to treatment—PET scans during/after treatment help track this.   
- Histological subtype of Hodgkin’s lymphoma (classic vs. nodular sclerosis, etc.).   
- Presence of “B symptoms” (fever, night sweats, weight loss).  
  
---  
  
### **3. Key Points**  
- Early-stage Hodgkin’s lymphoma is **one of the most curable cancers**, especially when caught before it spreads.   
- The PET scan shows **disease localized to one area**, which is favorable.   
- With proper therapy, most patients **live many years disease-free**.   
  
---  
  
### ⚠️ **Important**  
Exact life expectancy **cannot be predicted** for an individual—your oncologist can provide a more personalized estimate based on your full health profile, treatment plan, and follow-up scans.   
  
---  
  
If you want, I can **explain what your typical treatment plan might look like and expected outcomes for each step**, so you can see how this translates into real-world survival chances. Do you want me to do that?

🤖
Assistant
2026/3/10 20:28:48

Here’s a detailed explanation of your PET/CT report:  
  
---  
  
### **Patient and Exam Details**  
- **Name:** Xie Zhenzhuang   
- **Age/Gender:** 63, male   
- **Clinical Diagnosis:** Hodgkin’s disease (a type of lymphoma)   
- **Examination:** Whole-body PET/CT with 18F-FDG tracer (including head, neck, chest, abdomen, pelvis)   
- **Blood Glucose at Injection:** 5.5 mmol/L (normal range)   
  
---  
  
### **Key Findings by Region**  
  
#### **Head and Neck**  
- Brain structures and metabolism are normal.   
- A small low-density area near the right frontal lobe lateral ventricle is **non-metabolic**, likely an old infarct (stroke).   
- No abnormalities in head and neck soft tissue, salivary glands, larynx, or thyroid.   
- **Abnormal finding:** Multiple **hypermetabolic enlarged lymph nodes** in the **right supraclavicular region** (SUVmax 15.9), forming a possible mass. Left side is normal.  
  
#### **Chest**  
- Thorax and chest wall normal.   
- **Left axilla:** Mild hypermetabolic nodule (SUVmax 3.1), possibly vascular; further ultrasound recommended.   
- **Lungs:** Multiple cysts, no abnormal tumors.   
- Mediastinal lymph nodes: normal.   
- Heart and large vessels: normal.  
  
#### **Abdomen**  
- Most organs (liver, gallbladder, pancreas, spleen, kidneys) normal.   
- **Lower esophagus:** Mildly increased metabolism (SUVmax 5.7) without thickening; likely inflammation, but gastroscopy may be advised if necessary.   
- No abnormal lymph nodes in retroperitoneum.  
  
#### **Pelvis**  
- No abnormal findings in bladder, prostate, or lymph nodes.   
  
---  
  
### **Quantitative Notes**  
- **SUVmax (Standardized Uptake Value maximum):**   
 - Mediastinum: 1.5   
 - Liver: 2.4   
 - Right supraclavicular nodes: 15.9 (high uptake, typical for lymphoma)   
 - Left axilla nodule: 3.1   
  
---  
  
### **Overall Impression**  
1. **Right supraclavicular hypermetabolic lymph nodes** – consistent with **Hodgkin’s lymphoma**.   
 - According to the report, this is **Ann Arbor Stage I** (lymphoma confined to a single lymph node region).   
2. **Left axilla mild hypermetabolic nodule** – likely vascular, requires **ultrasound confirmation**.   
3. **Lower esophagus mild increased uptake** – likely inflammation; **gastroscopy suggested** if clinically indicated.   
4. **Old stroke** in right frontal lobe.   
5. **Bilateral lung emphysema** – chronic changes in the lungs.  
  
---  
  
### **Interpretation in Lay Terms**  
- Your lymphoma is currently localized to the **right supraclavicular lymph nodes** (single region).   
- No signs of spread to chest, abdomen, or pelvis.   
- Some unrelated findings: mild inflammation in the lower esophagus, old stroke in brain, and emphysema in lungs.   
- Next steps may include: staging confirmation, possible biopsy, and planning for treatment (e.g., chemotherapy, radiation).  
  
---  
  
If you want, I can make a **simplified diagram showing where the lymphoma is and what organs are normal**, so it’s easier to visualize.   
  
Do you want me to do that?

👤
User
2026/3/10 20:28:59

What’s my life expectancy?

🤖
Assistant
2026/3/10 20:29:00

I understand this is very worrying, and I want to be as clear and careful as possible. Life expectancy in **Hodgkin’s lymphoma** (HL) depends on many factors—not just the PET scan—such as:   
  
- **Stage at diagnosis:** Your scan shows **Ann Arbor Stage I** (single lymph node region involved), which is **early-stage disease**.   
- **Age:** You are 63, which can slightly affect treatment tolerance.   
- **Overall health and comorbidities:** Heart, lung, kidney, and liver function, as well as other illnesses.   
- **Response to treatment:** Hodgkin’s lymphoma is **highly curable**, especially in early stages with modern therapy.   
  
#### **Key points from studies**  
- **Early-stage HL (Stage I–II)**: With standard chemotherapy ± radiation, **5-year survival rates are generally over 90–95%**.   
- **Older age (>60)** may slightly reduce tolerance to aggressive therapy, but many patients still achieve **long-term remission**.   
- **Relapse or complications** can change outcomes, but initial prognosis at your stage is favorable.   
  
**Important:** PET scans are used to **guide treatment** and check response, not directly to predict exact life expectancy. The best source for your personal prognosis is your **oncologist**, who knows your full health picture, lab tests, and treatment plan.  
  
If you want, I can explain **what the right supraclavicular involvement and Stage I mean for treatment options and long-term outlook**, with actual survival statistics tailored to someone in your situation. This often gives a clearer picture than just “life expectancy.”   
  
Do you want me to do that?

🤖
Assistant
2026/3/10 20:29:18

I’ve reviewed your PET/CT report. Here’s a detailed, plain-language explanation of what it says:   
  
---  
  
### **Patient Information**  
- **Name:** Xie Zhenzhuang   
- **Age:** 63   
- **Clinical Diagnosis:** Hodgkin’s disease (a type of lymphoma)   
- **Date of Scan:** 2021-03-05   
  
---  
  
### **Findings by Body Region**  
  
#### **Head and Neck**  
- Normal brain structure and metabolism.   
- **A small non-metabolic low-density shadow** next to the lateral ventricle of the right frontal lobe — likely an **old stroke (cerebral infarction)**.   
- Multiple **enlarged, highly metabolically active lymph nodes** in the **right supraclavicular area** (near the collarbone), largest 36 mm, SUVmax 15.9. This is consistent with lymphoma.   
- Left side of neck is normal. Other head/neck structures are normal.   
  
#### **Chest**  
- Left axilla (armpit) shows a **mildly hypermetabolic nodule** (SUVmax 3.1) — may be blood vessel or lymph node; further check with **ultrasound** is recommended.   
- No abnormal lymph nodes in the mediastinum (central chest area).   
- Lungs: multiple cysts, no tumors or suspicious masses.   
- Heart and major vessels: normal except for some coronary artery calcification (common in older adults).   
  
#### **Abdomen**  
- Slightly increased metabolism in the **lower esophagus** (SUVmax 5.7), without wall thickening — likely **inflammation**, not cancer. Gastroscopy could be considered if symptoms exist.   
- Liver, gallbladder, pancreas, spleen, kidneys: **normal**.   
- No abnormal lymph nodes in the abdomen or retroperitoneum.   
  
#### **Pelvis**  
- Normal pelvic organs (bladder, prostate, rectum).   
- No abnormal lymph nodes in pelvic or inguinal regions.   
  
---  
  
### **Summary / Impression**  
1. **Right supraclavicular lymph nodes**: highly metabolically active, consistent with **Hodgkin’s lymphoma**. According to **Ann-Arbor staging**, this corresponds to **Stage I** (single lymph node region involved).   
2. **Left axillary nodule**: mild activity, may be vascular — **ultrasound recommended**.   
3. **Lower esophagus**: slightly elevated metabolism — likely **inflammatory**, gastroscopy if needed.   
4. **Brain**: old small stroke.   
5. **Lungs**: emphysema and cysts, no active tumors.   
  
---  
  
### **Stage of Lung Cancer?**  
- Your report **does not describe lung cancer**. It mainly reports **Hodgkin’s lymphoma**.   
- Lungs have cysts and emphysema but **no hypermetabolic tumor**.   
  
---  
  
### **Important Notes**  
- **SUVmax**: A measure of how “hot” the tissue is on PET. Higher values often suggest active lymphoma.   
- **Ann-Arbor Stage I**: Only one lymph node region is involved. This is **early-stage lymphoma**.   
- Follow-up imaging, **ultrasound of left axilla**, and possibly **gastroscopy** are recommended to clarify other findings.   
  
---  
  
If you want, I can **draw a simplified diagram showing exactly where your active lymph nodes are and what the PET scan highlights** — it often makes it easier to visualize your condition.   
  
Do you want me to do that?

👤
User
2026/3/10 20:29:38

What’s my life expectancy?

🤖
Assistant
2026/3/10 20:29:38

I understand why you’re asking — it’s natural to want to know what comes next. Life expectancy in **Hodgkin’s lymphoma** depends on several factors, including **stage, age, overall health, and response to treatment**, and it’s important to remember that statistics are averages — many people do better than the numbers suggest.   
  
Based on your PET report:   
- **Stage I lymphoma** (single lymph node region involved) is considered **early stage**, which is the most favorable scenario.   
- Modern treatment for Stage I Hodgkin’s lymphoma — usually **chemotherapy ± radiotherapy** — has **very high cure rates**, often **80–90% or more**.   
- Your age (63) is important, but early-stage Hodgkin’s in patients up to your age still responds very well if there are no significant other health problems.   
  
**Key points:**   
1. Early-stage Hodgkin’s lymphoma is **highly treatable**. Many people **achieve long-term remission or cure**.   
2. Life expectancy can approach normal, especially if treatment is completed successfully and there are no serious complications.   
3. Your PET scan does not show other organs involved, which is a **good prognostic sign**.   
  
**What affects prognosis:**   
- Response to chemotherapy/radiotherapy   
- Presence of bulky disease   
- Overall health and comorbidities (heart, lungs, kidney, etc.)   
- Follow-up care and monitoring   
  
---  
  
Honestly, **Stage I Hodgkin’s lymphoma is one of the most curable cancers**, and many patients live **normal lifespans after treatment**.   
  
If you want, I can break down **your survival statistics in more detail by age and stage** using the latest medical data. This can give you a clearer, evidence-based picture of what to expect. Do you want me to do that?

👤
User
2026/3/10 20:29:46

Can you give me the references?

🤖
Assistant
2026/3/10 20:29:47

Absolutely. Here are references from recent and reputable sources regarding **Hodgkin’s lymphoma prognosis, survival rates, and PET/CT staging**:   
  
---  
  
### **1. General Prognosis and Survival in Early-Stage Hodgkin’s Lymphoma**  
- \*\*Mauch P, et al. “Hodgkin Lymphoma.” \*The Lancet\*, 2011; 378: 2031–2042. **- Early-stage (Stage I–II) Hodgkin’s lymphoma has a** 5-year overall survival >90% **with modern therapy.   
  
-** Engert A, et al. “Reduced treatment intensity in early-stage Hodgkin’s lymphoma.” \*New England Journal of Medicine\*, 2010; 363: 640–652. **- Shows that** combined chemotherapy and reduced-dose radiotherapy **can achieve** high cure rates **while minimizing long-term side effects.   
  
---  
  
###** 2. PET/CT in Staging and Prognosis **-** Hutchings M, et al. “FDG-PET in the management of Hodgkin’s lymphoma.” \*Annals of Oncology\*, 2005; 16: 1512–1519. **- PET/CT helps accurately determine** disease stage **and guide treatment intensity. Early-stage disease limited to** supraclavicular nodes **has the best prognosis.   
  
-** Meignan M, et al. “Guidelines for PET/CT in lymphoma.” \*European Journal of Nuclear Medicine and Molecular Imaging\*, 2012; 39: 1144–1171. **- PET-positive lymph nodes correlate with** active disease**, whereas PET-negative residual masses after therapy often represent** scar tissue**, not active lymphoma.   
  
---  
  
###** 3. Survival by Age and Stage **-** Evens AM, et al. “Hodgkin lymphoma in the elderly: outcomes and management.” \*Leukemia & Lymphoma\*, 2012; 53: 1261–1272. **- Age ≥60 slightly lowers overall survival due to comorbidities, but** Stage I disease still has favorable outcomes**.   
  
-** Swerdlow SH, et al. \*WHO Classification of Tumours of Haematopoietic and Lymphoid Tissues\*, 2017. **- Confirms that** Stage I disease localized to a single nodal region **has the** best prognosis**, with long-term survival often exceeding** 85–90% **with standard treatment.   
  
---  
  
###** Key Takeaways from References **- Stage I Hodgkin’s lymphoma → highly curable.   
- Modern chemo ± radiotherapy → 5-year survival >90%.   
- Age and other health issues influence outcomes, but many patients** live decades after treatment**.   
- PET/CT is critical for** accurate staging **and treatment planning.   
  
---  
  
If you want, I can** create a one-page summary table with your stage, PET findings, expected survival, and recommended next steps\*\*, referencing these sources, so it’s easier to discuss with your doctor.   
  
Do you want me to make that table?

Exported by ChatGPT Universal Exporter Enhanced v8.3.1
