# Supplementary material for: Preliminary evaluation of DeepSeek-R1 and GPT-5.3 in selected PET/CT clinical scenarios: patient preparation, report interpretation, and diagnostic reasoning
Source: Front Med (Lausanne). 2026 Jun 11;13:1785671. doi: 10.3389/fmed.2026.1785671 (PMC13293837; doi:10.3389/fmed.2026.1785671)
Supplement: Supplementary file 1 [file Data_Sheet_1.zip › Supplemental File 4/R11-stage IVA lung cancer.html]

R11


# R11

**Conversation ID:** 69b013bc-2dc0-8320-aab3-0204e34256d9

**Model:** auto

**Created:** 2026/3/10 20:51:09

**Last Updated:** 2026/3/11 01:20:39

👤
User
2026/3/10 20:51:08

Please explain my PET report to me

🤖
Assistant
2026/3/10 20:51:09

I’ve carefully reviewed your PET/CT report. Here’s a detailed explanation in plain language:   
  
---  
  
### **1. Main Findings in the Lungs**  
- **Right upper lobe:** There is a hypermetabolic nodule (about 27×26×21 mm) with features suggestive of cancer, such as irregular borders and pleural pulling. This is consistent with **peripheral lung cancer** in the right lung.  
- **Right middle lobe:** A very small “ground-glass” nodule (4 mm) with clear borders, likely early or low-activity lesion. **Follow-up observation** is recommended.  
- **Right lower lobe:** Two tiny nodules (<3 mm) that do not show increased metabolism, likely **benign**.  
  
### **2. Lymph Nodes**  
- Multiple **mediastinal lymph nodes** (regions 2R, 4R, 7) show increased metabolism (SUVmax 5.1). This indicates **lymph node metastases** from the lung cancer.  
  
### **3. Bone**  
- Left transverse process of the 8th thoracic vertebra shows a **metabolic increase (SUVmax 8.4) with structural destruction**, indicating **bone metastasis**.  
  
### **4. Other Findings**  
- Mild inflammation around the right shoulder.  
- Hypermetabolic area in the lower esophagus, likely **reflux esophagitis**.  
- Prostate slightly enlarged with calcification (common in older men).  
- No abnormal findings in liver, spleen, kidneys, adrenal glands, or pelvis.  
  
---  
  
### **5. Summary**  
- **Primary cancer:** Right lung, upper lobe.  
- **Metastases:** Mediastinal lymph nodes and bone (thoracic vertebra).  
- **Other lesions:** Small nodules in right middle/lower lobes (mostly benign/early).  
- **Incidental findings:** Reflux esophagitis, prostatic hyperplasia.  
  
---  
  
### **6. Likely Stage**  
Based on **lung cancer with mediastinal lymph node metastasis and bone metastasis**, the disease would likely be considered **Stage IV (advanced metastatic disease)** according to standard lung cancer staging (TNM classification)【6†source】.   
  
- **T:** Tumor size in right upper lobe (T1-T2 based on 27 mm)  
- **N:** Mediastinal lymph nodes involved (N2)  
- **M:** Distant metastasis to bone (M1b)  
  
---  
  
### **7. Next Steps**  
- Oncologist consultation for **treatment planning** is crucial. Options often include:  
 - **Systemic therapy:** Chemotherapy, targeted therapy, immunotherapy  
 - **Bone-directed therapy** if symptomatic  
 - **Local therapy** (surgery/radiotherapy) is usually limited in Stage IV  
  
---  
  
If you want, I can make a **visual diagram of your PET findings** showing the tumor, lymph nodes, and bone metastasis—this can help you understand exactly where the cancer has spread.   
  
Do you want me to make that diagram?

👤
User
2026/3/10 20:51:18

What is the stage of my lung cancer?

🤖
Assistant
2026/3/10 20:51:19

Based on your PET/CT report, your lung cancer involves:   
  
1. **Primary tumor** – a nodule in the **right upper lobe** (~27 mm).   
2. **Lymph nodes** – **mediastinal nodes** (2R, 4R, 7) are involved.   
3. **Distant metastasis** – **bone metastasis** in the left transverse process of the 8th thoracic vertebra.   
  
---  
  
### **TNM Staging Interpretation**  
- **T (Tumor size/extent):** T1b–T2a (because the nodule is 2.7 cm, under 3 cm, but has pleural involvement; usually T1c if >2–3 cm)   
- **N (Lymph nodes):** N2 (mediastinal nodes involved)   
- **M (Metastasis):** M1b (single extrathoracic metastasis, bone)   
  
---  
  
### **Overall Stage**  
According to the **AJCC 8th edition for lung cancer**, **T1–2, N2, M1b corresponds to Stage IV** (specifically **Stage IVb** for a single distant metastasis).   
  
✅ This is **advanced/metastatic lung cancer**.  
  
---  
  
If you want, I can also explain **what this stage means for treatment options and prognosis**, in simple terms. Do you want me to do that?

🤖
Assistant
2026/3/10 20:51:30

I’ve reviewed your PET/CT report. Here’s a clear breakdown in understandable terms:  
  
---  
  
### **1. Primary Lung Cancer**  
- **Location:** Posterior segment of the upper lobe of the right lung.  
- **Size:** About 27 × 26 × 21 mm (roughly 2.7 cm × 2.6 cm × 2.1 cm).  
- **Features on PET/CT:** High uptake of the tracer (SUVmax 12.3) — this is consistent with active cancer. Other features like spiculated edges (“burr”), lobulation, and pleural pulling also suggest malignancy.  
- **Interpretation:** Confirms peripheral lung cancer in the right upper lobe.  
  
---  
  
### **2. Lymph Node Involvement (Mediastinum)**  
- **Locations:** 2R, 4R, and 7 mediastinal regions.  
- **PET Uptake:** SUVmax 5.1 in the largest nodes.  
- **Size:** Largest short diameter ~7 mm.  
- **Interpretation:** These are considered metastatic (cancer spread) to mediastinal lymph nodes.  
  
---  
  
### **3. Bone Metastasis**  
- **Location:** Left transverse process of the 8th thoracic vertebra.  
- **PET Uptake:** SUVmax 8.4 (high metabolic activity).  
- **Interpretation:** Cancer has spread to this vertebra.  
  
---  
  
### **4. Other Lung Findings**  
- **Middle lobe right lung:** A small 4 mm ground-glass nodule. **Recommendation:** Follow-up observation, likely not immediately concerning.  
- **Lower lobe right lung:** Two tiny non-metabolic nodules (<3 mm), likely benign.  
  
---  
  
### **5. Other Observations**  
- **Right shoulder soft tissue:** Slightly increased metabolism, probably inflammation.  
- **Lower esophagus:** Mildly increased metabolism, likely reflux esophagitis (inflammation, not cancer).  
- **Prostate:** Slight enlargement with calcifications; common in older men.  
- **Abdomen, liver, spleen, kidneys, pelvis:** No abnormal findings or cancer spread seen.  
  
---  
  
### **6. Summary**  
- **Confirmed cancer:** Right upper lobe of the lung.  
- **Metastases:** Mediastinal lymph nodes and bone (T8 vertebra).  
- **Other findings:** Mostly benign or inflammatory changes.  
  
**Likely cancer stage:** Based on the PET/CT:  
- Tumor (T): Primary tumor ~2.7 cm → T1c-T2a depending on exact invasion.  
- Node (N): Multiple mediastinal nodes → N2.  
- Metastasis (M): Bone metastasis → M1b.  
  
This corresponds to **Stage IV lung cancer** (advanced), because bone metastasis indicates distant spread.  
  
---  
  
If you want, I can make a **diagram showing your tumor, lymph nodes, and bone metastasis** so it’s visually easier to understand.   
  
Do you want me to do that?

👤
User
2026/3/10 20:51:59

What is the stage of my lung cancer?

🤖
Assistant
2026/3/10 20:52:00

Based on your PET/CT report, here’s the staging analysis:  
  
---  
  
### **1. Tumor (T)**  
- **Primary tumor:** Posterior segment of the right upper lobe, ~2.7 cm in size.   
- **Features:** Peripheral, spiculated, invading no nearby structures mentioned.   
- **T stage:** Likely **T1c–T2a** (T1c = 2–3 cm, T2a = 3–4 cm) depending on precise tumor measurements and local invasion.  
  
---  
  
### **2. Lymph nodes (N)**  
- **Involved nodes:** Mediastinal regions 2R, 4R, and 7.   
- **PET uptake:** SUVmax 5.1, largest node 7 mm.   
- **N stage:** **N2** (ipsilateral mediastinal nodes involved).  
  
---  
  
### **3. Distant metastasis (M)**  
- **Bone metastasis:** Left transverse process of T8 vertebra (SUVmax 8.4).   
- **M stage:** **M1b** (single extrathoracic metastasis; if multiple distant organs, M1c).  
  
---  
  
### **4. Overall Stage**  
- **TNM:** T1c–T2a, N2, M1b   
- **Clinical stage:** **Stage IV** lung cancer (advanced stage), because any distant metastasis—including bone—defines Stage IV.  
  
---  
  
**Important:** Stage IV means the cancer has spread beyond the lung and nearby lymph nodes, which affects treatment choices. It doesn’t mean treatment isn’t possible—systemic therapies (like targeted therapy, immunotherapy, chemotherapy) are typically considered. Surgery is rarely used at this stage unless for symptom relief or specific circumstances.  
  
---  
  
If you want, I can also **explain what this stage means for prognosis and treatment options** in clear, practical terms. Do you want me to do that?

🤖
Assistant
2026/3/10 20:52:07

Here’s a detailed explanation of your PET/CT report from Hebei Provincial People's Hospital:  
  
---  
  
### **Patient and Examination Overview**  
- **Name:** Xie Xiaomin   
- **Age:** 64   
- **Clinical Diagnosis:** Lung cancer after chemotherapy   
- **Examination:** Whole-body PET/CT (base of skull to mid-thigh) with 18F-FDG tracer   
- **Purpose:** To evaluate the presence, size, metabolic activity, and spread of cancer after treatment.  
  
---  
  
### **Key Findings**  
  
#### **Neck & Head**  
- Normal metabolism and structure in sinuses, pharynx, larynx, salivary glands, thyroid, and neck lymph nodes.   
- **Interpretation:** No evidence of tumor or metastasis in this region.  
  
#### **Thorax (Chest and Lungs)**  
- **Primary Tumor:**   
 - **Location:** Posterior segment, upper lobe of the right lung   
 - **Size:** 27 × 26 × 21 mm   
 - **Metabolic activity:** SUVmax 12.3 (high uptake, typical of active cancer)   
 - **Characteristics:** Spiculated edges, lobulation, pleural traction, adjacent bronchus truncated → consistent with peripheral lung cancer.   
  
- **Other Lung Nodules:**   
 - Middle lobe, right lung: 4 mm ground-glass nodule, clear border, non-metabolic → monitor.   
 - Lower lobe, right lung: two <3 mm non-metabolic nodules → likely benign.   
  
- **Lymph Nodes:**   
 - Mediastinal regions 2R, 4R, 7 show hypermetabolic lymph nodes (SUVmax 5.1, largest 7 mm) → indicates **lymph node metastases**.   
  
- **Bone Metastasis:**   
 - Left transverse process of the 8th thoracic vertebra shows metabolic activity (SUVmax 8.4) with localized bony destruction → **bone metastasis confirmed**.   
  
- **Other Chest Findings:**   
 - Soft tissue around the right shoulder joint is mildly hypermetabolic (SUVmax 3.7) → likely inflammatory.   
 - Pleura, heart, great vessels, bronchi normal.   
 - Lower esophagus shows slight uptake (SUVmax 4.0) → possibly reflux esophagitis.  
  
#### **Abdomen**  
- Liver, spleen, pancreas, adrenal glands, kidneys: normal structure and metabolism.   
- No enlarged lymph nodes or abnormal metabolic activity.   
  
#### **Pelvis**  
- Prostate slightly enlarged with calcifications, but no abnormal metabolic activity.   
- No abnormal lymph nodes or metabolic activity in pelvic soft tissues.  
  
---  
  
### **Summary and Interpretation**  
1. **Confirmed Findings:**  
 - Active **primary lung cancer** in the right upper lobe.  
 - **Mediastinal lymph node metastases**.  
 - **Bone metastasis** in the thoracic spine (T8).   
  
2. **Other findings:**   
 - Small ground-glass lung nodule (monitor).   
 - Non-metabolic nodules (likely benign).   
 - Mild inflammation around right shoulder.   
 - Mild esophageal inflammation (likely reflux).   
 - Benign prostatic enlargement.  
  
---  
  
### **Staging Implication (Based on PET/CT)**  
- **Primary tumor (T):** T2 (size >2 cm but ≤5 cm, peripheral)   
- **Lymph nodes (N):** N2 (mediastinal lymph node involvement)   
- **Metastasis (M):** M1b (single extrathoracic metastasis – bone in T8)   
  
**Likely stage:** **IVa** lung cancer (according to the AJCC 8th edition) due to bone metastasis.  
  
---  
  
### **Clinical Notes**  
- This report confirms **advanced lung cancer with spread to mediastinal nodes and one bone site**.   
- **Recommended:** Discussion with an oncology team about systemic therapy (chemotherapy, immunotherapy, targeted therapy) and possible local treatments for bone metastasis (radiotherapy or bone-protective therapy).   
- **Follow-up:** Small lung nodules and other inflammatory findings can be monitored.  
  
---  
  
If you want, I can also **create a visual diagram showing your tumor, lymph nodes, and bone metastasis locations**, which can make this report easier to understand.   
  
Do you want me to do that?

👤
User
2026/3/10 20:52:19

What is the stage of my lung cancer?

🤖
Assistant
2026/3/10 20:52:20

Based on your PET/CT report, here’s a careful breakdown of your lung cancer stage:  
  
---  
  
### **Key Findings Relevant to Staging**  
1. **Primary tumor (T):**   
 - Right upper lobe, 27 mm (2.7 cm), spiculated → **T2** (tumor >2 cm but ≤5 cm).  
  
2. **Lymph node involvement (N):**   
 - Hypermetabolic mediastinal lymph nodes in **regions 2R, 4R, and 7** → **N2** (mediastinal node metastasis).  
  
3. **Distant metastasis (M):**   
 - **Bone metastasis** at the left transverse process of T8 vertebra → **M1b** (single extrathoracic metastasis).  
  
---  
  
### **Overall Stage**  
According to the **AJCC 8th edition TNM staging for non-small cell lung cancer**:  
  
- **T2** (primary tumor size)   
- **N2** (mediastinal lymph nodes)   
- **M1b** (single distant metastasis, bone)   
  
✅ **Stage IVa lung cancer**   
  
This is **advanced stage disease**, which generally requires systemic therapy rather than surgery alone.  
  
---  
  
If you want, I can also **explain what this stage means for treatment options and prognosis** in clear, practical terms. Do you want me to do that?

👤
User
2026/3/10 20:52:25

Can you give me the references?

🤖
Assistant
2026/3/10 20:52:26

Yes! Here are authoritative references for the staging and PET/CT findings of lung cancer, including your situation:  
  
---  
  
### **Lung Cancer Staging References**  
1. **AJCC Cancer Staging Manual, 8th Edition (2017)**   
 - The standard reference for TNM staging of non-small cell lung cancer (NSCLC).   
 - Describes T (tumor size), N (nodal involvement), and M (distant metastasis) categories.   
 - Source: entity["book","AJCC Cancer Staging Manual, 8th Edition","lung cancer section, 2017"]   
  
2. **Detterbeck FC, et al. “The Eighth Edition Lung Cancer Stage Classification.” Chest. 2017;151(1):193-203**   
 - Peer-reviewed paper detailing how the 8th edition TNM system applies to clinical practice.   
 - DOI: 10.1016/j.chest.2016.10.015   
  
---  
  
### **PET/CT in Lung Cancer**  
3. **Lardinois D, et al. “Staging of non-small-cell lung cancer with integrated PET/CT.” N Engl J Med. 2003;348:2500-7**   
 - Demonstrates PET/CT accuracy for detecting primary tumor, nodal, and distant metastases.   
  
4. **Boellaard R, et al. “FDG PET/CT: EANM procedure guidelines for tumor imaging.” Eur J Nucl Med Mol Imaging. 2015;42:328–354**   
 - Guidelines for interpreting FDG uptake (SUVmax), including differentiation of tumor vs inflammation.   
  
5. **National Comprehensive Cancer Network (NCCN) Guidelines: Non-Small Cell Lung Cancer, Version 3.2025**   
 - Provides recommended imaging, staging, and treatment based on TNM stage.   
 - URL: https://www.nccn.org/professionals/physician\_gls/pdf/nscl.pdf   
  
---  
  
These references are the **standard sources used by clinicians worldwide** to stage lung cancer and interpret PET/CT findings.   
  
If you want, I can **also make a simple table linking your PET findings directly to the TNM stage**, so you can see exactly why your cancer is Stage IVa. It makes it much easier to understand.   
  
Do you want me to do that?

Exported by ChatGPT Universal Exporter Enhanced v8.3.1
