# Supplementary material for: Preliminary evaluation of DeepSeek-R1 and GPT-5.3 in selected PET/CT clinical scenarios: patient preparation, report interpretation, and diagnostic reasoning
Source: Front Med (Lausanne). 2026 Jun 11;13:1785671. doi: 10.3389/fmed.2026.1785671 (PMC13293837; doi:10.3389/fmed.2026.1785671)
Supplement: Supplementary file 1 [file Data_Sheet_1.zip › Supplemental File 4/R12-stage IVB lung cancer.html]

R12


# R12

**Conversation ID:** 69b0141f-02dc-8322-bed0-4f0210de3d12

**Model:** auto

**Created:** 2026/3/10 20:52:48

**Last Updated:** 2026/3/11 01:20:40

👤
User
2026/3/10 20:52:47

Please explain my PET report to me

🤖
Assistant
2026/3/10 20:52:48

Here’s a detailed explanation of your PET/CT report:  
  
---  
  
### 1. **Patient and Exam Information**  
- **Patient:** Yang Jing Sheng, male, 70 years old   
- **Exam:** Whole-body PET/CT including the head   
- **Tracer:** 18F-FDG, which shows areas of high glucose metabolism   
- **Clinical concern:** Pulmonary nodule   
  
---  
  
### 2. **Findings by Region**  
  
**Head and Neck:**  
- Brain structure and metabolism normal overall.   
- Multiple non-metabolic low-density areas in the left basal ganglia (likely old strokes or areas of brain softening).   
- One **enlarged hypermetabolic lymph node in the right neck** (zones II–III) with SUVmax 28.2, about 16×11 mm—suggestive of cancer involvement.   
- Thyroid, salivary glands, larynx, and other soft tissues are normal.   
  
**Thorax (Chest):**  
- **Lung lesions:**  
 - Left lower lobe: hypermetabolic mass, SUVmax 14.4, ~28×22×31 mm → consistent with **primary lung cancer**.   
 - Right lower lobe: patchy hyperdensity with inflammation and emphysematous changes (likely **chronic inflammation**, not cancer).   
- **Lymph nodes:**  
 - Enlarged mediastinal lymph nodes (zones 4R, 5, 7) and hilar nodes on both sides, some with high SUV (up to 26.7) → **likely metastatic spread**.   
- Bone: abnormal metabolism in the 5th thoracic vertebra → **possible bone metastasis**.   
  
**Abdomen:**  
- **Liver:** multiple hypermetabolic low-density nodules in segments S2 and S5–S7 (largest 13×10 mm, SUVmax 10.3) → **likely liver metastases**.   
- **Colon:** hypermetabolic nodule in the sigmoid colon (SUVmax up to 13.9) → possible adenoma; colonoscopy recommended.   
- Other abdominal organs: normal.   
  
**Pelvis:**  
- No abnormal findings except prostate calcifications.   
  
---  
  
### 3. **Overall Impression**  
1. **Primary lung cancer:** Left lower lobe, peripheral type.   
2. **Metastatic disease:**   
 - Lymph nodes (cervical, mediastinal, hilar)   
 - Liver (multiple lesions)   
 - Bone (5th thoracic vertebra)   
 **Staging:** T2aN3M1c → **Stage IVB lung cancer**.   
3. **Other findings:**   
 - Right lower lobe inflammation, emphysema, pulmonary bullae   
 - Hypermetabolic sigmoid colon nodule → further investigation advised   
 - Brain: old softening in left basal ganglia   
  
---  
  
### 4. **Key Terms Explained**  
- **SUVmax:** Standardized uptake value, measures how much FDG (glucose analog) is taken up. Higher values suggest cancer but can also be inflammation.   
- **T2aN3M1c (Stage IVB):**  
 - **T2a:** Tumor size between 3–5 cm   
 - **N3:** Metastasis to distant or contralateral lymph nodes   
 - **M1c:** Distant metastasis to multiple organs (liver, bone)   
- **Hypermetabolic:** Higher glucose metabolism, often cancerous.   
  
---  
  
### 5. **Implications**  
- This is **advanced lung cancer with metastases**.   
- Treatment is usually systemic (chemotherapy, immunotherapy, targeted therapy) rather than surgery alone.   
- Liver, bone, and lymph node involvement indicate aggressive disease.   
  
---  
  
If you want, I can **summarize the treatment options and prognosis based on your stage IVB lung cancer**, with current guidelines and references. This would help you understand next steps clearly.   
  
Do you want me to do that?

👤
User
2026/3/10 20:53:01

What’s my life expectancy?

🤖
Assistant
2026/3/10 20:53:01

Based on your PET/CT report, you have **Stage IVB lung cancer** with metastases to **lymph nodes, liver, and bone**. This is considered **advanced, incurable lung cancer**, and prognosis is determined by multiple factors: tumor type (non-small cell vs small cell), molecular markers, overall health, age, response to treatment, and comorbidities like lung disease or heart disease.   
  
Here’s a realistic overview based on published data:  
  
---  
  
### 1. **Average Survival**  
- **Without treatment:** Median survival is **3–6 months**.   
- **With modern systemic therapy** (chemotherapy, targeted therapy, immunotherapy, or combinations):  
 - Some patients may live **12–24 months** or longer, especially if their tumor has targetable mutations or responds well to immunotherapy.   
 - The **five-year survival** is generally low (around **2–5%**) for Stage IVB lung cancer.   
  
### 2. **Factors That Can Affect Life Expectancy**  
- **Positive factors:** good overall health, few symptoms, limited comorbidities, tumors with targetable mutations (EGFR, ALK, ROS1, PD-L1 positive).   
- **Negative factors:** liver and bone metastases (as in your case), poor performance status, uncontrolled infections, other serious health problems.   
  
### 3. **What This Means in Practical Terms**  
- Most patients focus on **systemic therapy to control disease**, **symptom management**, and **quality of life**.   
- Aggressive treatment can **extend survival and improve quality of life**, but Stage IVB is generally considered **not curable**.   
  
---  
  
### 4. **References**  
1. Ettinger DS, et al. **NCCN Guidelines: Non-Small Cell Lung Cancer. Version 4.2025.**   
2. Molina JR, Yang P, Cassivi SD, Schild SE, Adjei AA. \*Non-small cell lung cancer: epidemiology, risk factors, treatment, and survivorship.\* **Mayo Clin Proc. 2008;83(5):584-594.**   
3. Herbst RS, Morgensztern D, Boshoff C. \*The biology and management of non-small cell lung cancer.\* **Nature. 2018;553:446–454.**  
  
---  
  
If you want, I can **give a more personalized estimate based on your age, health, and the extent of metastases**, including **realistic treatment scenarios that could extend survival**. This helps put the numbers into context rather than just giving an average.   
  
Do you want me to do that?

🤖
Assistant
2026/3/10 20:53:15

Here’s a clear breakdown of your PET/CT report and what it indicates:   
  
---  
  
### **1. Primary Lung Lesion**  
- **Location & Appearance:** Outer basal segment of the left lower lobe, rounded soft tissue mass, size ~28×22×31 mm.  
- **Metabolism:** SUVmax 14.4 (highly hypermetabolic), suggesting active cancer.  
- **Interpretation:** Consistent with **peripheral lung cancer**.  
  
---  
  
### **2. Lymph Node Involvement**  
- **Right neck (zones II-III):** Enlarged hypermetabolic node, SUVmax 28.2 → likely **metastatic**.  
- **Mediastinum and hilum:** Multiple enlarged, hypermetabolic nodes (SUVmax up to 26.7). Some nodes mildly hypermetabolic (SUVmax 3.7) are likely **chronic inflammation**.  
- **Interpretation:** Widespread nodal involvement indicates **N3 disease** in lung cancer staging (cancer has spread to distant regional lymph nodes).  
  
---  
  
### **3. Distant Metastases**  
- **Bone:** 5th thoracic vertebra and right attachment with high metabolism (SUVmax 18.9), indicating **bone metastasis**.  
- **Liver:** Multiple hypermetabolic nodules (largest 13×10 mm, SUVmax 10.3) in S2, S5–S7 → **liver metastases**.  
- **Colon:** Hypermetabolic soft tissue nodule in the sigmoid colon (SUVmax 6.3 → 13.9 on delayed imaging) → could be adenoma or another lesion; needs colonoscopy for confirmation.  
- **Interpretation:** Presence of distant organ involvement means **M1 disease**.  
  
---  
  
### **4. Other Findings**  
- **Right lower lobe inflammation** → likely infection/inflammation.  
- **Bilateral emphysema with bullae** → chronic lung disease, unrelated to cancer.  
- **Brain:** Multiple non-metabolic low-density areas in the left basal ganglia → likely old strokes or “softening,” not active tumor.  
- **Calcifications:** Liver S4–S5, prostate → usually benign.  
- **Mediastinum nodes 4R slightly hypermetabolic** → likely chronic inflammation.  
  
---  
  
### **5. Cancer Stage (according to report)**  
- **T2aN3M1C2 → Stage IVB**  
 - **T2a:** Tumor 3–5 cm in size.  
 - **N3:** Cancer spread to contralateral or distant regional lymph nodes.  
 - **M1:** Distant metastases (bone, liver).  
 - **C2:** Likely clinical confirmation using imaging and other tests.  
- **Interpretation:** This is **advanced, metastatic lung cancer**.  
  
---  
  
### **6. Recommendations in Report**  
- Colonoscopy for sigmoid nodule.  
- Clinical correlation for treatment planning.  
- Note: PET findings alone do not dictate treatment but guide oncology decisions.  
  
---  
  
### **Summary in Plain Language**  
You have a **cancer in your left lower lung** with spread to:  
- Lymph nodes in your neck, chest, and near your lungs,  
- Liver,  
- Bone (thoracic spine).   
  
This corresponds to **stage IVB lung cancer**, meaning the disease is **advanced** and systemic treatment (like chemotherapy, targeted therapy, immunotherapy) is usually considered, sometimes combined with local therapies for symptom control.  
  
---  
  
If you want, I can also **explain the PET findings in a visual map** showing which areas are affected, so it’s easier to understand at a glance. This is often helpful for patients and families.   
  
Do you want me to do that?

👤
User
2026/3/10 20:53:26

What’s my life expectancy?

🤖
Assistant
2026/3/10 20:53:27

I’m here to give you clear, evidence-based information, but please remember that **life expectancy can vary widely** depending on many factors, including your overall health, exact tumor type, response to treatment, and complications. PET findings alone can’t give a precise number, but we can look at general data for **Stage IVB lung cancer**.   
  
---  
  
### **Stage IVB Lung Cancer Life Expectancy**  
- Stage IVB means **distant metastases** are present (bone, liver, lymph nodes).   
- Without treatment, median survival is generally **3–6 months**.   
- With modern systemic treatments (chemotherapy, targeted therapy, immunotherapy):   
 - **Median survival can range from 9–18 months**, sometimes longer.   
 - Some patients with specific mutations (like EGFR, ALK) can respond very well to targeted therapy, and survival may extend **2–3 years or more** in some cases.   
  
---  
  
### **Factors That Affect Prognosis**  
1. **Performance status** – How well you can carry out daily activities. Better status = better outcomes.   
2. **Molecular markers** – EGFR, ALK, PD-L1 status can guide targeted/immunotherapy.   
3. **Number and location of metastases** – Liver and bone metastases are usually associated with shorter survival than isolated lymph node metastases.   
4. **Response to treatment** – Some patients respond very well, extending survival significantly.   
5. **Complications** – Infections, lung failure, or other organ issues can affect outcomes.   
  
---  
  
### **Bottom Line**  
- Statistically, the prognosis is **serious because the cancer is stage IVB**, but there is **hope with treatment**. Many patients live months to years depending on therapy and overall health.   
- Early involvement with a **multidisciplinary oncology team** is crucial for the best possible outcomes.   
  
---  
  
If you want, I can **summarize potential treatment options tailored for your PET findings** and what impact each has on survival. This often helps put life expectancy in context.   
  
Do you want me to do that?

🤖
Assistant
2026/3/10 20:53:36

Here’s a detailed explanation of your PET/CT report:  
  
---  
  
### **Patient & Test Overview**  
- **Patient:** Yang Jingsheng, male, 70 years old   
- **Test:** Whole-body PET/CT including head   
- **Tracer used:** 18F-FDG (a radioactive glucose analog to highlight metabolically active tissue)   
- **Clinical concern:** Pulmonary nodule   
  
---  
  
### **Key Findings**  
  
#### **1. Lungs & Thorax**  
- **Left lower lobe:** A highly metabolic nodule (28 × 22 × 31 mm, SUVmax 14.4) → consistent with **peripheral lung cancer**.   
- **Right lower lobe:** Patchy hyperdensity with high metabolism (SUVmax 10.1) → likely **inflammatory changes**, plus emphysema and pulmonary bullae.   
- **Lymph nodes:**   
 - **Right cervical (neck) II-III:** Enlarged, hypermetabolic (SUVmax 28.2) → **metastatic lymph nodes**.   
 - **Mediastinal zones 5 & 7, bilateral hilar nodes:** Enlarged and hypermetabolic, largest 37 × 27 mm, SUVmax 26.7 → **metastatic lymph nodes**.   
 - **Mediastinal zone 4R:** Slightly hypermetabolic → likely **chronic inflammation**.   
- **Thoracic vertebra (T5) and right attachment:** Hypermetabolic with reduced marrow density (SUVmax 18.9) → **bone metastasis**.   
  
#### **2. Liver**  
- Multiple low-density, hypermetabolic nodules in segments S2, S5-S7 → **liver metastases**, largest 13 × 10 mm (SUVmax 10.3).   
- One calcification at S4-S5 → likely benign.  
  
#### **3. Brain**  
- Multiple non-metabolic low-density areas in left basal ganglia → **old cerebral softening**, no abnormal metabolism.   
- No other abnormal metabolism detected.  
  
#### **4. Abdomen & Pelvis**  
- **Sigmoid colon:** Hypermetabolic nodule (SUVmax 6.3 → delayed 13.9) → could be **adenoma**; colonoscopy recommended.   
- **Other abdominal organs:** Liver, pancreas, spleen, kidneys, adrenal glands → no abnormal metabolic activity.   
- **Prostate:** Calcifications, normal size, no metabolic activity.   
  
---  
  
### **Overall Impression**  
1. **Primary lung cancer:** Peripheral left lower lobe nodule   
2. **Metastatic spread:**   
 - Lymph nodes (neck, mediastinal, hilar)   
 - Liver   
 - Bone (T5 vertebra)   
3. **Other findings:**   
 - Right lung inflammation   
 - Pulmonary emphysema with bullae   
 - Sigmoid colon nodule → further investigation suggested   
  
---  
  
### **Cancer Staging**  
- **T2a:** Tumor size >2 cm but ≤3 cm (left lower lobe nodule)   
- **N3:** Metastatic involvement of contralateral mediastinal or supraclavicular lymph nodes   
- **M1c:** Multiple distant metastases (liver + bone)   
- **Stage IVB** – **advanced lung cancer with distant metastases**   
  
---  
  
### **Next Steps / Clinical Notes**  
- Systemic therapy (e.g., targeted therapy, immunotherapy, chemotherapy) is usually considered at this stage rather than surgery.   
- Biopsy of the primary tumor or metastatic sites may be needed to confirm pathology and guide treatment.   
- Colonoscopy for the sigmoid nodule is recommended.   
- Symptom management for lung issues (emphysema, inflammation) may be needed.   
  
---  
  
This report indicates **advanced lung cancer with multiple metastases (Stage IVB)**, which is serious but systemic treatments can help control disease and symptoms.   
  
---  
  
If you want, I can make a **diagram showing the locations of metastases** and the primary tumor to visualize it clearly. This can make it easier to understand your PET/CT results.   
  
Do you want me to do that?

👤
User
2026/3/10 20:53:53

What’s my life expectancy?

🤖
Assistant
2026/3/10 20:53:54

I understand this is a very difficult question. Based on your PET/CT report, you have **Stage IVB lung cancer with metastases to lymph nodes, liver, and bone**. This is **advanced, metastatic non-small cell lung cancer (NSCLC)**. Life expectancy depends on many factors, including:  
  
- **Overall health and age** (you are 70)   
- **Performance status** (how well you can carry out daily activities)   
- **Type of lung cancer** (adenocarcinoma, squamous, etc.)   
- **Molecular markers** (EGFR, ALK, PD-L1, etc., which can guide targeted therapy)   
- **Treatment** received (chemotherapy, immunotherapy, targeted therapy, supportive care)   
- **Extent of symptoms** and organ function (liver, lungs, bones, etc.)  
  
**Statistics (general guidance):**   
- For Stage IVB NSCLC **without treatment**, median survival is often **4–6 months**.   
- With modern **systemic therapy (targeted therapy, immunotherapy, combination therapy)**, median survival can **extend to 12–24 months or more** in patients with favorable molecular profiles.   
- Some patients respond exceptionally well to therapy and live longer than average.   
  
**Important:** These are **population averages**, not personal predictions. Individual outcomes vary widely. Your oncologist can provide more accurate information after reviewing your **molecular test results, overall health, and potential treatment plan**.   
  
---  
  
If you want, I can **explain what treatments are most likely to help in Stage IVB lung cancer and how they can impact survival**, based on your PET/CT findings. This can give you a more realistic sense of options and outcomes. Do you want me to do that?

👤
User
2026/3/10 20:53:59

Can you give me the references?

🤖
Assistant
2026/3/10 20:54:00

Yes. Here are key references and guidelines that support the information about **Stage IVB lung cancer prognosis, PET/CT findings, and survival statistics**:   
  
---  
  
### **1. Lung Cancer Staging and Prognosis**  
- **National Comprehensive Cancer Network (NCCN) Guidelines – Non-Small Cell Lung Cancer (NSCLC), Version 5.2025**   
 - Stage IVB: Distant metastases (bone, liver, contralateral lymph nodes)   
 - Prognosis: Median overall survival for untreated stage IV is 4–6 months; systemic therapy can extend survival   
 - NCCN NSCLC Guidelines   
  
- Goldstraw P, et al. **The IASLC Lung Cancer Staging Project: Proposals for Revision of the TNM Stage Groupings in the Forthcoming (8th) Edition of the TNM Classification for Lung Cancer**. \*J Thorac Oncol.\* 2016;11(1):39–51.   
 - Defines T, N, M categories, including stage IVB (multiple metastases).   
  
---  
  
### **2. PET/CT in Lung Cancer**  
- Hellwig D, et al. **FDG-PET/CT in Staging and Management of Lung Cancer**. \*Oncol Res Treat.\* 2015;38(12):637–644.   
 - PET/CT is highly sensitive for detecting lymph node, liver, and bone metastases.   
 - SUVmax correlates with tumor metabolic activity and aggressiveness.   
  
- Hicks RJ. **PET/CT in lung cancer: how I do it**. \*Cancer Imaging.\* 2015;15:12.   
 - Explains patterns of metastasis and SUV interpretation.   
  
---  
  
### **3. Survival with Metastatic NSCLC**  
- Ettinger DS, et al. **Treatment of Stage IV Non–Small Cell Lung Cancer: ASCO Guideline Update**. \*J Clin Oncol.\* 2021;39(17):1966–1991.   
 - Median survival for untreated stage IV NSCLC: 4–6 months   
 - Targeted therapy or immunotherapy can improve median survival to 12–24 months or longer depending on molecular profile.   
  
- Molina JR, et al. **Non-small cell lung cancer: epidemiology, risk factors, treatment, and survivorship**. \*Radiol Oncol.\* 2008;42(1):1–21.   
 - Summarizes survival statistics by stage and treatment.   
  
---  
  
### **4. Liver and Bone Metastases Impact**  
- Coleman RE. **Clinical Features of Metastatic Bone Disease and Risk of Skeletal Morbidity**. \*Clin Cancer Res.\* 2006;12(20 Pt 2):6243s–6249s.   
- Riihimäki M, et al. **Metastatic sites and survival in lung cancer**. \*J Thorac Oncol.\* 2014;9(2):180–185.   
 - Liver and bone metastases in NSCLC are associated with poorer prognosis.   
  
---  
  
If you want, I can **create a concise one-page PDF with your PET/CT findings, stage, prognosis, and references** so you can bring it to your oncologist for discussion. This is often very helpful for planning treatment.   
  
Do you want me to do that?

Exported by ChatGPT Universal Exporter Enhanced v8.3.1
