# Supplementary material for: Preliminary evaluation of DeepSeek-R1 and GPT-5.3 in selected PET/CT clinical scenarios: patient preparation, report interpretation, and diagnostic reasoning
Source: Front Med (Lausanne). 2026 Jun 11;13:1785671. doi: 10.3389/fmed.2026.1785671 (PMC13293837; doi:10.3389/fmed.2026.1785671)
Supplement: Supplementary file 1 [file Data_Sheet_1.zip › Supplemental File 4/R2-lymphoma stage II.html]

R2


# R2

**Conversation ID:** 69b00f43-9d8c-83a2-94bf-141680036b89

**Model:** auto

**Created:** 2026/3/10 20:32:04

**Last Updated:** 2026/3/11 01:20:32

👤
User
2026/3/10 20:32:02

Please explain my PET report to me

🤖
Assistant
2026/3/10 20:32:04

Here’s a clear explanation of your PET/CT report. I’ll go section by section and summarize the main points.   
  
---  
  
### **Patient and Exam Context**  
- **Name/Age/Gender:** Wang Xianrui, 38-year-old female   
- **History:** You were previously treated for **Hodgkin’s lymphoma (nodular sclerosis type)**, had one relapse, and now have new findings in your right breast and right axilla.   
- **Purpose of Exam:** Evaluate for **possible lymphoma recurrence** and assess overall post-treatment status.   
- **Tracer Used:** **18F-FDG**, which highlights areas of increased glucose metabolism, often seen in cancer.   
  
---  
  
### **Key Findings**  
  
#### 1. **Lymph Nodes / Right Upper Body**  
- **Multiple hypermetabolic lymph nodes** were seen:  
 - Medial part of the right upper arm  
 - Right subclavian region  
 - Right axilla (largest: 65 × 55 mm, SUVmax 12.6)  
 - Outer edge of right scapula  
- Some nodes **fused into a mass**, suggesting significant involvement.  
- The **pattern and history** strongly suggest **active lymphoma relapse**.  
  
#### 2. **Right Breast / Chest Wall**  
- **Enlarged right breast** and **thickened skin** of right upper arm, chest wall, and breast.  
- Mildly hypermetabolic nodule in **deep right breast** (10 mm, SUVmax 2.1) — may represent **lymphoma involvement** or a separate breast nodule.  
- Suggests **lymphatic obstruction** due to lymphoma.  
  
#### 3. **Head, Neck, Thyroid**  
- **No abnormal metabolism** in most of the head and neck structures.  
- A **small nodule in the left thyroid lobe** (18 × 13 mm, SUVmax 2.3) — mild activity; **ultrasound recommended** to clarify.  
  
#### 4. **Chest**  
- Left chest and lung areas are **normal**.  
- **No mediastinal or left axillary lymph node involvement**.  
  
#### 5. **Abdomen and Pelvis**  
- Most organs normal.  
- **Mild thickening and mild metabolic activity in the beginning of the ascending colon** (SUVmax 5.6); colonoscopy **may be recommended**.  
  
---  
  
### **Impression / Interpretation**  
1. **Relapsed Hodgkin’s lymphoma** affecting right axilla, subclavian, upper arm lymph nodes.   
2. **Right breast enlargement and skin changes** likely due to lymphoma-related lymphatic obstruction.   
3. **Small deep breast nodule** — may be lymphoma or separate lesion; further evaluation needed.   
4. **Mildly hypermetabolic thyroid nodule** — recommend **ultrasound**.   
5. **Mild ascending colon wall thickening** — consider colonoscopy if clinically indicated.   
  
**SUVmax Explanation:**   
- SUVmax (Standardized Uptake Value maximum) measures glucose uptake.   
- Values >10 are generally highly suspicious for active lymphoma (your largest node: 12.6).   
- Mild values (2–3) may be benign or low-grade activity (thyroid nodule, breast nodule).   
  
---  
  
### **Summary in Plain Terms**  
- Your PET/CT shows **active lymphoma recurrence** mainly in the **right axilla and adjacent lymph nodes**.   
- Right breast and chest wall changes are likely secondary to **lymphatic blockage**.   
- Some **other mild findings** (thyroid nodule, colon wall thickening) are noted but may be unrelated; further tests are recommended.   
- No lymphoma detected in other regions (chest, abdomen, pelvis, head/neck).  
  
---  
  
If you want, I can also **explain what this means for your prognosis and next steps**, including treatment options and follow-up testing.   
  
Do you want me to go over that?

👤
User
2026/3/10 20:32:17

What is the stage of my lymphoma?

🤖
Assistant
2026/3/10 20:32:17

Based on your PET/CT report, here’s how the **stage of your Hodgkin’s lymphoma** can be interpreted using the **Ann Arbor staging system**, which is the standard for Hodgkin’s lymphoma:  
  
---  
  
### **Key Findings Relevant to Staging**  
1. **Involvement of lymph nodes:**  
 - Multiple nodes in **right axilla, right subclavian region, medial right upper arm, outer edge of right scapula**.  
 - Some nodes **fused into a mass**.  
 - No lymph nodes involved on **left side**, mediastinum, or abdomen/pelvis (except mild ascending colon wall thickening — unclear if lymphoma).   
  
2. **Extranodal involvement:**  
 - Right breast and chest wall **skin changes** likely due to **lymphatic obstruction**, possibly **direct extension** of lymphoma.  
 - No other organ involvement detected.   
  
---  
  
### **Ann Arbor Staging**  
- **Stage I:** Single lymph node region or single extranodal site.   
- **Stage II:** ≥2 lymph node regions on **same side of the diaphragm**.   
- **Stage III:** Lymph nodes on **both sides of the diaphragm**.   
- **Stage IV:** **Diffuse or disseminated involvement** of one or more extranodal organs (bone marrow, liver, lungs, etc.).   
  
- You have **multiple lymph nodes on the right side (same side of diaphragm)** → fits **Stage II**.   
- **Extranodal extension** to right breast/chest wall can be noted as **“Stage IIE”**, where “E” stands for **extranodal involvement contiguous with lymph node disease**.   
  
---  
  
### ✅ **Conclusion**  
- Most likely, your current lymphoma is **Stage IIE Hodgkin’s lymphoma (right side, contiguous extranodal involvement)**.   
- No evidence of **Stage III or IV** since **nodes on the left side or distant organs are not involved**.   
  
---  
  
If you want, I can **explain what Stage IIE means for treatment options and prognosis** in your situation. This can help you understand next steps clearly.   
  
Do you want me to do that?

🤖
Assistant
2026/3/10 20:32:37

Here’s a clear breakdown of your PET/CT report from 2020-07-30:  
  
---  
  
### **Patient Information**  
- **Name:** Wang Xianrui   
- **Age:** 38   
- **Gender:** Female   
- **Clinical History:** Hodgkin's lymphoma diagnosed 13 years prior; treated with radiotherapy; one relapse. Recently, enlargement of the right breast and right axillary lymph nodes noted.  
  
---  
  
### **Purpose of PET/CT**  
- Evaluate post-treatment status and check for possible recurrence of Hodgkin’s lymphoma.  
  
---  
  
### **Key Findings**  
  
1. **Lymph Nodes (Right Upper Body)**  
 - Multiple hypermetabolic lymph nodes in:  
 - Medial root of the right upper arm   
 - Right subclavian region   
 - Right axilla   
 - Outer edge of right scapula   
 - Some lymph nodes fused into a mass.   
 - **Highest SUVmax:** 12.6 (SUVmax = measure of metabolic activity)   
 - **Largest node:** ~65mm × 55mm   
 - Interpretation: Suggests active lymphoma involvement.  
  
2. **Right Breast & Skin**  
 - Enlargement and skin thickening in:  
 - Right breast   
 - Right upper arm   
 - Right chest wall   
 - A small hypermetabolic nodule in deep right breast (SUVmax 2.1, 10 mm)   
 - Interpretation: Could represent lymphoma involvement; obstruction of lymphatic drainage is likely causing swelling.  
  
3. **Thyroid**  
 - Mildly hypermetabolic nodule in the left lobe of thyroid (18 × 13 mm, SUVmax 2.3)   
 - Recommendation: Thyroid ultrasound for further evaluation.  
  
4. **Colon**  
 - Mild thickening at beginning of ascending colon, mildly increased metabolism (SUVmax 5.6)   
 - Recommendation: Colonoscopy if clinically indicated.  
  
5. **Other Areas**  
 - **Head & Neck:** Normal   
 - **Chest (lungs, mediastinum, heart):** Normal   
 - **Abdomen (liver, pancreas, spleen, kidneys):** Normal   
 - **Pelvis:** Normal   
  
---  
  
### **Summary / Impression**  
- **Recurrence of Hodgkin's lymphoma** in right axillary and supraclavicular lymph nodes is highly likely.   
- Right breast enlargement and skin thickening are likely due to lymphatic obstruction from lymphoma.   
- Small breast nodule and thyroid nodule need follow-up investigations.   
- Mild ascending colon changes may require colonoscopy.  
  
---  
  
### **What This Means**  
- The PET/CT shows **active lymphoma in right lymph nodes and possibly the right breast**.   
- Other organs appear normal, so no widespread disease is currently evident.   
- Follow-up with your oncologist is crucial to decide next steps, which could include biopsy of suspicious nodes, systemic therapy, or targeted radiation.  
  
---  
  
If you want, I can also **explain what SUVmax values mean for prognosis and what this could imply about disease activity**. This helps put the numbers in context for you.   
  
Do you want me to do that?

👤
User
2026/3/10 20:32:48

What is the stage of my lymphoma?

🤖
Assistant
2026/3/10 20:32:48

Based on your PET/CT report and clinical history, we can make an **approximate staging** using the **Ann Arbor system**, which is standard for Hodgkin’s lymphoma.   
  
Here’s how it applies to your case:  
  
---  
  
### **Key points from your report**  
1. **Lymph node involvement**:  
 - Multiple lymph nodes on the **right side**: axilla, supraclavicular, subclavian, upper arm.   
 - Some nodes **fused into a mass**.  
  
2. **Right breast and skin**:  
 - Enlarged breast and thickened skin due to **lymphatic obstruction** (likely secondary to lymphoma, not a separate site).   
 - Small hypermetabolic nodule in the deep right breast (possibly involved).  
  
3. **Other organs**:  
 - No evidence of lymphoma in lungs, liver, spleen, bones, abdomen, or other lymph node regions on the left side or below the diaphragm.  
  
---  
  
### **Ann Arbor Staging Approximation**  
  
| Stage | Criteria | How it fits your case |  
|-------|---------|----------------------|  
| I | Involvement of **one lymph node region** or **single extralymphatic organ** | Not your case – multiple nodes involved |  
| II | Involvement of **two or more lymph node regions on the same side of the diaphragm** | ✅ Likely – right axilla, right supraclavicular, subclavian, upper arm (all on same side of diaphragm) |  
| III | Lymph nodes **on both sides of the diaphragm** | ❌ Not your case – left side not involved |  
| IV | **Disseminated involvement** of one or more extralymphatic organs | ❌ No widespread organ involvement |  
  
**Extra info:**   
- “B” symptoms (fever, night sweats, weight loss) aren’t mentioned in the report. If absent → Stage II **A**; if present → Stage II **B**.   
- The breast nodule could be considered extranodal involvement, but since it’s contiguous/secondary to lymph node mass, it likely **does not automatically upstage** to Stage IV.  
  
---  
  
### **Likely Stage:**   
**Stage II (probably II-A)** – multiple lymph nodes on the **right side of the diaphragm**, with local extranodal extension (breast/skin).   
  
---  
  
**Important:** Staging is **ultimately confirmed by your hematologist/oncologist**, because biopsy results, clinical exam, and symptoms are also factored in. PET/CT alone gives strong clues but isn’t the final word.   
  
If you want, I can also explain **what Stage II Hodgkin’s lymphoma generally means for treatment options and prognosis**. This can help you understand your next steps.   
  
Do you want me to go over that?

🤖
Assistant
2026/3/10 20:33:01

Here’s a clear explanation of your PET/CT report from 2020-07-30. I’ll break it down section by section in plain language:  
  
---  
  
### 1. **Background**  
- You have a history of **Hodgkin’s lymphoma** (nodular sclerosis type) diagnosed over 13 years ago, treated with **radiotherapy**, with **one relapse** in the past.  
- Two months before this PET/CT, you noticed **right breast enlargement** and **enlarged lymph nodes in the right axilla and supraclavicular region**. This scan was done to **evaluate possible lymphoma recurrence**.  
  
---  
  
### 2. **PET/CT Findings**  
  
#### **Lymph Nodes**  
- **Multiple hypermetabolic (active) lymph nodes** were seen on the **right side**: upper arm, axilla, subclavian region, and near the right scapula.  
- Some of these nodes **fused into a mass**, largest ~65×55 mm, **SUVmax 12.6**.  
 - SUVmax measures metabolic activity; higher numbers can indicate **active lymphoma**.  
- **Right breast changes**: thickened skin and a small nodule (10 mm) with **mild uptake, SUVmax 2.1**.  
  
**Interpretation:**   
These findings are consistent with **relapsed lymphoma** in the right axilla and surrounding areas. The breast nodule may also be involved but is less metabolically active.  
  
---  
  
#### **Head & Neck**  
- Normal **brain, skull, sinuses, pharynx, thyroid (except left lobe nodule), salivary glands, and vocal cords**.  
- A **small nodule in the left thyroid lobe** (18×13 mm, SUVmax 2.3) was seen.   
 **Recommendation:** thyroid ultrasound to check further.  
  
---  
  
#### **Chest**  
- No abnormal findings in the **lungs, mediastinum, left axilla, heart, or pleura**.  
  
---  
  
#### **Abdomen**  
- **Mostly normal**, except:  
 - Mild thickening and slightly increased metabolism at the **start of the ascending colon (SUVmax 5.6)**.  
 - **Recommendation:** colonoscopy if needed.  
  
---  
  
#### **Pelvis**  
- **Normal**: bladder, uterus, adnexa, pelvic fat, no enlarged lymph nodes.  
  
---  
  
### 3. **Overall Impression**  
1. **Relapsed lymphoma**: Right axilla, right upper arm, right subclavian, and scapular region. Likely causing **lymphatic obstruction**, leading to **swelling of the right breast and skin**.  
2. **Right breast nodule**: possibly related to lymphoma. Follow-up or biopsy may be needed.  
3. **Left thyroid nodule**: mild uptake; recommend **ultrasound**.  
4. **Ascending colon**: mild uptake; **colonoscopy** if clinically indicated.  
  
---  
  
### 4. **Key Points**  
- The scan shows **active lymphoma relapse** on the right side.  
- **No distant spread** to lungs, mediastinum, abdomen, or pelvis was seen.  
- Some **incidental findings** (thyroid nodule, mild colon thickening) require **further evaluation** but are not immediately concerning.  
  
---  
  
If you want, I can also **explain what this means in terms of treatment options and prognosis** based on current guidelines for relapsed Hodgkin’s lymphoma.   
  
Do you want me to do that next?

👤
User
2026/3/10 20:33:15

What is the stage of my lymphoma?

🤖
Assistant
2026/3/10 20:33:15

Based on your PET/CT report and your history, we can estimate the **stage of your lymphoma using the Ann Arbor staging system**, which is standard for **Hodgkin’s lymphoma**. Let’s go step by step carefully.   
  
---  
  
### 1. **Sites of involvement**  
From your report:   
- **Right axilla, right subclavian region, medial right upper arm, outer edge of right scapula** → **lymph node regions on the same side of the diaphragm**   
- **Right breast and overlying skin** → extranodal involvement (breast tissue, skin)   
- **No involvement of lymph nodes on the left side, no mediastinal, abdominal, or pelvic nodes**   
- **No distant organ involvement** (lungs, liver, spleen, kidneys, brain normal)   
  
---  
  
### 2. **Ann Arbor staging**  
The system considers:   
- **Stage I:** Single lymph node region or single extranodal site   
- **Stage II:** Two or more lymph node regions **on the same side of the diaphragm**   
- **Stage III:** Lymph node regions **on both sides of the diaphragm**   
- **Stage IV:** Disseminated involvement of **one or more extranodal organs** (liver, bone marrow, lungs, etc.)   
  
**Additional letters:**   
- **E** = involvement of a single **extranodal site adjacent to known nodes**   
- **A/B** = absence (A) or presence (B) of systemic symptoms (fever, night sweats, weight loss)  
  
---  
  
### 3. **Applying to your case**  
- Multiple lymph nodes on the **right side** only → still **same side of diaphragm** → **Stage II**   
- Right breast and skin involvement → extranodal extension → **Stage IIE or IIE**   
- No systemic B symptoms mentioned → **“A”**   
  
✅ **Most likely stage: Stage IIEA Hodgkin’s lymphoma (relapsed)**   
  
---  
  
### 4. **Summary**  
- **Stage II:** Involvement of 2 or more lymph node regions **on the same side of the diaphragm**   
- **E:** Extranodal involvement of breast and skin   
- **A:** No systemic “B symptoms” reported   
  
> This is a **relapsed lymphoma**, so it’s important to discuss **treatment options**, which often include **chemotherapy, immunotherapy, or possibly radiotherapy**, depending on your prior treatments and overall health.  
  
---  
  
If you want, I can make a **diagram showing the affected lymph nodes and staging**, which makes it easier to visualize. It can help you understand the **extent of disease and why it’s Stage IIE**.   
  
Do you want me to do that?

👤
User
2026/3/10 20:33:27

Can you give me the references?

🤖
Assistant
2026/3/10 20:33:27

Absolutely! Here are authoritative references for **Hodgkin’s lymphoma staging and PET/CT interpretation** relevant to your report:   
  
---  
  
### **1. Ann Arbor Staging System**  
- **Connors JM. Clinical manifestations and diagnosis of Hodgkin lymphoma in adults.** \*UpToDate.\* 2024.   
 - Explains staging: I–IV, with “E” (extranodal) and A/B symptoms.   
 - UpToDate link   
  
- **Carbone PP, Kaplan HS, Musshoff K, et al.** \*Report of the Committee on Hodgkin’s Disease Staging Classification.\* \*Cancer Res.\* 1971;31:1860–1861.   
 - Original Ann Arbor staging reference.   
  
---  
  
### **2. PET/CT in Hodgkin’s Lymphoma**  
- **Barrington SF, et al. Role of imaging in the staging and response assessment of lymphoma.** \*Lancet Oncol.\* 2010;11(11):982–994.   
 - PET/CT is the gold standard for **detecting active lymphoma**, evaluating **relapse**, and measuring **SUVmax**.   
  
-  **Cheson BD, et al.** \*Recommendations for initial evaluation, staging, and response assessment of Hodgkin and non-Hodgkin lymphoma: The Lugano classification.\* \*J Clin Oncol.\* 2014;32:3059–3068.   
 - Updated guidelines for **PET-based staging** and extranodal involvement (the “E” designation).   
  
---  
  
### **3. SUVmax Interpretation**  
- **Juweid ME, Cheson BD.** \*Positron-emission tomography and assessment of cancer therapy.\* \*N Engl J Med.\* 2006;354:496–507.   
 - Explains how **SUVmax values** correlate with tumor activity.   
  
- **Meignan M, Gallamini A, Haioun C.** \*Report on the First International Workshop on Interim-PET Scan in Lymphoma.\* \*Leuk Lymphoma.\* 2009;50:1257–1260.   
  
---  
  
### **4. Relapsed Hodgkin’s Lymphoma**  
- **Moskowitz CH, et al.** \*Treatment strategies in relapsed and refractory Hodgkin lymphoma.\* \*Lancet Haematol.\* 2015;2:e442–e453.   
 - Discusses patterns of relapse, typical nodal involvement, and treatment options.   
  
---  
  
If you want, I can also make a **short PDF with the references and a visual summary of your PET/CT findings** so you can take it to your oncologist. This is often very helpful for discussion about **relapse and treatment planning**.   
  
Do you want me to prepare that?

Exported by ChatGPT Universal Exporter Enhanced v8.3.1
