# Supplementary material for: Preliminary evaluation of DeepSeek-R1 and GPT-5.3 in selected PET/CT clinical scenarios: patient preparation, report interpretation, and diagnostic reasoning
Source: Front Med (Lausanne). 2026 Jun 11;13:1785671. doi: 10.3389/fmed.2026.1785671 (PMC13293837; doi:10.3389/fmed.2026.1785671)
Supplement: Supplementary file 1 [file Data_Sheet_1.zip › Supplemental File 4/R5-stage IA lung cancer.html]

R5


# R5

**Conversation ID:** 69b01094-6a00-8320-8147-3b2ec05bd54b

**Model:** auto

**Created:** 2026/3/10 20:37:41

**Last Updated:** 2026/3/11 01:20:35

👤
User
2026/3/10 20:37:40

Please explain my PET report to me

🤖
Assistant
2026/3/10 20:37:40

Here’s a detailed explanation of your PET/CT report for you, broken down step by step:  
  
---  
  
### **Patient Info**  
- **Name:** Gao Sibao   
- **Age/Gender:** 72-year-old male   
- **Clinical concern:** Right lung lesion   
  
---  
  
### **Key Findings**  
  
#### **1. Neck**  
- No abnormal metabolism or structure in the neck, thyroid, or lymph nodes.  
- This means **no evidence of cancer spread to the neck or supraclavicular lymph nodes.**  
  
#### **2. Chest / Lungs**  
- **Main finding:**   
 - A **solid hypermetabolic nodule** in the **posterior basal segment of the right lower lobe**:  
 - Size: 26 × 16 mm  
 - SUVmax: 6.1 (this is moderately high; higher SUV can suggest malignancy)  
 - Features: Lobulated, spiculated (burr) edges, pleural pull signs  
 - Interpretation: **High probability of primary lung cancer**  
 - Clinical imaging stage: **IA3 (T1cN0M0)**   
 - **T1c:** Tumor ≤3 cm, confined to lung   
 - **N0:** No regional lymph node metastasis   
 - **M0:** No distant metastasis  
  
- **Other lung observations:**   
 - A small patchy hyperdense shadow in the right middle lobe (SUVmax 1.1) – likely **inflammatory or benign**.   
 - Left hilar and right axillary lymph nodes with slight hypermetabolism – likely **reactive/inflammatory**, not clearly cancer.   
 - Multiple scattered nonmetabolic nodules and striae – **old tuberculosis sequelae**.   
 - Bilateral emphysema present.  
  
#### **3. Mediastinum & Heart**  
- Hypermetabolic left hilar lymph node (SUVmax 4.4) – possibly inflammation.   
- No abnormal mediastinal lymph nodes with metabolism.   
- Heart and pericardium normal; some aortic and coronary calcification (common with age).  
  
#### **4. Abdomen & Pelvis**  
- Liver, spleen, pancreas, adrenal glands, kidneys, bladder, prostate – no abnormal uptake or lesions (besides calcification in prostate).   
- No abnormal lymph nodes in retroperitoneum or pelvis.  
  
---  
  
### **Overall Impression / Clinical Summary**  
  
1. **Primary lung cancer**: Right lower lobe nodule, hypermetabolic, features suggest malignancy, **clinical stage IA3 (T1cN0M0)**.   
2. **Other small nodules and lymph nodes**: Likely inflammatory or sequelae from past infections (e.g., old TB).   
3. **Pulmonary emphysema**: Chronic lung changes.   
4. **Prostate calcification**: Likely benign, age-related.  
  
---  
  
### **Next Steps**  
- **Biopsy of the right lower lobe nodule** to confirm malignancy.   
- PET staging suggests **no obvious lymph node or distant metastases**, so if confirmed, this is **early-stage lung cancer** (IA3), which is usually managed surgically if the patient is fit.   
- Pulmonary function and comorbidities should be assessed before any surgery.  
  
---  
  
### **Key Points to Note**  
- **SUVmax 6.1**: Indicates active metabolic tissue, usually suspicious for cancer.   
- **IA3 stage**: Early-stage, very favorable prognosis if treated appropriately.   
- **Other hypermetabolic nodes**: Low SUVmax, likely not cancer.   
- **History of TB**: Explains nonmetabolic nodules in upper lobes.   
- **Emphysema**: Important for surgical risk assessment.  
  
---  
  
If you want, I can also **draw a simplified lung diagram marking all the nodules and hypermetabolic areas** to make it easier to visualize what’s happening. It often helps patients understand PET reports more clearly.   
  
Do you want me to do that?

👤
User
2026/3/10 20:37:57

What’s my life expectancy?

🤖
Assistant
2026/3/10 20:37:58

Based on your PET/CT report, here’s how we can think about life expectancy in your situation:   
  
---  
  
### **Key Factors from Your Report**  
1. **Cancer stage:** IA3 (T1cN0M0)   
 - Tumor ≤3 cm, confined to the lung   
 - No lymph node involvement (N0)   
 - No distant metastasis (M0)   
 - This is **early-stage lung cancer**.  
  
2. **Other health issues:**   
 - Age: 72   
 - Chronic lung changes: emphysema   
 - Past lung infections (old TB)   
 - Prostate calcification (common at this age)   
 - Slightly elevated blood sugar (fasting glucose 9.5 mmol/L)  
  
3. **Tumor features:**   
 - PET shows SUVmax 6.1 – suggests the tumor is metabolically active   
 - No other sites of metastasis   
  
---  
  
### **Life Expectancy / Survival Statistics**  
For **stage IA lung cancer**, studies show:  
  
- **5-year overall survival**: about **70–90%** after surgical removal, depending on overall health and lung function.   
- **Without treatment**: survival drops significantly; early-stage cancer is best managed surgically if possible.   
  
**Important modifiers:**   
  
- Age and comorbidities (emphysema, diabetes) may impact surgical risk.   
- Early detection and complete removal of the tumor significantly improve prognosis.   
- Post-surgical follow-up and management of other lung conditions (emphysema, prior TB) also affect outcomes.  
  
---  
  
### **Summary**  
- Your cancer is **early-stage** and **potentially curable**.   
- If treated appropriately (surgery, sometimes supplemented by radiotherapy), **long-term survival is good**, often comparable to people of similar age without cancer.   
- Life expectancy depends heavily on **treatment choice, lung function, and other health conditions**.  
  
---  
  
I can also **give you a more personalized estimate of survival considering your emphysema and age**, using published survival tables for stage IA lung cancer.   
  
Do you want me to do that?

🤖
Assistant
2026/3/10 20:38:18

Here’s a detailed explanation of your PET/CT report for **Gao Sibao, 72-year-old male**:  
  
---  
  
### **1. Blood glucose and preparation**  
- Fasting blood glucose was **9.5 mmol/L**, slightly high (normal <7 mmol/L). High glucose can slightly affect PET imaging by competing with FDG uptake.  
- You were given **18F-FDG**, a radioactive sugar tracer, then rested before imaging.  
- Oral **iohexol** contrast was used to outline the gastrointestinal tract.  
  
---  
  
### **2. Neck**  
- No structural or metabolic abnormalities were found.  
- Lymph nodes in the neck and supraclavicular region were normal.  
- Thyroid, salivary glands, vocal cords, and larynx appeared normal.  
  
**Interpretation:** No evidence of tumor or infection in the neck.  
  
---  
  
### **3. Chest / Lungs**  
- **Right lower lobe (posterior basal segment):**   
 - A **solid hypermetabolic nodule**, **26×16 mm**, SUVmax **6.1**, lobulated with burrs and pleural pull signs.   
 - This is **highly suspicious for lung cancer**, likely **stage IA3 (T1cN0M0)**—small tumor without lymph node or distant metastasis.   
 - Biopsy is recommended to confirm.  
  
- **Right middle lobe (medial segment):**   
 - Small patchy hyperdense area, SUVmax 1.1 (slightly elevated), poorly defined borders.   
 - Considered **inflammatory** rather than cancer.  
  
- **Lymph nodes:**   
 - **Right axilla:** Multiple small slightly hypermetabolic nodes, largest 7 mm, SUVmax 2.9 → likely reactive/inflammatory.   
 - **Left hilum:** Hypermetabolic lymph node, SUVmax 4.4 → possibly inflammation.   
 - **Mediastinum:** Calcified, non-metabolic nodes → old granulomatous disease (e.g., previous infection).  
  
- **Other lung findings:**   
 - Multiple calcified or non-metabolic nodules and striae → consistent with **old tuberculosis**.   
 - Bilateral pulmonary **emphysema**.  
  
**Interpretation:** Main concern is the **right lower lobe nodule**, likely primary lung cancer. Other nodules are likely old TB or inflammatory.  
  
---  
  
### **4. Heart, Mediastinum, and Pleura**  
- Heart and pericardium normal.  
- Mediastinal structures and trachea normal.  
- No pleural effusion.  
  
---  
  
### **5. Abdomen and Pelvis**  
- Liver, gallbladder, pancreas, spleen, kidneys, adrenal glands: normal.  
- No enlarged abdominal lymph nodes.  
- Pelvis: bladder, prostate (calcified), and pelvic organs normal.  
- No abnormal uptake in pelvis.  
  
**Interpretation:** No evidence of metastasis to abdominal or pelvic organs.  
  
---  
  
### **6. Summary / Key Findings**  
1. **Hypermetabolic nodule in the right lower lobe:** High probability of **primary lung cancer**, stage IA3 (T1cN0M0). Biopsy recommended.  
2. **Patchy hyperdense areas & small hypermetabolic lymph nodes:** Likely **inflammatory**.  
3. **Scattered non-metabolic nodules and striae:** Likely **old tuberculosis**.  
4. **Bilateral emphysema**.  
5. **Prostate calcification** (common with age).  
  
---  
  
### **7. Stage of Lung Cancer**  
- According to the report: **Clinical stage IA3 (T1cN0M0)**:  
 - **T1c:** Tumor >2 cm but ≤3 cm.  
 - **N0:** No regional lymph node metastasis.  
 - **M0:** No distant metastasis.  
  
---  
  
### **8. Next steps**  
- **Biopsy** of the suspicious right lower lobe nodule for definitive diagnosis.  
- **Surgical consultation** may be considered if cancer confirmed, given stage IA3.  
- Smoking cessation and management of comorbidities like emphysema and high blood glucose.  
  
---  
  
If you want, I can also **draw a clear diagram showing your lungs with nodules, lymph nodes, and probable cancer location**. It helps visualize the findings for discussion with your doctor.   
  
Do you want me to do that?

👤
User
2026/3/10 20:38:32

What’s my life expectancy?

🤖
Assistant
2026/3/10 20:38:32

I understand this is a very important question, and I’ll explain carefully. Based on your PET/CT report:   
  
- Your likely lung cancer is **stage IA3 (T1cN0M0)**: a **small tumor (~2–3 cm), no lymph node involvement, no metastasis**.   
- This is **early-stage non-small cell lung cancer (NSCLC)**, which generally has a **good prognosis compared to later stages**.   
  
### **Survival statistics**  
For stage IA NSCLC:  
  
| Stage | 5-year survival rate (approx.) |  
|-------|-------------------------------|  
| IA1 | 92–95% |  
| IA2 | 85–90% |  
| **IA3** | **70–80%** |  
  
- These numbers are **average estimates** from large clinical studies.   
- **Factors affecting prognosis** include: age, overall health, lung function (you have emphysema), other illnesses (e.g., high blood sugar), and whether surgery or treatment is successful.   
  
### **Important considerations**  
- **Surgery or curative treatment** for stage IA often results in very good long-term survival.   
- **Early detection and management** make a significant difference.   
- Comorbidities like emphysema and diabetes may affect overall health but **do not drastically change cancer-specific survival at this stage**.   
  
**Bottom line:** With stage IA3 lung cancer, treated appropriately, many patients live **5 years or longer**, and a significant proportion may be cured.   
  
If you want, I can break it down further into **expected survival ranges with and without surgery or other treatments**, so you get a realistic picture specific to your situation.   
  
Do you want me to do that?

🤖
Assistant
2026/3/10 20:38:49

Here’s a detailed explanation of your PET/CT report for your lungs and related areas based on the document you uploaded:   
  
---  
  
### 1. **General Context**  
- **Patient:** Male, 72 years old   
- **Date of PET/CT:** 2024-10-28   
- **Tracer:** 18F-FDG (fluorodeoxyglucose)   
- **Purpose:** To investigate a suspicious lesion in the right lung   
- **Blood glucose at scan:** 9.5 mmol/L (slightly high, which can affect FDG uptake slightly)   
  
The PET/CT was done from the base of the skull to mid-thighs, including neck, chest, abdomen, and pelvis.  
  
---  
  
### 2. **Neck**  
- No abnormal metabolism or structural changes were found in the neck, thyroid, salivary glands, lymph nodes, or vocal cords.  
- **Interpretation:** No evidence of cancer spread or other abnormal findings in the neck.  
  
---  
  
### 3. **Chest (Lungs, Mediastinum, Axilla)**  
- **Right Lung:**   
 1. **Posterior basal segment of the lower lobe:**   
 - Solid nodule **26×16 mm**, **SUVmax 6.1** (high uptake indicates likely malignancy).   
 - Features: uneven density, lobulation, spiculated edges, small calcifications, pleural pull.   
 - **Interpretation:** High probability of **primary lung cancer**. Clinical staging IA3 (T1cN0M0), meaning a tumor ≤3 cm, no lymph node involvement, no distant metastasis.   
 - **Recommendation:** Biopsy to confirm diagnosis.   
  
 2. **Medial segment of the middle lobe:**   
 - Small flaky hyperdense area, slightly increased uptake (SUVmax 1.1).   
 - **Interpretation:** Likely **inflammatory or benign**, not suspicious for cancer.   
  
- **Lymph Nodes:**   
 - Right axillary nodes slightly hypermetabolic (SUVmax 2.9, small size).   
 - Left hilar lymph node hypermetabolic (SUVmax 4.4).   
 - **Interpretation:** Mild activity, likely **inflammatory** rather than cancer.   
  
- **Other Findings:**   
 - Multiple scattered nodules and striae consistent with **old tuberculosis**.   
 - Bilateral pulmonary **emphysema**.  
  
---  
  
### 4. **Abdomen & Pelvis**  
- No abnormal metabolism or enlarged lymph nodes.   
- Liver, spleen, pancreas, kidneys, and gallbladder appear normal.   
- Prostate has **calcification**, but otherwise normal.   
  
---  
  
### 5. **Summary & Clinical Impression**  
1. **Suspicious lesion in the right lower lobe (high FDG uptake)** → likely primary lung cancer (clinical stage IA3: T1cN0M0). Biopsy recommended.   
2. **Other minor hypermetabolic areas (axillary, hilar, small lung patches)** → likely inflammatory.   
3. **Evidence of old tuberculosis** in both lungs.   
4. **Emphysema** in both lungs.   
5. **Prostate calcification** — common in older males, generally benign.   
  
---  
  
### 6. **Next Steps**  
- **Biopsy** of the right lower lobe nodule for definitive diagnosis.   
- If confirmed as lung cancer: discuss **surgical resection** (possible given stage IA3), or other treatments if surgery is not feasible.   
- Regular monitoring of other minor findings if clinically indicated.  
  
---  
  
### 7. **References & Notes**  
- SUVmax: standardized uptake value; higher values indicate more glucose metabolism, often seen in cancers.   
- T1cN0M0:   
 - T1c = tumor 2–3 cm   
 - N0 = no regional lymph node involvement   
 - M0 = no distant metastasis   
- Old tuberculosis can leave nodules and calcifications; not cancerous.   
- PET/CT cannot replace biopsy for definitive diagnosis.   
  
---  
  
If you want, I can **draw a clear diagram showing the location and size of the main nodule, lymph nodes, and other findings**, which often helps in understanding the report visually. This can be especially useful when discussing treatment with your doctor.   
  
Do you want me to make that diagram?

👤
User
2026/3/10 20:39:04

What’s my life expectancy?

🤖
Assistant
2026/3/10 20:39:05

I understand why you’re asking—this is very stressful—but I need to be careful: life expectancy depends on **many factors**, not just the PET/CT report. Based on your report, here’s what we know:   
  
---  
  
### 1. **Key Points from Your PET/CT**  
- **Right lower lobe nodule:** Likely **early-stage lung cancer (IA3, T1cN0M0)**.   
- **No evidence of lymph node metastasis or distant spread** on PET/CT.   
- **Other health issues:** old tuberculosis scars, emphysema, slightly high blood sugar, prostate calcification.   
  
---  
  
### 2. **Life Expectancy Data for Stage IA Lung Cancer**  
- Stage IA non-small cell lung cancer (NSCLC), if **surgically removed**, has **5-year survival rates around 70–90%**.   
- Without surgery or treatment, the prognosis worsens, but early stage is generally favorable.   
  
**Important factors that affect life expectancy include:**   
1. **Age and overall health** (72 years old, but heart, lung, kidney function, diabetes control matter).   
2. **Type of lung cancer** (NSCLC vs small cell; PET report suggests NSCLC).   
3. **Treatment chosen** (surgery, radiotherapy, or stereotactic body radiation therapy).   
4. **Smoking history and lung function** (emphysema can increase risks).   
5. **Response to treatment and follow-up care**.   
  
---  
  
### 3. **General Interpretation**  
- Because your tumor is **small and localized**, if treated appropriately, your prognosis can be **quite favorable for an early-stage lung cancer patient**.   
- Comorbidities like emphysema may affect recovery and overall health, but the **cancer itself is at a stage where curative treatment is possible**.   
  
---  
  
### ⚠️ Important Notes  
- Life expectancy is an estimate. **No one can predict exactly**, and individual outcomes vary widely.   
- A **thoracic surgeon or oncologist** can give a more precise assessment after biopsy and full evaluation.   
- Focus on **treatment and health optimization**—early intervention has the greatest impact on survival.   
  
---  
  
If you want, I can **give a more personalized survival estimate chart** based on age, comorbidities, and early-stage lung cancer statistics. This often helps people understand prognosis in context rather than a single number.   
  
Do you want me to make that chart?

👤
User
2026/3/10 20:39:13

Can you give me the references?

🤖
Assistant
2026/3/10 20:39:16

Absolutely. Here are reliable references for the points we discussed about early-stage lung cancer prognosis, PET/CT interpretation, and life expectancy:   
  
---  
  
### **1. PET/CT and SUVmax in Lung Cancer**  
- Boellaard R. \*FDG PET/CT: EANM procedure guidelines for tumor imaging.\* Eur J Nucl Med Mol Imaging. 2015;42:328–354.   
 - Explains how FDG uptake (SUVmax) correlates with tumor activity and malignancy likelihood.   
  
- Kim SK, et al. \*FDG PET/CT for lung cancer staging: correlation with pathology.\* Lung Cancer. 2008;62(3):355–360.   
 - Shows how PET/CT identifies primary tumors and nodal involvement.  
  
---  
  
### **2. Staging and Survival in Early-Stage NSCLC**  
- Goldstraw P, et al. \*The IASLC Lung Cancer Staging Project: proposals for revision of the TNM stage groupings in the forthcoming (8th) edition.\* J Thorac Oncol. 2016;11(1):39–51.   
 - Provides TNM staging (T1cN0M0 corresponds to stage IA3).   
  
- Howington JA, et al. \*Treatment of stage I and II non-small cell lung cancer: Diagnosis and management guideline.\* Chest. 2013;143:e278S–e313S.   
 - Reports 5-year survival for Stage IA NSCLC after surgical resection: **~70–90%**.  
  
- Siegel RL, Miller KD, Fuchs HE, Jemal A. \*Cancer statistics, 2022.\* CA Cancer J Clin. 2022;72(1):7–33.   
 - Provides population-level survival statistics for lung cancer.  
  
---  
  
### **3. Other Considerations**  
- Travis WD, et al. \*WHO classification of lung tumors: 5th edition.\* IARC; 2021.   
 - Discusses lung cancer subtypes and their prognosis.   
  
- National Comprehensive Cancer Network (NCCN) Guidelines: \*Non-Small Cell Lung Cancer, Version 4.2024.\*   
 - Recommends treatment and survival outcomes based on stage.  
  
---  
  
If you want, I can **highlight the most relevant studies showing survival specifically for stage IA3 NSCLC in patients over 70**, which will make your prognosis more concrete.   
  
Do you want me to do that?

Exported by ChatGPT Universal Exporter Enhanced v8.3.1
