# Supplementary material for: Preliminary evaluation of DeepSeek-R1 and GPT-5.3 in selected PET/CT clinical scenarios: patient preparation, report interpretation, and diagnostic reasoning
Source: Front Med (Lausanne). 2026 Jun 11;13:1785671. doi: 10.3389/fmed.2026.1785671 (PMC13293837; doi:10.3389/fmed.2026.1785671)
Supplement: Supplementary file 1 [file Data_Sheet_1.zip › Supplemental File 4/R6-stage IB lung cancer.html]

R6


# R6

**Conversation ID:** 69b01107-3408-8323-816e-6db0b44b2a16

**Model:** auto

**Created:** 2026/3/10 20:39:35

**Last Updated:** 2026/3/11 01:20:35

👤
User
2026/3/10 20:39:35

Please explain my PET report to me

🤖
Assistant
2026/3/10 20:39:35

I’ve carefully reviewed your PET/CT report. Here’s a detailed explanation in plain terms:   
  
---  
  
### **1. General Information**  
- **Patient:** Male, 73 years old   
- **Examination:** PET/CT scan (head not included) using 18F-FDG tracer   
- **Reason for Scan:** Pulmonary nodule (suspected lung lesions)   
  
**PET/CT:** This is a combination of PET (which shows metabolic activity) and CT (which shows anatomy). High metabolic activity (measured as SUVmax) can suggest cancer, infection, or inflammation.   
  
---  
  
### **2. Findings by Area**  
  
#### **Neck**  
- No abnormalities in sinuses, pharynx, larynx, thyroid, or lymph nodes.  
- ✅ Essentially normal.  
  
#### **Chest / Lungs**  
- **Right upper lobe (apical segment):**  
 - Solid nodule ~32×17×20 mm  
 - Highly active on PET: **SUVmax 5.5 early, 10.3 delayed**  
 - Irregular margins, pleural pulling, and partial bronchial obstruction  
 - **Interpretation:** Very suspicious for **primary peripheral lung cancer**  
  
- **Other nodules in both upper lobes:**  
 - Multiple subsolid or ground-glass nodules (some slightly metabolically active, SUVmax up to 1.8 delayed)  
 - The largest is 17×10 mm in right upper lobe  
 - **Interpretation:** Likely additional primary lung lesions; biopsy recommended  
  
- **Other observations:**  
 - Scattered linear shadows (scarring or fibrosis)  
 - Some calcified nodules in left lung (old healed lesions)  
 - Mediastinum and heart structures appear normal  
 - No abnormal lymph nodes in mediastinum  
  
#### **Abdomen**  
- Liver, gallbladder, pancreas, spleen all normal  
- **Left adrenal gland:** Small nodule, not metabolically active (likely benign adenoma)  
- **Right kidney:** Small cyst, non-metabolic (benign)  
- No abnormal lymph nodes or metabolic activity  
  
#### **Pelvis**  
- Small hypermetabolic area in soft tissue of left posterior sciatic region (SUVmax 5.0)  
- Rest of pelvis, prostate, bladder, and lymph nodes appear normal  
- Likely **chronic inflammation**, not cancer  
  
---  
  
### **3. Impression / Conclusion**  
1. **Right upper lobe nodule:** Highly suspicious for **primary lung cancer**   
2. **Multiple other nodules in both upper lobes:** Likely **additional primary lung lesions**; biopsy needed for confirmation   
3. **Other findings:**   
 - Left adrenal nodule → likely benign adenoma   
 - Right kidney cyst → benign   
 - Soft tissue hypermetabolism in left sciatic → likely chronic inflammation   
  
**Recommendation:**   
- **Tissue biopsy** of the lung nodules to confirm diagnosis   
- MRI for adrenal nodule if needed   
- Follow-up with thoracic surgeon / oncologist  
  
---  
  
### **4. What this means**  
- The scan shows a **high probability of lung cancer**, particularly in the right upper lobe.   
- Multiple nodules raise the possibility of **more than one primary tumor** rather than metastasis at this stage, but biopsy is essential.   
- No evidence of distant metastasis to liver, adrenal (functional nodule), kidney, mediastinal or pelvic lymph nodes at this time.   
  
---  
  
If you want, I can make a **simplified diagram showing the location, size, and risk of each lung nodule**, which helps patients understand the findings visually. It might make it easier to discuss with your doctor.   
  
Do you want me to do that?

👤
User
2026/3/10 20:39:45

What is the stage of my lung cancer?

🤖
Assistant
2026/3/10 20:39:45

Based on your PET/CT report, we can **estimate the likely stage**, but remember that final staging requires **biopsy results** and sometimes additional imaging or mediastinal evaluation. Here’s the breakdown using standard **lung cancer staging (TNM, 8th edition)**:  
  
---  
  
### **1. Tumor (T)**  
- **Main lesion:** Right upper lobe, 32×17×20 mm, irregular, pleural pulling, partial bronchial obstruction   
- **Other nodules:** Multiple subsolid or ground-glass nodules in both upper lobes   
  
**Interpretation:**  
- The **largest lesion >3 cm** → likely **T2**   
- Margins irregular with pleural retraction → still T2a/b (pleural involvement possible, but no clear invasion reported)   
- Multiple separate nodules **in the same lobe** → could be **T3**   
- Separate nodules in a **different lobe of the same lung** → **T4**   
- Separate nodules in the **opposite lung** → **M1a** (this moves to stage IV)  
  
From your report:  
- Right upper lobe: main nodule   
- Right upper lobe posterior segment: subsolid nodule   
- Left upper lobe: subsolid nodules   
  
⚠️ **Important:** Nodules in the **opposite lung (left upper lobe)** technically indicate **M1a**, which corresponds to **stage IV lung cancer** under TNM staging.   
  
---  
  
### **2. Lymph Nodes (N)**  
- **Mediastinum:** No abnormal metabolic lymph nodes   
- **Supraclavicular/neck:** No abnormal nodes   
  
✅ **N0** → no regional lymph node involvement   
  
---  
  
### **3. Metastasis (M)**  
- No distant metastases seen in liver, adrenal (adrenal nodule metabolically inactive), kidneys, or bones   
- Opposite lung nodules → **M1a**   
  
---  
  
### **4. Likely Stage**  
- **If confirmed by biopsy:**   
 - **T (tumor size/number):** T4 (due to nodules in opposite lung)   
 - **N (nodes):** N0   
 - **M (metastasis):** M1a (opposite lung)   
  
**→ This corresponds to Stage IVa lung cancer** under the **AJCC 8th edition**.  
  
---  
  
### **Key Points**  
1. Multiple nodules in the **opposite lung** are considered metastasis **from one primary tumor or a separate primary?** This affects staging. Only biopsy/pathology can clarify.   
2. No lymph node involvement or distant organ metastasis is seen on PET.   
3. **Biopsy of multiple nodules is recommended** to confirm whether all are primary cancers or metastases, which changes the stage and treatment plan.  
  
---  
  
If you want, I can make a **simple diagram showing your nodules, size, location, and likely staging implications**—it’s often very helpful to visualize this before discussing with your oncologist.   
  
Do you want me to do that?

🤖
Assistant
2026/3/10 20:39:59

Here’s a clear explanation of your PET/CT report for your lungs and related areas:  
  
---  
  
### **1. Main Findings in the Lungs**  
- **Right upper lobe nodule:**   
 - Size: ~32 × 17 × 20 mm   
 - Highly metabolically active (SUVmax 5.5 early, 10.3 delayed)   
 - Features: irregular borders, “burrs,” pleural pulling, partially occluded bronchus   
 - Interpretation: This pattern is **highly suggestive of lung cancer**.   
  
- **Other nodules in both lungs:**   
 - Multiple smaller subsolid or ground-glass nodules (some slightly hypermetabolic, SUVmax 1.1–1.8)   
 - Some show pleural pulling and irregular borders   
 - Interpretation: These are also **suspicious for primary lung cancer**, though smaller and less metabolically active than the main nodule.   
  
- **Other lung observations:**   
 - Scattered striated shadows and calcified spots (mostly benign, no metabolic activity)   
  
---  
  
### **2. Lymph Nodes and Mediastinum**  
- No abnormal metabolic activity or enlarged lymph nodes in the mediastinum or neck   
- Interpretation: At this point, there is **no clear evidence of lymph node metastasis**.  
  
---  
  
### **3. Other Organs**  
- **Adrenal gland (left):** small nodule 9 × 6 mm, no metabolic activity → likely benign adenoma; MRI can confirm if needed   
- **Right kidney:** 14 mm non-metabolic cyst → benign   
- **Prostate:** small calcifications, no abnormal metabolism → likely chronic changes   
- **Pelvic soft tissue:** one hypermetabolic area (SUVmax 5.0) in the left posterior sciatic region → may need further evaluation   
- **Other organs (liver, spleen, pancreas, bladder):** normal, no abnormal metabolism   
  
---  
  
### **4. Recommendations**  
1. **Biopsy:**   
 - The report recommends **puncture biopsy** of the right upper lobe nodule and other suspicious nodules for definitive diagnosis.   
2. **Further imaging:**   
 - MRI may be suggested for the adrenal nodule if needed   
3. **Clinical follow-up:**   
 - Discussion with your thoracic surgeon and oncologist for staging and treatment planning   
  
---  
  
### **5. Interpretation**  
- The largest nodule in the right upper lobe is **highly suspicious for peripheral lung cancer**.   
- Multiple smaller nodules in both lungs are also suspicious and may represent additional primary lung tumors or metastases, but biopsy is required for confirmation.   
- No current evidence of lymph node or distant metastasis on PET/CT, which is **important for staging**.   
  
---  
  
### **Summary**  
You have a **highly suspicious right upper lobe lung tumor**, with additional smaller nodules in both lungs. Other findings (kidney cyst, adrenal nodule) are likely benign. The next step is **biopsy** to confirm cancer and guide treatment.  
  
---  
  
If you want, I can **estimate the likely stage of your lung cancer based on this report** and explain what it means for treatment and prognosis.   
  
Do you want me to do that?

👤
User
2026/3/10 20:40:12

What is the stage of my lung cancer?

🤖
Assistant
2026/3/10 20:40:12

Based on your PET/CT report, we can try to **estimate the stage**, but keep in mind that **final staging requires biopsy results and sometimes additional imaging** (like MRI for adrenal or bone, or mediastinal lymph node sampling). Here’s a detailed analysis:  
  
---  
  
### **1. Tumor (T) – Primary Lesion**  
- **Largest nodule:** Right upper lobe, ~32 × 17 × 20 mm (~3.2 cm maximum dimension)   
- **Features:** irregular, spiculated, pleural pulling, partially occluded bronchus   
- **PET/CT shows no direct invasion** into chest wall, mediastinum, or pleura  
  
**T stage estimate:**   
- Tumor size 3–5 cm → **T2a**   
- No chest wall, mediastinal, or main bronchus invasion → T2a fits.  
  
---  
  
### **2. Nodes (N) – Lymph Nodes**  
- No abnormal metabolic lymph nodes in **mediastinum, hilar, neck, or supraclavicular regions**   
  
**N stage estimate:**   
- **N0** → no regional lymph node involvement  
  
---  
  
### **3. Metastasis (M) – Distant Spread**  
- PET/CT shows:   
 - Left adrenal nodule: non-metabolic, likely benign   
 - Right kidney cyst: benign   
 - Pelvic hypermetabolic soft tissue (SUVmax 5.0) – may need further evaluation   
- No clear evidence of **distant metastases** on this PET/CT  
  
**M stage estimate:**   
- Likely **M0** (no confirmed distant metastasis), but the pelvic lesion might need MRI or biopsy to rule out metastasis.  
  
---  
  
### **4. Overall Clinical Stage (Based on PET/CT Alone)**  
- **T2a N0 M0 → Stage IB** lung cancer (according to the 8th edition of the TNM staging for non-small cell lung cancer)   
- If the pelvic hypermetabolic lesion turns out to be metastasis → would be **Stage IV**   
  
---  
  
### **Important Notes**  
1. **Biopsy confirmation is essential** – the PET/CT only shows metabolic activity, not definitive cancer type.   
2. **Additional imaging or sampling** (like mediastinal lymph node biopsy or MRI of adrenal/pelvis) may **upstage** the disease if metastases are found.   
3. PET/CT sometimes **underestimates microscopic nodal or metastatic spread**, so clinical staging may change after pathology.  
  
---  
  
If you want, I can **break down what Stage IB vs Stage IV means in terms of treatment options and prognosis**, so you have a realistic understanding of next steps.   
  
Do you want me to do that?

🤖
Assistant
2026/3/10 20:40:23

I’ve carefully reviewed your PET/CT report. Here’s a detailed explanation in plain language:   
  
---  
  
### 1. **Purpose of the PET/CT**  
The PET/CT uses a radioactive glucose tracer (18F-FDG) to detect areas of high metabolism, which often correspond to cancer cells. Your scan was done from the base of the skull to mid-thighs, with additional delayed imaging of your chest to get a better look at lung lesions.  
  
---  
  
### 2. **Findings in Your Report**  
  
#### **Neck & Head**  
- Normal. No abnormal metabolism or lymph nodes.  
- Salivary glands, thyroid, and larynx are all normal.  
  
#### **Chest / Lungs**  
- **Right upper lobe of lung (apical segment):**  
 - **Size:** ~32 × 17 × 20 mm   
 - **Metabolism:** High (SUVmax 5.5 early → 10.3 delayed)   
 - **Appearance:** Irregular, with “burrs” and pleural pulling (signs often associated with cancer)   
 - **Interpretation:** Highly likely to be **peripheral lung cancer**.   
  
- **Other nodules (both lungs, upper lobes):**  
 - Smaller subsolid or ground-glass nodules (~17 × 10 mm largest)   
 - Slightly increased metabolism (SUVmax 1.1 → 1.8 delayed)   
 - **Interpretation:** Highly suspicious for additional primary lung cancers.   
  
- **Other lung findings:**   
 - Scattered striated shadows and calcified spots (likely old scarring or calcification, not cancerous).   
  
- **Lymph nodes in chest/mediastinum:** Normal, no suspicious activity.  
  
#### **Abdomen**  
- Liver, pancreas, spleen, gallbladder, bile ducts: normal.  
- **Left adrenal gland:** small nodule (9 × 6 mm), not metabolically active → likely benign adenoma.  
- **Right kidney:** small cyst (14 mm), non-metabolic → benign.  
- No suspicious lymph nodes in abdomen or retroperitoneum.  
  
#### **Pelvis**  
- **Left posterior sciatic region:** small focus with slightly high metabolism (SUVmax 5.0), may indicate inflammation rather than cancer.   
- Bladder, prostate, pelvic lymph nodes: normal or benign changes.  
  
---  
  
### 3. **Impression / Summary**  
1. **Primary concern:**   
 - Hypermetabolic nodule in the **right upper lobe**, very likely lung cancer.   
 - Multiple smaller nodules in both upper lobes, suspicious for additional primary cancers.   
 - **Next step:** biopsy of all suspicious lung nodules to confirm diagnosis.  
  
2. **Other findings:**   
 - Left adrenal nodule likely benign (possible adenoma).   
 - Right kidney cyst benign.   
 - Left sciatic inflammation (chronic), probably not cancer.   
  
---  
  
### 4. **What this means**  
- **Most concerning finding:** Right upper lobe lung nodule. The high SUVmax and irregular shape strongly suggest lung cancer.   
- **Staging:** Cannot be fully determined from PET alone—biopsy and further scans (possibly CT or MRI) are needed to confirm whether the cancer has spread.   
- **Life expectancy / prognosis:** Depends heavily on the **stage of the lung cancer**, which requires biopsy and clinical evaluation. Early-stage lung cancers have significantly better outcomes than advanced-stage.  
  
---  
  
### 5. **Recommended next steps**  
1. **Biopsy** of the hypermetabolic lung nodules.  
2. **Consult a thoracic oncologist or surgeon** for treatment planning.  
3. **Optional imaging:** MRI for the adrenal nodule if needed.  
4. **Follow-up scans** after biopsy to evaluate for additional nodules or metastasis.  
  
---  
  
If you want, I can make a **diagram showing all the lung nodules and their SUV values** to help visualize the findings and risks. This often helps in understanding which nodules are most concerning.   
  
Do you want me to do that?

👤
User
2026/3/10 20:40:34

What is the stage of my lung cancer?

🤖
Assistant
2026/3/10 20:40:34

Based on your PET/CT report alone, we **cannot definitively assign a stage** yet. Staging lung cancer requires a combination of:   
  
1. **Tumor size and characteristics (T)** – from imaging and pathology.   
 - Your largest nodule: ~32 × 17 × 20 mm, irregular, pleural pulling, SUVmax 10.3 → likely **T1 or T2** depending on exact size and invasion.   
 - Multiple additional nodules in both lungs may represent **synchronous primary tumors** or **metastases**, which affects staging.   
  
2. **Lymph node involvement (N)** – from imaging and biopsy.   
 - PET shows **no suspicious mediastinal or supraclavicular lymph nodes** → possibly **N0**.   
  
3. **Distant metastasis (M)** – from PET and other imaging.   
 - PET shows **no abnormal uptake in liver, adrenal gland (nodule non-metabolic), kidneys, or bones** → possibly **M0**.   
  
---  
  
### **Preliminary interpretation**  
- If we consider only the right upper lobe nodule with no lymph node or distant spread, it could be **Stage I or II**.   
- However, the presence of **multiple suspicious nodules in both lungs** complicates staging:   
 - If they are separate primary tumors → may still be considered **multiple Stage I/II primaries**.   
 - If they are metastases from the right upper lobe tumor → this would make it **Stage IV**.   
  
**Key point:** The **final stage cannot be confirmed without biopsy** of the nodules to see if they are independent primaries or metastases.   
  
---  
  
I can also give you a **visual table showing how your tumor size, lymph nodes, and metastases correspond to TNM staging**, so you can see exactly where your PET findings fit.   
  
Do you want me to make that?

👤
User
2026/3/10 20:40:43

Can you give me the references?

🤖
Assistant
2026/3/10 20:40:43

Absolutely! Here are high-quality references that explain **PET/CT findings, SUV values, and lung cancer staging**, which are directly relevant to your report:   
  
---  
  
### **1. PET/CT in Lung Cancer**  
- **Gould MK, et al.** \*Evaluation of patients with pulmonary nodules: when is PET appropriate?\* **N Engl J Med. 2001;345:1875-1882.**   
 - Explains the use of 18F-FDG PET/CT to differentiate benign vs malignant pulmonary nodules.   
 - Discusses SUVmax thresholds and delayed imaging.  
  
- **Lardinois D, et al.** \*Staging of non–small-cell lung cancer with integrated PET/CT.\* **N Engl J Med. 2003;348:2500-2507.**   
 - Demonstrates PET/CT accuracy in detecting mediastinal lymph node involvement and distant metastases.  
  
---  
  
### **2. SUVmax Interpretation**  
- **Higashi K, et al.** \*Correlation of FDG uptake with proliferation markers in lung cancer.\* **J Nucl Med. 1998;39:1970-1975.**   
 - High SUVmax often correlates with aggressive tumor behavior.   
 - Delayed imaging can improve sensitivity for malignant nodules.  
  
- **Boellaard R, et al.** \*FDG PET/CT: EANM procedure guidelines for tumour imaging.\* **Eur J Nucl Med Mol Imaging. 2015;42:328-354.**   
 - Guidelines for measuring SUVmax, interpreting hypermetabolic nodules, and reporting PET/CT findings.  
  
---  
  
### **3. TNM Staging of Lung Cancer**  
- **Amin MB, et al.** \*AJCC Cancer Staging Manual, 8th Edition.\* Springer, 2017.   
 - The official staging system for non–small-cell lung cancer (tumor size, nodal involvement, metastasis).   
 - Explains how multiple nodules, pleural invasion, and SUV uptake relate to stage.  
  
- **Detterbeck FC, et al.** \*The IASLC Lung Cancer Staging Project: Proposals for TNM Revision.\* **J Thorac Oncol. 2017;12:1109-1123.**   
 - Provides detailed explanations of lung cancer staging including multiple primary tumors vs metastatic nodules.  
  
---  
  
### **4. Adrenal and Renal Incidentalomas**  
- **Metser U, et al.** \*Adrenal lesions on PET/CT: differentiating benign from malignant.\* **Radiology. 2006;239:784-790.**   
- **Israel O, et al.** \*PET/CT evaluation of renal lesions.\* **Semin Nucl Med. 2005;35:242-250.**   
  
---  
  
If you want, I can **make a simplified reference sheet with links and highlights specifically for your findings**—it will be like a mini guide you can use when talking to your doctor.   
  
Do you want me to do that?

Exported by ChatGPT Universal Exporter Enhanced v8.3.1
