# Supplementary material for: Preliminary evaluation of DeepSeek-R1 and GPT-5.3 in selected PET/CT clinical scenarios: patient preparation, report interpretation, and diagnostic reasoning
Source: Front Med (Lausanne). 2026 Jun 11;13:1785671. doi: 10.3389/fmed.2026.1785671 (PMC13293837; doi:10.3389/fmed.2026.1785671)
Supplement: Supplementary file 1 [file Data_Sheet_1.zip › Supplemental File 4/R8-stage IIB lung cancer.html]

R8


# R8

**Conversation ID:** 69b012a4-28a0-8320-b3cc-1538428ec092

**Model:** auto

**Created:** 2026/3/10 20:46:29

**Last Updated:** 2026/3/11 01:20:37

👤
User
2026/3/10 20:46:28

Please explain my PET report to me

🤖
Assistant
2026/3/10 20:46:29

I’ve reviewed your PET/CT report. Here’s a detailed explanation in plain language:  
  
---  
  
### **Patient Overview**  
- **Name:** Lee Yueh Cong   
- **Age:** 61, Female   
- **Clinical Concerns:** Right middle lobe lung mass (uncertain nature: tumor vs infection), pulmonary nodules in both lungs, type 2 diabetes.  
  
---  
  
### **Findings from the PET/CT**  
  
1. **Neck and Head**  
 - No abnormal metabolism or structure in the sinuses, pharynx, larynx, salivary glands, thyroid, or lymph nodes.   
 - ✅ This means no suspicious cancer or infection detected in these areas.  
  
2. **Chest / Lungs**  
 - **Right middle lobe mass:**   
 - **Size:** 37 × 22 × 25 mm (about 3.7 cm × 2.2 cm × 2.5 cm)   
 - **Metabolism:** Very high uptake of 18F-FDG (SUVmax 18.1), which is strongly suggestive of a cancerous tumor.   
 - **Appearance:** Irregular edges, lobulated, with vascular clusters and bronchial involvement. This is typical for malignant lung lesions.   
 - **Spread:** Invades nearby lung tissue (anterior segment of the right upper lobe) and touches the pleura.   
 - **Additional distal shadow:** Slightly hypermetabolic (SUVmax 2.2), possibly related to inflammation or partial collapse of the lung (atelectasis).  
 - **Other lung nodules:** Multiple tiny nodules in both lungs (largest 6 × 4 mm in right upper lobe). These are non-metabolic, meaning they are likely benign, but follow-up is recommended.  
  
 - **Mediastinum:** No enlarged or metabolically active lymph nodes.   
 - ✅ Suggests no obvious spread to central chest lymph nodes yet.  
  
3. **Heart and Large Vessels**  
 - Heart size and function appear normal; no metabolic abnormalities in the aorta or major branches.  
  
4. **Abdomen**  
 - Liver, gallbladder, pancreas, spleen, kidneys, adrenal glands all normal in size, shape, and metabolic activity.   
 - ✅ No evidence of cancer spread to abdominal organs.  
  
5. **Pelvis**  
 - Bladder, uterus, adnexa, and surrounding lymph nodes are normal.   
 - ✅ No signs of pelvic involvement.  
  
---  
  
### **Interpretation**  
1. **Primary Concern:**   
 - The hypermetabolic mass in the **right middle lobe** is **highly suggestive of lung cancer**. The PET findings combined with its invasive features support this.   
  
2. **Secondary Findings:**   
 - Multiple **small nodules in both lungs** are currently not metabolically active and likely benign, but monitoring is recommended.   
 - **Right breast calcifications** are noted, usually benign, but could be evaluated separately if needed.  
  
3. **Complications:**   
 - Distal atelectasis (partial collapse of lung tissue) near the mass, which can occur when a tumor obstructs airways.  
  
---  
  
### **Next Steps (Typical Clinical Approach)**  
- **Tissue confirmation:** A biopsy of the right lung mass is usually done to confirm the type of lung cancer.   
- **Staging:** Further tests may be needed to see if cancer has spread, even though PET/CT shows no obvious distant metastases.   
- **Treatment:** Options could include surgery, radiotherapy, chemotherapy, targeted therapy, or immunotherapy, depending on tumor type, stage, and overall health.   
  
---  
  
### **Summary in Simple Terms**  
- You have a suspicious tumor in the right middle lobe of your lung, which likely represents lung cancer.   
- No obvious spread to lymph nodes or other organs was seen on this PET/CT.   
- Tiny spots in both lungs are probably harmless but need follow-up.   
- Further confirmation with a biopsy and discussion with a lung cancer specialist is essential.  
  
---  
  
If you want, I can also **estimate the likely stage of your lung cancer based on this PET/CT** and explain what that means for treatment and prognosis.   
  
Do you want me to do that?

👤
User
2026/3/10 20:46:48

What is the stage of my lung cancer?

🤖
Assistant
2026/3/10 20:46:49

Based on your PET/CT report, we can make an **estimated staging** of your lung cancer, but remember **final staging requires a biopsy and sometimes additional imaging** (like brain MRI or bone scan). I’ll go step by step using the **TNM system** (Tumor, Node, Metastasis), which is standard for lung cancer staging.  
  
---  
  
### **Step 1: Tumor (T)**  
- **Location:** Right middle lobe   
- **Size:** ~3.7 cm in largest dimension   
- **Characteristics:** Irregular borders, lobulated, invading the **anterior segment of the right upper lobe** (crossing the horizontal fissure).   
  
✅ According to TNM (8th edition):  
- **T1:** ≤3 cm   
- **T2:** >3 cm but ≤5 cm, or invading visceral pleura, or causing partial lung collapse (atelectasis) **but not entire lung**   
- **T3:** Tumor >5 cm or invading adjacent lobe **or invading chest wall, diaphragm, or mediastinal structures**   
  
Your tumor is **3.7 cm and invading an adjacent lobe (upper lobe anterior segment)** → **T3**.  
  
---  
  
### **Step 2: Node (N)**  
- **Findings:** No abnormal lymph nodes detected in mediastinum, hilum, or supraclavicular regions.   
  
✅ So **N0** (no regional lymph node metastasis visible on PET/CT).  
  
---  
  
### **Step 3: Metastasis (M)**  
- **Findings:** No abnormal metabolic activity in liver, adrenal glands, pelvis, bones, or other organs.   
- ✅ **M0** (no distant metastasis detected).  
  
---  
  
### **Estimated Stage**  
- **TNM:** T3 N0 M0   
- **Stage:** **IIA or IIB** depending on exact T3 criteria   
 - For **T3 N0 M0** lung cancer, it is **Stage IIB** according to the AJCC 8th edition.  
  
---  
  
### **Important Notes**  
1. **This is an estimation from PET/CT only.** Final staging may change after:   
 - Biopsy of the tumor (to confirm type)   
 - Mediastinal lymph node evaluation (sometimes PET misses small metastases)   
 - Additional imaging (brain MRI or bone scan) if clinically indicated  
2. **Treatment depends on stage, tumor type, and patient health**, so your oncologist will combine PET results with biopsy results to decide.  
  
---  
  
If you want, I can **explain what Stage IIB right lung cancer usually means for treatment options and prognosis**, in clear terms. This will help you plan your next steps.   
  
Do you want me to do that?

🤖
Assistant
2026/3/10 20:47:06

I’ve reviewed your PET/CT report carefully. Here’s a clear breakdown of what it says and what it generally means:  
  
---  
  
### 1. Main Finding: Right Lung Mass  
- **Location:** Lateral segment of the middle lobe of the right lung, extending into the anterior segment of the right upper lobe.  
- **Size:** Approximately 37 mm × 22 mm × 25 mm.  
- **PET Findings:** Strong uptake of FDG with **SUVmax 18.1** (SUVmax measures how “active” the tissue is; higher values often suggest cancer).  
- **CT Characteristics:** Irregular edges, lobulated shape, spiculations (“burrs”), and bronchial truncation — all features that suggest malignancy.  
- **Additional note:** There is a strip of slightly hypermetabolic tissue at the distal end of the lesion connecting to the pleura, SUVmax 2.2, likely representing localized atelectasis (collapsed lung tissue) rather than tumor.  
  
**Interpretation:** This pattern is **highly suggestive of lung cancer**, specifically a right lung carcinoma. The distal area of lower uptake is likely collapsed lung due to obstruction rather than tumor spread.  
  
---  
  
### 2. Other Lung Findings  
- **Multiple small nodules** in both lungs, **non-metabolic** (they do not take up FDG).  
 - Largest is 6 mm × 4 mm in the anterior segment of the upper lobe of the right lung.  
 - **Interpretation:** Likely benign (e.g., scar tissue, inflammation, or very early nodules). Follow-up is recommended.  
  
- **No abnormal lymph nodes** in the mediastinum or elsewhere in the chest.  
 - This is a positive sign because it suggests **no obvious spread to lymph nodes** at this time.  
  
---  
  
### 3. Other Organs  
- Neck, thyroid, liver, pancreas, spleen, kidneys, pelvis: **No abnormal uptake or structural lesions.**  
- Heart and major vessels appear normal.  
- Right breast shows **some calcified foci**, which are usually benign.  
  
---  
  
### 4. Summary from Report  
1. **Hypermetabolic mass in the right middle lung lobe** with features consistent with **right lung carcinoma**.  
2. **Distal lung collapse (atelectasis)** is seen, likely secondary to the mass.  
3. **Multiple small non-metabolic lung nodules** in both lungs, recommended for follow-up.  
4. **No evidence of metastasis** in lymph nodes or other organs on PET/CT.  
  
---  
  
### Key Points  
- The main mass is very suggestive of **malignant lung tumor**.  
- The **SUVmax of 18.1** is relatively high, which supports malignancy.  
- Absence of hypermetabolic lymph nodes is a **good prognostic sign**, though staging requires pathology and sometimes further imaging (e.g., brain MRI, bone scan if indicated).  
- Small nodules in both lungs are **not currently concerning**, but monitoring is recommended.  
  
---  
  
### What Usually Comes Next  
- **Tissue diagnosis (biopsy)** is essential to confirm lung cancer.  
- **Staging work-up** may include:  
 - Chest CT  
 - Brain MRI  
 - Bone scan (if symptoms or suspicion)  
- **Treatment** depends on the stage:  
 - Surgery, chemotherapy, radiation, or a combination.  
- **Follow-up** for the small nodules in both lungs.  
  
---  
  
If you want, I can make a **diagram showing your lung with the tumor, atelectasis, and other nodules**, which often helps patients understand their report visually. This can also help explain why follow-up and staging are important.   
  
Do you want me to do that?

👤
User
2026/3/10 20:47:25

What is the stage of my lung cancer?

🤖
Assistant
2026/3/10 20:47:25

Based on your PET/CT report, we can make an **educated estimate**, but the exact stage requires **pathology and sometimes additional imaging**. Here’s how it looks:   
  
---  
  
### Step 1: Tumor Size and Local Invasion (T)  
- Your main tumor: **37 × 22 × 25 mm (~3.7 cm)** in the **right middle lobe**, extending into the **upper lobe across the horizontal fissure**.  
- **CT features:** Spiculated edges, vascular clusters, and bronchial truncation; some transpleural contact but no clear invasion of chest wall.  
- **Interpretation:** Tumor is >3 cm but ≤5 cm and involves one lobe (with minor extension to adjacent lobe).   
 → Likely **T2b** (tumor >3 cm but ≤5 cm or tumor involving main bronchus >2 cm from carina, without invasion of visceral pleura or chest wall).  
  
---  
  
### Step 2: Lymph Node Involvement (N)  
- **PET/CT:** No abnormal hypermetabolic lymph nodes in the mediastinum or elsewhere.   
- **Interpretation:** Likely **N0** (no regional lymph node metastasis detected).  
  
---  
  
### Step 3: Distant Metastasis (M)  
- **PET/CT:** No hypermetabolic lesions in liver, adrenal glands, bones, brain, or other organs.   
- **Interpretation:** Likely **M0** (no distant metastasis detected).  
  
---  
  
### Probable Stage (Based on AJCC 8th Edition)  
- **T2b N0 M0 → Stage IIA** lung cancer   
 - This is an early-stage lung cancer, **potentially operable** depending on your overall health and lung function.   
  
---  
  
### Important Notes  
1. **PET/CT is excellent but not 100% sensitive** for small lymph node metastases; sometimes a biopsy of mediastinal lymph nodes is needed for confirmation.  
2. **Histopathology (biopsy)** is required to confirm tumor type (e.g., adenocarcinoma, squamous cell carcinoma) and to guide treatment.  
3. If other tests reveal additional nodal involvement, the stage could increase (e.g., Stage III).  
  
---  
  
If you want, I can **draw a simple diagram showing your tumor, lobes involved, and why this corresponds to Stage IIA**, which can make it much easier to visualize.   
  
Do you want me to do that?

🤖
Assistant
2026/3/10 20:47:38

I’ve carefully reviewed your PET/CT report. Here’s a detailed explanation in understandable terms:   
  
---  
  
### 1. **Key Findings from Your PET/CT**  
- **Right middle lobe lung mass**:  
 - There is a **hypermetabolic mass** (meaning it takes up a lot of the radioactive glucose tracer used in PET, which is often seen in cancer cells) in the **lateral segment of the middle lobe of the right lung**.   
 - **Size:** ~37 × 22 × 25 mm.   
 - **SUVmax:** 18.1 (this is high; malignant tumors usually have higher uptake than benign lesions).   
 - The mass has features suggestive of malignancy: **burrs, lobulation, vascular clusters, bronchial truncation**, and it **crosses the fissure** into the upper lobe.   
 - There is **distal lung tissue collapse (atelectasis)** connected to the lesion and some contact with the pleura.   
  
- **Multiple small nodules in both lungs**:  
 - These are **non-metabolic** (don’t take up the tracer), mostly very small (~6 mm or less).   
 - They are likely **benign or indeterminate**. Follow-up imaging is recommended.   
  
- **Mediastinum, lymph nodes, and other organs**:  
 - No abnormal metabolic activity or enlarged lymph nodes were seen.   
 - Heart, liver, spleen, pancreas, kidneys, bladder, and pelvic organs all appear normal.   
 - There is a **small calcified shadow in the right breast**, but it is not metabolically active.   
  
---  
  
### 2. **Interpretation**  
- The **primary concern is the right middle lobe mass**, which has PET/CT features typical of **lung cancer**.   
- The **multiple small nodules** in both lungs are not showing increased metabolism and could be harmless, but should be monitored.   
- No spread to **mediastinal lymph nodes or distant organs** was observed on this scan, which is important for staging.   
  
**Summary of likely diagnosis:**   
> The findings are consistent with **right lung carcinoma with distal atelectasis**.   
  
---  
  
### 3. **Next Steps / Clinical Considerations**  
1. **Pathology confirmation**: A **biopsy** of the lung mass is required to confirm the diagnosis and determine the exact type of lung cancer.   
2. **Staging**: Additional imaging or tests may be done to check for **lymph node involvement or distant metastases**.   
3. **Treatment planning**: Depending on the type and stage, options may include **surgery, radiation, chemotherapy, targeted therapy, or immunotherapy**.   
4. **Follow-up for small nodules**: Regular imaging to ensure these nodules don’t grow or become metabolically active.   
  
---  
  
### 4. **Important Notes**  
- **SUVmax 18.1**: Very suggestive of malignancy.   
- **No evidence of metastasis** seen in this scan, which is a positive sign for treatment planning.   
- The report also notes **type 2 diabetes**, which may affect treatment planning and blood sugar management during therapy.   
  
---  
  
If you want, I can make a **simplified diagram of your lungs showing the tumor, atelectasis, and other nodules**, which often helps patients understand their PET results visually.   
  
Do you want me to do that?

👤
User
2026/3/10 20:47:50

What is the stage of my lung cancer?

🤖
Assistant
2026/3/10 20:47:53

Based on your PET/CT report, I can give a reasoned estimate of the **likely stage** of your lung cancer. Keep in mind that **final staging requires pathology, clinical evaluation, and sometimes additional imaging**.   
  
---  
  
### 1. **Tumor Size and Location (T)**  
- The main tumor is **37 × 22 × 25 mm** in the **right middle lobe**, with **invasion into the anterior segment of the upper lobe**.   
- Features such as **burrs, lobulation, vascular clusters, and bronchial truncation** suggest locally invasive cancer.   
- Since it **crosses the fissure but hasn’t invaded nearby structures like the chest wall, diaphragm, or mediastinum**, it is likely classified as **T2b or T3** depending on exact invasion.  
  
---  
  
### 2. **Lymph Node Involvement (N)**  
- **No abnormal metabolic lymph nodes** in the mediastinum, hilum, or neck.   
- This suggests **N0** (no regional lymph node metastasis).  
  
---  
  
### 3. **Distant Metastasis (M)**  
- **No evidence of spread** to other organs (liver, adrenal glands, bones, brain were not imaged in this scan).   
- This corresponds to **M0**.  
  
---  
  
### 4. **Putting it Together (TNM → Stage)**  
- **T2b–T3, N0, M0** → This generally corresponds to **Stage IIB–IIIA** lung cancer in most TNM staging systems (UICC/AJCC 8th edition).   
  
**Key point:**   
> Your PET/CT shows a **localized, fairly aggressive tumor in the right lung with no lymph node or distant spread visible**. This is **potentially resectable**, but exact staging needs **biopsy and surgical/pathological assessment**.  
  
---  
  
If you want, I can also **explain what this stage means for treatment options and prognosis** in plain language. This helps patients understand next steps and outcomes.   
  
Do you want me to do that?

👤
User
2026/3/10 20:48:14

Can you give me the references?

🤖
Assistant
2026/3/10 20:48:15

Absolutely. Here are reliable references that support the interpretation of your PET/CT report, TNM staging, and lung cancer assessment:   
  
---  
  
### **1. PET/CT in Lung Cancer**  
- **Kandathil AJ, et al.** \*FDG-PET/CT in Lung Cancer: Principles and Clinical Applications.\* Radiographics. 2020;40(7):1983–2000.   
 - Explains SUVmax, hypermetabolic lesions, and differentiating malignant vs benign nodules.   
- **Lardinois D, et al.** \*Staging of non-small-cell lung cancer with integrated PET/CT: a prospective study.\* Lancet. 2003;362:1440–1446.   
 - Demonstrates PET/CT accuracy in detecting primary tumors and mediastinal lymph node involvement.  
  
---  
  
### **2. TNM Staging of Lung Cancer**  
- **Detterbeck FC, et al.** \*The IASLC Lung Cancer Staging Project: Proposals for Revision of the TNM Stage Groupings in the Forthcoming 8th Edition.\* J Thorac Oncol. 2017;12:1109–1120.   
 - Defines tumor size categories (T1–T4), lymph node involvement (N0–N3), and metastasis (M0–M1).   
- **National Comprehensive Cancer Network (NCCN) Guidelines – Non-Small Cell Lung Cancer (NSCLC), Version 4.2025**   
 - Provides staging, imaging recommendations, and clinical decision-making pathways.  
  
---  
  
### **3. SUVmax Interpretation**  
- **Boellaard R, et al.** \*FDG PET and PET/CT: EANM procedure guidelines for tumour PET imaging.\* Eur J Nucl Med Mol Imaging. 2015;42:328–354.   
 - Explains standardized uptake values (SUV), tumor metabolic activity, and clinical significance.   
  
---  
  
### **4. Management of Lung Nodules**  
- **MacMahon H, et al.** \*Guidelines for Management of Pulmonary Nodules.\* Radiology. 2017;284:228–243.   
 - Discusses follow-up strategies for small, non-metabolic lung nodules.  
  
---  
  
✅ **Summary of Evidence-Based Points Relevant to Your Case**  
- Hypermetabolic mass with SUVmax >10 is highly suspicious for malignancy.   
- PET/CT negative lymph nodes suggest N0.   
- Small non-metabolic nodules require follow-up but are likely benign.   
- TNM staging integrates tumor size, invasion, nodes, and metastasis.  
  
---  
  
If you want, I can **make a simplified table mapping your PET/CT findings to TNM stage, SUVmax interpretation, and recommended follow-up** so it’s easier to understand for you and your doctors.   
  
Do you want me to do that?

Exported by ChatGPT Universal Exporter Enhanced v8.3.1
