# Supplementary material for: Preliminary evaluation of DeepSeek-R1 and GPT-5.3 in selected PET/CT clinical scenarios: patient preparation, report interpretation, and diagnostic reasoning
Source: Front Med (Lausanne). 2026 Jun 11;13:1785671. doi: 10.3389/fmed.2026.1785671 (PMC13293837; doi:10.3389/fmed.2026.1785671)
Supplement: Supplementary file 1 [file Data_Sheet_1.zip › Supplemental File 5/Difficult Case 1.html]

Difficult Case 1


# Difficult Case 1

**Conversation ID:** 69b141d5-2d28-8324-904f-d07b5bc9b955

**Model:** auto

**Created:** 2026/3/11 18:20:13

**Last Updated:** 2026/3/11 19:03:58

👤
User
2026/3/11 18:20:14

Can you write an examination opinion based on this PET report and medical history (with differential diagnosis)?

🤖
Assistant
2026/3/11 18:20:34

Based on the information in the PET/CT report and the clinical history you provided, the following is an example of a **formal “Examination Opinion / Impression” section** that could be written for this case, including **differential diagnosis**.  
  
---  
  
## Examination Opinion (Impression)  
  
1. **Diffuse peritoneal and omental abnormalities with ascites**   
 PET/CT demonstrates **irregular thickening of the peritoneum, greater omentum, and mesentery (“omental cake” appearance)** with multiple nodular lesions showing **mild to moderate FDG uptake (SUVmax up to ~6.4)** and **more extensive uptake on Ga-FAPI imaging**.   
 These findings, together with **large-volume ascites and encapsulated peritoneal effusions**, strongly suggest **peritoneal malignant involvement**.  
  
2. **Possible gastrointestinal primary lesion**   
 The **gastric fundus and body show diffuse metabolic elevation (FDG SUVmax ~4.5; FAPI SUVmax ~7.6)** without obvious focal wall thickening on CT.   
 In the context of markedly elevated **CA19-9, CEA, and CA125**, a **gastrointestinal primary tumor (especially gastric carcinoma)** with **peritoneal carcinomatosis** should be considered.  
  
3. **No definite distant parenchymal metastasis detected**   
 - Liver, spleen, pancreas, adrenal glands, and kidneys show **no focal abnormal hypermetabolic lesions**.   
 - No significant **retroperitoneal or mediastinal lymphadenopathy** with abnormal metabolism is identified.   
 - Small pulmonary micronodules (<5 mm) without FDG uptake are **indeterminate but likely benign or inflammatory**.  
  
4. **Other findings**  
 - Diffuse mild FDG uptake in the esophagus without structural abnormality, likely **physiologic or inflammatory**.   
 - Left ischial tuberosity soft-tissue swelling with mild hypermetabolism (SUVmax 3.6), possibly **inflammatory or post-traumatic**.   
 - Healing fractures of the right 4th–5th ribs.  
  
---  
  
# Differential Diagnosis  
  
Based on imaging and clinical findings, the main differential diagnoses include:  
  
### 1. Peritoneal carcinomatosis (most likely)  
Most consistent with:  
- **Gastric carcinoma with peritoneal metastasis**  
- **Pancreatobiliary or gastrointestinal malignancy**  
- Less likely: **colorectal carcinoma**  
  
Supporting features:  
- Omental cake appearance   
- Peritoneal nodules and ascites   
- Elevated tumor markers (CEA, CA125, CA19-9)   
- Gastric metabolic abnormality  
  
---  
  
### 2. Primary peritoneal malignancy  
Possible but less common:  
- **Primary peritoneal carcinoma**  
- **Peritoneal mesothelioma**  
  
These can also present with:  
- Diffuse peritoneal thickening   
- Ascites   
- FAPI-avid lesions  
  
---  
  
### 3. Peritoneal tuberculosis  
Should be considered in endemic regions because it may mimic carcinomatosis with:  
- Peritoneal thickening   
- Ascites   
- Mild to moderate FDG uptake   
  
However:  
- Negative T-cell TB test and tumor marker profile make this **less likely**.  
  
---  
  
### 4. Severe inflammatory peritonitis  
Chronic inflammatory or granulomatous disease could produce similar imaging findings, but **tumor marker elevation and nodular peritoneal pattern favor malignancy**.  
  
---  
  
# Recommendations  
  
1. **Diagnostic confirmation**  
 - **Ultrasound- or CT-guided peritoneal/omental biopsy** (strongly recommended).  
 - **Cytology of ascitic fluid**.  
  
2. **Further evaluation of the stomach**  
 - **Repeat gastroscopy with multiple deep biopsies**, especially in the fundus/body.  
 - Consider **endoscopic ultrasound (EUS)** if initial biopsies are negative.  
  
3. **Tumor marker correlation**  
 - Follow-up measurements of **CA19-9, CEA, and CA125**.  
  
4. **Multidisciplinary evaluation**  
 - Consultation with **gastroenterology, oncology, and surgery** teams.  
  
---  
  
✅ **Summary:**   
PET/CT findings most strongly suggest **peritoneal carcinomatosis with massive ascites, likely originating from a gastrointestinal malignancy (particularly gastric cancer)**, though **primary peritoneal malignancy or tuberculosis peritonitis** remain in the differential. **Histopathologic confirmation is essential.**  
  
---  
  
If you want, I can also help you write a **more formal version suitable for a radiology report**, or a **shorter clinical conclusion that doctors typically include at the end of PET/CT reports.**

Exported by ChatGPT Universal Exporter Enhanced v8.3.1
