# Supplementary material for: Preliminary evaluation of DeepSeek-R1 and GPT-5.3 in selected PET/CT clinical scenarios: patient preparation, report interpretation, and diagnostic reasoning
Source: Front Med (Lausanne). 2026 Jun 11;13:1785671. doi: 10.3389/fmed.2026.1785671 (PMC13293837; doi:10.3389/fmed.2026.1785671)
Supplement: Supplementary file 1 [file Data_Sheet_1.zip › Supplemental File 5/Difficult Case 10.html]

Difficult Case 10


# Difficult Case 10

**Conversation ID:** 69b149e1-9844-8323-9bab-c9986b4b2a97

**Model:** auto

**Created:** 2026/3/11 18:54:35

**Last Updated:** 2026/3/11 19:05:10

👤
User
2026/3/11 18:54:35

Can you write an examination opinion based on this PET report and medical history (with differential diagnosis)?

🤖
Assistant
2026/3/11 18:55:36

**Examination Opinion (based on PET/CT findings and provided medical history)**  
  
**Summary of PET/CT findings**   
Whole-body **18F-FDG PET/CT** demonstrates:  
  
1. **Multiple hypermetabolic lymph nodes in the bilateral cervical regions (Ib, II, V)** with SUVmax approximately **1.2–3.6**, the largest located in the **right cervical level II** (about **24 × 15 mm**) with relatively indistinct margins.   
2. **One larger hypermetabolic lymph node at the left cervical root** adjacent to the inferior portion of the left thyroid lobe (**~25 × 21 × 37 mm**, SUVmax **3.9**) with relatively clear borders and homogeneous density.   
3. **No abnormal FDG uptake in the aorta or major branches**, and no obvious vascular wall thickening on CT within the scanned field.   
4. **No abnormal hypermetabolic lesions in the lungs, mediastinum, abdominal organs, or retroperitoneum.**   
5. **Right adnexal oval hypodense lesion** (~17 × 10 mm) with **mild FDG uptake (SUVmax 2.4)**, likely physiologic or benign.   
6. **Diffuse mild skeletal muscle FDG uptake** without structural abnormality, likely physiologic or related to muscle activity.   
  
---  
  
# Integrated Imaging Impression  
  
### 1. Cervical lymphadenopathy with mild–moderate FDG uptake  
The presence of multiple bilateral cervical lymph nodes with mild–moderate metabolic activity suggests **reactive or inflammatory lymphadenopathy** as the most likely etiology, particularly given:  
  
- Relatively **low SUV values**  
- **Homogeneous density**  
- **Absence of extranodal lesions**  
  
However, the **left cervical root lymph node (SUVmax 3.9, relatively large size)** warrants further clinical correlation and possible follow-up.  
  
**Differential diagnosis includes:**  
  
**Most likely**  
- Reactive inflammatory lymphadenopathy   
- Infection-related lymph node enlargement  
  
**Autoimmune/inflammatory causes**  
- Autoimmune disease–related lymphadenopathy (e.g., systemic autoimmune activation given ANA positivity)  
- Thyroid autoimmune disease–associated reactive lymph nodes  
  
**Less likely but should be considered**  
- Early lymphoproliferative disease (e.g., lymphoma)  
- Metastatic lymphadenopathy (unlikely given low metabolic activity and absence of primary tumor)  
  
If the lymph node persists or enlarges, **ultrasound-guided biopsy may be considered.**  
  
---  
  
### 2. No PET/CT evidence of active large-vessel vasculitis  
Despite clinical suspicion of **aortitis**, the study shows:  
  
- **No abnormal FDG uptake in the aortic wall**  
- **No hypermetabolic signal in major branch vessels**  
  
These findings **do not support active large-vessel inflammatory vasculitis** at the time of imaging.  
  
**Differential considerations for the patient's vascular symptoms:**  
  
- **Subclavian artery stenosis or anatomical variation**  
- **Postoperative vascular changes**  
- **Early or low-grade vasculitis below PET detection threshold**  
  
Clinical correlation with **CTA/MRA or Doppler ultrasound** may be useful.  
  
---  
  
### 3. Right adnexal lesion with mild FDG uptake  
The small oval lesion in the **right adnexal region** with low metabolic activity most likely represents:  
  
**Most likely**  
- Physiologic ovarian follicle or corpus luteum  
- Functional ovarian cyst  
  
**Less likely**  
- Benign ovarian tumor  
  
Follow-up with **pelvic ultrasound** is recommended if clinically indicated.  
  
---  
  
### 4. Thyroid function abnormality  
Laboratory tests indicate **significant hyperthyroidism with elevated TRAb**, compatible with **autoimmune hyperthyroidism (likely Graves’ disease)**. Although no focal thyroid FDG uptake abnormality was observed, autoimmune thyroid disease may be associated with **reactive cervical lymphadenopathy**.  
  
---  
  
# Overall Impression  
1. **Multiple mildly hypermetabolic cervical lymph nodes**, most consistent with **reactive or inflammatory lymphadenopathy**, though the **left cervical root node** should be followed clinically.   
2. **No PET/CT evidence of active large-vessel vasculitis or aortitis.**   
3. **Small right adnexal lesion** with mild uptake, likely **physiologic ovarian activity or benign cystic lesion**.   
4. Findings should be interpreted in conjunction with **clinical history of congenital heart disease surgery, autoimmune markers, and hyperthyroidism**.  
  
---  
  
✅ **Suggested follow-up**  
- Cervical **ultrasound ± biopsy** if lymph node enlargement persists or progresses   
- **CTA/MRA of supra-aortic vessels** if vascular stenosis is suspected   
- **Endocrinology evaluation for hyperthyroidism**   
- **Pelvic ultrasound** for adnexal lesion if symptoms occur or on routine follow-up  
  
---  
  
If you'd like, I can also help you convert this into a **formal radiology “Conclusion/Impression” section suitable for a PET/CT report** (shorter, like what physicians actually write).

Exported by ChatGPT Universal Exporter Enhanced v8.3.1
