# Supplementary material for: Preliminary evaluation of DeepSeek-R1 and GPT-5.3 in selected PET/CT clinical scenarios: patient preparation, report interpretation, and diagnostic reasoning
Source: Front Med (Lausanne). 2026 Jun 11;13:1785671. doi: 10.3389/fmed.2026.1785671 (PMC13293837; doi:10.3389/fmed.2026.1785671)
Supplement: Supplementary file 1 [file Data_Sheet_1.zip › Supplemental File 5/Difficult Case 2.html]

Difficult Case 2


# Difficult Case 2

**Conversation ID:** 69b141f9-cd2c-8324-a87a-c19035dff20c

**Model:** auto

**Created:** 2026/3/11 18:21:45

**Last Updated:** 2026/3/11 19:04:04

👤
User
2026/3/11 18:21:46

Can you write an examination opinion based on this PET report and medical history (with differential diagnosis)?

🤖
Assistant
2026/3/11 18:22:14

Based on the **PET/CT findings and the provided clinical history**, an **examination opinion with differential diagnosis** can be structured as follows.  
  
---  
  
# PET/CT Examination Opinion (with Differential Diagnosis)  
  
## 1. Key PET/CT Findings  
1. **Diffuse skeletal hypermetabolism**  
 - Markedly increased FDG uptake in **cranial bones, axial skeleton, and proximal appendicular bones** (SUVmax ≈ 28.8).  
 - **Multiple osteolytic lesions** in ribs and thoracic vertebrae.  
 - **Soft-tissue mass** adjacent to the right posterior 8th rib (~27 × 20 mm).  
 - Findings indicate **extensive metabolically active bone disease**.  
  
2. **Thoracic findings**  
 - **Hypermetabolic pulmonary nodule** in the apical segment of the right upper lobe   
 - Size ~11 × 9 mm   
 - SUVmax ≈ 4.5   
 - Lobulated margins with spiculation and pleural retraction.  
 - **Markedly hypermetabolic mediastinal lymph node (4R)**   
 - Size ~28 × 21 mm   
 - SUVmax ≈ 23.8.  
  
3. **Thyroid**  
 - **Focal hypermetabolic nodule in left thyroid lobe**  
 - Size ~12 × 11 mm  
 - SUVmax ≈ 8.5.  
  
4. **Liver**  
 - Diffuse increased FDG uptake (SUVmax 15.8)  
 - Reduced CT attenuation (42 HU), compatible with **fatty liver** but metabolic increase requires correlation.  
  
5. **Laboratory abnormalities**  
 - **Hypercalcemia (2.62 mmol/L)**  
 - Elevated **ALP, LDH**  
 - Elevated **GGT**  
 - Systemic bone pain and weakness.  
  
---  
  
# Integrated Imaging Impression  
  
The PET/CT demonstrates **diffuse metabolically active skeletal lesions with osteolytic destruction and associated soft-tissue components**, combined with **hypermetabolic mediastinal lymphadenopathy** and a **suspicious right upper lobe pulmonary nodule**.  
  
These findings strongly suggest a **systemic malignant process involving the skeleton**.  
  
---  
  
# Most Likely Diagnostic Considerations  
  
## 1. Multiple Myeloma / Plasma Cell Myeloma (Highly Considered)  
Supporting features:  
  
- Diffuse skeletal FDG uptake  
- Multiple osteolytic lesions  
- Rib soft-tissue plasmacytoma-like mass  
- Hypercalcemia  
- Elevated ALP/LDH  
- Severe bone pain and weakness  
  
PET findings of **diffuse bone marrow hypermetabolism with focal lytic lesions** are typical for active myeloma.  
  
Recommended confirmation tests:  
  
- Serum/urine protein electrophoresis  
- Serum free light chain assay  
- Bone marrow biopsy  
- Whole-body MRI if needed.  
  
---  
  
## 2. Metastatic Lung Cancer with Diffuse Bone Metastases  
Also plausible because:  
  
- Hypermetabolic **right upper lobe nodule** with malignant morphology  
- **High-SUV mediastinal lymph node**  
- Diffuse bone metastases are common in advanced lung cancer.  
  
However:  
  
- The **extremely diffuse skeletal pattern** is somewhat more typical of **hematologic malignancy** than lung cancer metastasis.  
  
Tissue confirmation from **lung nodule or mediastinal lymph node** may be required.  
  
---  
  
## 3. Lymphoma with Extensive Bone Involvement  
Possible but less typical:  
  
- Lymphoma can produce **diffuse bone marrow uptake and lymphadenopathy**  
- However, **destructive osteolytic lesions and rib soft-tissue masses** are less common than in myeloma.  
  
---  
  
# Additional Incidental or Secondary Considerations  
  
### 1. Thyroid FDG-avid nodule  
SUVmax 8.5 suggests a **significant risk of thyroid malignancy** (e.g., papillary thyroid carcinoma).  
  
Recommendation:  
  
- Thyroid ultrasound  
- Fine-needle aspiration biopsy (FNAB)  
  
---  
  
### 2. Diffuse hepatic uptake  
Most likely related to:  
  
- **Fatty liver**  
- Reactive metabolic change  
  
But clinical correlation with liver function tests is advised.  
  
---  
  
# Suggested Next Diagnostic Steps  
  
1. **Bone marrow biopsy** (priority)  
2. **Serum and urine monoclonal protein tests**  
 - SPEP/UPEP  
 - Immunofixation  
 - Free light chains  
3. **Biopsy of mediastinal lymph node or rib lesion**  
4. **Chest CT follow-up or lung nodule biopsy**  
5. **Thyroid ultrasound and FNA**  
  
---  
  
# Overall Impression  
  
The imaging findings most strongly suggest:  
  
**1️⃣ Multiple myeloma with diffuse skeletal involvement (most likely)**   
**2️⃣ Metastatic lung carcinoma with widespread bone metastases**   
**3️⃣ Hematologic malignancy such as lymphoma**  
  
Histopathological confirmation is essential for definitive diagnosis.  
  
---  
  
If you want, I can also help you write a **more formal radiology-style “PET/CT Conclusion” section (the type used in hospital reports)** or a **clinical diagnostic reasoning note for case discussion**.

Exported by ChatGPT Universal Exporter Enhanced v8.3.1
