# Supplementary material for: Preliminary evaluation of DeepSeek-R1 and GPT-5.3 in selected PET/CT clinical scenarios: patient preparation, report interpretation, and diagnostic reasoning
Source: Front Med (Lausanne). 2026 Jun 11;13:1785671. doi: 10.3389/fmed.2026.1785671 (PMC13293837; doi:10.3389/fmed.2026.1785671)
Supplement: Supplementary file 1 [file Data_Sheet_1.zip › Supplemental File 5/Difficult Case 3.html]

Difficult Case 3


# Difficult Case 3

**Conversation ID:** 69b1425e-0078-8322-89ca-25545d3c889d

**Model:** auto

**Created:** 2026/3/11 18:22:36

**Last Updated:** 2026/3/11 19:04:12

👤
User
2026/3/11 18:22:36

Can you write an examination opinion based on this PET report and medical history (with differential diagnosis)?

🤖
Assistant
2026/3/11 18:23:00

**Examination Opinion Based on PET/CT Findings and Clinical History (with Differential Diagnosis)**   
  
**Patient:** Male, 34 years   
**Clinical indication:** Evaluation of pulmonary lesion and lymphadenopathy.   
**Tumor markers:** NSE 32.8 ng/mL ↑, CYFRA21-1 6.04 ng/mL ↑; CEA, SCC, ProGRP normal.   
**TB immune test:** Negative.   
  
---  
  
# 1. Integrated Imaging Assessment  
  
The PET/CT demonstrates **extensive hypermetabolic lymphadenopathy** involving multiple nodal stations, including:  
  
- Right supraclavicular region   
- Mediastinum   
- Bilateral hilar regions   
- Diaphragmatic pedicle   
- Retroperitoneum   
- Left inguinal region   
  
These nodes show **marked FDG uptake (SUVmax up to ~21.5)** with relatively uniform density and well-defined margins. The largest node measures approximately **25 × 18 mm**.  
  
Additional findings include:  
  
- **Multiple pleural and subpleural hypermetabolic nodules**, particularly on the right side (SUVmax up to ~17.2).  
- **Diffuse splenic involvement**, showing multiple small hypermetabolic nodules (SUVmax ~8.1) without corresponding CT density abnormalities.  
- Small pulmonary nodules in the right lung (≤7 mm) **without obvious FDG uptake**.  
- No significant hypermetabolic lesions in the liver, pancreas, kidneys, adrenal glands, or pelvis.  
  
Overall, the imaging pattern is characterized by:  
  
- **Diffuse nodal involvement above and below the diaphragm**  
- **Splenic metabolic lesions**  
- **Pleural hypermetabolic nodularity**  
- **Absence of a clearly dominant metabolically active lung mass**  
  
---  
  
# 2. Most Likely Diagnosis  
  
### **Malignant Lymphoma (highly suspected)**  
  
The imaging pattern strongly favors lymphoma due to several characteristic features:  
  
1. **Multiple nodal groups involved simultaneously**  
2. **High FDG uptake (SUVmax >20)**  
3. **Uniform nodal morphology**  
4. **Splenic metabolic lesions**  
5. **Distribution both above and below the diaphragm**  
  
These findings are highly typical of **systemic lymphoma**, particularly:  
  
- **Hodgkin lymphoma**  
- **Aggressive non-Hodgkin lymphoma (e.g., diffuse large B-cell lymphoma)**  
  
The patient's **young age (34 years)** also supports lymphoma as a leading diagnosis.  
  
---  
  
# 3. Alternative Diagnoses (Differential Diagnosis)  
  
## 1. Metastatic Lung Cancer with Extensive Nodal Metastasis  
Possible but **less likely** because:  
  
- No clearly hypermetabolic primary lung mass is identified.  
- Lung nodules are small and mostly **non-FDG avid**.  
- Splenic nodular uptake is less typical for lung cancer metastases.  
  
However, the elevated **CYFRA21-1** and **NSE** may raise concern for lung malignancy (e.g., squamous cell carcinoma or neuroendocrine tumor).  
  
---  
  
## 2. Sarcoidosis or Sarcoid-like Granulomatous Disease  
Could present with:  
  
- Bilateral hilar and mediastinal lymphadenopathy  
- FDG uptake in lymph nodes and spleen  
  
However:  
  
- **SUV values are unusually high** for sarcoidosis  
- Retroperitoneal and inguinal node involvement is less typical  
- Pleural nodules are uncommon in sarcoidosis  
  
Therefore this is **possible but less favored**.  
  
---  
  
## 3. Disseminated Infection (e.g., Tuberculosis or Fungal Disease)  
  
Disseminated infection can produce hypermetabolic lymph nodes and splenic lesions.  
  
Arguments against this diagnosis:  
  
- TB immunologic test negative   
- No systemic inflammatory laboratory abnormalities   
- Very high nodal SUV values more consistent with malignancy  
  
Thus infection is **unlikely but cannot be fully excluded** without tissue diagnosis.  
  
---  
  
# 4. Suggested Next Diagnostic Step  
  
Because PET/CT cannot provide a definitive pathological diagnosis, **histological confirmation is essential**.  
  
Recommended biopsy targets (highest diagnostic yield):  
  
1. **Right supraclavicular lymph node biopsy** (easiest and least invasive)  
2. Mediastinal lymph node biopsy (EBUS-TBNA)  
3. Pleural lesion biopsy if nodal biopsy is inconclusive  
  
Pathology should include:  
  
- Histology  
- Immunohistochemistry  
- Flow cytometry  
- Molecular testing if lymphoma suspected  
  
---  
  
# 5. Provisional Imaging Impression  
  
**PET/CT findings suggest a systemic lymphoproliferative disease, most consistent with malignant lymphoma, involving multiple lymph node stations, spleen, and pleura.**   
  
Metastatic malignancy (including lung cancer) and granulomatous diseases such as sarcoidosis or infection remain **secondary differential considerations**, and **pathological biopsy is required for definitive diagnosis**.  
  
---  
  
✅ **Summary:**   
The PET/CT pattern is **most compatible with lymphoma rather than primary lung cancer**, despite the clinical suspicion of lung malignancy.  
  
---  
  
If you want, I can also help you with:  
  
- **What stage this disease would be if it is lymphoma**   
- **Which biopsy site is best according to PET findings**   
- **What the survival rates and treatments might look like** based on each possible diagnosis.

Exported by ChatGPT Universal Exporter Enhanced v8.3.1
