# Supplementary material for: Preliminary evaluation of DeepSeek-R1 and GPT-5.3 in selected PET/CT clinical scenarios: patient preparation, report interpretation, and diagnostic reasoning
Source: Front Med (Lausanne). 2026 Jun 11;13:1785671. doi: 10.3389/fmed.2026.1785671 (PMC13293837; doi:10.3389/fmed.2026.1785671)
Supplement: Supplementary file 1 [file Data_Sheet_1.zip › Supplemental File 5/Difficult Case 4.html]

Difficult Case 4


# Difficult Case 4

**Conversation ID:** 69b14272-9f34-8322-a11f-c941df487750

**Model:** auto

**Created:** 2026/3/11 18:22:48

**Last Updated:** 2026/3/11 19:04:23

👤
User
2026/3/11 18:22:48

Can you write an examination opinion based on this PET report and medical history (with differential diagnosis)?

🤖
Assistant
2026/3/11 18:23:07

Based on the PET/CT findings and the clinical history provided in the report, the following **examination opinion (impression) with differential diagnosis** can be formulated in a format commonly used in nuclear medicine reports.  
  
---  
  
# Examination Opinion (PET/CT Impression)  
  
### 1. Hypermetabolic lesion in the pancreatic head–neck region  
PET/CT demonstrates **focal nodular FDG uptake in the pancreatic head–neck region** (SUVmax ≈3.7 early, 3.9 delayed; size ≈24 × 17 mm) with **ill-defined margins, associated pancreatic duct dilatation, and distal pancreatic atrophy**, with **unclear fat planes around the pancreas** and possible proximity to the **portal vein**.  
  
Combined with:  
  
- Progressive obstructive jaundice history   
- Elevated **CA19-9 (587 U/mL)**   
- Prior CT/MR findings suggesting pancreatic head mass   
- EUS/biopsy showing atypical ductal epithelial proliferation   
  
These findings are **highly suspicious for pancreatic ductal adenocarcinoma (PDAC)**.  
  
However, the **FDG uptake is only mildly increased**, therefore histologic correlation remains necessary if clinically indicated.  
  
---  
  
### 2. Possible regional nodal involvement  
An **elliptical soft-tissue nodule in the hepatogastric space** (≈19 × 13 mm, SUVmax ≈2.0) demonstrates **mild FDG uptake**, which may represent:  
  
- **Metastatic lymph node** (most likely given pancreatic malignancy context)   
- Reactive inflammatory lymph node  
  
Correlation with contrast-enhanced CT, MRI, or follow-up imaging is recommended.  
  
---  
  
### 3. Right paravertebral soft-tissue nodule (T5 level)  
A **round soft-tissue density lesion adjacent to the right side of T5 vertebra** (≈21 × 18 mm, SUVmax ≈1.7) with **low metabolic activity**.  
  
Possible considerations include:  
  
- Neurogenic tumor (e.g., schwannoma or neurofibroma)   
- Benign paravertebral soft tissue lesion   
- Less likely metastatic disease due to low FDG avidity  
  
Further characterization with **MRI of the thoracic spine** may be helpful.  
  
---  
  
### 4. Hepatic segment S8 low-density lesion without FDG uptake  
A **18 × 15 mm hypodense lesion in segment S8** shows **no abnormal FDG metabolism**.  
  
Most likely:  
  
- Hepatic cyst   
- Hemangioma   
- Focal benign lesion  
  
No clear evidence of hepatic metastasis on current PET/CT.  
  
---  
  
### 5. Additional incidental findings  
- Multiple **small pulmonary nodules (<5 mm)** without FDG uptake — likely benign.   
- **Right middle lobe pulmonary cystic change** without metabolic activity.   
- **Accessory splenic tissue (possible splenule)** medial to the spleen.   
- **Coronary artery calcifications** suggesting atherosclerosis.   
- Status post **biliary stent placement** with pneumobilia and biliary dilatation.  
  
---  
  
# Overall Imaging Assessment  
Findings are **most consistent with pancreatic head malignancy**, likely **pancreatic ductal adenocarcinoma**, with **possible regional lymph node involvement**, and **no definite distant metastasis on PET/CT**.  
  
Clinical staging correlation is recommended.  
  
---  
  
# Differential Diagnosis  
Although pancreatic cancer is most likely, alternative possibilities include:  
  
1. **Pancreatic ductal adenocarcinoma** (most likely)   
2. **Mass-forming chronic pancreatitis / inflammatory pseudotumor**   
3. **Autoimmune pancreatitis (focal type)**   
4. **Pancreatic neuroendocrine tumor** (less likely due to imaging pattern)   
5. **Pancreatic metastasis from another primary** (rare)  
  
---  
  
# Suggested Next Steps  
- Correlate with **EUS-guided biopsy pathology** (repeat if necessary).   
- **Contrast-enhanced pancreatic protocol CT or MRI** for surgical staging.   
- Multidisciplinary discussion (surgery / oncology / gastroenterology).   
- Monitor **CA19-9 trend**.  
  
---  
  
If you'd like, I can also help you convert this into:  
  
- **A formal radiology report format used in hospitals**, or   
- **A staging assessment (TNM stage estimate)** based on this PET/CT.

Exported by ChatGPT Universal Exporter Enhanced v8.3.1
