# Supplementary material for: Preliminary evaluation of DeepSeek-R1 and GPT-5.3 in selected PET/CT clinical scenarios: patient preparation, report interpretation, and diagnostic reasoning
Source: Front Med (Lausanne). 2026 Jun 11;13:1785671. doi: 10.3389/fmed.2026.1785671 (PMC13293837; doi:10.3389/fmed.2026.1785671)
Supplement: Supplementary file 1 [file Data_Sheet_1.zip › Supplemental File 5/Difficult Case 5.html]

Difficult Case 5


# Difficult Case 5

**Conversation ID:** 69b14283-42f0-8321-952d-633cac57db41

**Model:** auto

**Created:** 2026/3/11 18:23:04

**Last Updated:** 2026/3/11 19:04:29

👤
User
2026/3/11 18:23:05

Can you write an examination opinion based on this PET report and medical history (with differential diagnosis)?

🤖
Assistant
2026/3/11 18:23:23

Based on the PET/CT report and the provided clinical history, the following **examination opinion (impression) with differential diagnosis** can be formulated in a style similar to clinical nuclear medicine reporting.  
  
---  
  
# Examination Opinion (Impression)  
  
1. **Right upper lobe pulmonary nodule**  
 - An **irregular soft-tissue nodule in the anterior segment of the right upper lobe** measuring approximately **15 × 11 × 11 mm** demonstrates **mild–moderate FDG uptake (SUVmax 4.0 early, 5.6 delayed)** with **spiculated (burr) margins**.  
 - These imaging features are **suspicious for a primary lung malignancy**, particularly **early-stage non–small cell lung cancer (NSCLC)**.  
  
2. **Mediastinal and hilar lymph nodes**  
 - Multiple **FDG-avid mediastinal and bilateral hilar lymph nodes** (stations **4R/L, 5, 7, 8**) with **SUVmax up to 8.8**, the largest measuring about **12 mm**.  
 - Some nodes show **calcification and indistinct margins**, which raises the possibility of **granulomatous inflammatory disease** rather than definite metastatic disease.  
  
3. **Small pulmonary nodules**  
 - Several **tiny nodules (<5 mm)** in both lungs without abnormal FDG uptake.  
 - These are **more likely benign**, such as **post-inflammatory or fibrotic nodules**, but **follow-up CT** is recommended.  
  
4. **Adrenal glands**  
 - **Mildly increased metabolism in bilateral adrenal glands** (SUVmax 3.7 right, 2.8 left) without clear mass lesion.  
 - Findings may represent **physiological uptake or mild adrenal hyperplasia** rather than metastasis; correlation with follow-up imaging or clinical findings is suggested.  
  
5. **Liver lesions**  
 - Multiple **small hypodense lesions without FDG uptake**, most consistent with **hepatic cysts**.  
  
6. **No definite distant metastatic disease**  
 - No abnormal FDG-avid lesions identified in **brain, liver, bones, or abdominal organs** to suggest clear distant metastasis.  
  
---  
  
# Differential Diagnosis  
  
### For the right upper lobe pulmonary nodule  
  
1. **Primary lung cancer (most likely)**  
 - Early **non-small cell lung cancer**, especially **adenocarcinoma**.  
  
2. **Granulomatous inflammatory lesion**  
 - **Tuberculosis**  
 - **Sarcoidosis**  
 - **Fungal infection**  
 - Supported by **hypermetabolic mediastinal lymph nodes with calcifications**.  
  
3. **Inflammatory pseudotumor or organizing pneumonia**  
  
4. **Benign pulmonary tumor**  
 - Less likely given **spiculated margins and FDG uptake**.  
  
---  
  
# Overall Imaging Assessment  
  
- **Suspicious pulmonary malignancy in the right upper lobe** with **FDG-avid mediastinal lymph nodes**.  
- However, **granulomatous or inflammatory lymphadenopathy remains an important differential**, particularly given the **calcified lymph nodes**.  
  
---  
  
# Recommended Next Steps  
  
1. **Histopathologic confirmation**  
 - Bronchoscopy with biopsy or  
 - CT-guided percutaneous biopsy.  
  
2. **Mediastinal lymph node evaluation**  
 - **EBUS-TBNA (endobronchial ultrasound-guided biopsy)** to distinguish:  
 - metastatic nodes   
 - granulomatous disease.  
  
3. **Short-term imaging follow-up** if pathology is inconclusive.  
  
---  
  
If you want, I can also help you with:  
  
- **Possible TNM staging based on this PET/CT**  
- **Whether the lymph nodes are more likely inflammatory or metastatic**  
- **Expected treatment options if this is lung cancer**.

Exported by ChatGPT Universal Exporter Enhanced v8.3.1
