# Supplementary material for: Preliminary evaluation of DeepSeek-R1 and GPT-5.3 in selected PET/CT clinical scenarios: patient preparation, report interpretation, and diagnostic reasoning
Source: Front Med (Lausanne). 2026 Jun 11;13:1785671. doi: 10.3389/fmed.2026.1785671 (PMC13293837; doi:10.3389/fmed.2026.1785671)
Supplement: Supplementary file 1 [file Data_Sheet_1.zip › Supplemental File 5/Difficult Case 6.html]

Difficult Case 6


# Difficult Case 6

**Conversation ID:** 69b149af-dbf4-8324-8588-14e6d3072f73

**Model:** auto

**Created:** 2026/3/11 18:53:40

**Last Updated:** 2026/3/11 19:04:43

👤
User
2026/3/11 18:53:41

Can you write an examination opinion based on this PET report and medical history (with differential diagnosis)?

🤖
Assistant
2026/3/11 18:54:17

**Examination Opinion (based on the provided PET/CT report and clinical history)**  
  
**Summary of Key PET/CT Findings**  
- A solitary **hypermetabolic splenic mass** located within the splenic parenchyma measuring approximately **58 × 48 × 45 mm**, with **SUVmax ≈ 8.7**. The lesion shows **peripheral FDG uptake with a central hypometabolic area**, suggesting possible necrosis. The margin appears relatively clear.  
- **Multiple hypermetabolic bone lesions** involving **T5, T7, L2, L4–L5 vertebrae and the right ischium**, with the most prominent uptake in **T5 and the right ischium (SUVmax ≈ 8.7)**. CT shows **increased bone density without obvious destructive changes**.  
- **Mildly hypermetabolic bilateral hilar lymph nodes** (SUVmax ~3.8–4.5) without significant enlargement.  
- **No abnormal hypermetabolic lesions** in the liver, pancreas, adrenal glands, kidneys, mediastinum, pelvis, or brain.  
- Incidental findings include **multiple hepatic cysts**, **pulmonary cystic changes**, mild **subpleural ground-glass opacity in the right lung**, and **coronary artery calcifications**.  
  
---  
  
# Examination Opinion  
  
PET/CT demonstrates a **hypermetabolic splenic mass with central necrosis accompanied by multiple hypermetabolic skeletal lesions**, suggesting a **systemic malignant process involving the spleen and bone**. No clear primary extra-splenic tumor is identified on this examination.  
  
Given the clinical history of **recently detected splenic occupying lesion** and hematologic evaluation showing **no monoclonal B-cell population on flow cytometry**, the imaging findings most strongly suggest a **hematologic malignancy or splenic primary tumor with secondary skeletal involvement**.  
  
---  
  
# Most Likely Diagnosis  
### 1. **Primary splenic lymphoma with bone involvement (most likely)**  
Supporting features:  
- Splenic mass with **intense FDG uptake**  
- **Multiple FDG-avid bone lesions**  
- Absence of a clear extra-splenic primary tumor  
- Clinical suspicion of lymphoma on MRI  
  
Primary splenic lymphoma or secondary involvement by **diffuse large B-cell lymphoma (DLBCL)** is the leading consideration.  
  
---  
  
# Differential Diagnosis  
  
### 2. **Metastatic malignancy with splenic metastasis**  
Less common but possible, especially with bone involvement. Potential primary sites include:  
- Lung carcinoma   
- Gastrointestinal malignancies   
- Melanoma   
  
However, **no primary hypermetabolic lesion was identified**, making this less likely.  
  
---  
  
### 3. **Primary splenic angiosarcoma or other primary splenic sarcoma**  
Consider if pathology excludes lymphoma. Features supporting this possibility include:  
- Large splenic mass  
- Necrotic center  
- Potential hematogenous skeletal spread  
  
However, this is **rare** and usually more aggressive with hemorrhagic features.  
  
---  
  
### 4. **Infectious or granulomatous disease**  
Examples:  
- Splenic abscess  
- Tuberculosis  
- Fungal infection  
  
These conditions can be FDG-avid, but **multifocal bone involvement with this pattern and SUV level favors malignancy**.  
  
---  
  
# Recommendations  
1. **Histopathological confirmation is essential**  
 - Image-guided **splenic biopsy** or **bone lesion biopsy**.  
2. **Further hematologic evaluation**  
 - Bone marrow biopsy  
 - Expanded immunophenotyping and molecular testing.  
3. **Correlation with laboratory data**  
 - LDH  
 - β2-microglobulin  
 - Complete blood count and peripheral smear.  
  
---  
  
✅ **Overall Impression:**   
Findings are **most suspicious for lymphoma involving the spleen with multifocal skeletal involvement**, though **metastatic disease or rare primary splenic malignancy cannot be excluded without tissue diagnosis**.  
  
---  
  
If you want, I can also help you write a **more formal radiology-style “Impression” section (the concise version used in hospital PET/CT reports)** or a **multidisciplinary tumor board interpretation**.

Exported by ChatGPT Universal Exporter Enhanced v8.3.1
