# Supplementary material for: Preliminary evaluation of DeepSeek-R1 and GPT-5.3 in selected PET/CT clinical scenarios: patient preparation, report interpretation, and diagnostic reasoning
Source: Front Med (Lausanne). 2026 Jun 11;13:1785671. doi: 10.3389/fmed.2026.1785671 (PMC13293837; doi:10.3389/fmed.2026.1785671)
Supplement: Supplementary file 1 [file Data_Sheet_1.zip › Supplemental File 5/Difficult Case 7.html]

Difficult Case 7


# Difficult Case 7

**Conversation ID:** 69b142ba-b754-8324-bee4-5834a55822a5

**Model:** auto

**Created:** 2026/3/11 18:23:59

**Last Updated:** 2026/3/11 19:04:50

👤
User
2026/3/11 18:23:49

Can you write an examination opinion based on this PET report and medical history (with differential diagnosis)?

🤖
Assistant
2026/3/11 18:24:46

**Examination Opinion (based on PET/CT findings and clinical history)**   
  
The whole-body **18F-FDG PET/CT** demonstrates **multiple hypermetabolic lymph nodes** involving the pharyngeal lymphatic ring, bilateral cervical regions (II–V), supraclavicular areas, axillae, mediastinum, hilar regions, abdominal para-aortic and mesenteric regions, iliac vessels, and bilateral inguinal regions. The lesions show **SUVmax ranging from 2.0 to 17.4**, and the largest node is located in the **left cervical region II (approximately 33 × 26 mm)** with relatively uniform density. In addition, the **spleen is enlarged with diffusely increased FDG uptake (SUVmax ≈ 4.9)**.   
  
Diffuse increased metabolic activity is also seen in the **bone marrow of the proximal humeri, proximal femora, vertebrae, and pelvis** (SUVmax ≈ 4.2) without definite structural bone destruction on CT. Other organs—including brain, lungs, liver, pancreas, kidneys, and pelvic organs—do not show focal abnormal hypermetabolic lesions suggestive of a primary solid malignancy.   
  
Combined with the **clinical presentation of fever, sore throat, leukocytosis with marked lymphocytosis, and generalized lymphadenopathy**, as well as **bone marrow findings showing increased T-lymphocytes (CD3+) suggesting a T-lymphoid proliferative tendency**, the PET/CT findings mainly indicate a **systemic lymphoproliferative process**.   
  
### Most likely consideration  
1. **Malignant lymphoma (especially T-cell lymphoma)**   
 - Extensive systemic lymphadenopathy with high metabolic activity.   
 - Associated splenomegaly and increased bone marrow metabolism.   
 - Bone marrow immunohistochemistry showing increased **CD3-positive T lymphocytes**, supporting a T-cell lymphoproliferative disorder.   
  
### Differential diagnosis  
1. **Reactive lymphoid hyperplasia secondary to infection**   
 - Such as **infectious mononucleosis (e.g., Epstein–Barr virus infection)** or severe systemic infection.   
 - These conditions can present with diffuse lymphadenopathy, splenic enlargement, and bone marrow activation.  
  
2. **Other lymphoproliferative disorders**   
 - Including **T-cell lymphoproliferative disease or early lymphoma** not yet fully characterized by pathology.  
  
3. **Systemic inflammatory or infectious disease**   
 - Severe bacterial or viral infection with reactive lymph node and bone marrow activation.  
  
### Recommendation  
- **Definitive diagnosis requires histopathological confirmation**, preferably via **excisional biopsy of the most metabolically active lymph node** (e.g., left cervical node).   
- Correlate with **molecular pathology, flow cytometry, EBV serology/viral load**, and **hematologic evaluation**.   
- Continued hematology consultation is recommended to distinguish **malignant lymphoma from reactive or infectious lymphoproliferative conditions**.   
  
**Overall impression:**   
PET/CT findings suggest **systemic lymphoproliferative disease**, with **malignant lymphoma (particularly T-cell lymphoma) strongly suspected**, although **infectious or reactive lymphoid hyperplasia** remains an important differential diagnosis pending pathological confirmation.

Exported by ChatGPT Universal Exporter Enhanced v8.3.1
