# Supplementary material for: Preliminary evaluation of DeepSeek-R1 and GPT-5.3 in selected PET/CT clinical scenarios: patient preparation, report interpretation, and diagnostic reasoning
Source: Front Med (Lausanne). 2026 Jun 11;13:1785671. doi: 10.3389/fmed.2026.1785671 (PMC13293837; doi:10.3389/fmed.2026.1785671)
Supplement: Supplementary file 1 [file Data_Sheet_1.zip › Supplemental File 5/Difficult Case 8.html]

Difficult Case 8


# Difficult Case 8

**Conversation ID:** 69b142cb-ac60-8321-8f03-e8dedc89f3be

**Model:** auto

**Created:** 2026/3/11 18:24:17

**Last Updated:** 2026/3/11 19:04:58

👤
User
2026/3/11 18:24:18

Can you write an examination opinion based on this PET report and medical history (with differential diagnosis)?

🤖
Assistant
2026/3/11 18:24:39

### Examination Opinion (based on PET/CT findings and clinical history)  
  
**Clinical background:**   
- History of **entity["disease","Malignant Melanoma"]** of the left parotid gland, status post surgical resection 2 years ago.   
- Current presentation: **fever of unknown origin for >20 days**.   
- Whole-body **entity["medical\_imaging","18F-FDG PET/CT"]** performed for evaluation.  
  
---  
  
## 1. Main PET/CT Findings  
1. **Extensive hypermetabolic lymphadenopathy**   
 - Multiple lymph nodes with markedly increased FDG uptake involving:  
 - Left supraclavicular and infraclavicular regions   
 - Left axilla   
 - Mediastinum and bilateral hilar regions   
 - Hepatic hilar region and hepatogastric space   
 - Retroperitoneum (upper and lower abdomen)   
 - Bilateral iliac vascular regions   
 - Bilateral inguinal regions   
 - The largest lymph node in the **left inguinal region** measures approximately **20 × 16 mm**, **SUVmax ≈ 18.5**.  
  
2. **Splenomegaly with increased metabolic activity**   
 - Enlarged spleen (~7 rib units) with **SUVmax ≈ 7.9**, suggesting diffuse splenic involvement.  
  
3. **Bone marrow metabolic activation**   
 - Diffuse, mildly increased FDG uptake in the bone marrow of the spine, pelvis, and proximal long bones (**SUVmax 3.0–3.4**), **without osteolytic or osteoblastic destruction**.  
  
4. **No focal hypermetabolic lesions** in the brain, lungs, liver, pancreas, kidneys, adrenal glands, gastrointestinal tract, or pelvic organs.  
  
---  
  
# Imaging Interpretation  
  
The PET/CT demonstrates **systemic lymph node involvement with splenic enlargement and diffuse bone marrow metabolic activation**, suggesting a **systemic lymphoproliferative or metastatic process**.  
  
---  
  
# Most Likely Diagnosis  
### 1. **entity["disease","Lymphoma"] (highly suspected)**  
Features supporting lymphoma:  
  
- Widespread lymph node involvement across **multiple nodal stations** above and below the diaphragm   
- **Very high FDG uptake (SUVmax 18.5)**   
- **Splenic involvement**   
- **Diffuse bone marrow activation without destructive lesions**   
- Associated **fever (B symptoms)**  
  
This pattern is highly typical of **systemic lymphoma**, especially:  
  
- **entity["disease","Diffuse Large B-Cell Lymphoma"]**  
- **entity["disease","Hodgkin Lymphoma"]**  
  
---  
  
# Differential Diagnosis  
  
### 2. **Metastatic recurrence of entity["disease","Malignant Melanoma"]**  
Consider because:  
  
- History of parotid melanoma surgery  
- Melanoma can metastasize to lymph nodes and spleen  
  
However, several features are **less typical**:  
  
- No dominant primary recurrence  
- Diffuse symmetrical nodal distribution typical of lymphoma  
- Absence of visceral metastases (lung, liver, brain)  
  
Thus melanoma metastasis is **possible but less likely**.  
  
---  
  
### 3. **Systemic infection or inflammatory disease**  
Examples include:  
  
- **entity["disease","Tuberculosis"]**  
- **entity["disease","Sarcoidosis"]**  
- Severe systemic infection causing **reactive lymphadenopathy**  
  
However:  
  
- SUVmax values are **very high**  
- Node distribution and splenic uptake favor malignancy.  
  
---  
  
# Suggested Next Diagnostic Steps  
  
1. **Lymph node biopsy (strongly recommended)**  
 - Preferably **inguinal node** (largest and most accessible).  
  
2. **Histopathology + immunohistochemistry**  
 - To differentiate lymphoma vs metastatic melanoma.  
  
3. Additional tests:  
 - Peripheral blood smear  
 - Bone marrow biopsy (if lymphoma suspected)  
 - LDH, β2-microglobulin  
  
---  
  
# Summary Impression  
  
PET/CT demonstrates **extensive hypermetabolic lymphadenopathy with splenic involvement and diffuse bone marrow activation**, highly suggestive of **systemic lymphoma**.   
  
Given the patient’s history, **metastatic malignant melanoma** remains a secondary consideration. **Histopathological confirmation via lymph node biopsy is essential.**  
  
---  
  
✅ **In one sentence:**   
The imaging pattern most strongly suggests **systemic lymphoma**, but **metastatic melanoma recurrence** must be excluded by biopsy.  
  
---  
  
If you'd like, I can also help you write a **formal radiology “Impression/Conclusion” section exactly in the style used in PET/CT reports** (the format nuclear medicine physicians typically use).

Exported by ChatGPT Universal Exporter Enhanced v8.3.1
