# Supplementary material for: Preliminary evaluation of DeepSeek-R1 and GPT-5.3 in selected PET/CT clinical scenarios: patient preparation, report interpretation, and diagnostic reasoning
Source: Front Med (Lausanne). 2026 Jun 11;13:1785671. doi: 10.3389/fmed.2026.1785671 (PMC13293837; doi:10.3389/fmed.2026.1785671)
Supplement: Supplementary file 1 [file Data_Sheet_1.zip › Supplemental File 5/Difficult Case 9.html]

Difficult Case 9


# Difficult Case 9

**Conversation ID:** 69b142e2-853c-8323-b9dd-c11409731cdd

**Model:** auto

**Created:** 2026/3/11 18:24:40

**Last Updated:** 2026/3/11 19:05:05

👤
User
2026/3/11 18:24:41

Can you write an examination opinion based on this PET report and medical history (with differential diagnosis)?

🤖
Assistant
2026/3/11 18:25:31

**Examination Opinion (based on the provided PET/CT report and clinical history)**   
  
**Clinical background:**   
A 22-year-old male presented with **fever of unknown origin for ~3 weeks**, intermittent temperatures up to 38–38.5 °C, mild cough, and **persistent leukopenia** (WBC ~2.6–3.18 × 10⁹/L). Infectious screening including respiratory pathogens, EBV, tuberculosis T-SPOT, Borrelia, and blood cultures were negative. Bone marrow biopsy demonstrated **myeloproliferative hypoplasia without definite malignant infiltration**.   
  
**PET/CT findings:**   
Whole-body ^18F-FDG PET/CT demonstrates:   
- **Multiple hypermetabolic lymph nodes** involving:   
 - Bilateral cervical levels I–III   
 - Bilateral supraclavicular regions   
 - Bilateral axillary regions   
 - Mediastinal (stations II and IV) and hilar regions   
 - Retroperitoneal and mesenteric lymph nodes   
 - Nodes along bilateral iliac vessels   
- **SUVmax range: ~1.8–21.7**, with several nodes mildly enlarged (e.g., right supraclavicular ~22 × 9 mm; mesenteric ~21 × 7 mm).   
- Nodal morphology generally **well-defined with relatively homogeneous density**.   
- **No abnormal FDG uptake in solid organs** including lung parenchyma, liver, spleen, bone, or brain.   
- **Spleen size and metabolism appear normal.**   
- No focal extranodal hypermetabolic lesion identified.  
  
---  
  
# Imaging Impression  
The PET/CT demonstrates **systemic hypermetabolic lymphadenopathy involving multiple nodal regions above and below the diaphragm** without obvious extranodal disease. The metabolic activity is markedly elevated in some nodes (SUVmax up to ~21.7), which raises strong concern for a **lymphoproliferative disorder**, although inflammatory or immune-mediated etiologies remain possible given the clinical context of fever and leukopenia.  
  
---  
  
# Differential Diagnosis  
  
### 1. Malignant lymphoma (most likely)  
The imaging pattern is highly suspicious for lymphoma due to:   
- **Multistation lymph node involvement** (cervical, mediastinal, axillary, abdominal).   
- **High FDG uptake (SUV >20)** in some nodes.   
- Relatively preserved nodal morphology without necrosis.   
  
Possible subtypes include:   
- **Hodgkin lymphoma**, particularly nodular sclerosis or mixed cellularity type.   
- **Aggressive non-Hodgkin lymphoma** (e.g., diffuse large B-cell lymphoma).   
  
However, the **normal spleen metabolism and absence of extranodal lesions** make early-stage or nodal-dominant disease possible.  
  
---  
  
### 2. Systemic inflammatory or immune-mediated lymphadenitis  
Some inflammatory conditions can produce **diffuse hypermetabolic lymphadenopathy**:  
  
- **Kikuchi-Fujimoto disease (histiocytic necrotizing lymphadenitis)**   
 - Often occurs in young adults with fever and leukopenia.   
 - PET uptake may be intense and mimic lymphoma.   
  
- **Systemic autoimmune disease** (e.g., early systemic lupus erythematosus)   
 - ANA positivity (1:100) may suggest an autoimmune background.   
  
---  
  
### 3. Infectious lymphadenopathy  
Despite negative testing, certain infections can produce similar findings:  
  
- **Viral infection** (EBV/CMV or other viral lymphadenitis)   
- **Tuberculosis or atypical mycobacterial infection**   
- **Other systemic infections**   
  
However, the **very high SUV values and widespread nodal distribution** make infection somewhat less likely.  
  
---  
  
### 4. Hematologic or immune disorders  
Less likely possibilities include:   
- **Hemophagocytic lymphohistiocytosis (HLH)**   
- **Early hematologic malignancy without marrow infiltration**  
  
---  
  
# Overall Assessment  
The PET/CT findings show **extensive metabolically active lymphadenopathy highly suspicious for a lymphoproliferative disorder**, particularly **lymphoma**, in the setting of fever of unknown origin and leukopenia. However, **inflammatory lymphadenitis (especially Kikuchi disease) and systemic infection remain important differential diagnoses** in a young patient.  
  
---  
  
# Recommended Next Steps  
1. **Excisional biopsy of the most metabolically active and accessible lymph node**   
 - Preferably **supraclavicular or cervical node** identified on PET/CT.   
2. Comprehensive **histopathology with immunohistochemistry and flow cytometry**.   
3. Consider additional laboratory evaluation:   
 - LDH, ferritin, soluble IL-2 receptor   
 - Viral serology (EBV, CMV)   
 - Autoimmune markers if clinically indicated.   
4. Correlate with bone marrow findings and clinical course.  
  
---  
  
✅ **Key point:**   
PET/CT demonstrates systemic hypermetabolic lymphadenopathy most suggestive of **lymphoma**, but **definitive diagnosis requires lymph node biopsy** because inflammatory diseases in young patients can closely mimic malignant lymphoma on PET imaging.  
  
---  
  
If you want, I can also help you:   
- Turn this into a **formal radiology “Impression” section** suitable for a PET/CT report.   
- Estimate the **most likely lymphoma stage if the biopsy confirms lymphoma**.   
- Explain **which lymph node should be biopsied based on PET findings.**

Exported by ChatGPT Universal Exporter Enhanced v8.3.1
