# Supplementary material for: Preliminary evaluation of DeepSeek-R1 and GPT-5.3 in selected PET/CT clinical scenarios: patient preparation, report interpretation, and diagnostic reasoning
Source: Front Med (Lausanne). 2026 Jun 11;13:1785671. doi: 10.3389/fmed.2026.1785671 (PMC13293837; doi:10.3389/fmed.2026.1785671)
Supplement: Supplementary file 1 [file Data_Sheet_1.zip › Supplemental File 3.docx]

Supplemental File 3

Content (each entry is a hyperlink)

[R1: Provide primary and differential diagnosis based on the report with clinical history.?------------------------------------------------3](#R1)

[R2: Provide primary and differential diagnosis based on the report with clinical history.------------------------------------------------10](#R2)

[R3: Provide primary and differential diagnosis based on the report with clinical history.------------------------------------------------18](#R3)

[R4: Provide primary and differential diagnosis based on the report with clinical history.------------------------------------------------25](#R4)

[R5: Provide primary and differential diagnosis based on the report with clinical history.------------------------------------------------32](#R5)

[R6: Provide primary and differential diagnosis based on the report with clinical history.------------------------------------------------39](#R6)

[R7: Provide primary and differential diagnosis based on the report with clinical history.------------------------------------------------46](#R7)

[R8: Provide primary and differential diagnosis based on the report with clinical history.------------------------------------------------53](#R8)

[R9: Provide primary and differential diagnosis based on the report with clinical history.------------------------------------------------59](#R9)

[R10: Provide primary and differential diagnosis based on the report with clinical history.------------------------------------------------67](#R10)

Authors' prompts are presented in blue, The sections manually deleted are marked in orange.whereas DeepSeek responses are shown in black.

R1: Provide primary and differential diagnosis based on the report with clinical history.

**Gender**:male **Age**:58

**Inspecti on items:**PET/CT (body without head).

**check on medication:** ^18^ Fluoro-deoxyglucose (^18^F -FDG)

**clinical diagnosi:** Abdominal fluid (to be investigated)

**Inspection equipment:** GE Discovery Elite PET/CT

Patient preparation, methods of examination and what is seen on examination:

Fasting blood glucose: 6.1 mmol/L, IV 18 F-FDG followed by sedentary rest. Oral 1.2% iohexol 1000 ml filled the gastrointestinal tract. Seventy-five minutes after the injection, supine position with arms raised to hold the head, body (from the base of the skull to the middle of the thighs) PET/CT was performed: body PET acquisition 7 beds, 2 minutes/bed. After intravenous injection of 68 Ga-FAPI, lying down and resting, PET/CT was performed 43 minutes after drug injection: 7 beds, 2 minutes/bed, 3D-TOF acquisition, OSEM reconstruction, CT attenuation correction; low-dose CT scanning on the same machine with a layer thickness of 3.3 mm. fusion imaging of PET and CT.

Neck: There are no structural or metabolic abnormalities in the soft tissues of the neck and cervical vertebrae. There were no abnormalities in the metabolism and structure of the sinuses, pharynx and parapharyngeal space. There were no abnormalities in the metabolism and density of submandibular gland and parotid gland on both sides. The vocal cords were symmetrical bilaterally, and there were no abnormalities in the metabolism and structure of the larynx. The thyroid gland was symmetrical in both lobes, with uniform metabolic distribution and density in the parenchyma. There were no abnormal lymph node concentrations in the neck and bilateral supraclavicular region.

Chest: Thorax is symmetrical, with increased breast glands bilaterally and mildly elevated metabolism, SUVmax 1.6. There is no abnormality in the morphology, density or metabolism of the chest wall's soft tissues and bony structures. The pleura was smooth bilaterally, and no pleural effusion shadow was seen. The texture of both lung fields was clear, and there were several scattered non-metabolic micronodules with a diameter of <5mm; non-metabolic streaks were seen in the lower lobe of the right lung. The mediastinum was centered, the trachea and bronchi were patent, and no enlarged lymph nodes or abnormal metabolism were seen in the mediastinum. The cardiac shadow was not large, the size and morphology of each atrium was in the normal range, the pericardial thickness was normal, there was no pericardial effusion, and the pulmonary artery trunk was thickened, with a diameter of about 36 mm. no abnormality was seen in the ascending aorta and the branches of the major blood vessels. There was no abnormal calcification in the coronary arteries. Multiple strips of hypermetabolism were seen throughout the esophagus, SUVmax 3.0-5.4, and no significant thickening of the wall was seen in the corresponding areas. A small, slightly hypermetabolic lymph node, SUVmax 1.7, diameter about 6 mm, was seen in the right internal breast area.

Abdominopelvic region: the abdomen is bulging, the peritoneum is morphologically disorganized with pancake-like thickening, especially in the greater omentum and mesentery, and multiple slightly elevated or hypermetabolic nodular shadows are seen within, with SUVmax 1.8-6.4.68 Increased uptake of abnormal contrast agent is seen on Ga-FAPI PET/CT imaging, which is wider than that of FDG. Massive fluid density shadows were seen in the abdominopelvic cavity, and multiple encapsulated effusions were seen in the perihepatic and subhepatic periosteum, the larger of which was about 19 mm× 28 mm, with the liver becoming smaller under compression. Gastric filling was poor, with diffuse metabolic elevation in the fundus and body of the stomach, SUVmax 4.5.68 Ga-FAPI PET/CT imaging showed that increased uptake of contrast was seen in the corresponding areas, SUVmax 7.6. A slightly higher metabolic small lymph node shadow was seen in the cardiophrenic angle area, SUVmax 0.8, and the size of the node was about 9mm× 7mm.

There were no abnormalities in the density and metabolism of the soft tissue and bone structure of the abdominopelvic wall. The location and metabolic distribution of the liver were not abnormal, with smooth margins and normal lobe proportions; there was uniform density within the liver parenchyma, no dilatation of the intrahepatic bile ducts, and normal hepatic portal structures. The gallbladder was not enlarged, the wall was not thick, there was no positive stone shadow in the gallbladder, and the common bile duct was not dilated. The pancreas did not show any abnormality in morphology, location and metabolic distribution, with clear contours, no abnormal density shadows in the pancreatic parenchyma, no dilatation of the pancreatic ducts, and clear peripancreatic fat interstitium. The spleen was not large, with smooth margins and uniform density and metabolism in the splenic parenchyma. The morphology, density and metabolism of bilateral adrenal glands were not abnormal. The morphology and density of both kidneys were not abnormal, and the bilateral renal pelvis and ureters were not dilated, with no abnormal contrast material retention within them. No enlarged lymph nodes or abnormal metabolic shadows were seen in the retroperitoneum. The bladder was poorly filled and no positive stone images were seen in the bladder. The prostate was enlarged with punctate calcified shadows and no metabolic abnormalities. No abnormal enlarged lymph nodes or metabolic shadows were seen in the pelvis or bilateral inguinal region. Some colorectal mesentery was also seen as physiologic regurgitant shadows.

Left posterior sciatic tuberosity with soft tissue swelling and increased metabolism, SUVmax 3.6, with internal calcified shadows. Right anterior 4th and 5th ribs with scab formation and mild metabolic increase, SUVmax 1.6.

Medical History:

Patient originated with abdominal distension, not accompanied by abdominal pain, consulted the Second Hospital of Hebei Medical University, investigated thoracic, abdominal and pelvic CT suggesting multiple tiny nodules in both lungs, peritoneal pancake and nodular thickening, epigastric is prominent, peritoneal inflammatory changes or peritoneal metastasis may be possible, large amount of peritoneal and pelvic fluid; perihepatic, hepatic periheperitoneal multiple encapsulated effusions, the larger one is about 5.3cm*2.9cm; hepatic volume is reduced; Multiple small cysts in both kidneys. Gastroscopy showed: multiple polyps of the stomach, chronic non-atrophic gastritis. Enteroscopy: fecal stone in the appendiceal fossa. Autoantibody assay: no abnormality seen. T-cell test for tuberculosis infection: no abnormality. After admission, he was given piperacillin sodium tazobactam for anti-infection, peritoneal puncture and drainage, furosemide combined with spironolactone diuretic treatment, the patient's abdominal distension was relieved, but the cause of ascites was not clear, and it was suggested that further peritoneal biopsy should be carried out, which the patient's family refused. The patient's family refused. 4 days ago, the patient's abdominal distension worsened, and on 2024.10.13, she visited Gaoyi County Hospital, where abdominal ultrasound indicated ascites. cEA: 97.77ng/ml, CA125: 76.25U/ml, CA199: 1168.92IU/ml, and was not diagnosed and treated.

Purpose of the examination: to find the primary focus of unexplained ascites.

**Inspection Observations:**

1 Diffuse peritoneal thickening with multiple hypermetabolic nodules and abdominopelvic effusion, consider the possibility of metastatic peritoneal carcinoma with abdominopelvic effusion, suggest peritoneal biopsy for pathologic confirmation.

2 FDG and FAPI are metabolically increased in the fundus and body of the stomach, primary gastric cancer is not excluded, and another gastroscopy biopsy is recommended for pathology; the esophagus has multiple striated hypermetabolic shadows, and the possibility of esophagitis is considered.

3 Slightly hypermetabolic small lymph nodes in the right internal breast area and in the cardiophrenic angle area, consider inflammatory lymph nodes as possible.

Scattered non-metabolic microscopic nodules in both lungs are considered benign and regular review is recommended. Fibrous striae in the lower lobe of the right lung.

Male breast development. Thickening of the pulmonary trunk.

4 Inflammatory nodule posterior to the left sciatic tuberosity. Old fracture of the right anterior 4th and 5th ribs. Enlarged prostate with calcification.

Follow-up findings: (Peritoneal mass) Punctured tissue: small amount of fibrovascular and large amount of non-epithelial cellular mucus, consider abdominal pseudomucinous tumor. Immunohistochemical staining: CKpan (-), CDX-2 (-), CD34 (vascular +), Ki-67 (scattered +).

Thinking:

Okay, I need to write an examination opinion based on this PET/CT report and the provided medical history, including a differential diagnosis. Let me start by going through the information step by step.

First, the patient is a 58-year-old male presenting with abdominal distension accompanied by ascites. The ascites' cause remains unclear despite previous investigations. The medical history mentions peritoneal thickening, multiple lung nodules, elevated tumor markers (CEA, CA125, CA199), and a refusal of a peritoneal biopsy. The PET/CT was done to find the primary cause of the ascites.

Looking at the PET/CT findings:

**Neck:** No significant abnormalities.

**Chest:** Mildly increased metabolism in breast glands, non-metabolic lung nodules, and a hypermetabolic lymph node in the right internal mammary area.

**Abdominopelvic Region:** Peritoneal thickening with hypermetabolic nodular shadows, massive ascites, liver compression, diffuse metabolic elevation in the stomach, and a small hypermetabolic lymph node near the diaphragm. Elevated SUVmax values in both FDG and Ga-FAPI scans.

**Other Findings:** Left posterior sciatic tuberosity swelling and right rib lesions.

The key points here are the peritoneal involvement, ascites, elevated tumor markers, and hypermetabolic lesions. The differential diagnosis should consider both malignant and non-malignant causes. Malignant causes could include peritoneal carcinomatosis (from gastric, pancreatic, or other gastrointestinal cancers), mesothelioma, or lymphoma. Non-malignant possibilities might be tuberculosis peritonitis, inflammatory conditions, or chronic infections. However, the elevated tumor markers and hypermetabolic lesions lean towards malignancy.

The patient’s elevated CA199 is often associated with pancreatic or gastrointestinal cancers. The diffuse stomach metabolic elevation might suggest gastric cancer, which could metastasize to the peritoneum. The Ga-FAPI uptake being higher than FDG is notable since FAPI is a newer tracer targeting fibroblast activation protein, often upregulated in cancers.

But I should also consider other possibilities. For example, peritoneal tuberculosis can present similarly with ascites and peritoneal thickening, but the tumor markers might not be as elevated. However, the patient had a negative T-cell test for tuberculosis, which reduces this likelihood. Inflammatory conditions like sclerosing peritonitis are possible but less likely with the metabolic activity seen.

The presence of multiple lung nodules adds complexity. They are described as non-metabolic, which might suggest benign lesions, but in the context of possible malignancy, metastatic disease can't be ruled out entirely.

Given the refusal of a biopsy, definitive diagnosis is challenging. However, the combination of imaging findings and tumor markers strongly suggests a malignant process, likely peritoneal carcinomatosis secondary to an occult gastrointestinal primary, possibly gastric given the stomach findings.

In the differential diagnosis, I should list the most probable conditions in order of likelihood, supported by the available data. I need to structure the examination opinion clearly, starting with the most likely diagnosis and then alternatives.

Answer:

**Examination Opinion and Differential Diagnosis**

**Summary of Findings:**
A 58-year-old male presents with progressive abdominal distension and unexplained ascites. PET/CT reveals:

1. **Peritoneal Involvement:** Pancake-like thickening, hypermetabolic nodules (SUVmax 1.8–6.4 on FDG; higher on Ga-FAPI), and massive ascites with encapsulated effusions.
2. **Gastric Abnormalities:** Diffuse metabolic elevation in the fundus/body (SUVmax 4.5 FDG, 7.6 Ga-FAPI).
3. **Elevated Tumor Markers:** CEA (97.77 ng/mL), CA125 (76.25 U/mL), CA199 (1168.92 IU/mL).
4. **Ancillary Findings:** Small hypermetabolic lymph nodes (right internal mammary, diaphragmatic), non-metabolic lung micronodules, and rib/scapular lesions.

**Key Clinical Concerns:**
The primary concern is **malignant ascites secondary to peritoneal carcinomatosis**, likely originating from an occult gastrointestinal malignancy. The elevated CA199 and gastric metabolic activity suggest a gastric or pancreatic primary. Ga-FAPI avidity (targeting cancer-associated fibroblasts) further supports malignancy.

**Differential Diagnosis:**

1. **Peritoneal Carcinomatosis** (Most Likely):
   - **Primary Suspicions:**
     - **Gastric Adenocarcinoma:** Diffuse gastric metabolic elevation, elevated CA199/CEA.
     - **Pancreaticobiliary Malignancy:** High CA199, though pancreatic imaging was unremarkable.
   - **Supporting Evidence:** Hypermetabolic peritoneal nodules, ascites, tumor markers.
2. **Peritoneal Mesothelioma** (Less Likely):
   - Chronic ascites and peritoneal thickening are typical, but mesothelioma often lacks elevated CA199. Ga-FAPI uptake may occur but is non-specific.
3. **Tuberculous Peritonitis** (Unlikely but to Consider):
   - Negative T-cell TB test and lack of constitutional symptoms (e.g., fever, weight loss) reduce likelihood. Non-caseating granulomas would typically show FDG avidity.
4. **Lymphoma** (Possible but Atypical):
   - Disseminated lymphoma could explain hypermetabolic nodes and ascites, but diffuse gastric involvement is less common.
5. **Metastatic Disease from Occult Primary**:
   - Elevated CEA/CA125/CA199 may indicate colorectal, ovarian, or hepatobiliary origins, though imaging lacks clear evidence.

**Recommendations:**

1. **Tissue Diagnosis:** Urge peritoneal biopsy or laparoscopic exploration for histopathology, despite prior refusal.
2. **Gastroscopic Re-evaluation:** Target areas of metabolic activity in the stomach for biopsy.
3. **Cytology of Ascitic Fluid:** Repeat paracentesis with cytology and immunohistochemistry (e.g., CK7/CK20, CA199 staining).
4. **Monitoring:** Serial tumor markers and imaging to assess progression.

**Conclusion:**
The findings strongly suggest **malignant peritoneal carcinomatosis**, likely secondary to an occult gastric or pancreaticobiliary primary. Non-malignant etiologies are less probable given biochemical and imaging features. Definitive diagnosis requires histopathological confirmation.

R2 Provide primary and differential diagnosis based on the report with clinical history.

**Gender**:male **Age**:74

**Inspecti on items:**PET/CT (body including head).

**check on medication:** ^18^ Fluoro-deoxyglucose (^18^F -FDG)

**clinical diagnosi:** 1.(back) pain (cause to be investigated); 2. hypertension grade 3 (very high risk); 3. old cerebral infarction; 4.(lung) after radiofrequency ablation

**Inspection equipment:** GE Discovery Elite PET/CT

**Patient preparation, method of examination and what is seen on examination:**

Fasting blood glucose: 3.1 mmol/L, IV 18 F-FDG followed by sedentary rest. Oral 1.2% iohexol 1000 ml filled the gastrointestinal tract. Sixty-one minutes after the injection supine position arms up holding the head, body (from the base of the skull to the middle of the thighs) was performed+ Cranial PET/CT: Head PET examination 1× 108 counts, 1 bed. Body PET acquisition 7 beds, 2 min/bed, 3D-TOF acquisition, OSEM reconstruction, CT attenuation correction; low-dose CT scan on the same machine, layer thickness 3.3 mm. fusion imaging of PET, CT.

Skeletal: Diffuse hypermetabolism of cranial bones, midshaft bones, proximal bones of extremities, SUVmax 28.8, uneven bone density of ribs bilaterally, multiple small patchy hypodense shadows were seen, in which significant osteolytic bone destruction was seen in the right 8th posterior ribs, right 12th posterior ribs, 11th thoracic vertebrae and 8th thoracic vertebrae, and a swollen soft-tissue shadow was seen adjacent to the area of bone destruction of the right 8th posterior ribs with an extent of about 27mm× 20mm. There was a diffuse increase in cranial bone density, with a plate barrier CT value of 890 HU.

Head and neck: There were no abnormalities in the soft tissue structure and metabolism of the head and neck. Bilateral cerebral hemispheres had normal morphology, symmetrical metabolic distribution, no abnormalities in metabolism and density in the gray and white matter areas of each lobe of the brain, clear visualization of the basal ganglia, widening and deepening of the ventricles and cerebral pools, and centered structures in the midline. The cerebellar hemispheres on both sides were symmetrical, with no abnormalities in density and metabolism. There were no abnormalities in the metabolism and structure of the sinuses, pharynx and parapharyngeal space. Metabolism and density of submandibular gland and parotid gland were not abnormal. The vocal cords were symmetrical bilaterally, and there were no abnormalities in the metabolism and structure of the larynx. The thyroid lobes were symmetrical, with a nodular hypermetabolic shadow in the left lobe, SUVmax 8.5, hypermetabolic range of about 12 mm× 11 mm, and no obvious abnormalities in the morphology and density of the corresponding areas; the rest of the thyroid parenchyma had a uniform metabolic distribution and density. A small hypermetabolic lymph node in the right supraclavicular region, SUVmax 5.5, clear border, size about 7mm× 4mm, no abnormal lymph node concentration in the neck and left supraclavicular region.

Thorax: The thorax is symmetrical, and there is no abnormality in the morphology, density and metabolism of the soft tissue structures of the chest wall. Bilateral pleura was less smooth and no pleural effusion shadow was seen. There was no metabolic abnormality in the anterior segment of the right upper lobe; hypermetabolic nodule in the apical segment of the right upper lobe, SUVmax 4.5, with lobulation, burr and pleural pulling at the margins, about 11mm× 9mm in size, and multiple anaplastic nodules in the upper lobes of both lungs and the outer basal segment of the lower lobe of the left lung, with the largest being a ground-glass nodule in the anterior segment of the right upper lobe of the right lung, about 7mm× 5mm in size, and calcified foci in the posterior basal segment of the lower lobe of the left lung, about 4mm 3mm in size. The size of the left lower lobe was about 4mm× 3mm. The rest of the lungs had multiple cystic translucent shadows. The mediastinum was centered, trachea and bronchus were patent, and the mediastinal area 4R was highly metabolically enlarged with lymph nodes, SUVmax 23.8, borders were still clear, size of about 28mm× 21mm. The heart shadow was not large, the size and morphology of atria and chambers were in the normal range, pericardium was of normal thickness, there was no pericardial effusion, and there was no abnormality of the ascending aorta and the major vascular branches. There is no abnormality in the ascending aorta and major vascular branches. There is calcification in the coronary artery tract.

Abdomen: There is no abnormality in the soft tissue structure density and metabolism of the abdominal wall, and there is no sign of fluid accumulation in the abdominal cavity. Gastric filling was acceptable, and no abnormalities were seen in the metabolism of the gastric wall. Liver morphology and position were not abnormal, metabolism was diffusely increased, SUVmax 15.8, margins were smooth, proportion of liver lobes was normal; density within liver parenchyma was diffusely decreased, mean CT value 42HU, intrahepatic bile ducts were not dilated, hepatic portal structure was normal. The gallbladder was not enlarged, the wall was not thick, there were no positive stone shadows in the gallbladder, and the common bile duct was not dilated. The pancreas did not show any abnormality in morphology, location and metabolic distribution, with clear contours, no abnormal density shadows in the pancreatic parenchyma, no dilatation of the pancreatic ducts, and clear peripancreatic fat interstitium. The spleen was not large, with smooth margins and uniform density and metabolism in the splenic parenchyma. The morphology, density and metabolism of bilateral adrenal glands were not abnormal. The morphology and density of both kidneys were not abnormal, and the bilateral renal pelvis and ureters were not dilated, with no abnormal contrast material retention within them. There were no enlarged lymph nodes or abnormal metabolic shadows in the retroperitoneum. Some of the colonic intestinal tubes showed physiologic uptake shadows.

Pelvis: There were no abnormalities in the density and metabolism of the soft tissue structures of the pelvic wall. No signs of fluid accumulation in the pelvis. There were no positive stone shadows in the poorly filled bladder or bladder. There are no abnormalities in density and metabolism in the prostate area. The fat interstitial spaces in the pelvis were clear, and no abnormal enlarged lymph nodes or metabolic shadows were seen in the pelvis or bilateral inguinal region. Some rectal intestines in the pelvis were also seen as physiologic regurgitant shadows.

**History:** The patient had back pain for 1 month with no obvious cause, intermittent dull pain, aggravated by coughing, accompanied by dull pain and discomfort in the forehead and lower limbs, accompanied by muscle weakness, limited movement, need to walk with support, accompanied by difficulty in turning over at night, no burning pain, no aggravation of the symptom after activity, in order to seek further information, the outpatient clinic checked ★ alanine aminotransferase: 83.9U/L↑ ; ★ aspartate aminotransferase enzyme: 71.7U/L ; γ-glutamyltransferase:398.7U/L ; alkaline phosphatase:231.2U/L ; ★ aspartate aminoacyltransferase: 398.7U/L ; alkaline phosphatase: 231.2U/L 71.7U/L ;↑★ γ-glutamyltransferase:398.7U/L↑ ;Alkaline phosphatase:231.2U/L↑ ;★Lactate dehydrogenase:386.9U/L↑ ;αhydroxybutyrate dehydrogenase:326.4U/L↑ ;Creatine kinase-MB isoenzyme:101.2U/L↑ ;★Calcium:2.62mmol/L↑ ;★Total cholesterol:5.48mmol/L↑ ; ★ Triglycerides:3.22mmol/L↑ ;C-Reactive Protein (CRP):8.15mg/L↑ ;Sedimentation:23mm/h↑ ;Admitted to the department of medicine with "pain (cause to be investigated)". 2 years after ablation of a pulmonary nodule

Since the onset of the disease, the patient's spirit can be, appetite, sleep can be, normal urination and defecation, weight compared with the previous no significant changes. The patient had no fever, sore throat, cough, diarrhea, loss of smell (taste) and other symptoms in the past two weeks, and no antipyretic drugs were used.

Inspection Observations:

1 Diffuse hypermetabolism with localized bone destruction in cranium, midshaft bones, and proximal bones of limbs; diffuse metabolic increase in liver; and hypermetabolic enlarged lymph nodes in mediastinal region 4R. Primary consideration for hematologic malignancy, lymphoma possibility, not except solid tumors with bone, liver and lymph node metastasis, suggest bone marrow biopsy pathology.

2 Hypermetabolic nodule in the apical segment of the upper lobe of the right lung, lung cancer? Granulomatous nodule? Striated and striated shadow in the anterior segment of the right upper lobe of the lung, consider post ablation changes. Multiple nonmetabolic nodules in the upper lobes of both lungs and the lower lobe of the left lung, consider benign.

3 Hypermetabolic shadow in the left lobe of the thyroid gland, suggesting ultrasound. Small hypermetabolic lymph node on the right supraclavicular bone, consider chronic inflammatory lymph node.

4 Brain atrophy. Coronary artery calcification. Fatty liver.

RESULTS: (Bone marrow) Biopsy tissue: carcinoma was seen in bone trabeculae and interstitial bone marrow, and combined with immunohistochemical staining, it was consistent with lung complex small cell carcinoma (small cell carcinoma + large cell neuroendocrine carcinoma).

Immunohistochemical staining: CKpan (+), TTF-1 (+), Syn (+), CgA (+), LCA (-), Ki-67 (~40%+), CD56 (+), CK5/6 (-), P40 (-)

Thinking:

Alright, I need to write an examination opinion based on the provided PET/CT report and medical history, including a differential diagnosis. Let me start by understanding the key findings from the report.

First, the patient is a 74-year-old male with a history of back pain for one month, hypertension grade 3, old cerebral infarction, and a past pulmonary nodule ablation two years ago. The PET/CT shows several significant findings:

1. **Skeletal System**: Diffuse hypermetabolism in cranial bones, midshaft bones, and proximal extremity bones with SUVmax 28.8. There are osteolytic lesions in the right 8th and 12th posterior ribs, 11th and 8th thoracic vertebrae, and a swollen soft-tissue shadow near the right 8th rib. High bone density in cranial bones (CT value 890 HU).

2. **Thyroid**: A hypermetabolic nodule in the left lobe (SUVmax 8.5).

3. **Lungs**: A hypermetabolic nodule in the right upper lobe (SUVmax 4.5) with lobulation and pleural pulling. Multiple anaplastic nodules in both lungs, including a frosted-glass nodule in the right upper lobe.

4. **Mediastinum**: Enlarged lymph node in the 4R area with SUVmax 23.8.

5. **Liver**: Diffuse increased metabolism (SUVmax 15.8) and decreased parenchymal density (CT value 42 HU).

6. **Lab Findings**: Elevated liver enzymes (ALT, AST, GGT, ALP), LDH, CK-MB, calcium, cholesterol, triglycerides, CRP, and ESR.

The main clinical issue is unexplained back pain with potential metastatic bone lesions. The differential diagnosis should consider possible malignancies given the hypermetabolic lesions. Given the multiple osteolytic lesions and hypermetabolic lymph nodes, metastatic cancer is a strong possibility. The primary could be from the lungs (given past ablation and current lung nodules), thyroid (thyroid nodule with SUVmax 8.5), or another source. The liver findings could indicate metastases or a primary hepatic process. Additionally, metabolic bone diseases like Paget’s disease (due to cranial sclerosis) or multiple myeloma (due to osteolytic lesions) should be considered. Non-malignant causes like infection or inflammatory conditions are less likely but need exclusion.

I need to structure the examination opinion to summarize findings, list differential diagnoses with reasoning, and suggest further steps.

Answer:

**Examination Opinion and Differential Diagnosis**

**Summary of Key Findings**

1. **Skeletal System**:
   - Diffuse hypermetabolic activity (SUVmax 28.8) in cranial bones, midshaft, and proximal extremity bones.
   - Multiple osteolytic lesions (right 8th/12th ribs, T8/T11 vertebrae) with adjacent soft-tissue swelling.
   - Cranial bone sclerosis (CT 890 HU).
2. **Thyroid**:
   - Hypermetabolic nodule in the left lobe (SUVmax 8.5, 12mm×11mm).
3. **Lungs**:
   - Hypermetabolic nodule in the right upper lobe (SUVmax 4.5) with lobulation/pleural involvement.
   - Multiple bilateral anaplastic nodules, including a frosted-glass lesion.
4. **Mediastinum**:
   - Enlarged hypermetabolic lymph node in 4R region (SUVmax 23.8, 28mm×21mm).
5. **Liver**:
   - Diffuse hypermetabolism (SUVmax 15.8) and decreased parenchymal density (CT 42 HU).
6. **Laboratory Abnormalities**:
   - Elevated liver enzymes (ALT, GGT, ALP), LDH, CK-MB, inflammatory markers (CRP, ESR), and hypercalcemia.

**Differential Diagnosis**

1. **Metastatic Malignancy**:
   - **Primary Suspects**:
     - **Lung Cancer**: History of pulmonary nodule ablation and current hypermetabolic lung nodule with pleural involvement.
     - **Thyroid Carcinoma**: Hypermetabolic thyroid nodule (SUVmax 8.5) raises concern for malignancy.
     - **Hepatocellular Carcinoma (HCC)**: Diffuse hepatic hypermetabolism and hypoattenuation suggest possible primary liver malignancy.
   - **Supporting Evidence**: Widespread osteolytic lesions, mediastinal lymphadenopathy, and hypercalcemia (paraneoplastic syndrome).
2. **Multiple Myeloma**:
   - Lytic bone lesions, hypercalcemia, and elevated LDH/CRP/ESR align with myeloma. Requires serum protein electrophoresis and bone marrow biopsy for confirmation.
3. **Paget’s Disease of Bone**:
   - Cranial sclerosis and elevated ALP suggest Paget’s, but high SUVmax and osteolytic lesions are atypical.
4. **Infectious/Inflammatory Process**:
   - Osteomyelitis or granulomatous disease (e.g., tuberculosis) could explain lytic lesions and inflammation but less likely given lack of fever and high SUVmax.
5. **Metabolic Bone Disease**:
   - Secondary hyperparathyroidism from hypercalcemia, but imaging findings are more consistent with malignancy.

**Recommendations**

1. **Tissue Diagnosis**:
   - Biopsy the most accessible lesion (e.g., right 8th rib soft-tissue mass or mediastinal lymph node).
   - Fine-needle aspiration (FNA) of the thyroid nodule.
2. **Laboratory Workup**:
   - Serum/urine protein electrophoresis, free light chains (for myeloma).
   - Tumor markers: CEA, AFP, CA19-9, thyroglobulin, calcitonin.
   - Parathyroid hormone (PTH) and vitamin D levels.
3. **Imaging**:
   - Contrast-enhanced CT/MRI of the liver to evaluate for HCC.
   - Whole-body MRI or skeletal survey for myeloma staging.
4. **Multidisciplinary Review**:
   - Coordinate with oncology, endocrinology, and orthopedics for further management.

**Conclusion**: The findings are highly suspicious for metastatic malignancy, with lung, thyroid, or liver as potential primary sites. Multiple myeloma remains a critical differential. Urgent histopathological confirmation is warranted to guide therapy.

R3 Provide primary and differential diagnosis based on the report with clinical history.

**Gender**:male **Age**:34

**Inspecti on items:**PET/CT (body without head).

**check on medication:** ^18^ Fluoro-deoxyglucose (^18^F -FDG)

**clinical diagnosi:** lung cancer

**Inspection equipment:** GE Discovery Elite PET/CT

Patient preparation, method of examination and what is seen on examination:

Fasting blood glucose: 4.8 mmol/L, IV 18 F-FDG followed by sedentary rest. Oral 1.2% iohexol 1000 ml filled the gastrointestinal tract. Fifty minutes after injection supine position arms raised above head, body (from skull base to mid-thigh) PET/CT was performed: acquisition of 8 beds, 2 min/bed. 3D-TOF acquisition, OSEM reconstruction, CT attenuation correction; low-dose CT scanning on the same machine with layer thickness of 3.3 mm. fusion imaging of PET and CT.

Lymphatic system: multiple hypermetabolic lymph node shadows were seen in the right supraclavicular, mediastinum, both lung hilums, diaphragm foot zone, retroperitoneum and left inguinal region within the scanning area, with predominantly metabolically elevated lymph nodes at the mediastinum and both lung hilums with increased size, SUVmax 21.5, with the largest being about 25mm× 18mm in diameter, with homogeneous density and well-defined margins. The spleen was small, with smooth margins and homogeneous density, and multiple nodular metabolically elevated shadows were seen in the splenic parenchyma, with a SUVmax of 8.1, and the largest one was about 8 mm in diameter, and no density abnormality was seen in the corresponding areas. No density or metabolic abnormality was seen in the lymphatic system in the rest of the scan.

Nasopharynx and neck: no metabolic or structural abnormalities in the sinuses, pharynx and parapharyngeal space. The vocal cords were not thick bilaterally, and there were no metabolic or structural abnormalities in the larynx. Bilateral submandibular gland and parotid gland metabolism and density were not abnormal. The density of the bilobar thyroid gland is not uniform, and no abnormal metabolic shadows are seen.

Chest: The thorax was symmetrical, and no abnormal density or hypermetabolic shadows were seen in the soft tissue and bony structures of the chest wall. Bilateral pleura is poorly illuminated, multiple nodular hypermetabolic shadows are seen in pleura and subpleura, SUVmax 2.5, margins are poorly defined. Multiple mound-like hypermetabolic elevations were seen in the pleural region bilaterally, with rough edges, especially on the right side, SUVmax 17.2; no effusion was seen in the pleural cavity bilaterally. There were multiple small nodules and micronodular shadows in the middle and lower lobes of the right lung, with no metabolic abnormality, and the large one was about 7 mm in diameter. interstitial thickening in the upper lobe of the right lung, and the remaining lung fields did not have any obvious density or metabolic abnormality. The mediastinum was centered, and the trachea and bronchi were patent. The cardiac shadow was not large, the size and morphology of each atrium was in the normal range, the pericardial thickness was normal, no pericardial effusion was seen, and there was no abnormality in the ascending aorta and the branches of the major vessels. No calcification was seen in the coronary artery tract.

Abdomen: no abnormal density or hypermetabolic shadows were seen in the remaining abdominal wall soft tissues and bony structures, and there were no signs of fluid accumulation in the abdominal cavity. Gastric filling is possible, and the metabolism of the gastric wall shows no abnormality. There was no abnormality in the morphology, position and contrast distribution of the liver, with smooth margins and normal lobe proportions; the liver parenchyma was diffusely slightly hypodense, with an average CT value of about 44 HU, and a dense nodular shadow was seen at the apex of the liver. No abnormal density or metabolic shadows were seen in the remaining liver parenchyma; there was no dilatation of the intrahepatic bile ducts, and the hepatic portal structure was normal. The gallbladder was not enlarged, the wall was not thick, no stone shadow was seen in the gallbladder, and the common bile duct was not dilated. The pancreas did not show any abnormality in morphology, location and distribution of visualizer, with clear contour, no abnormal density shadow in the pancreatic parenchyma, no dilatation of pancreatic ducts, and clear peripancreatic fat interstitial space. The morphology, density and metabolism of bilateral adrenal glands showed no abnormality. The morphology and density of both kidneys were not abnormal, and there was no dilatation of bilateral renal pelvis and ureter, and no abnormal contrast retention shadow was seen in them. Some of the intestines in the abdominal cavity physiologically ingested the contrast medium.

Pelvis: There were no abnormal densities or hypermetabolic shadows in the soft tissues and bony structures of the remaining pelvic wall, and no signs of fluid accumulation in the pelvis. Bladder filling was fair, and no positive stone images were seen in the bladder. The density and metabolism of the prostate and seminal vesicle region were not abnormal. Intrapelvic fat spaces were clear and no abnormal hypermetabolic shadows were seen. The density and metabolism of the intestines in the pelvis were not abnormal.

History: One year ago, the patient underwent chest CT in our hospital, which indicated: "Inflammation in the upper lobes of both lungs and the lower lobe of the right lung, please combine with the clinic. Small solid nodule in the anterior basal segment of the lower lobe of the right lung, and it was recommended to review it in six months. Tiny nodule in the outer basal segment of the lower lobe of the left lung. Localized thickening of bilateral interlobular pleura. Fatty liver. S8 calcified foci in the liver." . No special treatment was given and the patient complained of no special discomfort.

2024-12-07 The patient's chest CT was reviewed in our outpatient clinic and showed: "Shadows in both lungs were enlarged and increased compared with the previous one, and the lymph nodes in the hilar and mediastinal regions were enlarged". Chest CT plain scan+ enhancement suggests: mass shadow of the upper lobe of the right lung; multiple nodular and mound-like increased density shadows in the pleura and interlobar fissures of both lungs; multiple enlarged lymph nodes in the mediastinum and in the hilum of both lungs, in order to -consider that the possibility of nodular disease is high, and biopsy is recommended to exclude malignant lesions. Solid nodule in the anterior basal segment of the lower lobe of the right lung, with - significant change from the previous (2024-12-07), suggesting review in six months. Fatty liver. S8 calcified foci in the liver. Scattered small lymph nodes lateral to the lesser curvature of the stomach and retroperitoneum.

Lung Tumor IV. (20241209): NSE 32.8↑ ng/mL,Cyfra211: 6.04↑ ng/mL; remaining CEA, SCC, proGRP normal.

Mycobacterium tuberculosis-specific cellular immunoreactivity assay^(20241210): Mycobacterium tuberculosis-specific cellular immunoreactivity assay Negative, no antigenic stimulus value N 0.124 IU/ml, positive antigenic stimulus value P >10.000 IU/ml, Mycobacterium tuberculosis-specific antigenic stimulus value T 0.145 IU/ml.

Blood sedimentation, blood routine, liver and kidney function, antinuclear antibody profile, and rheumatology were normal.

Purpose of examination: characterization of lung occupancy.

Inspection Observations:

1 Multiple hypermetabolic lymph nodes above and below the diaphragm within the scanning range, some of them enlarged, multiple nodular-like metabolic increase in the spleen, multiple nodular, mound-like hypermetabolic elevations or patchy shadows in the pleura and subpleura bilaterally (the right side is prominent), all of the above are considered to be malignant, and the first and foremost consideration is that the possibility of lymphoma extensive involvement is high, and it is recommended that biopsies at the right upper subpleural hypermetabolic lesion or the high metabolic lymph nodes of the left inguinal area should be taken to make a definitive diagnosis.

Interstitial changes in the right lung, consider the possibility of intrapulmonary lymphatic involvement.

2 Mild fatty liver.

Follow-up results: nodular disease

Thinking:

Alright, I need to write an examination opinion based on this PET/CT report and the patient's medical history, including a differential diagnosis. Let me start by understanding the key points from the report.

The patient is a 34-year-old male with a clinical diagnosis of lung cancer. The PET/CT scan shows multiple hypermetabolic lymph nodes in various regions, including the mediastinum and both lung hilar areas, with high SUVmax values (up to 21.5). There are also hypermetabolic nodules in the spleen and pleural regions. The patient's history includes previous chest CT findings of lung inflammation and nodules, which have increased in size over time. Recent tumor markers like NSE and Cyfra211 are elevated, while other markers like CEA and SCC are normal. The tuberculosis test is negative, and other blood work is normal.

First, I need to summarize the significant findings. The high metabolic activity in the lymph nodes and pleural nodules suggests malignancy, likely metastatic lung cancer. However, other possibilities should be considered. For example, infections like tuberculosis or fungal infections could cause lymphadenopathy, but the negative TB test makes TB less likely. Sarcoidosis is another possibility, though the elevated tumor markers point more towards malignancy. Lymphoma could also present with widespread hypermetabolic lymph nodes, but the presence of lung nodules and pleural involvement might favor a primary lung cancer with metastases.

I should also mention the splenic nodules. While they have a lower SUVmax, they could be metastatic lesions or possibly benign lesions. The previous liver findings (fatty liver, calcified foci) don't seem directly related but should be noted. The differential diagnosis should list the most probable conditions based on these findings, ordered by likelihood. Finally, recommendations for biopsy to confirm malignancy and further imaging or tests if needed.

Wait, the patient's age is 34, which is young for lung cancer, but not impossible. Also, the history of inflammation in the lungs a year ago that progressed could suggest an aggressive malignancy. I need to make sure the differential includes both malignant and benign conditions, even if less likely. Maybe also consider metastatic disease from another primary, though lung primary seems most likely given the clinical context.

Answer:

**Examination Opinion and Differential Diagnosis**

**Summary of Findings:**

1. **PET/CT Findings:**
   - Multiple hypermetabolic lymph nodes (SUVmax 21.5) in the mediastinum, bilateral lung hilar regions, retroperitoneum, and left inguinal area.
   - Pleural/subpleural nodular hypermetabolic lesions (SUVmax up to 17.2) with poorly defined margins.
   - Splenic parenchymal nodular hypermetabolic foci (SUVmax 8.1).
   - Right lung middle/lower lobe nodules (non-metabolic) and interstitial thickening.
   - Fatty liver and hepatic calcified foci (S8 segment).
2. **Clinical History:**
   - Progressive enlargement of lung lesions and lymphadenopathy over one year.
   - Elevated tumor markers: NSE (32.8↑ ng/mL) and Cyfra211 (6.04↑ ng/mL).
   - Negative tuberculosis-specific immunoreactivity.
   - Previous imaging suggestive of inflammatory changes evolving into suspicious malignant features.

**Differential Diagnosis:**

1. **Primary Lung Malignancy with Metastases (Most Likely):**
   - Aggressive lung adenocarcinoma or squamous cell carcinoma, given the high metabolic activity in lymph nodes, pleural involvement, and elevated Cyfra211/NSE. The young age (34 years) is atypical but not exclusionary.
   - Splenic and pleural lesions likely represent metastatic spread.
2. **Lymphoma:**
   - Widespread hypermetabolic lymphadenopathy and splenic involvement could suggest Hodgkin’s or non-Hodgkin’s lymphoma. However, pleural nodules and lung primary lesions are less characteristic.
3. **Metastatic Disease from an Extrapulmonary Primary:**
   - Less likely given the absence of other primary tumor indicators, but requires exclusion via thorough clinical evaluation.
4. **Granulomatous Disease (e.g., Sarcoidosis):**
   - May present with lymphadenopathy and pulmonary nodules, but elevated tumor markers and progressive lesions argue against this.
5. **Infectious Etiology (e.g., Fungal Infection):**
   - Negative TB testing and lack of response to prior conservative management reduce likelihood.

**Recommendations:**

1. **Tissue Biopsy:** Urgent histopathological confirmation (e.g., mediastinal lymph node or pleural lesion biopsy) to confirm malignancy and subtype.
2. **Staging Workup:** Brain MRI and bone scan to exclude distant metastases.
3. **Oncologic Referral:** For multidisciplinary management pending biopsy results.
4. **Follow-Up Imaging:** Short-interval CT for non-metabolic lung nodules to monitor stability.

**Conclusion:**
The imaging and clinical findings strongly suggest a primary lung malignancy with regional and distant metastases. Lymphoma and granulomatous disease remain less likely but require exclusion. Immediate biopsy is critical to guide definitive therapy.

R4 Provide primary and differential diagnosis based on the report with clinical history.

**Gender**:male **Age**:58

**Inspecti on items:**PET/CT (body without head).

**check on medication:** ^18^ Fluoro-deoxyglucose (^18^F -FDG)

**clinical diagnosi:** Pancreatic Occupancy(?)

**nspection equipment:** GE Discovery Elite PET/CT

Patient preparation, method of examination and what is seen on examination:

Fasting blood glucose: 11.0 mmol/L, IV 18F-FDG followed by sedentary rest. Oral 1.2% iohexol 1000 ml filled the gastrointestinal tract. Forty-eight minutes after drug injection, supine position with arms raised to hold the head, body (from the base of the skull to the middle of the thighs) PET/CT was performed: body PET acquisition in 7 beds, 2 minutes/bed, 3D-TOF acquisition, OSEM reconstruction, CT attenuation correction; low-dose CT scanning on the same machine, layer thickness of 3.3 mm. fusion imaging of PET and CT.

Neck: The soft tissues of the neck and the bone structure of the cervical vertebrae are free of abnormalities, with normal density and metabolism. The metabolism and structure of the nose, pharynx and neck intervals were normal. The vocal cords were symmetrical bilaterally, and there were no abnormalities in the metabolism and structure of the larynx. There were no abnormalities in the metabolism and density of the submandibular gland and parotid gland bilaterally. No abnormal density or hypermetabolic shadow was seen in the bilobar thyroid gland. There were no enlarged lymph nodes in the neck and bilateral supraclavicular region, and the metabolism was not abnormal.

Thorax: The thorax is symmetrical, with no abnormalities in the soft tissue and bony structure of the chest wall, and normal density and metabolism. The pleura was smooth bilaterally and no pleural effusion was seen. Scattered non-metabolic small solid nodular shadows in both lungs, short diameter <5mm, clear borders. A cystic air-containing translucent shadow in the medial segment of the right middle lobe of the lung, with no metabolic abnormality, was about 10mm× 5mm in size, with clear borders. The remaining lung fields were clear, with no abnormal density or hypermetabolic shadows. The mediastinum was centered, and the trachea and bronchi were patent. A round soft tissue density nodule on the right side of the 5th thoracic vertebra with slightly elevated metabolism, SUVmax 1.7, size about 21mm× 18mm, and still clear borders. There were no abnormally enlarged or hypermetabolic lymph nodes in the mediastinum. The cardiac shadow was not large, the size and morphology of each atrium was in the normal range, the pericardial thickness was normal, no signs of pericardial effusion were seen, and there were no abnormalities in the ascending aorta and the branches of the major vessels. No abnormalities were seen in the ascending aorta and major vascular branches. Calcified shadows were seen in the coronary arteries.

Abdomen: no abnormalities in the soft tissue and bony structure of the abdominal wall, normal density and metabolism. There is no fluid accumulation in the abdominal cavity. Gastric filling was acceptable, the gastric wall was not thick, there were no wall nodules, and there were no abnormalities in the metabolism of the gastric wall. There was no abnormality in the morphology, location and metabolic distribution of the liver, with smooth margins and normal lobe proportions; the S8 segment of the liver was a class of rounded low-density shadows, with no metabolic abnormality, and the size was about 18mm× 15mm, with unclear borders. No abnormal density shadow was seen in the remaining liver parenchyma. After choledochal stent placement, the intra- and extrahepatic bile ducts were dilated and pneumatized. The gallbladder was not enlarged, the wall was not thick, and no stones were seen in the gallbladder. The head and neck of the pancreas were thickened and a nodular hypermetabolic shadow was seen with SUVmax 3.7 (early) and 3.9 (delayed), slightly hypodense, measuring about 24mm× 17mm, with unclear borders and poor demarcation from the portal vein. The remaining pancreatic body is caudally atrophic and well defined, with no abnormalities in density or metabolism, the pancreatic ducts are dilated, and the peripancreatic fat interstitium is poorly defined. The spleen was small, with smooth margins, no abnormal density in the splenic parenchyma, and metabolism was normal. There is a round soft tissue density shadow on the medial side of the spleen, with no abnormal metabolism, CT value of about 49 HU, diameter of about 20 mm, and clear boundary. The morphology, density and metabolism of bilateral adrenal glands showed no abnormality. The morphology and density of both kidneys were not abnormal, the bilateral renal pelvis and ureters were not dilated, and no abnormal contrast retention was seen within them. An elliptical nodule appeared to be visible in the hepatogastric space, with slightly elevated metabolism, SUVmax 2.0, size of about 19mm× 13mm, and poorly defined borders. No abnormal enlarged lymph node shadow or hypermetabolic shadow was seen in the retroperitoneum. Part of the colonic bowel showed physiologic uptake shadow.

PELVIC: The pelvic wall has no abnormal soft tissue or bony structure, with normal density and metabolic shadows. There were no signs of fluid accumulation in the pelvis. The bladder was poorly filled with no wall thickening, no abnormal metabolism was seen, and no stones were seen in the bladder. The prostate was of normal size with no abnormal density or metabolic distribution. The fat spaces in the pelvis were clear, and no abnormal enlargement or hypermetabolic lymph nodes were seen in the pelvis or bilateral inguinal region. Some of the rectal intestinal tubes in the pelvis were also seen to show physiologic uptake shadows.

Brief history: the patient had jaundice without any obvious triggers nearly 1 year ago, accompanied by intermittent mild epigastric pain, which was not taken seriously. 7 months ago, jaundice worsened, and the patient was admitted to the Second Provincial Hospital of China, where he was investigated for the four tumors, CA199↑ 276. Abdominal CT showed that the head of the pancreas was pancreatic cancer, with low pancreatico-pancreatic bile duct obstruction secondary to the involvement of the portal and mesenteric superior veins, and accompanied by multiple lymph node metastasis in the adjacent lymph nodes. mr suggested that the tail of the pancreatic body had atrophied significantly, and there was a mass-like T1 slightly high T2 low-signal shadow at the head and neck. Massive T1 slightly high T2 low-signal shadow, the border is not clear, DWI high signal, enhancement is slightly low enhancement changes, lower than the pancreatic head hook clitoral area enhancement, consider pancreatic cancer. Ultrasonography was performed, and a hypoechoic solid mass was seen in the parenchyma of the pancreatic head, with irregular borders, infiltrating the surrounding tissues in the form of a crabfoot pattern, with a size of about 32× 27.9m, and blood flow was not abundant in it, and the mass encroached on the pancreatic section of the common bile duct, and there was no dilatation of the pancreatic duct in the body of the pancreas, and dilatation of the upper section of the common bile duct. The bile duct was stented to relieve cholestasis, and 3-needle puncture pathology was taken and returned: pancreatic head puncture biopsy, pancreatic tissue was seen in the puncture material with a few clumps of ductal epithelial cells, and some of the epithelial cells showed the morphologic manifestation of low-grade sexual heterogeneous hyperplasia. Immunohistochemistry showed CDX-2 (+), CK19 (+ ), CK20 (+ ), CK7 (+ ), Ki-67 (30% of the hot zone), P16 (-), P53 (-), S-100P (+ ), Syn (foci+ ). DNA quantitative cytology reported no DNA ploidy abnormal cells. Biopsy of the descending duodenum: active chronic inflammation of the mucosa, ulcer formation. Postoperative jaundice and abdominal pain were significantly reduced. Nearly 1 month ago, epigastric pain reappeared, aggravated from before, accompanied by back radiating pain. Checked tumor quadruple test, CA199↑ 587, CEA, AFP, CA125 (-).

Purpose of examination: characterization of pancreatic occupancy.

Inspection Observations:

1 Thickening of the head and neck of the pancreas with nodular hypermetabolism, poor demarcation from the portal vein, atrophy of the tail of the pancreatic body, and dilatation of the pancreatic ducts are considered to be pancreatic cancer not excepted, and enhancement examination by MRI is recommended.

A slightly hypermetabolic nodule appears to be seen in the hepatogastric space, and a concomitant MRI is recommended.

2 "After choledochal stent placement" with dilated pneumoperitoneum in the intra- and extrahepatic bile ducts.

A rounded hypodense shadow in the S8 segment of the liver with no metabolic abnormality is recommended for MRI enhancement.

3 Scattered nonmetabolic microscopic solid nodules in both lungs, considered benign.

Confined emphysema of the medial segment of the middle lobe of the right lung.

4 Soft tissue density nodule on the right side of the 5th thoracic vertebrae with slightly increased metabolism and no significant change in size compared to the previous (CT 2023-10-21, the Second Hospital of Hebei Medical University), consider it benign, and recommend regular review.

5 Parasplenic.

follow up

(Pancreatic puncture tissue sent secondarily) Small amount of pancreatic tissue with localized interstitial fibrosis with chronic inflammation.

Thinking:

Okay, I need to write an examination opinion based on the provided PET/CT report and medical history, including a differential diagnosis. Let me start by understanding the key information from the report.

First, the patient is a 58-year-old male with a history of jaundice starting a year ago, intermittent epigastric pain, and elevated CA199 levels. He was previously diagnosed with pancreatic adenocarcinoma based on imaging and biopsy results. The recent PET/CT shows a nodular hypermetabolic lesion in the pancreatic head/neck with SUVmax 3.7-3.9, which is concerning for malignancy. There's also a mention of a lesion in the hepatogastric space and a right lung nodule, but these have lower SUV values.

I need to structure the examination opinion starting with clinical history, followed by imaging findings, and then the differential diagnosis. For differentials, pancreatic adenocarcinoma is the primary consideration given the history and imaging. However, other possibilities like chronic pancreatitis with mass formation, neuroendocrine tumors, or metastasis from another primary should be considered. The lung nodule might be a benign lesion or a metastasis, but the SUV is low, so probably benign. The hepatogastric space lesion could be reactive lymphadenopathy versus metastatic involvement.

I should also mention the elevated CA199 levels and biopsy results supporting adenocarcinoma. The prior stent placement and pathology findings (ductal epithelial changes, immunohistochemistry markers) further support malignancy. However, the biopsy didn't confirm high-grade dysplasia or carcinoma definitively, so there's a slight ambiguity. The differentials should reflect these points.

Need to ensure the language is professional, concise, and covers all relevant aspects. Also, check if there are other incidental findings that need mentioning, like the liver lesion (low density, no hypermetabolism), but likely benign. Finally, recommendations for further steps if any, like follow-up imaging or biopsy.

Answer:

**Examination Opinion and Differential Diagnosis**

**Clinical Summary:**
A 58-year-old male with a history of progressive jaundice, intermittent epigastric pain, and elevated CA199 (276 → 587) over 7 months. Prior imaging (CT/MR) and biopsy suggested pancreatic head adenocarcinoma with portal/mesenteric vein involvement, biliary obstruction, and adjacent lymph node metastasis. Recent PET/CT was performed for further characterization of the pancreatic lesion.

**Key PET/CT Findings:**

1. **Pancreas:**
   - **Head/Neck:** Nodular hypermetabolic lesion (SUVmax 3.7 early, 3.9 delayed), hypodense, poorly demarcated (24 × 17 mm), with invasion into the portal vein.
   - **Body/Tail:** Atrophy, dilated pancreatic duct, and ill-defined peripancreatic fat.
2. **Other Findings:**
   - **Hepatogastric Space:** Soft tissue nodule with mild hypermetabolism (SUVmax 2.0), indeterminate etiology.
   - **Right Lung:** Non-metabolic cystic lesion (10 × 5 mm) and subcentimeter solid nodules, likely benign.
   - **Liver (S8):** Non-metabolic low-density lesion (18 × 15 mm), likely benign.
   - **No distant metastatic lymphadenopathy or visceral lesions.**

**Differential Diagnosis:**

1. **Pancreatic Ductal Adenocarcinoma (Primary Consideration):**
   - Supported by elevated CA199, prior imaging/biopsy findings (mass with portal vein invasion, ductal epithelial hyperplasia on pathology), and hypermetabolic pancreatic lesion.
   - Immunohistochemistry (CK19+, CK7+, S-100P+) aligns with pancreaticobiliary origin.
2. **Chronic Pancreatitis with Mass Formation:**
   - Atrophy and ductal dilation could suggest chronic inflammation. However, hypermetabolism and CA199 elevation favor malignancy.
3. **Pancreatic Neuroendocrine Tumor (PNET):**
   - Less likely given SUVmax <4 and absence of typical imaging features (e.g., hypervascularity). Focal Syn+ on biopsy is nonspecific.
4. **Metastatic Disease (Secondary Lesion):**
   - Unlikely given no hypermetabolic distant lesions. Hepatogastric nodule may represent reactive lymphadenopathy vs. local spread.
5. **Lung Lesions:**
   - Subcentimeter non-metabolic nodules and cystic lesion likely inflammatory or benign (e.g., granulomas).

**Conclusion:**
The findings strongly suggest **pancreatic adenocarcinoma** with local invasion (portal vein) and no definitive evidence of distant metastasis. Incidental benign lesions noted in the liver and lungs. The hepatogastric nodule requires close monitoring to exclude metastasis.

**Recommendations:**

1. Multidisciplinary review for staging and treatment planning (surgical resectability vs. palliative chemotherapy/radiotherapy).
2. Biopsy of the hepatogastric nodule if clinically indicated.
3. Serial CA199 monitoring and follow-up imaging to assess progression.

R5 Provide primary and differential diagnosis based on the report with clinical history.

**Gender**:male **Age**:69

**Inspecti on items:**PET/CT (body including head).

**check on medication:** ^18^ Fluoro-deoxyglucose (^18^F -FDG)

**clinical diagnosi:** lung cancer

**Inspection equipment:** GE Discovery Elite PET/CT

Patient preparation, methods of examination and what is seen on examination:

Fasting blood glucose 5.2 mmol/L, IV 18F-FDG followed by sedentary rest. Oral 1.2% iohexol 1000 ml filled the gastrointestinal tract. Seventy minutes after injection supine position arms raised above the head, body (from the base of the skull to the mid-thigh)+ Head PET/CT: Head PET examination 1× 108 counts, 1 bed. Body PET acquisition 7 beds, 2 min/bed. Delayed PET/CT of the chest 140 minutes after drug injection: 2 beds acquired, 2 min 30 sec/bed. 3D-TOF acquisition, OSEM reconstruction, CT attenuation correction; low-dose CT scanning on the same machine, layer thickness 3.3 mm. fusion imaging of PET, CT.

Head: There were no abnormalities in the metabolism of the soft tissue of the scalp and the bone structure of the skull. Bilateral cerebral hemispheres were symmetrical, with no abnormalities in metabolism and density of the gray and white matter areas of each lobe, clear visualization of the basal ganglia, normal ventricles and cerebral pools, and centered midline structures. The cerebellar hemispheres were symmetrical, with no abnormalities in density and metabolism.

Nasopharynx and neck: No metabolic or structural abnormalities in the sinuses, pharynx and parapharyngeal space. There were no abnormalities in the metabolism and structure of the larynx. Metabolism and density of bilateral submandibular gland and parotid gland were not abnormal. The left lobe of the thyroid gland showed a round, low-density nodule with no metabolic abnormality, measuring about 11 mm in diameter, with clear borders; the right lobe of the thyroid gland showed no abnormal density or metabolic shadows. The metabolism of the neck and bilateral supraclavicular region was not abnormal, and no abnormal metabolism or enlarged lymph nodes were seen.

Chest: thoracic symmetry, small patchy high-density shadow of the 12th thoracic vertebra, no metabolic abnormality; no abnormal density and high metabolic shadow of the remaining chest wall soft tissues and bony structures; limited thickening of the pleura bilaterally, no metabolic abnormality. There was no pleural effusion bilaterally. A slightly hypermetabolic irregular soft tissue nodule in the anterior bronchial segment of the upper lobe of the right lung, SUVmax 4.0 (early) 5.6 (delayed), size about 15mm× 11mm× 11mm, with a burr sign at the edge, the lesion border was relatively straight and the metabolism of the surrounding nodules was not abnormal, the diameters of the surrounding nodules were all less than 3mm; scattered scattered non-metabolic nodules could be seen in the lungs, with the larger one being located in the apical segment of the right lung, diameter about 5mm. The larger one was located in the upper lobe of the right lung, with a diameter of about 5 mm and clear borders. There were scattered punctate calcified shadows in both lungs, with no metabolic abnormality. The mediastinum was centered, the trachea and bronchi were patent, and there were multiple small hypermetabolic lymph node shadows in the mediastinal regions 4R/L, 5, 7, 8, and the hilum of both lungs, with a SUVmax of 8.8, and the larger one with a short diameter of about 12 mm, all of which were slightly higher in density, and some of which were seen to be calcified, with a poorly defined border. No abnormal metabolic lymph nodes were seen. The cardiac shadow was not large, the size and morphology of the atrial chambers were in the normal range, the pericardial thickness was normal, there was no pericardial effusion, and there were no abnormalities in the ascending aorta and the branches of the major vessels. There was no calcification in the coronary artery tract.

Abdomen: no abnormal density or hypermetabolic shadows were seen in the soft tissue and bony structures of the abdominal wall, and no fluid accumulation was seen in the abdominal cavity. Gastric filling was acceptable, the gastric wall was not thick, and the metabolism of the gastric wall was normal. The liver had no abnormal morphology, location and metabolism, with smooth margins and normal lobe proportions; there were multiple non-metabolic hypodense shadows in the liver parenchyma, the larger of which was located in the S4 segment of the liver, about 13 mm× 11 mm, with an average CT value of about 6 HU, and the borders were still clear. There was no dilatation of intrahepatic bile ducts, and the hepatic portal structure was normal. The gallbladder was normal in morphology, the wall was not thick, no stone shadow was seen in the gallbladder, and there was no dilatation of the common bile duct. The pancreas was not abnormal in morphology, location and metabolism, with clear contours, no abnormal density shadows in the pancreatic parenchyma, no dilatation of the pancreatic ducts, and clear peripancreatic fat interstitium. The spleen was not large, with smooth margins, and no abnormal density or hypermetabolic shadows were seen in the splenic parenchyma. The metabolism of the right adrenal gland was uniformly increased, SUVmax 3.7, with no abnormality in morphology and density; the body of the left adrenal gland was slightly thickened with a slightly high metabolism, SUVmax 2.8, with a diameter of about 9 mm, and the border was not clear. The morphology and density of both kidneys were not abnormal, and there was no dilatation of the bilateral renal pelvis and ureter, and no abnormal contrast material retention was seen in them. No abnormal enlarged lymph nodes or hypermetabolic shadows were seen in the retroperitoneum. Some of the intestines in the abdominal cavity physiologically ingested the contrast medium.

Pelvis: left acetabular flaky hyperdense shadow with no metabolic abnormality; no abnormal density or hypermetabolic shadow in the remaining pelvic wall soft tissue and bony structures. There was no fluid accumulation in the pelvis. Bladder filling was fair, no thickening of the bladder wall was seen, and no positive stone shadow was seen in the bladder. The prostate is not enlarged and there are no abnormalities in its internal density or metabolism. The pelvic fat spaces were clear, and there were no abnormal enlarged lymph node shadows or hypermetabolic shadows in the pelvis or bilateral inguinal region.

Brief history: The patient was examined 2 weeks ago with chest CT suggesting a space-occupying lesion in the upper lobe of the right lung near the hilum, not excepting malignancy, and the patient was not unwell.

Purpose of examination: characterization of right lung occupancy.

Inspection Observations:

1 Slightly hypermetabolic parabronchial nodule in the anterior segment of the upper lobe of the right lung, not excluding malignant lesions, close follow-up observation is recommended;

Multiple non-metabolic micronodules in both lungs, tentatively considered benign, follow-up recommended;

Multiple hypermetabolic lymph nodes in the mediastinum and bilateral hilar lungs are considered likely to be chronic inflammatory lymph nodes.

2 Metabolism-free low-density nodule in the left lobe of the thyroid gland, in conjunction with thyroid ultrasound and laboratory tests;

Bilateral limited pleural thickening, consider inflammation; scattered calcified foci in both lungs; hepatic cysts.

3 Uniformly increased metabolism in the right adrenal gland is considered a high probability of physiologic uptake;

Slight thickening of the body of the left adrenal gland with slight hypermetabolism is considered a possible adenoma, and follow-up is recommended;

No metabolic high-density lesions in the 12th thoracic vertebrae or left acetabulum, considered benign, bone islands?

FOLLOW-UP: Thoracoscopic mid-rotation open thoracotomy of the upper lobe of the right lung+ pleural adhesion release was performed. Postoperative pathology return: (right lung lesion tissue) Chronic inflammation of lung tissue.

Thinking:

Okay, I need to create an examination opinion based on this PET/CT report and the medical history provided. Let me start by understanding the key points from the report.

The patient is a 69-year-old male with a clinical diagnosis of lung cancer. The PET/CT was done to characterize a right lung lesion found on a previous CT. The main findings are in the chest: there's a slightly hypermetabolic irregular soft tissue nodule in the right upper lobe near the bronchus, with SUVmax increasing from 4.0 to 5.6 on delayed imaging. The nodule has a burr sign and is surrounded by small nodules. There are also multiple hypermetabolic lymph nodes in the mediastinal and hilar regions, with a SUVmax of 8.8. The thyroid has a low-density nodule without hypermetabolism, and the adrenal glands show some metabolic activity.

First, the differential diagnosis for the lung lesion. The primary concern is malignancy, given the history and increased SUV. However, infections like tuberculosis or fungal infections can also cause hypermetabolic lesions. The presence of calcified lymph nodes might suggest a chronic process, but the new hypermetabolic nodes are concerning for metastasis.

The thyroid nodule is low density but not hypermetabolic, which might be benign, like a cyst or adenoma. The adrenal glands: right has increased metabolism, which could be metastasis, but adrenal adenomas are also common. The left adrenal thickening with mild hypermetabolism might be hyperplasia or early metastasis.

The liver has hypodense lesions without hypermetabolism, possibly cysts or hemangiomas. The previous chest CT suggested a possible malignancy, so the current findings support that but need to rule out other possibilities.

I should structure the examination opinion to include the main findings, differential diagnoses for each significant finding, and recommendations. Make sure to mention the need for biopsy for the lung lesion and lymph nodes, possible further imaging or tests for adrenal and thyroid nodules, and correlation with clinical symptoms and lab results.

Answer:

**Examination Opinion and Differential Diagnosis**

**Patient Summary:**
A 69-year-old male with a history of suspected lung malignancy on prior chest CT underwent PET/CT for further characterization of a right upper lobe lesion. Key findings include:

1. **Right Lung Lesion:** Irregular soft tissue nodule (15 mm × 11 mm × 11 mm) in the anterior segment of the right upper lobe, adjacent to the bronchus. SUVmax increased from 4.0 (early) to 5.6 (delayed), with spiculated margins and surrounding subcentimeter nodules.
2. **Lymph Nodes:** Multiple hypermetabolic mediastinal/hilar lymph nodes (regions 4R/L, 5, 7, 8), largest short diameter 12 mm, SUVmax 8.8.
3. **Thyroid:** Left lobe 11 mm low-density nodule without hypermetabolism.
4. **Adrenals:** Right adrenal uniform hypermetabolism (SUVmax 3.7); left adrenal mild thickening with subtle hypermetabolism (SUVmax 2.8).
5. **Liver:** Non-metabolic hypodense lesions (largest 13 mm × 11 mm), likely benign.

**Differential Diagnosis:**

1. **Right Lung Nodule and Lymph Nodes:**
   - **Primary Lung Malignancy (e.g., NSCLC):** Most likely given hypermetabolism, spiculation, and associated hypermetabolic lymph nodes. Delayed SUV increase supports malignancy.
   - **Infectious/Inflammatory Process (e.g., tuberculosis, fungal infection):** Less likely due to lack of cavitation or calcification in the primary lesion, but possible given scattered lung calcifications.
   - **Metastatic Disease:** Possible if extrathoracic primary exists, though clinical history favors lung origin.
2. **Mediastinal/Hilar Lymph Nodes:**
   - **Metastatic Involvement:** Likely given high SUVmax (8.8) and correlation with the primary lung lesion.
   - **Reactive Hyperplasia:** Less likely due to SUV >2.5, but possible in chronic inflammation.
3. **Thyroid Nodule:**
   - **Benign Cyst/Adenoma:** Favored due to lack of hypermetabolism.
   - **Follicular Neoplasm:** Less likely but requires ultrasound/FNA for confirmation.
4. **Adrenal Lesions:**
   - **Metastasis:** Right adrenal SUVmax 3.7 raises suspicion; however, adrenal adenoma remains possible.
   - **Adrenal Hyperplasia/Non-functional Adenoma:** Left adrenal thickening may represent benign hyperplasia.
5. **Liver Lesions:**
   - **Simple Cysts/Hemangiomas:** Likely given non-metabolic hypodensity and clear borders.

**Recommendations:**

1. **Tissue Diagnosis:**
   - CT-guided biopsy of the right lung nodule and largest mediastinal lymph node (e.g., EBUS-TBNA) to confirm malignancy.
2. **Adrenal Evaluation:**
   - Dedicated adrenal protocol CT or MRI to characterize right adrenal lesion.
3. **Thyroid Assessment:**
   - Thyroid ultrasound with FNA if nodule meets TI-RADS criteria.
4. **Staging:**
   - Brain MRI (if not performed) to exclude occult metastases.
5. **Follow-up:**
   - Repeat PET/CT post-biopsy if malignancy confirmed, to guide treatment planning.

**Conclusion:**
Findings are highly suspicious for primary lung malignancy (NSCLC) with regional nodal metastasis. Further histopathological confirmation is critical. Adrenal and thyroid lesions require additional evaluation to exclude synchronous pathology.

R6 Provide primary and differential diagnosis based on the report with clinical history.

**Gender**:male **Age**:84

**Inspecti on items:**PET/CT (body including head).

**check on medication:** ^18^ Fluoro-deoxyglucose (^18^F -FDG)

**clinical diagnosi:** splenic occupation (medicine)

**Inspection equipment:** GE Discovery Elite PET/CT

Patient preparation, method of examination and what is seen on examination:

Fasting blood glucose: 5.3 mmol/L, IV 18F-FDG followed by sedation. Oral 1.2% iohexol 1000 ml filled the gastrointestinal tract. 60 minutes after the injection supine position arms up holding the head, perform body (from the base of the skull to the middle of the thighs)+ Head PET/CT: Head PET examination 1× 108 counts, 1 bed. Body PET acquisition 7 beds, 2 min/bed, 3D-TOF acquisition, OSEM reconstruction, CT attenuation correction; low-dose CT scanning on the same machine, layer thickness 3.3 mm. fusion imaging of PET, CT.

Skeleton and spleen: metabolic increase of bone was seen in thoracic 5 and 7 vertebrae, lumbar 2 vertebrae, lumbar 4-5 vertebrae, and right sciatic bone, and metabolic increase of thoracic 5 and right sciatic bone was more obvious, SUVmax 8.7, and the corresponding parts of the bone were increased in different degrees of bone density, and no bone damage was seen. An irregular hypermetabolic occupancy in the splenic parenchyma with increased metabolism at the margins, SUVmax 8.7, about 58mm× 48mm× 45mm, with a non-metabolic hypodense shadow in the center and clear borders.

Head and neck: There were no abnormalities in the soft tissue structure and metabolism of the head and neck. The morphology of bilateral cerebral hemispheres was as normal, metabolic distribution was symmetrical, metabolism and density of cerebral gray-white matter areas in each lobe were not abnormal, basal ganglia were clear, ventricles and cerebral pools were widened and deepened, and the midline structure was centered. The cerebellar hemispheres on both sides were symmetrical, with no abnormalities in density and metabolism. The metabolism and structure of the nose, pharynx and parapharyngeal space were not abnormal. Metabolism and density of submandibular gland and parotid gland on both sides were normal. The vocal cords were symmetrical bilaterally, and there were no abnormalities in the metabolism and structure of the larynx. No abnormal density and metabolic shadows were seen in the bilobed thyroid glands. There were no metabolic abnormalities in the neck and bilateral supraclavicular region, and no abnormal metabolism or enlarged lymph nodes were seen.

Thorax: The thorax is symmetrical, with no abnormalities in the soft tissue structures of the chest wall, and normal density and metabolism. The pleura was smooth bilaterally and no pleural effusion was seen. Scattered multiple cystic hypodense translucent shadows in both lungs, prominent in the lower lobe of the left lung, small patchy subpleural ground-glass hyperdense shadows in the apical segment of the upper lobe of the right lung, thickening of the interlobular septum, and metabolism were not seen. No abnormal density or hypermetabolic shadows were seen in the remaining lungs. The mediastinum was centered, trachea and bronchus were patent, and small slightly hypermetabolic lymph nodes were seen in both pulmonary hilums, SUVmax 3.8 (right) and 4.5 (left), and no abnormally enlarged lymph node shadows or hypermetabolic shadows were seen in the rest of the mediastinum. The cardiac shadow was not large, the size and morphology of each atrium was in the normal range, the pericardial thickness was normal, no signs of pericardial effusion were seen, and there were no abnormalities in the ascending aorta and the branches of the major vessels. There were no abnormalities in the ascending aorta and major vascular branches. Multiple calcified shadows were seen in the coronary arteries.

Abdomen: no abnormalities in the soft tissue structure of the abdominal wall, normal density and metabolism. There is no fluid accumulation in the abdominal cavity. Gastric filling was acceptable, the gastric wall was not thick, there were no wall nodules, and there were no abnormalities in the metabolism of the gastric wall. The morphology, location and metabolic distribution of the liver were not abnormal, with smooth margins and normal lobe proportions; there were multiple non-metabolic cystic low-density shadows in the liver, the larger one was located in the S4 segment, with a diameter of about 20 mm and a CT value of 8 HU; there was no dilatation of the intrahepatic bile ducts, and the structure of the hepatic hilum was normal. The gallbladder was not enlarged, the wall was not thick, no positive stones were seen in the gallbladder, and there was no dilatation of the extrahepatic bile ducts or common bile duct. The pancreas had no abnormalities in location, morphology and metabolism, with clear contours, uniform density of pancreatic parenchyma, no abnormal density, no dilatation of pancreatic ducts, and clear peripancreatic fat interstitium. The morphology, density and metabolism of bilateral adrenal glands showed no abnormality. The morphology and density of both kidneys were not abnormal, and there was no dilatation of the bilateral renal pelvis and ureter, and no abnormal contrast material retention was seen in them. No abnormal enlarged lymph nodes or hypermetabolic shadows were seen in the retroperitoneum. Some of the colonic bowel showed physiologic uptake shadows.

PELVIC: The pelvic wall has no abnormal soft tissue structure, with normal density and metabolic shadows. There were no signs of fluid accumulation in the pelvis. The bladder was poorly filled with no wall thickening, no abnormal metabolism was seen, and no positive stones were seen in the bladder. The prostate was of normal size with no abnormal density or metabolic distribution. The pelvic fat spaces were clear, and no abnormally enlarged or hypermetabolic lymph nodes were seen in the pelvis or bilateral inguinal region. Some rectal intestinal tubes in the pelvis were also seen to show physiologic uptake shadows.

Brief history: physical examination revealed splenic occupancy for 2 weeks, liver, gallbladder and spleen MRI scan+ enhancement scan suggests: splenic intrasplenic occupying lesion, consider malignant lesion is likely, not except lymphoma. Multiple hepatic cysts. Bone marrow classification shows a slightly higher percentage of red lineage, please combine with clinical. Flow immunophenotyping: impression: decreased percentage of lymphocytes, increased percentage of neutrophils, no monoclonal abnormal B lymphocytes. Tumor markers are approximately normal.

Purpose of examination: characterization of splenic occupancy.

Inspection Observations:

1 Intrasplenic circumferential hypermetabolic occupations, thoracic 5 and 7 vertebrae, lumbar 2 vertebrae, lumbar 4-5 vertebrae, and localized hypermetabolism of the right sciatic bone with increased bone mineral density, all of the above are considered malignant lesions, with a high likelihood of a splenic primary, and biopsy pathology is recommended.

2 Bilateral pulmonary emphysema. Minor subpleural inflammation in the apical segment of the upper lobe of the right lung. Inflammatory lymph nodes in both hilums.

3 Brain atrophy. Coronary artery calcification. Multiple cysts in the liver.

**Follow-up:** epithelioid angiosarcoma

Thinking:

Alright, I need to write an examination opinion based on the provided PET/CT report and the patient's medical history, including a differential diagnosis. Let me start by understanding the key findings and the patient's background.

The patient is an 84-year-old male with a splenic occupying lesion found during a physical examination two weeks prior. Previous MRI scans suggested a malignant lesion, possibly lymphoma, and there are multiple hepatic cysts. The PET/CT report highlights increased metabolic activity in several vertebrae and the right sciatic bone, as well as an irregular hypermetabolic lesion in the spleen with SUVmax 8.7. There's also mention of small hypermetabolic lymph nodes in the hilar regions of the lungs. Other areas like the head, neck, thorax, abdomen, and pelvis show no significant abnormalities except for some physiological uptake and non-metabolic cysts.

First, I should consider the splenic lesion. The high SUVmax suggests malignancy. Differential diagnoses for splenic masses include lymphoma, metastatic cancer, and less likely benign tumors like hemangioma. Given the MRI suggestion of malignancy and possible lymphoma, lymphoma is a strong contender. However, metastatic disease from another primary site should also be considered, especially with bone lesions. The bone lesions showing increased metabolism and density changes could indicate metastases or a primary bone tumor, but given the patient's age, metastases are more likely.

The hepatic cysts are noted as non-metabolic, which aligns with simple cysts, so they might be benign. The small hypermetabolic hilar lymph nodes (SUVmax 3.8 and 4.5) are slightly concerning but not highly active. This could be reactive or related to the primary splenic lesion.

The patient’s age and the presence of multiple sites with metabolic activity (spleen and bones) make me think of disseminated malignancy. Lymphoma, especially non-Hodgkin’s lymphoma, often involves the spleen and can present with multiple lymph node regions and bone involvement. Metastatic carcinoma from another primary site (like gastrointestinal, lung, etc.) should also be ruled out. However, the primary seems to be the spleen here. Primary splenic lymphoma is rare but possible.

Other considerations include infectious or inflammatory processes, but the lack of significant inflammatory markers and the focal nature of the lesions make this less likely. Myeloproliferative disorders could also present with splenomegaly and bone changes, but the PET findings are more indicative of a solid tumor.

In summary, the differential diagnosis should prioritize lymphoma, metastatic carcinoma, and possibly primary splenic malignancy. Further diagnostic steps might include biopsy of the splenic lesion, bone marrow biopsy, and possibly imaging to identify a primary tumor elsewhere if metastatic disease is suspected.

Answer:

**Examination Opinion and Differential Diagnosis**

**Patient Summary:**
An 84-year-old male presented with a splenic occupying lesion detected during physical examination. Prior MRI and clinical investigations raised suspicion for malignancy (likely lymphoma), alongside multiple hepatic cysts. PET/CT findings demonstrate:

1. **Splenic lesion:** Irregular hypermetabolic mass (58×48×45 mm, SUVmax 8.7) with central necrosis.
2. **Bone involvement:** Increased metabolic activity in thoracic (T5, T7), lumbar (L2, L4-5), and right sciatic bone, with SUVmax 8.7 in T5 and sciatic bone.
3. **Lymph nodes:** Mildly hypermetabolic bilateral hilar lymph nodes (SUVmax 3.8–4.5).
4. **Other findings:** Multiple hepatic cysts (non-metabolic), physiologic bowel uptake, and no significant abnormalities in head/neck, thorax, abdomen, or pelvis.

**Key Observations and Interpretation:**

1. **Splenic Lesion:**
   - High FDG avidity (SUVmax 8.7) strongly suggests malignancy.
   - Central necrosis and peripheral hypermetabolism align with aggressive neoplasms.
   - **Primary differentials:**
     - **Lymphoma (e.g., non-Hodgkin’s lymphoma):** Most likely given clinical suspicion, splenic involvement, and multifocal bone lesions.
     - **Metastatic carcinoma:** Possible if a primary tumor elsewhere (e.g., gastrointestinal, lung) metastasized to spleen and bones.
     - **Primary splenic malignancy (rare):** Angiosarcoma or sarcoma, though less common.
2. **Bone Lesions:**
   - Multifocal lytic/sclerotic changes with high FDG uptake suggest metastases or hematologic malignancy (e.g., lymphoma).
   - **Differential:** Metastatic disease vs. primary bone malignancy (unlikely given age and distribution).
3. **Hilar Lymph Nodes:**
   - Mild hypermetabolism (SUVmax <5) may represent reactive changes or early involvement by lymphoma/metastases.
4. **Hepatic Cysts:**
   - Non-metabolic, likely benign simple cysts.

**Differential Diagnosis:**

1. **Lymphoma (High Probability):**
   - Splenic mass, multifocal bone lesions, and hilar lymphadenopathy are classic for disseminated lymphoma (e.g., diffuse large B-cell lymphoma).
   - Further evaluation: Splenic or bone biopsy, flow cytometry, and bone marrow examination.
2. **Metastatic Carcinoma:**
   - Requires exclusion of primary malignancy (e.g., lung, prostate, or gastrointestinal).
   - Workup: Tumor markers (PSA, CEA), endoscopy, and imaging (e.g., colonoscopy, chest CT).
3. **Primary Splenic Malignancy (Rare):**
   - Angiosarcoma or other sarcomas, but typically more heterogeneous on imaging.
4. **Infectious/Inflammatory Process (Less Likely):**
   - Granulomatous disease (e.g., tuberculosis) or abscesses, but lack of systemic symptoms and necrotic splenic lesion favor malignancy.

**Recommendations:**

1. **Tissue Diagnosis:**
   - Ultrasound/CT-guided biopsy of the splenic lesion for histopathology and immunohistochemistry.
   - Bone biopsy (e.g., thoracic vertebra) if accessible.
2. **Staging Workup:**
   - Bone marrow biopsy to assess for hematologic involvement.
   - Contrast-enhanced CT chest/abdomen/pelvis to identify occult primary tumors.
3. **Oncologic Referral:**
   - Multidisciplinary review for lymphoma vs. metastatic carcinoma management.
4. **Follow-Up Imaging:**
   - Repeat PET/CT post-biopsy to assess treatment response if malignancy confirmed.

**Conclusion:**
The findings are highly concerning for a malignant process, with lymphoma or metastatic carcinoma as leading diagnoses. Urgent histopathologic confirmation is critical to guide therapy.

R7 Provide primary and differential diagnosis based on the report with clinical history.

**Gender**:male **Age**:24

**Inspecti on items:**PET/CT (body without head).

**check on medication:** ^18^ Fluoro-deoxyglucose (^18^F -FDG)

**clinical diagnosi:** 1. Fever to be investigated; acute suppurative tonsillitis(?) ; infectious mononucleosis? ; Lymphoma(?) ;2. Sinus tachycardia

**Inspection equipment:** GE Discovery Elite PET/CT

Patient preparation, method of examination and what is seen on examination:

Fasting blood glucose: 5.1 mmol/L, IV 18F-FDG followed by sedentary rest. Oral 1.2% iohexol 1000 ml filled the gastrointestinal tract. Fifty-three minutes after injection supine position arms up to hold the head, whole body (from the top of the head to the soles of the feet) PET/CT: Head PET examination 1× 108 counts, 1 bed. Body+ PET acquisition of extremities 12 beds, 2 min/bed, 3D-TOF acquisition, OSEM reconstruction, CT attenuation correction; low-dose CT scanning on the same machine, layer thickness 3.3 mm. PET, CT fusion imaging.

Lymphatic system: pharyngeal lymphatic ring, bilateral cervical region II~ V, bilateral supraclavicular region, bilateral axillae, mediastinal regions 2R, 4L, 4R, 6, 7, bilateral pulmonary hilar, right 5th thoracic rib joint medial, lower paraesophageal, hepatic hilar region, abdominal aorta paracentesis, retroperitoneal mesenteric region, bilateral iliac vessels paracentesis, bilateral inguinal lymph nodes of hypermetabolism, SUVmax 2.0~17.4, borders are still clear, and the large ones are located in the Left cervical region II, size about 33mm× 26mm, uniform density, mean CT value 52HU.Spleen was normal in location and density, increased in size with diffuse hypermetabolism, SUVmax 4.9, occupying about 11 rib units, mean CT value 51HU.

Head and neck: There were no abnormalities in the soft tissue structure and metabolism of the head and neck. Bilateral cerebral hemispheres had normal morphology, symmetrical metabolic distribution, no abnormalities in metabolism and density in the gray and white matter areas of each lobe of the brain, clear visualization of the basal ganglia, normal ventricles and cerebral pools, and centered midline structure. The cerebellar hemispheres on both sides were symmetrical, with no abnormalities in density and metabolism. There were no abnormalities in the metabolism and structure of the sinuses, pharynx and parapharyngeal space. Metabolism and density of submandibular gland and parotid gland on both sides were not abnormal. The vocal cords were symmetrical bilaterally, and there were no abnormalities in the metabolism and structure of the larynx. The thyroid gland was symmetrical in both lobes, with uniform distribution of radioactivity and density in the parenchyma.

Thorax: The thorax is symmetrical, and there is no abnormality in the morphology, density or metabolism of the soft tissues of the chest wall. Bilateral pleura was smooth, no pleural effusion shadow was seen. The lower lobe of the left lung had multiple striated cords, with no obvious abnormalities in metabolism; the remaining bilateral lung fields were well textured, with no abnormalities in density or metabolism. The mediastinum was centered, the trachea and bronchi were patent, the heart shadow was not large, the size and morphology of each atrium was in the normal range, the pericardial thickness was normal, there was no pericardial effusion, and there was no abnormality of the ascending aorta and branches of the major blood vessels. No abnormal calcification was seen in the coronary artery traveled area.

Abdomen: no abnormalities in soft tissue density or metabolism of the abdominal wall, no signs of fluid accumulation in the abdominal cavity. The stomach was full and the metabolism of the gastric wall was not abnormal. The location and radiological distribution of the liver were not abnormal, the margins were smooth, the liver was large, and the proportion of lobes was normal; the density in the liver parenchyma was homogeneous, and there was no dilatation of the intrahepatic bile ducts, and the structure of the hepatic hilum was normal. The gallbladder was not enlarged, the wall was thickened, the metabolism was not abnormal, the maximum thickness was about 8mm, no positive stone shadow was seen in the gallbladder, and the common bile duct was not dilated. The pancreas did not show any abnormality in morphology, location and radiological distribution, with clear contours, no abnormal density shadows in the pancreatic parenchyma, no dilatation of the pancreatic ducts, and clear peripancreatic fat interstitial space. The morphology, density and metabolism of bilateral adrenal glands showed no abnormality. The morphology and density of both kidneys were not abnormal, and the bilateral renal pelvis and ureters were not dilated, with no abnormal contrast material retention in them. Some of the colonic intestinal tubes showed physiologic uptake shadows.

PELVIC: There were no abnormalities in the density and metabolism of the soft tissues of the pelvic wall. No signs of fluid accumulation in the pelvis. There were no positive stone shadows in the poorly filled bladder or bladder. There are no abnormalities in density and metabolism in the prostate area. The fat interstitial space in the pelvis is clear, and some rectal bowel in the pelvis is also seen as a physiologic ingestive shadow.

Bone: Diffuse metabolic increase in bilateral proximal humerus, bilateral proximal femur, multiple vertebrae of the spine, and pelvis, SUVmax 4.2, with no obvious abnormality in bone morphology and density. There were no obvious abnormalities in bone structure and metabolism in the rest of the body.

Mediastinal blood pool SUVmax 1.5, hepatic blood pool 3.5.

Brief history: 2 weeks ago, the patient suffered from cold, fever, the highest temperature reached 38.6℃ , with profuse sweating, nausea and vomiting, vomit for stomach contents, oral amoxicillin, the effect is not good. 10 days ago, sore throat, the local clinic oral medication (unknown), temperature fluctuations in the 37.3℃ -37.5 ℃; 9 days ago, the patient worsened the sore throat, the local town health center to check the blood and chest CT (not reported) suggests that leukocytes are elevated. ) suggested elevated leukocytes. Symptomatic treatment effect is not good. Xingtang County Hospital blood routine: white blood cell count: 27.1× 10^9/L; lymphocyte count 19.9× 10^9/L; hemoglobin: 133g/L; platelet count: 136× 10^9/L; parotid ultrasonography: hypoechoic in the parotid gland on the left side (consider lymph nodes), and left cervical lymph nodes are enlarged. Chest CT: striated shadow in the lower lobes of both lungs, enlarged lymph nodes in the axilla and mediastinum on both sides. She was admitted to the outpatient clinic with "multiple enlarged lymph nodes (cause to be investigated)". （Bone marrow biopsy: Bone marrow proliferation was approximately normal (70%-80%), with a reduced granulocyte to red ratio, granulocyte lineage cells of all stages were seen, with a predominance of cells in the middle and lower stages, and eosinophils scattered, and erythroid lineage cells of all stages were seen, with a predominance of cells in the middle and later stages, and megakaryocytes were not uncommon, and were distributed in a scattered manner. The proportion of lymphocytes is increased, combined with immunohistochemical staining CD3 positive T lymphocytes increased significantly, tendency of T lymphoid tissue proliferative lesions (to be diagnosed by molecular pathology). Please combine with clinical and other examinations to further determine the diagnosis. Immunohistochemical staining: CD61 (megakaryocytes+ ), CD71 (erythroid cells+ ), MPO (granulocytes+ ), CD34 (individual cells+ ), CD3 (+ ), CD20 (a few cells+ ). Special stains: reticular fibers (grade 0-1), Masson (-), PAS (+ ).

Purpose of examination: fever to be investigated.

Inspection Observations:

1 Multiple regional hypermetabolic lymph nodes above and below the diaphragm, splenomegaly with hypermetabolism, diffuse hypermetabolism of the bone marrow, and hepatomegaly, above all, the primary consideration is a reactive hyperplastic lesion, hematological tumors are not excluded, please combine with pathology.

2 Striations in the lower lobe of the left lung. Edema of the gallbladder wall.

Follow-up:

(right cervical lymph node) Combined with immunohistochemical staining consistent with an acute T-zone proliferative lesion, consider infectious mononucleosis.

Immunohistochemical staining: CD3 (interfollicular zone+ ), CD20 (follicular zone+ ), CD21 (FDC network+ ), Ki-67 (~70%+), number of CD8-positive cells > number of CD4-positive cells, CD56 (weakly+ ), TIA-1 (+ ), Granzyme B (+ ), CD15 (partially+ ), PAX-5 (follicular zone+ ).

In situ hybridization: EBER (+ ).

Thinking:

Okay, so I need to write an examination opinion based on this PET/CT report and the patient's medical history. Let me start by understanding what's given. The patient is a 24-year-old male with symptoms like fever, sore throat, and swollen lymph nodes. He had a cold two weeks ago, followed by high fever, nausea, vomiting, and then persistent sore throat. Blood tests showed elevated white blood cells and lymphocytes. Imaging found multiple hypermetabolic lymph nodes and an enlarged spleen. The bone marrow biopsy suggests T-cell proliferation.

First, I should list the key findings. The PET/CT shows multiple hypermetabolic lymph nodes with high SUVmax (up to 17.4), which is concerning for malignancy like lymphoma. The spleen is enlarged with increased metabolism, which can also be a sign of lymphoma. However, infectious causes like infectious mononucleosis or acute bacterial infections (like tonsillitis) can also cause lymphadenopathy and splenomegaly. The elevated lymphocytes and history of recent infection might point towards viral infections, but the high SUVmax leans more towards malignancy.

The bone marrow biopsy mentions increased T-lymphocytes and possible T-cell proliferative disorder. Lymphoma, especially T-cell lymphoma, is a possibility. But other differentials like reactive lymphadenopathy due to infection (EBV, CMV) should be considered. The patient didn't respond to antibiotics, which reduces the likelihood of bacterial infection. The prolonged fever and lymphadenopathy despite treatment suggest something more chronic or aggressive.

Differential diagnosis should include:

1. **Lymphoma (especially T-cell type):** Given the hypermetabolic nodes, high SUV, and bone marrow findings.

2. **Infectious Mononucleosis (EBV):** Common in young adults, presents with fever, lymphadenopathy, splenomegaly. However, PET SUV is usually lower unless complicated.

3. **Acute Suppurative Tonsillitis:** Though he didn't respond to amoxicillin, maybe due to resistant bacteria or viral cause.

4. **Other Viral Infections (CMV, HIV):** Could cause similar symptoms but need serology.

5. **Tuberculosis:** Can cause lymphadenopathy and fever, but typically with different imaging features.

6. **Autoimmune Diseases:** Like SLE, but usually with other systemic symptoms.

7. **Reactive Lymphadenopathy:** Due to unresolved infection or other inflammatory processes.

The report also mentions sinus tachycardia, which could be secondary to fever or anemia, but his hemoglobin is normal. The bone marrow shows increased lymphocytes and T-cell proliferation, so lymphoma is high on the list. However, confirmatory tests like lymph node biopsy, flow cytometry, and molecular studies (like TCR rearrangement for T-cell clonality) are needed. Also, EBV serology or PCR would help rule out infectious mononucleosis.

In the examination opinion, I need to summarize the findings, list the differentials with supporting points, and suggest next steps. Make sure to prioritize the most likely diagnoses and recommend appropriate follow-up tests.

Answer:

**Examination Opinion and Differential Diagnosis**

**Summary of Findings:**
A 24-year-old male presented with prolonged fever (max 38.6°C), sore throat, and multiple enlarged lymph nodes (cervical, axillary, mediastinal). PET/CT revealed multifocal hypermetabolic lymph nodes (SUVmax up to 17.4), splenomegaly with diffuse hypermetabolism (SUVmax 4.9), and diffuse skeletal hypermetabolism. Laboratory findings include leukocytosis (27.1×10⁹/L), marked lymphocytosis (19.9×10⁹/L), and bone marrow biopsy showing T-lymphocyte proliferation with CD3+ predominance.

**Key Differential Diagnoses:**

1. **Lymphoma (T-cell subtype):**
   - Supporting evidence: Hypermetabolic lymph nodes (SUVmax >10), splenomegaly, and bone marrow findings (T-cell proliferation, CD3+).
   - High suspicion for aggressive lymphoma (e.g., peripheral T-cell lymphoma) given SUVmax 17.4.
2. **Infectious Mononucleosis (EBV):**
   - Common in young adults with fever, lymphadenopathy, and splenomegaly.
   - Elevated lymphocytes and atypical lymphocytosis may align, but extreme SUVmax values (>10) are atypical for EBV. Requires EBV serology/PCR for exclusion.
3. **Other Viral Infections (e.g., CMV, HIV):**
   - May cause prolonged fever and lymphadenopathy. Serologic testing (CMV IgM/IgG, HIV screening) recommended.
4. **Tuberculosis (TB):**
   - Lymphadenopathy and fever are common, but PET typically shows lower SUVmax and necrotic nodes. Acid-fast bacilli (AFB) testing and interferon-gamma release assay (IGRA) needed.
5. **Reactive Lymphadenopathy:**
   - Secondary to unresolved bacterial/viral infection (e.g., streptococcal tonsillitis, resistant pathogens). Poor response to antibiotics reduces likelihood.
6. **Autoimmune Disorders (e.g., SLE):**
   - Less likely without typical autoantibodies or multisystem involvement.

**Recommendations for Further Evaluation:**

1. **Lymph Node Excisional Biopsy:** Confirm histopathology and perform flow cytometry to assess clonality (TCR rearrangement for T-cell lymphoma).
2. **Infectious Workup:** EBV/CMV PCR, HIV serology, TB testing (sputum AFB, IGRA).
3. **Laboratory Studies:** LDH, β2-microglobulin (prognostic in lymphoma), and autoimmune panels (ANA, anti-dsDNA) if indicated.
4. **Imaging Follow-up:** Repeat CT/MRI if biopsy is delayed; monitor response if treatment initiated.
5. **Hematology/Oncology Consultation:** Urgent referral for suspected lymphoma to guide staging and management.

**Conclusion:**
The findings strongly suggest a malignant process, most likely **T-cell lymphoma**, given the hypermetabolic lymphadenopathy and bone marrow results. Infectious etiologies (e.g., EBV) remain less likely but require exclusion. Immediate histopathologic confirmation is critical to establish a definitive diagnosis and initiate appropriate therapy.

R8 Provide primary and differential diagnosis based on the report with clinical history.

**Gender**:female **Age**:60

**Inspecti on items:**PET/CT (body including head).

**check on medication:** ^18^ Fluoro-deoxyglucose (^18^F -FDG)

**clinical diagnosi:** Malignant melanoma (left parotid gland) (post-op)

**Inspection equipment:** GE Discovery Elite PET/CT

Patient preparation, method of examination and what is seen on examination:

Fasting blood glucose: 5.6 mmol/L, resting after intravenous 18F-FDG injection. Body (from the base of the skull to the middle of the thighs) containing head PET/CT was performed 71 minutes after injection: head PET examination 1× 108 counts, 1 bed, body PET examination, 7 beds, 2 minutes/bed, 3D-TOF acquisition, OSEM reconstruction, CT attenuation correction; low-dose CT scanning on the same machine with layer thickness of 3.3 mm. fusion imaging of PET and CT.

Lymphatic system: extensive lymph nodes with increased metabolism, partially enlarged, locally aggregated into clusters, large ones located in the left groin, size 20mm× 16mm, SUVmax 18.5, were seen in the left supraclavicular region, left axilla, multiple regions of the mediastinum, both pulmonary hilums, hepatic hilums, hepatogastric hiatus, retroperitoneum in the upper and lower abdomen, left and right iliac paravalvular vessels, and left and right groin. the spleen was enlarged, occupying 7 rib cells, with increased metabolism. SUVmax 7.9.

Skeletal: Uneven metabolic elevation was seen in the bone marrow cavity of the lower spine, pelvis and proximal portions of the upper and lower extremities bilaterally, SUVmax 3.0-3.4, no abnormal bone density or bone destruction was seen.

Cranium: There is no abnormality in the metabolism of the scalp soft tissues. Bilateral cerebral hemispheres have normal morphology, symmetrical distribution of visualizer, no abnormalities in metabolism and density of cerebral gray-white matter areas in each lobe, clear visualization of basal ganglia, normal ventricles and cerebral pools, and centered midline structure. The cerebellar hemispheres on both sides were symmetrical, with no abnormalities in density and metabolism. The maxillary sinus and sieve sinus were symmetrical with no thickening of the mucosa and no abnormal metabolism. Metabolism and density of submandibular gland and parotid gland on both sides were not abnormal.

Neck: No abnormal density or metabolic changes were seen in the soft tissues of the neck. The thyroid gland is symmetrical in both lobes, and no foci of increased contrast uptake or abnormal density shadows are seen in the thyroid parenchyma of both lobes. No metabolic or structural abnormalities were seen in the pharynx.

Chest: The thorax is symmetrical, and no abnormal density or hypermetabolic shadows are seen in the soft tissues of the chest wall. A few fluid density shadows were seen in the bilateral thoracic cavity, and metabolism was not significantly increased. Lung fields were clear, with no abnormal density or hypermetabolic shadows. The mediastinum was centered, and the trachea and bronchi were patent. The cardiac shadow was not large, the size and morphology of each atrium was in the normal range, the pericardial thickness was normal, no signs of pericardial effusion were seen, and the ascending main artery and the branches of the major vessels showed no abnormality. There were no abnormal calcified shadows in the coronary arteries.

Abdomen: no abnormal density or hypermetabolic shadows in the soft tissues of the abdominal wall, no signs of fluid accumulation in the abdominal cavity. The stomach was full and the metabolism of the gastric wall was not abnormal. There was no abnormality in the morphology, position and radiological distribution of the liver, with smooth margins and normal lobe proportions; there were no abnormal density shadows in the liver parenchyma, no dilatation of the intrahepatic bile ducts, and normal hepatic portal structure. The gallbladder was not enlarged, the wall was not thick, no positive stone shadow was seen in the gallbladder, and the common bile duct was not dilated. The pancreas did not show any abnormality in morphology, location and radiological distribution, with clear contours, no abnormal density shadow in the pancreatic parenchyma, no dilatation of the pancreatic ducts, and clear peripancreatic fat interstitium. The morphology, density and metabolism of bilateral adrenal glands showed no abnormality. The morphology and density of both kidneys were not abnormal, and the bilateral renal pelvis and ureter were not dilated, with no abnormal contrast material retention in them. Some of the colonic intestinal tubes showed physiologic uptake shadows.

Pelvis: No abnormal density or hypermetabolic shadows were seen in the soft tissues of the pelvic wall. No signs of fluid accumulation in the pelvis. There were no positive stone shadows in the poorly filled bladder or bladder. The uterus is full in shape with localized calcification, no abnormal increase in metabolism is seen, the fat interstitium in the pelvis is clear, and some of the rectal bowel in the pelvis is also seen as a physiologic ingestive shadow.

Brief history: unexplained fever for more than 20 days, fever to be investigated. left parotid malignant melanoma resection 2 years ago.

Inspection Observations:

1 Multiple lymph nodes above and below the diaphragm are metabolically elevated and partially enlarged; lymphoma involvement is considered. Splenomegaly with increased metabolism and localized increased bone metabolism are also considered lymphoma involvement. In summary, Lugano stage: stage IV, left inguinal lymph node excision biopsy is recommended for pathology.

2 A small amount of non-metabolic fluid in the pleural cavity bilaterally.

3 Full uterine morphology with localized calcifications suggests further examination by ultrasound if necessary.

Follow-up pathology: lymphadenitis

Thinking:

Okay, let me try to work through this. The user provided a PET/CT report for a patient named Zhao Huancai, a 60-year-old female with a history of malignant melanoma in the left parotid gland, which was resected two years ago. She now has an unexplained fever for over 20 days. The PET/CT findings show extensive lymph nodes with elevated metabolism in multiple regions, including the left clavicular, axilla, mediastinum, lung hilums, hepatic hilum, retroperitoneum, iliac regions, and groin. The largest node is in the left groin (20x16mm, SUVmax 18.5). The spleen is enlarged with increased metabolism (SUVmax 7.9). Bone marrow shows uneven metabolic elevation but no structural abnormalities. No other significant abnormalities in the brain, neck, chest, abdomen, or pelvis except for some physiological uptake in the colon and rectum.

First, I need to recall what SUVmax values indicate. Higher SUVmax generally suggests higher metabolic activity, often associated with malignancy. The patient's history of melanoma makes recurrence or metastasis a primary concern. Malignant melanoma is known to metastasize to lymph nodes and distant organs. The widespread lymphadenopathy with high SUVmax could indicate metastatic spread. The splenic enlargement and increased metabolism might be due to metastatic involvement or reactive changes from the malignancy.

Differential diagnoses to consider:

1. **Metastatic Malignant Melanoma**: Given the history, this is the most likely. The high SUVmax in lymph nodes and spleen supports this.

2. **Lymphoma**: Can present with widespread lymphadenopathy and splenomegaly. However, the patient's history of melanoma makes this less likely unless there's a secondary malignancy.

3. **Infection/Inflammatory Conditions**: The fever could indicate an infection. However, the PET findings (high SUV) are more suggestive of malignancy than infection unless there's an abscess or granulomatous disease.

4. **Sarcoidosis**: Can cause lymphadenopathy and splenomegaly with increased FDG uptake, but usually with different distribution (e.g., symmetric hilar nodes) and lower SUVmax.

5. **Reactive Lymphadenopathy**: Due to chronic inflammation or other causes, but again, the high SUVmax and history make this less likely.

Other considerations: The bone marrow findings (elevated metabolism without structural changes) could be due to reactive changes, myeloproliferative disorders, or metastatic disease. However, without structural abnormalities, metastatic involvement is less certain. The fever might be paraneoplastic or due to the disease burden itself.

I should also check if there's any mention of other primary cancers, but the report doesn't indicate that. The physiological uptake in the colon/rectum is likely normal. The lack of other organ involvement (brain, liver, etc.) is noted but doesn't rule out metastatic melanoma, which can be variable.

In conclusion, the most probable diagnosis is metastatic recurrence of malignant melanoma given the history and PET findings. However, other possibilities like lymphoma or infection should be considered, and further diagnostic tests (biopsy of the largest lymph node, blood cultures, etc.) might be warranted.

Answer:

**Examination Opinion and Differential Diagnosis**

**Patient Summary:**

A60-year-old female with a history of resected left parotid malignant melanoma (2 years prior), presents with unexplained fever lasting >20 days. PET/CT findings reveal widespread hypermetabolic lymphadenopathy, splenomegaly, and bone marrow metabolic activity without structural abnormalities.

**Key Findings:**

1. **Lymphatic System:**
   - Extensive hypermetabolic lymph nodes (SUVmax 18.5 in left groin) in multiple regions (clavicular, axillary, mediastinal, retroperitoneal, iliac, groin).
   - Splenomegaly (7 rib spaces) with increased metabolism (SUVmax 7.9).
2. **Bone Marrow:**
   - Diffuse metabolic elevation in lower spine, pelvis, and proximal extremities (SUVmax 3.0–3.4), no bone destruction.
3. **Other Systems:**
   - No evidence of recurrence at the primary parotid site or metastases in brain, lungs, liver, or other visceral organs.

**Clinical Interpretation:**
The findings are highly suspicious for **metastatic recurrence of malignant melanoma**, given the patient’s history and the widespread lymphadenopathy with markedly elevated FDG uptake. The splenic involvement and bone marrow activity may represent metastatic spread or paraneoplastic/systemic inflammatory response.

**Differential Diagnosis:**

1. **Metastatic Malignant Melanoma (Most Likely):**
   - Melanoma frequently metastasizes to lymph nodes and visceral organs. High SUVmax (>10) strongly supports malignancy.
2. **Lymphoma (Secondary Consideration):**
   - Widespread lymphadenopathy and splenomegaly could suggest lymphoma, though less likely given melanoma history. Biopsy of the largest node (left groin) is critical to exclude this.
3. **Infection/Inflammatory Process:**
   - Fever and lymphadenopathy may indicate disseminated infection (e.g., tuberculosis) or granulomatous disease. However, the absence of necrosis/low SUVmax makes this less probable.
4. **Sarcoidosis:**
   - May mimic malignancy with hypermetabolic nodes, but typically involves symmetric hilar/mediastinal nodes and has lower SUVmax.
5. **Reactive Lymphadenopathy:**
   - Secondary to chronic inflammation, but unlikely given the high SUVmax and clinical context.

**Recommendations:**

1. **Tissue Diagnosis:** Urgent biopsy of the left groin lymph node (largest, highest SUV) to confirm malignancy and differentiate melanoma recurrence from lymphoma.
2. **Laboratory Workup:**
   - LDH, CBC, inflammatory markers (CRP, ESR), blood cultures, and serum protein electrophoresis to assess for infection or paraneoplastic syndromes.
3. **Bone Marrow Evaluation:** Consider biopsy if hematologic abnormalities are present.
4. **Imaging Follow-Up:** MRI brain (if neurologic symptoms arise) and close surveillance for visceral metastases.

**Conclusion:**
The findings are most consistent with metastatic recurrence of malignant melanoma. Further histopathological confirmation and multidisciplinary evaluation are essential to guide treatment (e.g., immunotherapy, targeted therapy).

R9 Provide primary and differential diagnosis based on the report with clinical history.

Gender:male Age:22

**Inspecti on items:**PET/CT (body including head).

**check on medication:** ^18^ Fluoro-deoxyglucose (^18^F -FDG)

**clinical diagnosi:** 1. Fever (cause to be determined) 2. Bronchitis

**Inspection equipment:** GE Discovery Elite PET/CT

Patient preparation, method of examination and what is seen on examination:

Fasting blood glucose: 5.0 mmol/L, IV 18F-FDG followed by sedentary rest. Oral 1.2% iohexol 1000 ml filled the gastrointestinal tract. 85 minutes after drug injection supine position arms up holding the head, perform body (from the base of the skull to the soles of the feet)+ Cranial PET/CT: a total of 15 beds were acquired, 2 minutes/bed, 3D-TOF acquisition, OSEM reconstruction, CT attenuation correction; low-dose CT scanning on the same machine, layer thickness of 3.3 mm. fusion imaging of PET, CT.

Lymphatic system: Multiple hypermetabolic lymph node shadows in bilateral cervical regions Ⅰ, Ⅱ, Ⅲ, bilateral supraclavicular regions, bilateral axillae, mediastinal regions Ⅱ, Ⅳ, and right pulmonary hilar, hepatoportal region, retroperitoneum, mesenteric region and bilateral iliac blood vessels, SUVmax 1.8-21.7, some of them are slightly enlarged, and the larger ones are located in right supraclavicular and mesenteric regions, with sizes of about 22 mm× 9mm and 21 mm× 7mm, with clear densities and uniform boundaries. The density was homogeneous and the borders were clear. The spleen was small, with smooth margins, no abnormal density in the splenic parenchyma, and metabolism was normal.

Head and neck: There were no abnormalities in the soft tissues of the head and neck and the bone structure and metabolism of the skull and cervical vertebrae. The morphology of bilateral cerebral hemispheres was as normal, metabolic distribution was symmetrical, metabolism and density of cerebral gray-white matter areas in each lobe were not abnormal, basal ganglia were clear, ventricles and cerebral pools were normal, and the midline structure was centered. The cerebellar hemispheres on both sides were symmetrical, with no abnormalities in density and metabolism. The metabolism and structure of the nose, pharynx and parapharyngeal space were not abnormal. Metabolism and density of submandibular gland and parotid gland on both sides were normal. The vocal cords were symmetrical bilaterally, and there were no abnormalities in the metabolism and structure of the larynx. No abnormal density and metabolic shadows were seen in the bilobed thyroid gland.

Thorax: The thorax is symmetrical, with no abnormalities in the soft tissue and bony structure of the chest wall, and normal density and metabolism. Bilateral pleura was not smooth, metabolism was not abnormal, and no pleural effusion was seen. Lung fields were clear, with no abnormal density or hypermetabolic shadows. The mediastinum was centered and the trachea and bronchi were patent. The cardiac shadow was not large, the size and morphology of each atrium was in the normal range, the pericardial thickness was normal, there was no sign of pericardial effusion, and there was no abnormality in the ascending aorta and the branches of the major vessels. No abnormal calcification was seen in the coronary arteries.

Abdomen: no abnormalities in the soft tissue and bony structure of the abdominal wall, normal density and metabolism. There is no fluid accumulation in the abdominal cavity. Gastric filling was acceptable, the gastric wall was not thick, no wall nodules, and there was no abnormality in the metabolism of the gastric wall. Liver morphology, location and metabolic distribution were not abnormal, with smooth margins and normal proportions of the hepatic lobes; diffuse hypodensity of the hepatic parenchyma, with an average CT value of about 37 HU, a calcified focus in the S4 segment of the liver, no dilatation of the intrahepatic bile ducts, and a normal porta hepatis structure. The gallbladder was not enlarged, the wall was not thick, no positive stones were seen in the gallbladder, and there was no dilatation of the extrahepatic bile ducts or common bile duct. The pancreas had no abnormalities in location, morphology and metabolism, with clear contours, uniform density of pancreatic parenchyma, no abnormal density, no dilatation of pancreatic ducts, and clear peripancreatic fat interstitium. The morphology, density and metabolism of bilateral adrenal glands showed no abnormality. The morphology and density of both kidneys were not abnormal, and there was no dilatation of the bilateral renal pelvis and ureter, and no abnormal contrast media retention was seen in them. Some of the colonic intestinal tubes showed physiologic uptake shadows.

PELVIC: The pelvic wall has no abnormal soft tissue or bony structure, with normal density and metabolic shadows. There were no signs of fluid accumulation in the pelvis. The bladder was poorly filled with no wall thickening, no abnormal metabolism was seen, and no positive stones were seen in the bladder. The prostate was of normal size with no abnormal density or metabolic distribution. The fat interstitium in the pelvis was clear. Some rectal intestines in the pelvis were also seen as physiologic regurgitant shadows.

Extremities: no abnormalities in the soft tissue and bone structure of the extremities, no abnormal densities or hypermetabolic shadows.

Hepatic blood pool SUVmax 2.9; mediastinal blood pool SUVmax 2.2.

Brief history: 3 weeks ago, the patient had fever without obvious cause, up to 38.5 ℃, occasional cough, no cough sputum, the local clinic, given medication (specifics are not known), the body temperature can fall, down to 37 ℃, maintained 1-2 days after the rise again, the highest to 38 ℃, in Luquan District, Copper Township, Central Health Center, the examination of the blood: white blood cell count ↓ 3.18 * 109 / L, absolute value of neutrophils ↓ 1.66 * 109 / L, absolute value of lymphocytes ↓ 1.05 * 109 / L. C-reactive protein <10mg / L. Chest CT scan: chest CT scan: absolute value of lymphocytes ↓ 1.05 * 109 / L. Neutrophil absolute value ↓ 1.66*109/L, lymphocyte absolute value ↓ 1.05*109/L. C-reactive protein <10mg/L. Chest CT scan: double lung texture increase, disorder, double lung door clearer. Imaging opinion: 1. Bronchitis. Give moxifloxacin sedation, oral "montelukastna tablets", "roxithromycin" cough, anti-infection treatment. The patient's condition did not improve after treatment, and he still had intermittent fever, and was admitted to our hospital 10 days ago. On admission, leukocytes ↓2.6 (3.5-9.5), neutrophils ↓1.38 (1.8-6.3), lymphocytes ↓0.92 (1.1-6.2), sedimentation rate ↑16 (0-15), C-reactive protein ↑12.71 (0-6), and all male tumors (-). Influenza virus antigen, new coronavirus nucleic acid, respiratory pathogen nucleic acid, Borrelia burgdorferi agglutination test, tuberculosis T-SPOT, EBV, and blood bacterial culture (-). Antinuclear antibody 1:100, antinuclear antibody spectrum (-). Thoracic CT of the right lower lobe of the lung with solid micronodules in the anterior basal segment. Ultrasound suggested bilateral supraclavicular region lymph nodes were visible with partially full morphology. Bilateral abdominal and femoral lymph nodes are visible.11-10 Bone puncture (bone marrow) biopsy tissue: myeloproliferative hypoplasia (approximately 50%), granulocyte-red ratio is approximately normal, granulocyte lineage cells of all stages are visible, with predominance of cells of intermediate to young and lower stages, eosinophils are scattered, erythrocytes of all stages are visible, with predominance of red blood cells of intermediate to late juvenile stages and lower stages, and there are quite a few megakaryocytes, with predominance of lobulated nuclei. Lymphocytes and plasma cells are scattered. Please combine with clinical and other examinations to further determine the diagnosis. Immunohistochemical staining: CD61 (megakaryocytes+ ), CD71 (erythroid cells+ ), MPO (granulocytes+ ), CD34 (-), CD3 (few cells+ ), CD20 (few cells+ ). Special stains: reticular fibers (grade 0), Masson (-), PAS (+ ).

Purpose of examination: Cause of fever to be investigated. (Fever to be investigated)

Inspection Observations:

1 Multiple hypermetabolic lymph nodes above and below the transverse septum, some of them slightly enlarged, the primary consideration is the possibility of lymphoma, reactive lymphadenitis is not yet excluded, etc. It is recommended that pathologic biopsy of hypermetabolic lymph nodes be confirmed.

2 Fatty liver. Calcified foci in the S4 segment of the liver.

Follow-up: (Right cervical lymph node) proliferative lesion of lymphoid tissue, combined with immunohistochemistry consistent with histiocytic necrotizing lymphadenitis.

Immunohistochemical staining: CD3 (+), CD20 (-), CD21 (residual FDC network), Ki-67 (~70%+), CD43 (+), CD79α (-), MPO (+), CD123 (+), CD68 (+), CD163 (+), CD30 (-).

Thinking:

Okay, I need to write an examination opinion based on the provided PET/CT report and medical history, including a differential diagnosis. Let me start by going through the information step by step.

First, the patient is a 22-year-old male presenting with fever of unknown origin, occasional cough, and leukopenia. He had a history of bronchitis and fatty liver. The PET/CT scan was done to investigate the cause of the fever.

Looking at the PET findings: There are multiple hypermetabolic lymph nodes in various regions (cervical, supraclavicular, axillary, mediastinal, hilar, retroperitoneal, mesenteric, iliac) with SUVmax up to 21.7. Some are slightly enlarged. Other organs like the liver, spleen, head/neck, chest, abdomen, pelvis, and extremities show no significant abnormalities except for a small spleen and some physiologic uptake in the colon and rectum.

The medical history mentions persistent fever, initial treatment with antibiotics without improvement, and lab findings showing low WBC, neutrophils, lymphocytes, elevated CRP, and sedimentation rate. Bone marrow biopsy showed myeloproliferative hypoplasia, and various tests for infections and autoimmune diseases were negative.

Now, the key is to determine what's causing the fever and lymphadenopathy. The differential diagnosis for fever of unknown origin (FUO) with lymphadenopathy and hypermetabolic nodes can include infectious, autoimmune, or malignant etiologies.

Infectious causes: Tuberculosis, atypical mycobacteria, systemic fungal infections, or viral infections like EBV (but EBV was negative here). However, the patient’s tests for TB (T-SPOT), influenza, COVID, and others were negative. The high SUV in lymph nodes might suggest an aggressive infection, but the lack of response to antibiotics complicates this.

Autoimmune diseases: Conditions like sarcoidosis, lupus, or adult-onset Still's disease. The presence of antinuclear antibody (ANA) 1:100 is borderline, and the ANA profile was negative. Sarcoidosis typically presents with bilateral hilar lymphadenopathy, but the PET shows more widespread nodes. Inflammatory markers are elevated, which fits, but more specific tests would be needed.

Malignancy: Lymphoma is a prime consideration here. The hypermetabolic lymph nodes with high SUVmax are concerning for lymphoma, especially Hodgkin's or non-Hodgkin lymphoma. The patient's age and symptoms (fever, night sweats, weight loss?) fit. The bone marrow biopsy showing hypoplasia but no overt malignancy might not rule it out, as lymphoma can sometimes be patchy. The presence of multiple enlarged nodes in various regions and high SUVmax leans towards lymphoma. Other malignancies like metastatic cancer are less likely given the age and presentation.

Other possibilities: Hemophagocytic lymphohistiocytosis (HLH) could present with fever, cytopenias, and hyperferritinemia, but the report doesn't mention ferritin levels. Kikuchi-Fujimoto disease (histiocytic necrotizing lymphadenitis) is another possibility, which can present with fever and lymphadenopathy, usually in younger individuals, but typically has a benign course and might not show such high SUV values.

The fact that infectious and autoimmune workup is negative makes lymphoma more likely. However, the bone marrow biopsy didn't show lymphoma involvement, but that doesn't exclude it as the marrow might not be involved yet. A lymph node biopsy would be crucial for definitive diagnosis.

Differential diagnosis should list the most likely possibilities in order of likelihood based on the data. So:

1. Lymphoma (especially Hodgkin's lymphoma)

2. Sarcoidosis

3. Tuberculosis or atypical infection (despite negative initial tests)

4. Autoimmune disease (e.g., adult-onset Still's disease)

5. Kikuchi-Fujimoto disease

The examination opinion should summarize the findings, suggest the most likely diagnoses, and recommend next steps like lymph node biopsy.

Answer:

**Examination Opinion and Differential Diagnosis**

**Summary of Findings:**
A 22-year-old male presents with a 3-week history of persistent fever (38–38.5°C), leukopenia, and lymphopenia, unresponsive to prior antibiotic therapy. PET/CT reveals widespread hypermetabolic lymphadenopathy involving bilateral cervical, supraclavicular, axillary, mediastinal, hilar, retroperitoneal, and mesenteric regions (SUVmax up to 21.7), with the largest nodes in the right supraclavicular (22×9 mm) and mesenteric regions (21×7 mm). No focal lesions or metabolic abnormalities were detected in other organs. Laboratory findings include elevated CRP (12.71 mg/L) and ESR (16 mm/hr), with negative infectious, autoimmune, and viral workups (e.g., TB, EBV, COVID-19). Bone marrow biopsy demonstrates myeloproliferative hypoplasia but no definitive malignancy.

**Key Differential Diagnoses:**

1. **Lymphoma (Hodgkin’s/Non-Hodgkin’s):**
   - Most likely given widespread hypermetabolic lymphadenopathy (high SUVmax >10), persistent fever, and lack of response to antibiotics.
   - Supraclavicular and mesenteric involvement are common in lymphoma.
   - Bone marrow may not always be involved initially; excisional lymph node biopsy is critical for confirmation.
2. **Tuberculosis/Atypical Mycobacterial Infection:**
   - Lymphadenopathy with necrosis could mimic this, but negative T-SPOT and lack of granulomas on imaging reduce likelihood.
   - Consider PCR or culture of lymph node aspirate if biopsy is performed.
3. **Sarcoidosis:**
   - May present with symmetrical hilar/mediastinal lymphadenopathy, but widespread involvement and high SUVmax are atypical.
   - Elevated ACE levels or biopsy showing non-caseating granulomas would support this.
4. **Adult-Onset Still’s Disease (AOSD):**
   - Fever, leukocytosis (paradoxically absent here), rash, and arthralgias are typical. Elevated ferritin and synovitis would aid diagnosis.
5. **Kikuchi-Fujimoto Disease:**
   - Benign necrotizing lymphadenitis in young adults, but high SUVmax and systemic symptoms are unusual.
   - Requires histopathological confirmation.

**Recommendations:**

- **Excisional lymph node biopsy** (targeting the right supraclavicular node) for histopathology and immunohistochemistry to rule out lymphoma.
- Repeat **bone marrow biopsy** if cytopenias worsen or lymphoma is confirmed.
- Monitor inflammatory markers (ferritin, IL-6) and consider rheumatologic evaluation if autoimmune etiology remains suspected.
- Temporarily manage symptoms with antipyretics while awaiting definitive diagnosis.

**Conclusion:**
Lymphoma is the leading diagnostic consideration. Urgent tissue sampling is required to guide further management.

R10 Provide primary and differential diagnosis based on the report with clinical history.

Gender:female Age:31

**Inspecti on items:**PET/CT (body without head).

**check on medication:** ^18^ Fluoro-deoxyglucose (^18^F -FDG)

**clinical diagnosi:** 1. Aortitis (?) 2. Congenital heart disease

**Inspection equipment:** GE Discovery Elite PET/CT

Patient preparation, methods of examination and what is seen on examination:

Fasting blood glucose: 4.8 mmol/L. Intravenous 18F-FDG was administered followed by sedentary rest. Oral 1.2% iohexol 1000 ml filled the gastrointestinal tract. 105 minutes after injection supine position arms raised above head, body (from base of skull to mid-thigh)+ Head PET/CT: Head PET examination 1× 108 counts, 1 bed. Body PET acquisition of 1 bed, minutes/bed. Minutes after drug injection rows Departmental delayed PET/CT: Acquisition of one bed, minutes/bed. 3D-TOF acquisition, OSEM reconstruction, CT attenuation correction; low-dose CT scanning on the same machine with a layer thickness of 3.3 mm. fusion visualization of PET and CT.

Neck: no abnormalities in sinus and pharynx metabolism and structure. No abnormalities in metabolism and density of submandibular gland and parotid gland on both sides. The vocal cords were not thick bilaterally, and there were no abnormalities in laryngeal metabolism and structure. No abnormal density and metabolic shadows were seen in the bilobed thyroid gland. Multiple hypermetabolic lymph node shadows in bilateral cervical regions Ib, II, and V with SUVmax 1.2-3.6, the large and metabolically high ones were located in the right cervical region II, with a size of about 24mm× 15mm, a CT value of about 66HU, and poorly defined borders. An elliptical hypermetabolic nodal shadow in the left cervical root, adjacent to the middle and lower part of the thyroid gland in the left lobe, SUVmax 3.9, size about 25mm× 21mm× 37mm, density still homogeneous, CT value about 80HU, clear borders.

Chest: postoperative changes in the sternum, symmetrical thorax, no abnormal density or hypermetabolic shadows in the bony structures of the chest wall; smooth pleura bilaterally, no pleural effusion shadows. The texture of bilateral lung fields was clear, and no abnormal density or hypermetabolic shadow was seen. The mediastinum was centered, the trachea and bronchi were patent, and no abnormal enlarged lymph nodes or hypermetabolic shadows were seen in the mediastinum. The cardiac shadow was not large, the size and morphology of each atrium was in the normal range, the pericardial thickness was normal, and there was no sign of pericardial effusion, and there was no abnormality in the ascending aorta and the branches of the major blood vessels. There were no abnormal calcified shadows in the coronary arteries.

Abdomen: no abnormal density or hypermetabolic shadows of the bony structures of the abdominal wall, no signs of fluid accumulation in the abdominal cavity. Gastric filling was acceptable, gastric wall was not thick, and metabolism did not show any abnormality. The liver had no abnormal morphology, position or metabolism, with smooth margins and normal lobe proportions; no abnormal density in the liver parenchyma, no dilatation of the intrahepatic bile ducts, and a normal portal structure. The gallbladder was not enlarged, the wall was not thick, there were no positive stone shadows in the gallbladder, and the common bile duct was not dilated. The pancreas did not show any abnormality in morphology, location and metabolism, with clear contours, no abnormal density shadows in the pancreatic parenchyma, no dilatation of the pancreatic ducts, and clear peripancreatic fat interstitium. The spleen was small, with smooth margins, and no abnormal density or hypermetabolic shadows were seen in the splenic parenchyma. The morphology, density and metabolism of bilateral adrenal glands were not abnormal. The morphology and density of both kidneys were not abnormal, and the bilateral renal pelvis and ureters were not dilated, with no abnormal contrast material retention. The retroperitoneum showed no abnormal enlargement or hypermetabolic lymph nodes. Some of the intestines in the abdominal cavity showed physiologic uptake shadows.

Pelvis: No abnormal density or hypermetabolic shadows of the bony structures of the pelvic wall, no signs of fluid accumulation in the pelvis. There were no positive stone shadows in the poorly filled bladder or bladder. An oval hypermetabolic hypodense shadow in the right adnexal region, SUVmax 2.4, size about 17mm× 10mm, clear border. No metabolic abnormalities were seen in the uterus or left adnexal region. The fat interstitium in the pelvis was clear, and there were no abnormal enlargement or hypermetabolic lymph node shadows in the pelvis or bilateral inguinal region. Some of the intestines in the pelvis showed physiologic uptake shadows.

No vascular anomalous metabolic increase was seen within the scan.

Diffuse metabolic mild increase in skeletal muscle of the trunk and extremities within the scan, SUVmax 1.9, no density or morphologic abnormalities were seen.

Brief history: 2 days ago, the patient found that the blood pressure of the right upper limb could not be measured, and there was occasional weakness of the upper limb after activity, no temporal headache and loss of vision, no dizziness, black haze, no headache, fever, no nausea, abdominal pain, no claudication, no recurrent mouth ulcers, no alopecia and other discomforts. In our hospital, arterial ultrasound suggested bilateral cervical vessels + subclavian artery ultrasound: suggesting that the right subclavian artery may steal blood (the beginning of the display is not clear); the right vertebral artery spectral morphology is abnormal; the bilateral common carotid artery did not see obvious anomalies; bilateral internal carotid arteries and bilateral external carotid arteries did not see obvious anomalies; the left side of the vertebral artery did not see obvious anomalies; the left side of the subclavian artery did not see obvious anomalies (the beginning of the probe is not satisfactory); bilateral common carotid arteries did not see obvious abnormalities; the left side of the subclavian artery did not see obvious abnormalities (the beginning of the probe is not satisfactory). (unsatisfactory exploration); bilateral internal jugular veins; bilateral axillary, brachial, radial, and ulnar arteries ultrasound: right axillary artery, right brachial artery, right radial artery, and right ulnar artery with decreased blood flow velocity (further examination recommended); left axillary artery and left brachial artery with no obvious abnormality; left ulnar artery and left radial artery with no obvious abnormality; cardiac ultrasound: congenital heart disease; ruptured aortic sinus aneurysm Postoperative; ventricular septal defect (perimembranous portion); mitral valve prolapse with little-moderate regurgitation (consider possible mitral annular separation); small amount of regurgitation in the aortic valve; small amount of regurgitation in the tricuspid valve; poorly visualized right subclavian artery origin (consider stenosis) Variation of the right subclavian artery origin? Further examination is recommended). Laboratory tests: anti-streptococcal hemolysin O 528.00↑ U/mL, sedimentation rate 23, anti-nuclear antibody positive 1:100, the remaining anti-dsDNA antibody, anti-phospholipid antibody two, anti-nuclear antibody spectrum, ACCP + anti-AKA, vasculitis screening 1, HLA-B27, lupus anticoagulant two did not see any abnormality. tT3 4.990↑ nmol/L, tT4 253.800 nmol/L, fT4 253.800 ↑ nmol/L,FT3 22.450↑ pmol/L,FT4 85.070↑ pmol/L,TSH 0.008↓ μIU/mL,TPOAb 12.010 IU/mL,TRAb 11.780↑ IU/L.

Past history: aortic aneurysm repair + patent foramen ovale repair for congenital heart disease 3 years ago.

Purpose of examination: aortitis, left carotid root mass to be investigated.

Inspection Observations:

1 There were no metabolic abnormalities in the vascular traveled areas within the whole body scan, and there were no clear imaging signs of active vasculitis.

Slightly elevated diffuse metabolism of the trunk and skeletal muscles of the extremities is considered to be due to hyperthyroidism in conjunction with the history.

2 An oval hypermetabolic mass at the base of the left neck is considered a possible inflammatory lymph node, and a pathologic biopsy is recommended to exclude others.

Multiple high and slightly high metabolic lymph nodes in both necks, with the right cervical region II lymph nodes increasing in number, size and metabolism, are also considered to be inflammatory lymph nodes, and biopsy is recommended to confirm the pathology when necessary.

3 Oval hypermetabolic hypodense shadow in the right adnexal region, consider physiologic uptake.

(Read) Our follow-up: (Hypoechoic mass behind the cervical vessels at the base of the left neck) Punctured tissue combined with immunohistochemical staining: consistent with thymic tissue. Immunohistochemical staining: CD3 (+ ), CD20 (focally+ ), CD21 (-), CD34 (-), Ki-67 (80%+).

Thinking:

Okay, let's try to tackle this. The user has provided a detailed PET/CT report along with the patient's medical history and wants an examination opinion with differential diagnoses. First, I need to understand the key findings from the report.

The patient is a 31-year-old female with a history of congenital heart disease, including postoperative aortic sinus aneurysm and ventricular septal defect. She also had a patent foramen ovale repair. The current clinical diagnosis includes aortitis (questionable) and a left carotid root mass. The PET/CT shows hypermetabolic lymph nodes in the cervical regions, a right adnexal hypodense shadow, and some lab abnormalities like elevated anti-streptolysin O, positive ANA, and thyroid function tests indicating possible hyperthyroidism.

Starting with the hypermetabolic lymph nodes. The SUVmax values are moderate (up to 3.9). Possible causes could be reactive lymphadenopathy, infection (like tuberculosis), autoimmune (given positive ANA and history of aortitis), or lymphoma. The patient's elevated ASO suggests a recent streptococcal infection, which might cause reactive nodes. Autoimmune conditions like Takayasu arteritis (given aortitis suspicion) could also lead to lymphadenopathy.

The right adnexal lesion with SUVmax 2.4 could be a benign ovarian cyst, endometrioma, or a neoplasm. Given the SUV is not very high, maybe benign, but needs follow-up.

The elevated thyroid hormones (TSH low, T3/T4 high) suggest hyperthyroidism. The presence of TRAb elevated might indicate Graves' disease. However, the patient's other autoimmune markers (positive ANA) could suggest an overlap with another autoimmune condition.

The patient's history of congenital heart disease and previous surgeries might have some bearing, but the current issues seem more related to possible autoimmune or inflammatory processes. The aortitis in question could be part of a systemic vasculitis like Takayasu or giant cell arteritis, though the age is a bit young for GCA.

Differential diagnoses need to include:

1. **Autoimmune Aortitis (Takayasu Arteritis):** Given the history of possible aortitis, elevated inflammatory markers (sed rate), and positive ANA. The vascular ultrasound findings of subclavian artery steal and stenosis could support this.

2. **Reactive Lymphadenopathy:** Due to streptococcal infection (high ASO) or other infections. The lymph nodes' SUV is not very high, so maybe reactive.

3. **Lymphoma:** Though SUVmax is moderate, lymphoma can present with lymphadenopathy. However, the lack of other B symptoms (fever, weight loss) might make this less likely.

4. **Hyperthyroidism/Graves' Disease:** Elevated thyroid hormones and TRAb. Needs evaluation by endocrinology.

5. **Ovarian Pathology:** The right adnexal lesion could be benign (cyst) versus neoplastic. Further imaging or follow-up needed.

6. **Systemic Lupus Erythematosus (SLE):** Positive ANA and possible multi-system involvement. However, other antibodies (dsDNA) were negative, so less likely.

7. **Infectious Aortitis:** Though less likely given the negative cultures mentioned (if any were done), but could be considered if there's suspicion of infection.

The patient's presentation seems to be a mix of autoimmune and possible infectious etiologies. The combination of aortitis, lymphadenopathy, and hyperthyroidism suggests a systemic process. Takayasu arteritis is a strong consideration given the vascular findings and aortitis. The hyperthyroidism might be a separate issue like Graves', but could also be part of an autoimmune overlap.

Need to recommend further tests: CT angiography for vasculitis, thyroid ultrasound, biopsy of the hypermetabolic lymph nodes if persistent, and endocrine evaluation for hyperthyroidism. Also, consider infectious workup if there's ongoing suspicion.

Answer:

**Examination Opinion and Differential Diagnosis**

**Patient Summary:**
A 31-year-old female with a history of congenital heart disease (postoperative aortic sinus aneurysm repair, ventricular septal defect, mitral valve prolapse) and recent concerns for aortitis and a left carotid root mass, underwent PET/CT evaluation. Key findings include:

1. **Hypermetabolic cervical lymph nodes** (SUVmax 1.2–3.9), notably a 25mm×21mm×37mm node adjacent to the left thyroid lobe.
2. **Right adnexal hypodense lesion** (SUVmax 2.4, 17mm×10mm).
3. **Lab abnormalities**: Elevated anti-streptolysin O (528 U/mL), positive ANA (1:100), and hyperthyroidism (↓TSH, ↑FT3/FT4, ↑TRAb).
4. Vascular ultrasound findings suggestive of right subclavian artery stenosis/steal phenomenon.

**Key Clinical Considerations:**

1. **Suspected Aortitis/Autoimmune Vasculitis**:
   - **Takayasu Arteritis**: Fits the clinical picture of aortitis, vascular stenosis (subclavian artery), elevated inflammatory markers (ESR 23), and young age. PET findings of vascular inflammation (not explicitly stated) would further support this.
   - **Infection vs. Autoimmune**: Elevated ASO suggests prior streptococcal infection, but persistent inflammation and systemic features favor autoimmune etiology.
2. **Cervical Lymphadenopathy**:
   - **Reactive Lymphadenopathy**: Likely secondary to infection (e.g., streptococcal) or autoimmune activity.
   - **Lymphoma**: Moderate SUVmax and nodal size warrant consideration, though absence of B symptoms (fever/weight loss) lowers suspicion.
   - **Autoimmune-Associated Lymphoproliferation**: Seen in SLE or vasculitis, though anti-dsDNA/other antibodies were negative.
3. **Hyperthyroidism**:
   - **Graves’ Disease**: Elevated TRAb supports this diagnosis. Thyroid ultrasound and endocrine consultation are needed.
4. **Right Adnexal Lesion**:
   - **Benign Cyst/Physiologic Uptake**: Low SUVmax favors benignity.
   - **Neoplasm**: Less likely but requires follow-up imaging (pelvic ultrasound/MRI).
5. **Systemic Autoimmune Overlap**:
   - Coexistence of autoimmune thyroiditis (Graves’) and vasculitis (Takayasu) is rare but possible.

**Differential Diagnosis:**

1. **Takayasu Arteritis** (primary): Explains aortitis, vascular stenosis, and systemic inflammation.
2. **Post-Streptococcal Reactive Arthritis/Inflammation**: Elevated ASO and lymphadenopathy.
3. **Lymphoproliferative Disorder** (e.g., lymphoma): Requires nodal biopsy if nodes persist.
4. **Graves’ Disease with Autoimmune Overlap**: Hyperthyroidism + vasculitis.
5. **Ovarian Benign Cyst vs. Neoplasm**: Imaging follow-up recommended.

**Recommendations:**

1. **Vascular Imaging**: CT/MR angiography to assess aortic/vascular inflammation and confirm Takayasu arteritis.
2. **Lymph Node Biopsy**: For persistent/dominant cervical nodes to exclude malignancy.
3. **Thyroid Evaluation**: Ultrasound and endocrine consultation for Graves’ disease management.
4. **Pelvic Ultrasound**: Characterize the right adnexal lesion.
5. **Infectious Workup**: Rule out chronic infections (e.g., TB) given lymphadenopathy.
6. **Rheumatologic Follow-Up**: Monitor for vasculitis progression and consider immunosuppression if confirmed.

**Conclusion:**
The findings suggest a multifactorial process, with autoimmune vasculitis (Takayasu) and Graves’ disease as leading diagnoses. Further targeted imaging and biopsies are critical to refine management.
